# The Stereoselectivity of Neighboring Group-Directed Glycosylation Is Concentration-Dependent

**DOI:** 10.1021/jacs.4c14402

**Published:** 2025-02-05

**Authors:** Pallabita Basu, David Crich

**Affiliations:** a Department of Pharmaceutical and Biomedical Sciences, University of Georgia, 250 West Green Street, Athens, Georgia 30602, United States; b Complex Carbohydrate Research Center, University of Georgia, 315 Riverbend Road, Athens, Georgia 30602, United States; c Department of Chemistry, University of Georgia, 302 East Campus Road, Athens, Georgia 30602, United States

## Abstract

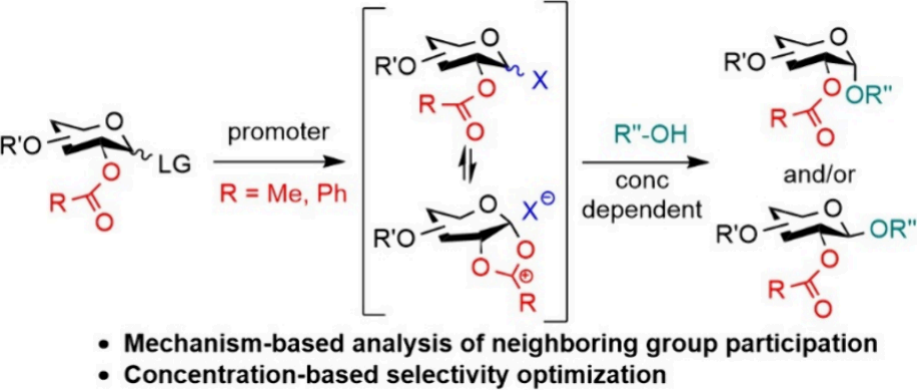

The formation of 1,2-*trans*-glycosides taking advantage of neighboring group participation by stereodirecting esters at the 2-position of glycosyl donors is widely held to be a robust and reliable protocol. Examples abound, however, of cases in which less-than-perfect selectivity is obtained, causing practitioners to survey different esters or resort to alternative strategies in the quest for optimal selectivities and yields. Consideration of the mechanism of neighboring group participation and in particular of the competing process of S_N_2-like glycosylation with activated covalent donors leads to the hypothesis that in cases of imperfect selectivity, more careful attention to reaction concentration and stoichiometry may be beneficial. Three case studies are presented to demonstrate the concentration dependence of neighboring group-directed glycosylation reactions targeting the formation of both 1,2-*trans*-equatorial and 1,2-*trans*-axial glycosides. Higher concentrations, whether achieved through increased acceptor:donor stoichiometry or through increased concentration at a fixed stoichiometry, mostly lead to erosion of 1,2-*trans*-selectivity as the competing S_N_2-like reaction with the covalent donors becomes increasingly important. These observations underline the importance of a rational, mechanism-based approach to glycosylation in general and more importantly suggest a simple approach to enhancing 1,2-*trans*-selectivity in neighboring group-directed glycosylation reactions displaying less-than-perfect 1,2-*trans*-selectivity, namely, moving to a different concentration regime.

## Introduction

**Scheme 1:**
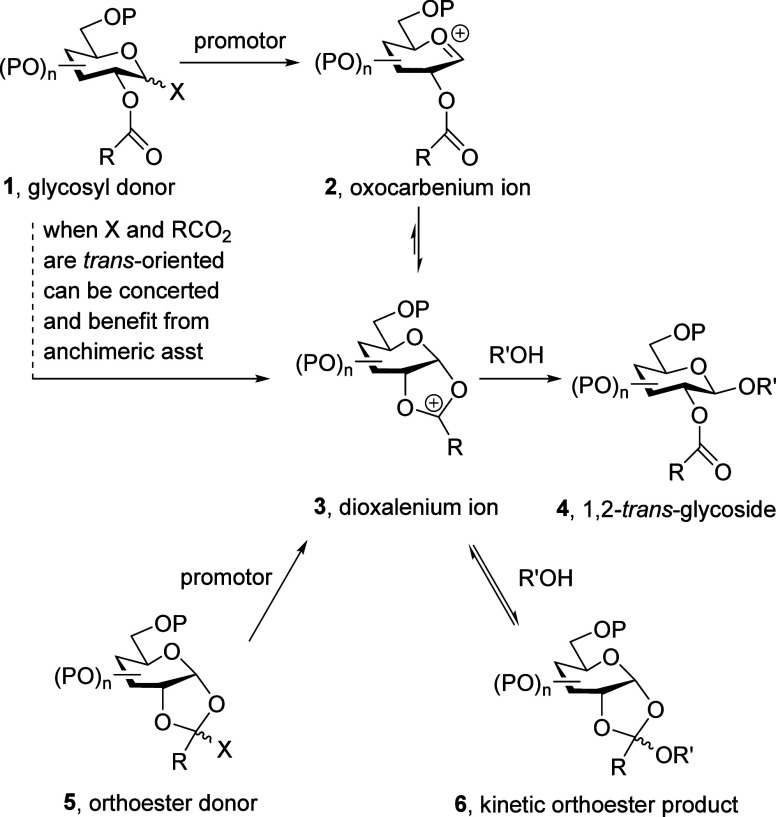
Classical Mechanism for Neighboring Group-Directed Glycosylation Illustrated for the Formation of a 1,2-*trans*-Equatorial Glycopyranoside

Since the introduction of the Koenigs–Knorr reaction at the beginning of the 20th century,^1^ neighboring group participation, recognized as such by Frush and Isbell in 1941^2^ and introduced to mainstream organic chemistry by Winstein and Buckles in 1942,^3^ has served as a robust, reliable method for the preparation of the 1,2-*trans*-glycosides, typified by the β-d-glucopyranosides and the α-d-mannopyranosides. The concept has been reviewed many times^4−17^ and classically has been mostly considered as taking place via the promoter-assisted departure of a leaving group from the anomeric position of a glycosyl donor **1** to give an intermediate glycosyl oxocarbenium ion **2**. The neighboring ester then cyclizes onto the oxocarbenium ion and generates a *cis*-fused dioxalenium ion **3**. In the final step of the reaction, the dioxalenium ion is opened S_N_2-wise to give the 1,2-*trans*-glycoside **4**, as illustrated for 1,2-*trans*-equatorial glycoside formation in Scheme 1. When the initial leaving group and the participating ester are themselves 1,2-*trans*-oriented, the formation of the *cis*-fused dioxalenium ion may take place in a concerted manner (Scheme 1), sometimes with kinetic acceleration of the process or anchimeric assistance.^18−21^ Bona fide examples of actual rate increases due to anchimeric assistance however are rare and the accelerations typically modest.^22−33^ Alternatively, in the ortho-ester glycosylation method, the stereodirecting intermediate dioxalenium ion **3** may be accessed from an ortho ester-type donor **5** (Scheme 1).^9,11,34^ Finally, kinetic attack of the acceptor alcohol on the intermediate dioxalenium ion **3** has been demonstrated to give rise to orthoesters **6**, which are readily isolable when formed under basic conditions and subsequently rearrange to the thermodynamic glycosides (Scheme 1).^35−41^

**Scheme 2:**
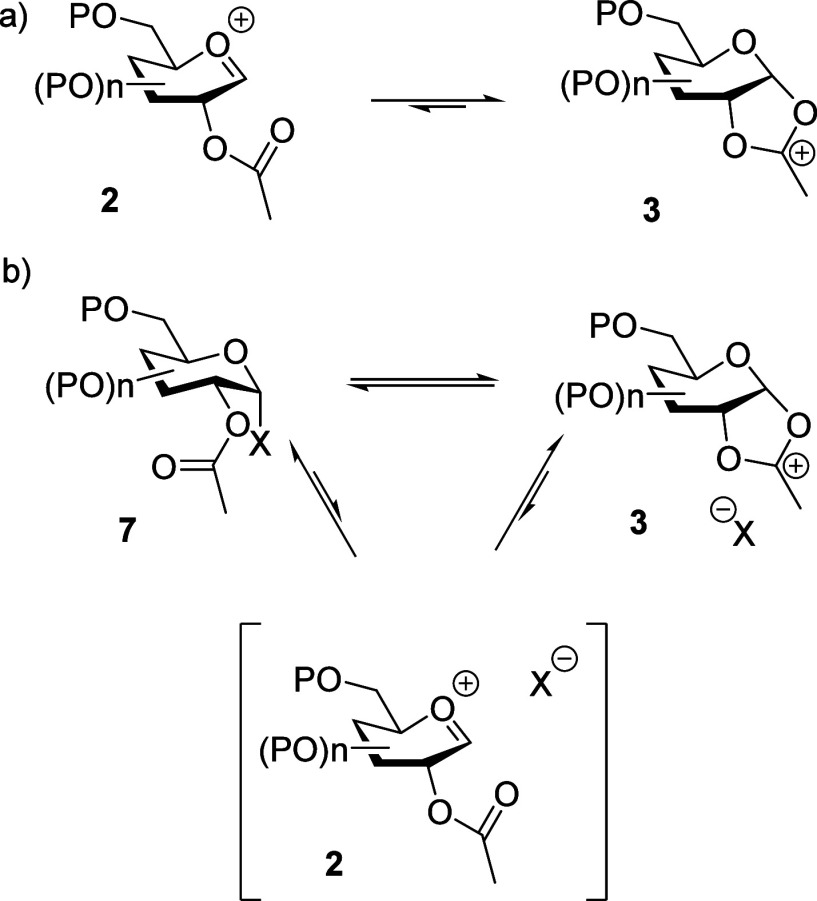
(a) Classical and (b) Non-classical Equilibria in Neighboring Group-Directed Glycosylation

Except in the rare cases of concerted dioxalenium ion **3** formation directly from glycosyl donor **1**, this classical picture of stereodirecting neighboring group participation is clouded by its reliance on the formation of oxocarbenium ion **2**. This is because the existence of glycosyl oxocarbenium ions in the condensed phase in the presence of the obligatory counterion has been heavily disputed since the NMR spectroscopic demonstration of the glycosyl triflates^42,43^ and the accumulation of a substantial body of kinetic evidence pointing to the bimolecular nature of simple (non-neighboring group-directed) glycosylation reactions.^44−53^ Indeed, despite the recent characterization of 2-deoxy- and 2-bromo-2-deoxy-glycosyl oxocarbenium ions in superacid media,^51,54,55^ that of more “typical” glycosyl oxocarbenium ions carrying a C–O bond at the 2-position has yet to be reported in any condensed phase. A more accurate picture of neighboring group-directed glycosylation, rather than relying on the simple equilibration of the oxocarbenium ion **2** with the more stable (and readily observable^41,54,56,57^ and isolable^17,58^) dioxalenium ion **3**, must therefore take into account the presence of the counterion and the stabilization of the oxocarbenium ion in the form of an activated covalent donor **7**.^59^ In other words, the true equilibrium influencing neighboring group participation, whatever the nature of the counterion,^52^ is that between the activated covalent donor **7** and the dioxalenium **3** (Scheme 2).^57,60^ This realization prompted us to suggest that “stereodirecting participation by a vicinal ester through a five-membered fused dioxalenium ion is a relatively borderline event in the presence of the triflate or other less nucleofugal counterions”.^61^ This realization, borne out by subsequent work from the Codée and Woerpel laboratories,^62^ in turn suggests that the stereochemical leakage often observed^40,56,57,62−94^ in neighboring group-directed glycosylation may be due to competing S_N_2-like glycosylation of the acceptor by equilibrating axial and equatorial forms of the activated covalent donor **7** and not to trapping of the glycosyl oxocarbenium ion **2** by the acceptor alcohol or to thermodynamic equilibration of the products. The incidence of stereochemical leakage can be gauged from inspection of Barresi and Hindsgaul’s 1995 survey of the 734 glycosylation reactions published in the year 1994.^95^ Of the 372 reactions reported, using what would typically be considered protecting groups affording neighboring group participation at the 2-position, approximately 5% were described as giving mixtures of anomers, which is an almost certain under-representation of the true extent of the phenomenon.

**Scheme 3:**
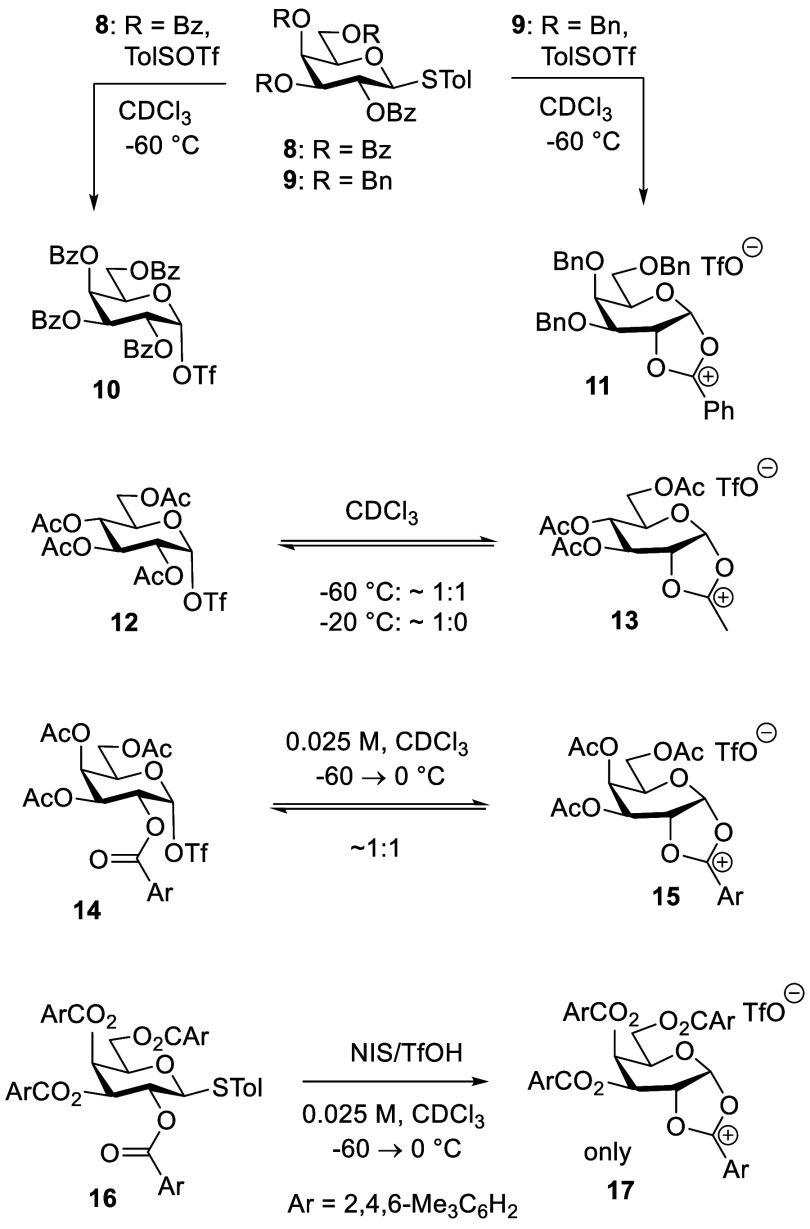
Protecting Group and Temperature-Dependent Nature of the Glycosyl Triflate–Dioxalenium Ion Equilibrium

The borderline nature of the neighboring group participation mechanism for glycosylation is best illustrated by variable temperature NMR studies from the Huang and Williams groups, which revealed the dioxalenium ion:covalent triflate equilibrium to be dependent on the nature of the remote protecting groups and on temperature.^56,57^ Thus, Huang and co-workers found that a tetra-*O*-benzoyl galactopyranosyl donor **8** cleanly gave galactosyl triflate **10** on activation at −60 °C in deuterochloroform at an unspecified concentration, whereas the corresponding 3,4,6-tri-*O*-benzyl-2-*O*-benzoyl donor **9** gave only dioxalenium ion **11**. The clear influence of the protecting groups on the structure of the product in these reactions was attributed to destabilizing effect of the more strongly electron-withdrawing benzoate esters on the positively charged dioxalenium ion. With the corresponding tetra-*O*-acetylglucopyranose system, the authors observed a temperature-dependent equilibrium consisting of a 1:1 mixture of the glycosyl triflate **12** and the fused dioxalenium ion **13** at −60 °C, but shifting to strongly favor the covalent triflate at −20 °C.^56^ Similarly, Williams and co-workers observed a 1:1 mixture of the fused dioxalenium ion **15** and the glycosyl triflate **14**, on activating a 3,4,6-tri-*O*-acetyl-2-*O*-mesitoyl galactosyl donor in deuterochloroform at −60 °C followed by warming to 0 °C, whereas the tetra-*O*-mesitoyl donor **16** gave only the fused dioxalenium ion **17** under the same conditions (Scheme 3).^57^

If, as early studies suggest, neighboring group-directed glycosylation reactions tend toward unimolecular kinetics with the rate independent of the acceptor concentration,^4,9,13,17^ and competing direct displacement of the leaving group by the acceptor is S_N_2-like,^53,96^ as recognized by some early investigators,^97,98^ it should be possible to vary stoichiometry and concentration to improve stereoselectivity in under-performing neighboring group-directed glycosylation reactions. With this in mind, we selected a set of glycosylation reactions that were reported to proceed with less than optimal selectivity and, in each case, studied the influence of stoichiometry and concentration on selectivity. Additionally, in some cases, we investigated the effect of the promoter and/or the reaction temperature on stereoselectivity. We report that poorly selective neighboring group-directed glycosylation reactions targeting both 1,2-*trans*-equatorial and 1,2-*trans*-axial selectivity are indeed concentration-dependent, with lower concentrations mostly favoring the desired 1,2-*trans*-selectivity. Higher concentrations, on the other hand, are more likely to lead to the erosion of selectivity as the more concentration-dependent bimolecular displacement of the leaving group from the covalent donor increases in importance.

## Results

### Synthesis of Glycosyl Donors and Acceptors

All glycosyl donors and acceptors employed in this study were prepared by literature methods or variations on them, as reported in the Supporting Information.

### System 1: Glycosylation of a d-Glucosamine-3-OH-Based Acceptor by Enantiomeric d- and l-Fucopyranosyl Bromides

**Scheme 4:**
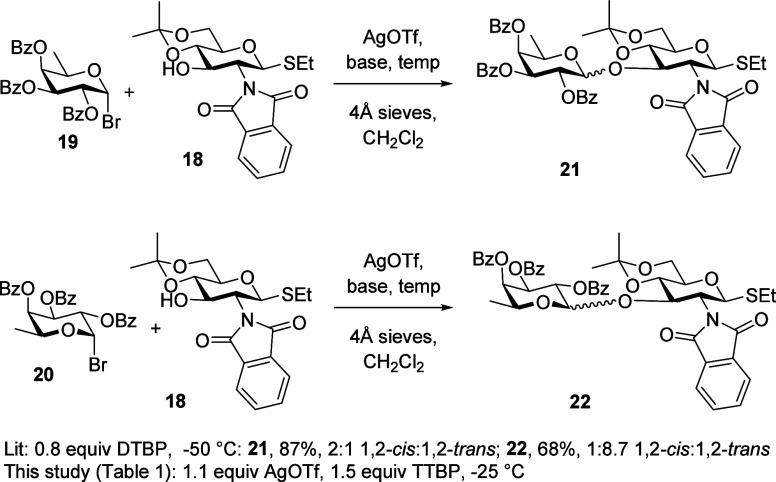
Glycosylation of the D-Acceptor **18** by the D- and L-Donors **19** and **20**

For the initial study, we selected the glycosylation of the d-glucosamine-based acceptor **18** by the d- and l-fucopyranosyl bromides **19** and **20** described by Spijker and van Boeckel in their seminal study^63^ extrapolating on Paulsen’s initial application^99^ of the concept of double stereodifferentiation and stereochemical matching and mismatching^100,101^ to carbohydrate chemistry.^102,103^ Spijker and van Boeckel found that coupling of the d-configurated donor **19** with an unspecified amount of the d-acceptor **18** at −50 °C in dichloromethane in the presence of 0.8 equiv of 2,6-di-*tert*-butylpyridine (DTBP) and molecular sieves with activation by an unspecified amount of silver triflate gave the d,d-disaccharide **21** in 87% overall yield as a 2:1 1,2-*cis*:1,2-*trans*-mixture. They concluded the poor selectivity to be the result of an unmatched pair of reactants, which was reinforced by the observation that replacement of the d-donor **19** by its l-enantiomer **20** gave the l,d-disaccharide **22** in 68% overall yield as a 1:8.7 1,2-*cis*:1,2-*trans*-mixture, owing to better stereochemical matching (Scheme 4).

Lacking sufficient information to reproduce the literature conditions, we elected to work with 1.1 equiv of AgOTf as the promoter, a 1:1 donor:acceptor stoichiometry, and 1.5 equiv of tri-*tert*-butylpyrimidine (TTBP) as the base operating at −25 °C, and varying the concentration of the reaction mixture as specified in Table 1.

**Table 1:** Concentration Dependence of the Coupling of Acceptor **18** with Donors **19** and **20**^a^^,^^b^

entry	donor	donor conc (M)	donor:acceptor stoichiometry	product	yield (%)^c^	1,2-*cis*:1,2-*trans* ratio^d^
1	**19**	0.033	1:1	**21**	76	1:6.4
2	**19**	0.066	1:1	**21**	72	1:3.7
3	**19**	0.1	1:1	**21**	68	1:3.0
4	**19**	0.2	1:1	**21**	74	1:2.9
5	**19**	0.3	1:1	**21**	71	1:3.0
6	**20**	0.033	1:1	**22**	89	>95% 1,2-*trans*
7	**20**	0.2	1:1	**22**	83	>95% 1,2-*trans*
8^e^	**20**	0.033	1:1	**22**	76	>95% 1,2-*trans*
9^e^	**20**	0.2	1:1	**22**	73	>95% 1,2-*trans*

a All reactions conducted at −25 °C unless otherwise stated.

b All reactions conducted on a scale of 0.15 mmol of acceptor.

c Isolated yields after chromatographic purification.

d Ratios determined by NMR of crude reaction mixtures.

e Conducted at −45 °C.

Inspection of Table 1, entries 1–5, reveals that under the conditions employed the d-acceptor **18** and the d-donor **19** couple with modest selectivity in favor of the 1,2-*trans*-isomer of the product **21**. Moreover, the selectivity is concentration-dependent decreasing from a maximum of 1:6.4 1,2-*cis*:1,2-*trans* at the lowest concentration studied (Entry 1, 0.033 M) to approximately 1:3.0 1,2-*cis*:1,2-*trans* at a donor concentration of 0.1 M (Entry 3), with no significant difference on further increasing the concentration to 0.3 M (Entries 4 and 5). While clearly showing the concentration dependence of the apparently mismatched coupling of **18** with **19**, the anomeric selectivities are nevertheless significantly different from the 2:1 1,2-*cis*:1,2-*trans* ratio reported by the original authors. Table 1 entries 6 and 7 record the selectivities observed on coupling of acceptor **18** with the enantiomeric donor **20** at two concentrations at our standard temperature of −25 °C: in both cases, excellent 1,2-*trans*-selectivity was observed, again in contrast to the 1:8.7 1,2-*cis*:1,2-*trans* ratio reported by the original authors. In an attempt to approach the original conditions more closely, the coupling of **18** and **20** was conducted at the same two concentrations but at the lower temperature of −45 °C, but with no change in selectivity (Table 1, entries 8 and 9).

As a common strategy to improve the selectivity of neighboring group-directed glycosylation reactions is to change the nature of the participating group so as to influence the stability of the intermediate dioxalenium ion, we prepared the corresponding peracetylated d- and l-fucopyranosyl donors **23** and **24** and studied their coupling to acceptor **18** under the same set of conditions giving rise to the d,d-disaccharides **25** and the d,l-disaccharides **26** (Table 2). The overall pattern observed with the peracetylated donors **23** and **24** was the same as that with their perbenzoylated counterparts **19** and **20**. Thus, the mismatched acceptor:donor pair **18** and **23** showed a strong concentration dependence (Table 2, entries 1–5) with the higher selectivities observed at the lower concentrations, while the matched acceptor:donor pair **18** and **24** gave very high selectivities under all conditions studied (Table 2, entries 6–9). Strikingly, however, the mismatched acceptor:peracetylated donor pair **18** and **23** was more concentration dependent than the corresponding mismatched acceptor:perbenzoylated donor pair **18** and **19** (compare Tables 1 and 2, entries 1–5). Thus, excellent selectivity was observed with peracetylated donor **23** at the lowest concentration employed (Table 2, entry 1), whereas the best selectivity observed with perbenzoylated donor **19** was 1:6.4 1,2-*cis*:1,2-*trans*.

**Table 2:**
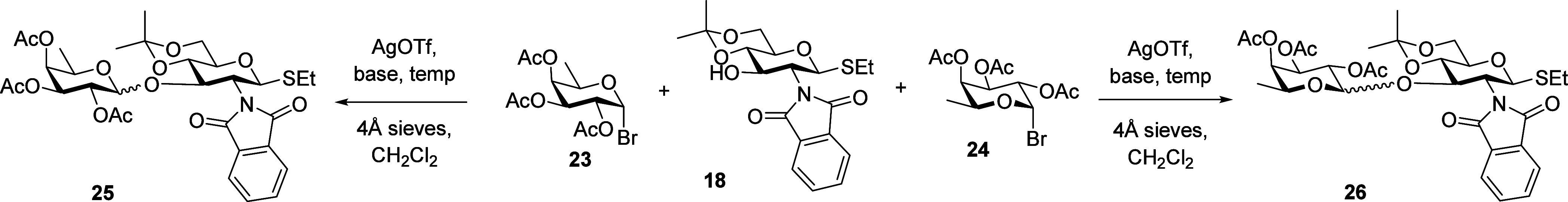
Concentration Dependence of the Coupling of Acceptor **18** with Donors **23** and **24**^a^^,^^b^

entry	donor	donor conc (M)	donor:acceptor stoichiometry	product	yield (%)^c^	1,2-*cis*:1,2-*trans* ratio^d^
1	**23**	0.033	1:1	**25**	79	>95% 1,2-*trans*
2	**23**	0.066	1:1	**25**	77	1:6.9
3	**23**	0.1	1:1	**25**	60	1:3.2
4	**23**	0.2	1:1	**25**	63	1:2.7
5	**23**	0.3	1:1	**25**	57	1:2.4
6	**24**	0.033	1:1	**26**	87	>95% 1,2-*trans*
7	**24**	0.2	1:1	**26**	83	>95% 1,2-*trans*
8^e^	**24**	0.033	1:1	**26**	66	>95% 1,2-*trans*
9^e^	**24**	0.2	1:1	**26**	61	>95% 1,2-*trans*

a All reactions conducted at −25 °C unless otherwise stated.

b All reactions conducted on a scale of 0.15 mmol of acceptor.

c Isolated yields after chromatographic purification.

d Ratios determined by NMR of crude reaction mixtures.

e Conducted at −45 °C.

### System 2: Glycosylation of Diacetone-d-glucofuranose **28** by l-Idopyranosyl Donor **27**

For a second study, we turned to an l-idopyranosyl donor **27** described by Ferro and co-workers to couple with varying temperature-dependent 1,2-*cis*:1,2-*trans* selectivity to a range of simple alcohols and glycosyl acceptors.^77,91^ Donor **27** carries an axial benzoate group at the 2-position intended to enforce formation of the 1,2-*trans*-axial glycoside by participation. We employed diacetone-d-glucofuranose **28** as the acceptor, judging it to be a representative carbohydrate alcohol with moderate nucleophilicity.^104−106^ We varied both the concentration of the donor and the donor:acceptor stoichiometry, activating with *N*-iodosuccinimide (NIS) and TMSOTf (2.0 and 0.2 equiv relative to the donor, respectively) at both −20 and −5 °C (Table 3). These conditions differ from those employed by Ferro and co-workers in that we typically employed less acceptor relative to the donor than the 5 equiv they specify and in that we operated at constant reaction temperatures and a fixed reaction time of 6 h rather than initiating the reactions at −78 °C and warming to −10 to 0 °C over the 3–7 h reported. Despite these differences in conditions and the use of a different acceptor alcohol, we observed poor selectivities consistent with the overall picture presented by the earlier workers. Unlike Ferro and co-workers, who described the isolation of all products after the loss of the 4,6-*O*-acetonide protecting group in the idopyranoside moiety, in our hands, all three acetonides were retained in the product, which we attribute to quenching with a soluble organic base rather than the aqueous sodium thiosulfate employed by the previous workers. As the extent of the change in selectivity with concentration and/or stoichiometry was less in system 2 than in system 1, all experiments were conducted in duplicate, and the data are presented as the mean of the two runs, with the raw data supplied in the Supporting Information.

**Table 3:**
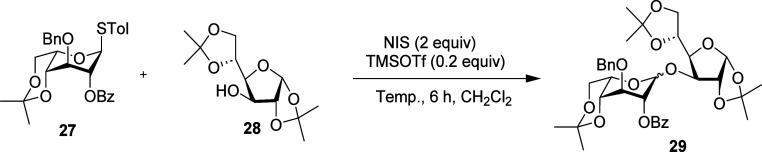
Concentration Dependence of the Coupling of Acceptor **28** with Donor **27**^a^^,^^b^

entry	donor conc (M)	donor:acceptor stoichiometry	temp (°C)	yield (%) **29**^b^^,^^c^	1,2-*cis*:1,2-*trans* ratio^c^^,^^d^
1	0.033	1:0.25	–20	67 ± 6	1:1.87 ± 0.03
2	0.033	1:0.5	–20	74 ± 1	1:1.28 ± 0.02
3	0.033	1:1	–20	85 ± 2	1:1.04 ± 0.04
4	0.2	1:0.25	–20	72.5 ± 3.5	1:1.41 ± 0.09
5	0.2	1:0.5	–20	76.5 ± 5.5	1:1.25 ± 0.05
6	0.2	1:0.83	–20	81 ± 5	1:1.15 ± 0.05
7	0.2	1:1	–20	79 ± 3	1:1.0 ± 0
8	0.2	1:2	–20	77 ± 0	1:1.05 ± 0.05
9	0.3	1:0.5	–20	83 ± 1	1:1.16 ± 0.04
10	0.3	1:1	–20	76.5 ± 7.5	1:1.0 ± 0
11	0.033	1:0.5	–5	76 ± 1	1:1.36 ± 0
12	0.033	1:1	–5	85 ± 2	1:1.27 ± 0.04
13	0.2	1:0.5	–5	84 ± 2	1:1.28 ± 0.02
14	0.2	1:1	–5	77.5 ± 4.5	1:1.04 ± 0.04
15	0.3	1:0.5	–5	84.5 ± 1.5	1:1.2 ± 0

a All reactions conducted on a scale of 0.15 mmol of donor.

b Isolated yields after chromatographic purification.

c Mean of two runs.

d Ratios determined by NMR of crude reaction mixtures.

At a fixed reaction temperature of −20 °C, at each of three different concentrations of donor (0.033 M, Table 3, entries 1–3; 0.2 M, Table 3, entries 4–8; 0.3 M, Table 3, entries 9 and 10), we observed a reduction in 1,2-*trans*-selectivity with increasing amounts of acceptor relative to the donor. At a fixed donor:acceptor stoichiometry, reactions conducted at the higher donor concentrations were generally less 1,2-*trans*-selective. A less extensive study at the higher temperature of −5 °C exhibited the same gross trends, namely, a reduction in 1,2-*trans*-selectivity with increased amounts of acceptor relative to the donor at a fixed donor concentration and reduced 1,2-*trans*-selectivity with increased donor concentration but a fixed donor:acceptor stiochiometry (Table 3, entries 11–15). Overall, the coupling of donor **27** with acceptor **28** was found to be concentration-dependent with reduced stereodirecting neighboring group participation as reflected by reduced 1,2-*trans*-selectivity at the higher concentrations.

### System 3: Synthesis of Protected Laminaribiose by Glycosylation of Acceptor **31** with Donor **30**

The synthesis of β-(1→3)-glucans,^107^ of which laminaribiose is the smallest member, has given rise to numerous descriptions of poor anomeric selectivity despite the application of neighboring group participation.^76,78−80,82,84,94^ For this study, we selected the 2,4,6-tri-*O*-acetyl-3-*O*-benzyl-d-glucopyranosyl *N*-phenyltrifluoroacetmidate^108^**30** as the donor and 1,2,4,6-tetra-*O*-acetyl-β-d-glucopyranose **31** as the acceptor activating with TMSOTf in dichloromethane at −25 °C. With this choice, we approximate the conditions of a study by Ensley and co-workers who employed the trichloroacetimidate analog of **30** as the donor, the α-anomer of **31** as the acceptor, and TMSOTf as the promoter. These earlier workers, however, specified neither donor concentration, stoichiometry, nor temperature, reporting only an 84% yield of a 1:1 1,2-*cis*:1,2-*trans*-mixture of a protected laminaribiose.^94^ We employed a 1:1 stoichiometry of **30** and **31** and 20 mol % of TMSOTf with respect to **30**, operating at 3 different concentrations to give the yields and product ratios reported in Table 4, entries 1–3. In a variation on the theme, we repeated the coupling of **30** and **31** with the same 1:1 stoichiometry in dichloromethane at −25 °C, but this time employing 1.5 equiv of boron trifluoride etherate as a promoter (Table 4, entries 4–9). As with system 2, all experiments in this series were conducted in duplicate, with the data presented as the mean of the runs, and the raw data supplied in the Supporting Information.

**Table 4:**
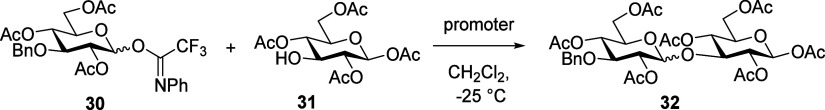
Glycosylation of Acceptor **31** by Donor **30**

entry	donor conc (M)	promoter (equiv)	yield (%) **32**^c^^,^^d^	1,2-*cis*:1,2-*trans* ratio^d^^,^^e^
1	0.033	TMSOTf (0.2)	89.5 ± 2.5	1:1.37 ± 0.07
2	0.2	TMSOTf (0.2)	82 ± 1	1:1.30 ± 0.05
3	0.3	TMSOTf (0.2)	86 ± 1	1:1.24 ± 0.04
4	0.033	BF_3_.Et_2_O (1.5)	69 ± 1	1:3.0 ± 0
5	0.2	BF_3_.Et_2_O (1.5)	76.5 ± 1.5	1:2.88 ± 0.02
6	0.25	BF_3_.Et_2_O (1.5)	74 ± 2	1:2.11 ± 0.01
7	0.3	BF_3_.Et_2_O (1.5)	73 ± 7	1:2.53 ± 0.13
8	0.35	BF_3_.Et_2_O (1.5)	73 ± 2	1:2.62 ± 0.17
9	0.4	BF_3_.Et_2_O (1.5)	70 ± 2	1:2.78 ± 0.08

a All reactions conducted at −25 °C with a 1:1 donor:acceptor stoichiometry.

b All reactions conducted on a scale of 0.12 mmol of acceptor.

c Isolated yields after chromatographic purification.

d Mean of two runs.

e Ratios determined by NMR of crude reaction mixtures.

Under the TMSOTf conditions, as the concentration increased from 0.033 to 0.3 M for the donor at a fixed 1:1 donor:acceptor stoichiometry, the proportion of 1,2-*trans*-product decreased (Table 4, entries 1–3) consistent with the trends observed in systems 1 and 2. With BF_3_-etherate as the promoter, the proportion of 1,2-*trans*-product again decreased on increasing the donor concentration from 0.033 to 0.25 M at a fixed 1:1 donor: acceptor stoichiometry (Table 4, entries 4–6). However, as the donor concentration was increased further, the trend reversed and the proportion of 1,2-*trans*-product increased with further increases in concentration, indicative of a change in the predominant reaction mechanism.

## Discussion

**Scheme 5:**
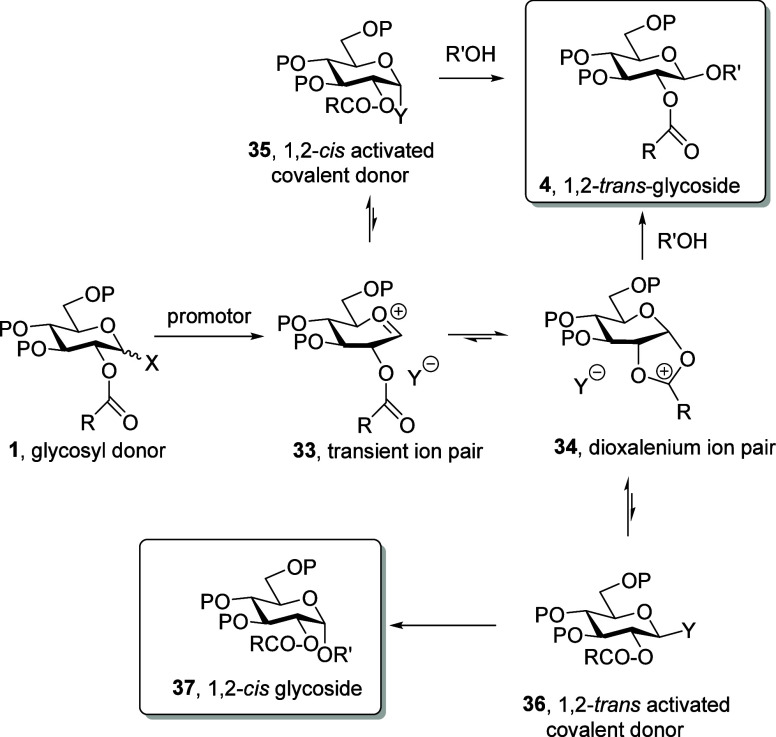
Revised Mechanism for Neighboring Group-Directed Glycosylation Accounting for Concentration Dependence, Illustrated for the Formation of a 1,2-*trans*-equatorial Glycoside Orthoester donors and products are omitted for clarity.

The examples presented demonstrate the influence of concentration and stoichiometry on the stereochemical outcome of a set of neighboring-group-assisted glycosylation reactions. In the examples studied, with one exception, lower concentrations and stoichiometries generally favor to a greater extent the 1,2-*trans*-glycosides viewed as arising from neighboring group participation. Higher concentrations and acceptor:donor stoichiometries on the other hand typically lead to erosion of selectivity. These observations are best explained by a revised general mechanism for neighboring group participation (Scheme 5) that critically incorporates the “counterion” Y^–^ formed during the activation process. This “counterion” will frequently be the triflate or other anion arising from the activation process but can in principle be any nucleophilic species present in the reaction mixture, including a nucleophilic catalyst.^109^ In this mechanism, the glycosyl donor **1** is activated by the promoter to the transient ion pair **33**, which may be either a contact ion pair (CIP) or a solvent-separated ion pair (SSIP), dependent on the particular system and the conditions. This ion pair is in equilibrium with fused dioxalenium ion pair **34**, which again may be either a CIP or an SSIP, and with activated covalent donor **35**. The fused dioxalenium ion pair **34** may undergo ring opening to give an isomeric activated covalent donor **36**. This mechanism does not preclude other entries into the dioxalenium ion, such as from an orthoester donor or the reversible kinetic formation of orthoester products, both as illustrated in Scheme 1, but they are not included here for the sake of clarity. Overall, therefore, the reaction mixture contains three activated donors, the dioxalenium ion pair **34**, and the two isomeric covalent donors **35** and **36**, each of which will proceed to one or more of the products on reaction with the acceptor alcohol, each with its own rate law and hence concentration and temperature dependence. We acknowledge that the product-forming reaction of the acceptor alcohol with any of the activated species **34**–**36** may proceed in a concerted manner or via kinetically equivalent pathways involving reversible formation of CIPs, but we do not distinguish between them here for the sake of clarity. The nonlinear nature of the concentration dependence observed in the examples studied presumably arises from the multiplicity of product forming pathways and their differing concentration dependences. Alternatively, as the influence of concentration tends to plateau, it is possible that at least some of the bimolecular reactions for formation of the 1,2-*cis*-glycosides from the highly reactive 1,2-*trans*-equatorial activated donor **36** are approaching the diffusion-controlled limit.^62,110,111^

**Scheme 6:**
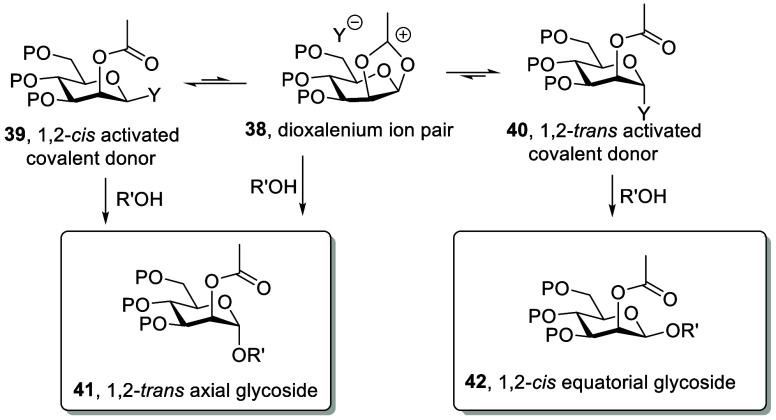
Abbreviated Mechanism for 1,2-*trans*-Axial Glycoside (β-D-Mannopyranoside) Formation with Neighboring Group Participation

It is important to note a subtle difference between the mechanism illustrated for neighboring group-directed 1,2-*trans*-equatorial glycoside formation (Scheme 5) and the analogous mechanism for 1,2-trans-*axial* glycoside formation. Thus, for the formation of the 1,2-*trans*-equatorial glycosides, two of the three activated intermediates, the dioxalenium ion **34** and the axial activated donor **35**, give the equatorial glycoside, while only one, the typically less stable but more reactive equatorial covalent donor **36** gives the axial glycoside. As shown in an abbreviated mechanism (Scheme 6), the opposite is true for the 1,2-*trans*-axial glycoside formation. Thus, two (**38** and **39**) of the three activated species still give the assumed product of neighboring group participation – the 1,2-*trans* axial glycoside **41** – and only one (**40**) gives the 1,2-*cis*-equatorial glycoside **42**, but it is the typically less-populated but more reactive of the two covalent donors **39** that pairs with the dioxalenium ion pair **38** in affording the 1,2-*trans*-glycoside **41**. It is to be expected, therefore, that neighboring group-directed 1,2-*trans*-axial and 1,2-*trans*-equatorial glycoside forming reactions show different dependences on concentration.

Two of the reactions studied, those of the per-*O*-benzoyl and per-*O*-acetyl-l-fucopyranosyl bromides **20** and **24** with the acceptor **18**, displayed essentially perfect 1,2-*trans*-selectivity (System 1) while the enantiomeric donors **19** and **23** showed strong concentration dependence in their coupling reactions with the same acceptor alcohol, consistent with stereochemical matching of the acceptor **18** with the l-donors and mismatching with the d-donors as reported originally by Spijker and van Boeckel.^63^ We suggest that for the case of the matched reaction pairs leading to very high 1,2-*trans*-selectivity the use of even higher concentrations and acceptor:donor stoichiometries would eventually lead to erosion of selectivity consistent with the overall picture. In one case study (System 3), two different promoters were employed, giving rise to concentration-dependent selectivity, albeit in different regions of the selectivity spectrum. This observation is consistent with a shift in the dioxalenium ion pair **34**:covalent donor **35** and **36** equilibria (Scheme 5) with the change in counterion, which in turn arises from the change in the relative stabilities of the ion pairs and the covalent donors. The use of BF_3_-etherate as a promoter for the coupling of donor **30** to acceptor **31** with formation of glycosides **32** (System 3, Table 4) is the one exception to the overall pattern of decreasing 1,2-*trans* selectivity with increasing concentration. Thus, in this series of experiments (Table 4, entries 4–9), the proportion of 1,2-*trans* product initially decreases as the donor concentration is increased from 0.033 to 0.25 M at a fixed 1:1 donor:acceptor stoichiometry but then increases again as the concentration is further increased. Such a discontinuity is consistent with a reaction operating close to the borderline between two or more mechanisms with different molecularities.

The overall message is clear: as with simple glycosylation reactions^96,112^ and perhaps with the still controversial distal-group directed glycosylation,^17,113−115^ the outcome of neighboring group-directed glycosylation reactions is concentration-dependent, albeit many such reactions operate in the regime of high 1,2-*trans*-selectivity. When less than optimal selectivity is observed, in addition to the more traditional approaches of varying the promoter and the participating ester, an increase in the desired 1,2-*trans*-selectivity can likely be engineered by reducing the overall reaction concentration and so limiting the competing bimolecular reaction pathways. As different acceptor alcohols are known to have different nucleophilicities toward a range of glycosyl donors,^105,110,116^ it follows that the observation of high neighboring group-directed 1,2-*trans* selectivity with a given donor:acceptor pair does not necessarily extrapolate to the use of a different acceptor with the same donor under otherwise identical conditions, with the use of more nucleophilic acceptors more likely to result in erosion of selectivity. Viewed in the broader context of glycosylation as a whole, neighboring group-directed reactions align with direct displacements in that their stereochemical outcome depends on multiple factors including protecting groups, promoters, counterions, whether catalytic or stoichiometric or leaving group-derived, acceptor nucleophilicity, solvent, temperature, and concentration.^17,52,53,117^ This level of complexity is inconsistent with the classical naked oxocarbenium ion centric view^118,119^ of glycosylation reactions and does not auger well for the development of broadly applicable general glycosylation conditions, whether in the solution-phase or on a solid support. Optimal solutions for individual glycosylations are likely to be mechanism-based and associative with transition states combining donor, acceptor, and promotor(s) as has been demonstrated in a number of elegant studies.^46,47,120−123^

Finally, the clear dependence of selectivity on concentration observed in the examples presented underlines the importance of thorough reporting of stoichiometry, temperature, and concentration for neighboring group-directed glycosylation reactions as we have already emphasized for glycosylations in the absence of ester protecting groups at the 2-position of the donor.^96^

## Conclusions

Evidence is presented that the stereochemical outcome of glycosylation reactions employing neighboring group participation to achieve 1,2-*trans*-selectivity, either axial or equatorial, can be concentration-dependent with lower concentrations favoring the desired 1,2-*trans*-product and higher concentrations likely to result in erosion of selectivity. These results are best interpreted in terms of a modern picture of neighboring group participation arising from competing reaction mechanisms for the formation of the glycoside (Scheme 5). In this picture, the nucleophilic acceptor alcohol combines either with the bridged dioxalenium ion pair **34** or the covalent donors **35** and **36** with which it is in equilibrium, with the two pathways having different concentration dependence. Careful reporting of stoichiometry, temperature, and concentration is critical to the reproducibility of all glycosylation reactions.

## Data Availability

The data underlying this study are available in the published article and its Supporting Information.

## Supporting Information Available

Full experimental details and copies of NMR spectra (PDF)
