# Supplementary material for: The Stereoselectivity of Neighboring Group-Directed Glycosylation Is Concentration-Dependent
Source: J Am Chem Soc. 2025 Feb 5;147(7):5808–18. doi: 10.1021/jacs.4c14402 (PMC11848824; doi:10.1021/jacs.4c14402)

Supporting Information

The Stereoselectivity of Neighboring Group-Directed Glycosylation is Concentration Dependent

Pallabita Basu[a,b] and David Crich[a,b,c,*]

a) Department of Pharmaceutical and Biomedical Sciences, University of Georgia, 250 West Green Street, Athens, GA 30602, USA

b) Complex Carbohydrate Research Center, University of Georgia, 315 Riverbend Road, Athens, GA 30602, USA

c) Department of Chemistry, University of Georgia, 302 East Campus Road, Athens, GA 30602, USA

Email: David.Crich@uga.edu

Table of contents

General experimental	S-3
Experimental procedure and characterization data	S-3-S-17
References	S-18
Copies of ^{1}H, ^{13}C, 2D and crude NMR Spectra	S-19-S152

General experimental

All reagents were purchased from commercial sources and used without further purification unless noted. All reactions were carried out under a positive pressure of argon or nitrogen. Solvents used for column chromatography were analytical grade and were purchased from commercial suppliers. Thin-layer chromatography was carried out with 250 µm glass backed silica (XHL) plates. Detection of compounds was achieved by UV absorption (254 nm) and by charring with a ceric ammonium molybdate solution. All organic solutions were concentrated at 30-45 ºC on a rotary evaporator. Purification of crude residues was performed by flash column chromatography using a COMBIFLASH® NextGen system, unless otherwise stated. Specific rotations were recorded on an automatic polarimeter in $CHCl_3$, at 589 nm and 23 ± 2 ºC with a path length of 10 cm. Nuclear magnetic resonance (NMR) spectra of all compounds were obtained in $CDCl_3$ (δ 7.27 and 77.1 ppm, respectively), C_6D_6 (δ 7.16 and 128.1 ppm, respectively) at 600 or 900 MHz as indicated. The chemical shifts (δ) are calculated with respect to residual solvent peak and are given in ppm. Multiplicities are abbreviated as follows: s (singlet), d (doublet), t (triplet), q (quartet), dd (doublet of doublet), b s (broad singlet) and m (multiplet). Peak assignments were based on two-dimensional NMR (COSY and HSQC) experiments, and the configurational or conformational assignments were determined with the aid of HMBC NMR experiments. High-resolution electrospray ionization (ESI) mass spectrometry spectra were recorded using a Thermo Scientific Orbitrap mass analyzer.

Experimental procedures and characterization data

Ethyl 2-deoxy-2-phthalimido-4,6-*O*-isopropylidene-1-thio-β-D-glucopyranoside (18)

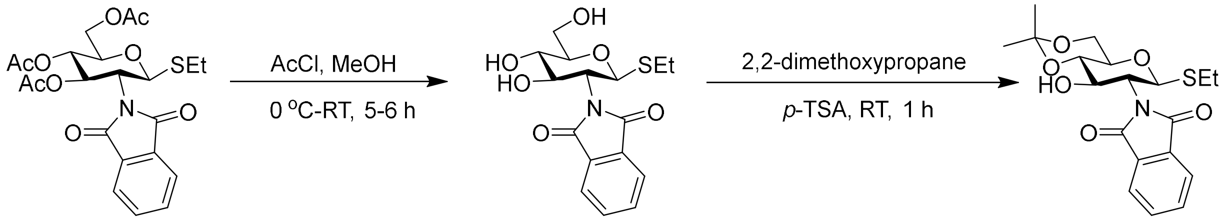

To a solution of ethyl 3,4,6-tri-*O*-acetyl-2-deoxy-2-phthalimido-1-thio-β-D-glucopyranoside[1] (7 gm, 14.6 mmol, 1 equiv) in dry methanol (100 mL) acetyl chloride (12.5 mL, 175.2 mmol,12 equiv) was added dropwise at 0 ºC under an inert atmosphere and the reaction mixture was stirred at room temperature. After the starting material disappeared (indicated by TLC, 5-6 h), the reaction was quenched with sat. aqueous $NaHCO_3$ (50 mL). The mixture was then extracted with EtOAc (3 X 100 mL), the combined organic phase was washed with brine, dried over Na_2SO_4, filtered, and evaporated under reduced pressure to give a crude residue. The crude residue was directly subjected to the next step without further purification.

The crude mixture was dissolved in 2,2-dimethoxypropane (75 mL), under an inert atmosphere followed by the addition of *p*-TSA (502 mg, 2.9 mmol, 0.2 equiv) at room temperature. The reaction mixture was stirred for 1 h at the same temperature before it was quenched with sat. aqueous $NaHCO_3$ solution (40 mL), the aqueous layer was extracted with EtOAc (3 x 50 mL). The combined organic phases were washed with brine, dried over Na_2SO_4, filtered, and evaporated under reduced pressure. The crude mixture was then subjected to flash chromatography over silica gel to obtain ethyl 2-deoxy-2-phthalimido-4,6-*O*-isopropylidene-1-thio-β-D-glucopyranoside **18** using hexane/ethyl acetate gradient (60-70%) in 72% yield (4.1 g) over two steps. $[\alpha]_D^{23}$ +1.7 (*c*=1.0, $CHCl_3$), R_f= ethyl acetate in hexane. White Solid. ^1H NMR (600 MHz, $CDCl_3$) δ 7.87-7.83 (m, 2H, Ar), 7.73-7.72 (m, 2H, Ar), 5.35 (d, *J* = 10.5 Hz, 1H, H1), 4.48 (ddd, *J* = 10.0, 8.7, 3.0 Hz, 1H, H3), 4.27 (t, *J* = 10.3 Hz, 1H, H2), 3.98 (dd, *J* = 10.9, 5.3 Hz, 1H, H5), 3.80 (t, *J* = 10.5 Hz, 1H, H6a), 3.63 (t, *J* = 9.2 Hz, 1H, H4), 3.51 (td, *J* = 9.9, 5.3 Hz, 1H, H6b), 2.72-2.60 (m, 2H, CH_2-SEt), 2.52 (Br S, 1H, OH), 1.51 (s, 3H, CH_3), 1.41 (s, 3H, CH_3), 1.17 (t, *J* = 7.5 Hz, 3H, CH_3-SEt). ^{13}C NMR (151 MHz, $CDCl_3$) δ 168.3, 167.9, 134.4, 131.9, 131.8, 124.0, 123.5, 100.0, 81.9 (C1), 75.1, 71.5, 70.2, 62.2, 55.8, 29.1, 24.3, 19.3, 15.0. ESI-HRMS: *m*/*z* calcd for $C_{19}H_{23}O_6NSNa$ $[M+Na]^+$ 416.1138, found 416.1130.

2,3,4-Tri-*O*-benzoyl-α-D-fucopyranosyl bromide (19) was prepared according to the literature procedure.[2a]

Glycosylation[3]

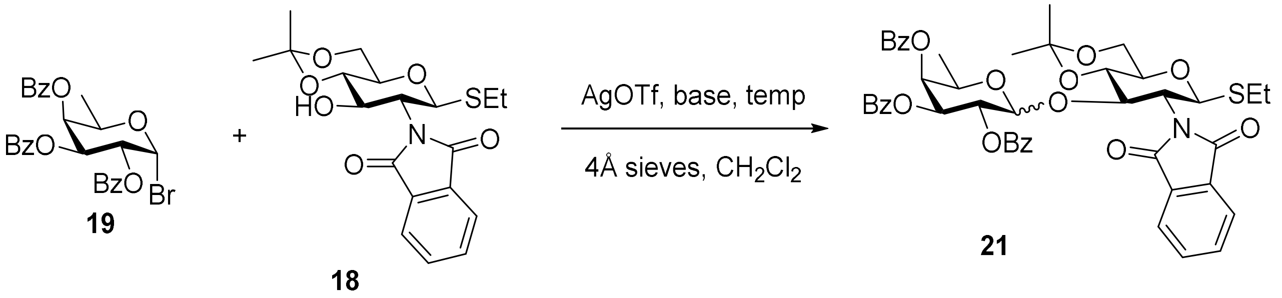

A mixture of donor 2,3,4-tri-*O*-benzoyl-α-D-fucopyranosyl bromide **19** and acceptor ethyl 2-deoxy-2-phthalimido-4,6-*O*-isopropylidene-1-thio-β-D-glucopyranoside **18**, in the specified ratios (see Table), was co-evaporated with toluene three times and then maintained under high vacuum in the presence of activated 4 Å molecular sieves (3 g/mmol) for 0.5 h. Anhydrous CH_2Cl_2 was added to reach the desired concentration (see Table), and the mixture was stirred for an additional 0.5 h at room temperature under an argon atmosphere. The reaction mixture was then cooled to -25 °C (bath temp.) and stirred continuously for 0.25 h. 2,4,6-Tri-*tert*-butylpyrimidine (TTBP, 1.5 equiv relative to the donor) followed by silver trifluoromethanesulfonate (AgOTf, 1.1 equiv relative to the donor) were added at the same temperature, and the reaction mixture was stirred at -25 °C for 6 h before it was quenched with triethylamine (12 equivalents, 0.25 mL). The reaction progress was monitored using TLC. After that, the reaction was diluted with dichloromethane (10 mL), filtered through P8

filter paper, and washed with saturated aqueous $NaHCO_3$ (10 mL X 2) and brine (10 mL). The organic layer was then separated, dried with Na_2SO_4, filtered, and concentrated under reduced pressure. The resulting products were purified by flash column chromatography on silica gel using a hexane/ethyl acetate gradient (30-40%), producing glycoside **21** as a white solid as an α/β-mixture. The anomeric ratio of the products was assessed from the crude reaction mixtures by analyzing the anomeric signals in the ^{1}H NMR spectra.

Entry	Donor (mmol)	Acceptor (mmol)	Donor Conc (M)	Donor:Acceptor Stoichiometry	CH_2Cl_2 (mL)	Temp. (°C)	Yield (%) **21**	1,2-*cis*:1,2-*trans* Ratio
1	0.15	0.15	0.033	1:1	4.5	-25	76	1:6.4
2	0.15	0.15	0.066	1:1	2.3	-25	72	1:3.7
3	0.15	0.15	0.1	1:1	1.5	-25	68	1:3.0
4	0.15	0.15	0.2	1:1	0.75	-25	74	1:2.9
5	0.15	0.15	0.3	1:1	0.5	-25	71	1:3.0

Ethyl 2-deoxy-2-phthalimido-4,6-*O*-isopropylidene-3-*O*-(2,3,4-tri-*O*-benzoyl-α-D-fucopyranosyl)-1-thio-β-D-glucopyranoside (21α)

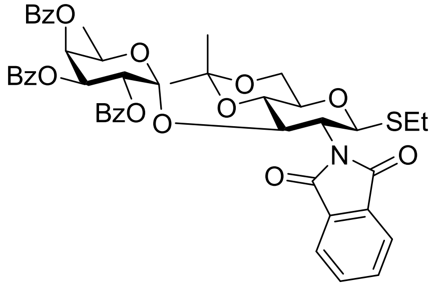

[α]$_D^{23}$ +254.6 (*c*=1.0, $CHCl_3$), R_f= 0.33 in 30% ethyl acetate in hexane. White Solid. ^{1}H NMR (600 MHz, $CDCl_3$, *α*-isomer) δ 8.04-8.01 (m, 3H, Ar), 7.99 (d, *J* = 7.4 Hz, 2H, Ar), 7.90 (d, *J* = 7.3 Hz, 1H, Ar), 7.83-7.76 (m, 2H, Ar), 7.72 (d, *J* = 7.6 Hz, 2H, Ar), 7.56 (t, *J* = 7.4 Hz, 1H, Ar), 7.52 (t, *J* = 7.4 Hz, 1H, Ar), 7.44-7.37 (m, 5H, Ar), 7.19 (t, *J* = 7.7 Hz, 2H, Ar), 5.79 (d, *J* = 4.0 Hz, 1H, H1), 5.68 (dd, *J* = 11.0, 3.4 Hz, 1H, H3), 5.55 (dd, *J* = 11.0, 4.0 Hz, 1H, H2), 5.37 (d, *J* = 10.6 Hz, 1H, H1′), 5.35 (d, *J* = 3.0 Hz, 1H, H4), 4.75 (apparent t, *J* = 9.4 Hz, 1H, H3′), 4.45 (t, *J* = 10.3 Hz, 1H, H2′), 3.89 (dd, *J* = 10.8, 5.4 Hz, 1H, H6a′), 3.74-3.64 (m, 3H, H4′, H6b′, H5), 3.52 (td, *J* = 10.0, 5.4 Hz, 1H, H5′), 2.73-2.62 (m, 2H, CH_2′-SEt), 1.26 (s, 3H, CH_3′), 1.18 (t, *J* = 7.4 Hz, 3H, CH_3′-SEt), 0.82 (s, 3H, CH_3′), 0.59 (d, *J* = 6.5 Hz, 3H, CH_3). ^{13}C NMR (151 MHz, $CDCl_3$) δ 168.1, 167.4, 166.0, 165.9, 165.5, 134.9, 134.7, 133.6, 133.5, 133.1, 131.9, 131.4, 130.1, 130.0, 129.7, 129.5, 129.5, 129.4, 128.7(x2), 128.3, 124.5, 123.6, 99.6, 97.1 (C1), 82.0 (C1′), 75.7, 74.4, 71.7, 71.2, 68.7, 68.2, 65.3, 61.8, 54.4, 28.8, 24.4, 18.1, 15.7, 15.0. ESI-HRMS: *m*/*z* calcd for $C_{46}H_{45}O_{13}NSNa$ $[M+Na]^+$ 874.2503, found 874.2487.

Ethyl 2-deoxy-2-phthalimido-4,6-*O*-isopropylidene-3-*O*-(2,3,4-tri-*O*-benzoyl-β-D-fucopyranosyl)-1-thio-β-D-glucopyranoside (21β)

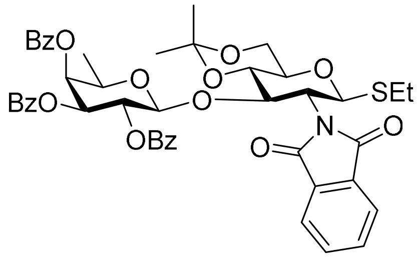

[α]$_D^{23}$ +192.2 (*c*=1.0, $CHCl_3$), R_f= 0.24 in 30% ethyl acetate in hexane. White Solid. ^{1}H NMR (600 MHz, $CDCl_3$, β-isomer) δ 8.06 (d, *J* = 7.2 Hz, 2H), 7.74 (d, *J* = 7.5 Hz, 1H, Ar), 7.62 (d, *J* = 7.3 Hz, 2H, Ar), 7.57 (dt, *J* = 15.3, 7.6 Hz, 2H, Ar), 7.46 (t, *J* = 7.8 Hz, 2H, Ar), 7.43-7.41 (m, 3H, Ar), 7.33 (t, *J* = 7.5 Hz, 1H, Ar), 7.29 (t, *J* = 7.5 Hz, 1H, Ar), 7.20 (t, *J* = 7.6 Hz, 2H, Ar), 7.13 (t, *J* = 7.7 Hz, 2H, Ar), 6.83 (d, *J* = 7.4 Hz, 1H, Ar), 5.62 (d, *J* = 3.5 Hz, 1H, H4), 5.49 (dd, *J* = 10.3, 8.0 Hz, 1H, H2), 5.32 (dd, *J* = 10.3, 3.5 Hz, 1H, H3), 5.26 (d, *J* = 10.6 Hz, 1H, H1′), 5.03 (d, *J* = 8.1 Hz, 1H, H1), 4.69 (apparent t, *J* = 9.2 Hz, 1H, H3′), 4.43 (t, *J* = 10.3 Hz, 1H, H2′), 4.03-3.96 (m, 3H, H4′, H5, H6a′), 3.86 (t, *J* = 10.6 Hz, 1H, H6b′), 3.56 (td, *J* = 10.0, 5.3 Hz, 1H, H5′), 2.67-2.54 (m, 2H, CH_2′-SEt), 1.61 (s, 3H, CH_3′), 1.50 (s, 3H, CH_3′), 1.33 (d, *J* = 6.4 Hz, 3H, CH_3), 1.11 (t, *J* = 7.4 Hz, 3H, CH_3′-SEt). ^{13}C NMR (151 MHz, $CDCl_3$) δ 168.1, 167.1, 166.2, 165.6, 164.8, 133.8, 133.7, 133.5, 133.2, 133.0, 131.3, 131.1, 130.2, 129.7 (x2), 129.2, 128.9, 128.7, 128.3, 128.2, 123.6, 122.9, 100.7, 99.7(C1), 81.6 (C1′), 77.8, 74.8, 72.8, 71.4, 71.0, 70.7, 69.8, 62.2, 54.1, 29.5, 23.9, 19.3, 16.8, 14.9. ESI-HRMS: *m*/*z* calcd for $C_{46}H_{45}O_{13}NSNa$ $[M+Na]^+$ 874.2503, found 874.2487.

2,3,4-Tri-*O*-benzoyl-α-L-fucopyranosyl bromide (20) was prepared according to literature procedure.[2a]

Glycosylation[3]

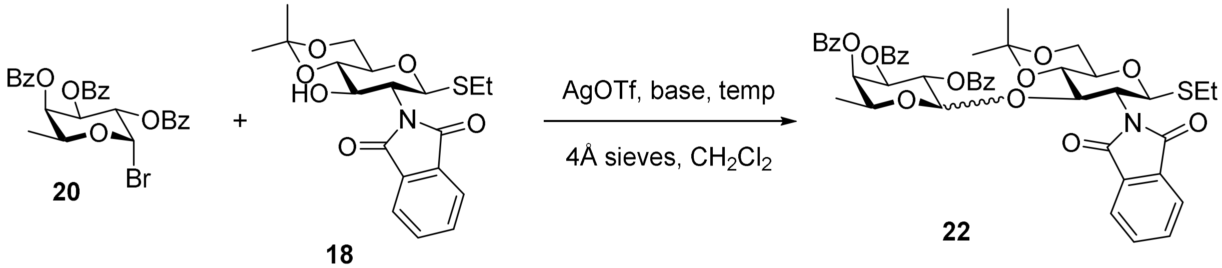

A mixture of donor 2,3,4-tri-*O*-benzoyl-α-L-fucopyranosyl bromide **20** and acceptor ethyl 2-deoxy-2-phthalimido-4,6-*O*-isopropylidene-1-thio-β-D-glucopyranoside **18**, in the specified ratios (see Table), was co-evaporated with toluene three times and then maintained under high vacuum in the presence of activated 4 Å molecular sieves (3 g/mmol) for 0.5 h. Anhydrous CH_2Cl_2 was added to reach the desired concentration (see Table), and the mixture was stirred for an additional 0.5 h at room temperature under an argon atmosphere. The reaction mixture was cooled to -25 °C/-45 °C (bath temp.) and stirred continuously for 0.25 h. 2,4,6-Tri-*tert*-butylpyrimidine (TTBP, 1.5 equiv relative to the donor) followed by silver trifluoromethanesulfonate (AgOTf, 1.1 equiv relative to the donor) were added at the same temperature, and the reaction mixture was stirred at -25

°C/-45 °C for 6 h before being quenched with triethylamine (12 equiv, 0.25 mL). The reaction progress was monitored using TLC. After that, the reaction was diluted with dichloromethane (10 mL), filtered through P8 filter paper, and washed with saturated aqueous $NaHCO_3$ (10 mL x 2) and brine (10 mL). The organic layer was then separated, dried with Na_2SO_4, filtered, and concentrated under reduced pressure. The resulting products were purified by flash column chromatography on silica gel using a hexane/ethyl acetate gradient (30-40%), producing pure β-glycoside **22** as a white solid. The anomeric ratio of the products was assessed from the crude reaction mixtures by analyzing the anomeric signals in the ^{1}H NMR spectra.

Entry	Donor (mmol)	Acceptor (mmol)	Donor Conc (M)	Donor:Acceptor Stoichiometry	CH_2Cl_2 (mL)	Temp. (°C)	Yield (%) **22**	1,2-*cis*:1,2-*trans* Ratio
1	0.15	0.15	0.033	1:1	4.5	-25	89	>95% 1,2-*trans*
2	0.15	0.15	0.2	1:1	0.75	-25	83	>95% 1,2-*trans*
3	0.15	0.15	0.033	1:1	4.5	-45	76	>95% 1,2-*trans*
4	0.15	0.15	0.2	1:1	0.75	-45	73	>95% 1,2-*trans*

Ethyl 2-deoxy-2-phthalimido-4,6-*O*-isopropylidene-3-*O*-(2,3,4-tri-*O*-benzoyl-β-L-fucopyranosyl)-1-thio-β-D-glucopyranoside (22β)

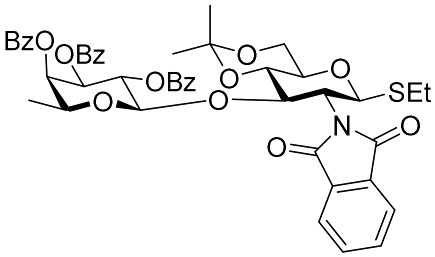

[α]$_D$^{23} +16.1 (*c*=1.0, $CHCl_3$), R_f= 0.28 in 30% ethyl acetate in hexane. White Solid. ^{1}H NMR (600 MHz, $CDCl_3$, β-isomer) δ 7.98 (d, *J* = 6.9 Hz, 2H, Ar), 7.82 (d, *J* = 6.8 Hz, 2H, Ar), 7.75 (d, *J* = 7.4 Hz, 1H, Ar), 7.72 (d, *J* = 7.0 Hz, 2H, Ar), 7.66 (t, *J* = 7.4 Hz, 1H, Ar), 7.60 (d, *J* = 7.5 Hz, 1H, Ar), 7.56-7.49 (m, 4H, Ar), 7.40-7.35 (m, 4H, Ar), 7.21 (t, *J* = 7.7 Hz, 2H, Ar), 5.50-5.46 (m, 1H, H2), 5.45 (d, *J* = 10.7 Hz, 1H, H1′), 5.41-5.38 (m, 2H, H3, H4), 4.82 (d, *J* = 7.7 Hz, 1H, H1), 4.51 (t, *J* = 9.4 Hz, 1H, H3′), 4.35 (t, *J* = 10.2 Hz, 1H, H2′), 3.91 (dd, *J* = 10.9, 5.3 Hz, 1H, H6a′), 3.80 (q, *J* = 6.4 Hz, 1H, H5), 3.67 (t, *J* = 10.6 Hz, 1H, H6b′), 3.62 (t, *J* = 9.4 Hz, 1H, H4′), 3.48 (td, *J* = 10.0, 5.3 Hz, 1H, H5′), 2.73-2.61 (m, 2H, CH_2′-SEt), 1.27 (s, 3H, CH_3′), 1.18 (t, *J* = 7.4 Hz, 3H, CH_3′-SEt), 0.82 (s, 3H, CH_3′), 0.51 (d, *J* = 6.4 Hz, 3H, CH_3). ^{13}C NMR (151 MHz, $CDCl_3$) δ 168.7, 167.4, 165.8, 165.7, 164.8, 133.8, 133.7, 133.6, 133.4, 133.3, 132.6, 132.0, 130.1, 130.0, 129.9, 129.8, 129.3, 129.0, 128.7, 128.5, 128.4, 123.5, 123.4, 101.4 (C1), 99.6, 81.7 (C1′), 77.0, 76.7, 74.3, 72.3, 71.5, 70.8, 70.1, 69.0, 62.1, 54.6, 29.0, 24.0, 18.1, 15.9, 15.0. ESI-HRMS: *m/z* calcd for $C_{46}H_{45}O_{13}NSNa$ [M+Na]$^+$ 874.2503, found 874.2476.

2,3,4-Tri-*O*-acetyl-α-D-fucopyranosyl bromide (23) was prepared according to the procedure.[2b]

Glycosylation

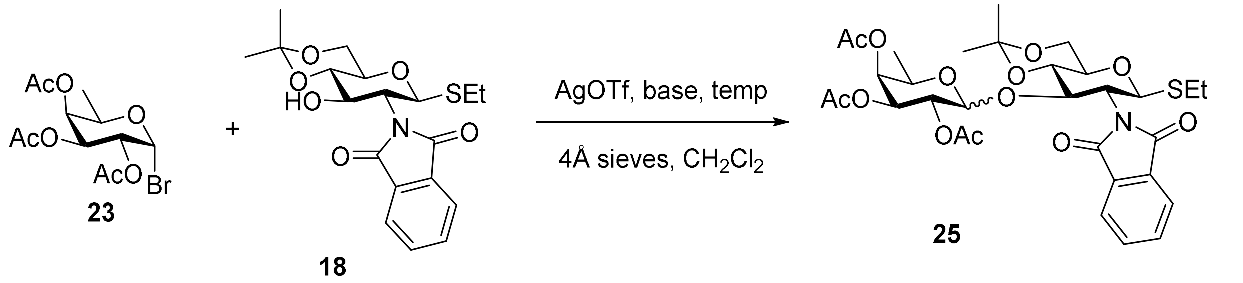

A mixture of 2,3,4-tri-*O*-acetyl-α-D-fucopyranosyl bromide **23** and ethyl ethyl 2-deoxy-2-phthalimido-4,6-*O*-isopropylidene-1-thio-β-D-glucopyranoside **18**, in the specified ratios (see Table), was co-evaporated with toluene three times and then maintained under high vacuum in the presence of activated 4 Å molecular sieves (3 g/mmol) for 0.5 h. Anhydrous CH_2Cl_2 was added to reach the desired concentration (see Table), and the mixture was stirred for an additional 0.5 h at room temperature under an argon atmosphere. The reaction mixture was cooled to -25 °C (bath temp.) and stirred continuously for 0.25 h. 2,4,6-Tri-*tert*-butylpyrimidine (TTBP, 1.5 equiv relative to the donor) followed by silver trifluoromethanesulfonate (AgOTf, 1.1 equiv relative to the donor) were introduced at the same temperature, and the reaction mixture was stirred at -25 °C for 6 h before being quenched with triethylamine (12 equiv, 0.25 mL). The reaction progress was monitored using TLC. After that, the reaction was diluted with dichloromethane (10 mL), filtered through P8 filter paper, and washed with saturated aqueous $NaHCO_3$ (10 mL x 2) and brine (10 mL). The organic layer was then separated, dried with Na_2SO_4, filtered, and concentrated under reduced pressure. The resulting products were purified by flash column chromatography on silica gel using a hexane/ethyl acetate gradient (40-60%), producing glycoside **25** as a white solid as an α/β-mixture. The anomeric ratio of the products was assessed from the crude reaction mixtures by analyzing the anomeric signals in the ^{1}H NMR spectra.

Entry	Donor (mmol)	Acceptor (mmol)	Donor Conc (M)	Donor:Acceptor Stoichiometry	CH_2Cl_2 (mL)	Temp. (°C)	Yield (%) **25**	1,2-*cis*:1,2-*trans* Ratio
1	0.15	0.15	0.033	1:1	4.5	-25	79	>95% 1,2-*trans*
2	0.15	0.15	0.066	1:1	2.3	-25	77	1:6.9
3	0.15	0.15	0.1	1:1	1.5	-25	60	1:3.2
4	0.15	0.15	0.2	1:1	0.75	-25	63	1:2.7
5	0.15	0.15	0.3	1:1	0.5	-25	57	1:2.4

Ethyl 2-deoxy-2-phthalimido-4,6-*O*-isopropylidene-3-*O*-(2,3,4-tri-*O*-acetyl-α-D-fucopyranosyl)-1-thio-β-D-glucopyranoside (25*α*)

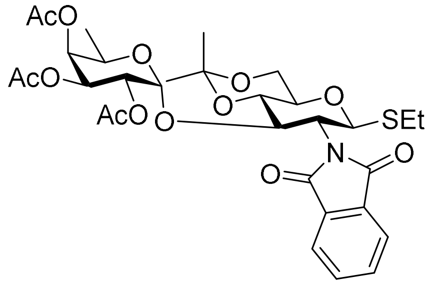

[α]D[23] +51.3 (c=1.0, $CHCl_3$), R_f = 0.54 in 50% ethyl acetate in hexane. White Solid. ^{1}H NMR (600 MHz, $CDCl_3$) δ 7.90-7.85 (m, 2H, Ar), 7.76-7.74 (m, 2H, Ar), 5.61 (d, J = 4.8 Hz, 1H, H1), 5.34 (d, J = 10.7 Hz, 1H, H1′), 5.04 (dd, J = 3.4, 1.8 Hz, 1H, H4), 4.63 (dd, J = 7.1, 3.4 Hz, 1H, H3), 4.49 (t, J = 9.2 Hz, 1H, H3′), 4.24 (dd, J = 10.7, 9.6 Hz, 1H, H2′), 4.05 (dd, J = 6.5, 1.8 Hz, 1H, H5), 3.98 (dd, J = 11.0, 5.4 Hz, 1H, H6a′), 3.79 (t, J = 10.7 Hz, 1H, H6b′), 3.68 (t, J = 9.3 Hz, 1H, H4′), 3.52 (td, J = 10.0, 5.2 Hz, 1H, H5′), 3.46 (dd, J = 7.1, 4.8 Hz, 1H, H2), 2.71-2.59 (m, 2H, CH_2′-SEt), 2.03 (s, 3H, CH_3), 1.89 (s, 3H, CH_3), 1.50 (s, 3H, CH_3), 1.46 (s, 3H, CH_3), 1.41 (s, 3H, CH_3), 1.16 (t, J = 7.5 Hz, 3H, CH_3), 1.07 (d, J = 6.5 Hz, 3H, CH_3). ^{13}C NMR (151 MHz, $CDCl_3$) δ 170.2, 170.0, 168.5, 167.5, 134.3, 134.2, 132.2, 131.8, 123.7, 123.5, 120.6, 99.8, 98.1 (C1), 81.8 (C1’), 73.7, 72.5, 72.0, 71.9, 71.8, 68.7, 67.3, 62.3, 55.2, 29.1, 24.4, 24.2, 20.9, 20.7, 19.2, 16.1, 15.0. ESI-HRMS: m/z calcd for $C_{31}H_{39}O_{13}NSNa$ $[M+Na]^+$ 688.2034, found 688.2019.

Ethyl 2-deoxy-2-phthalimido-4,6-*O*-isopropylidene-3-*O*-(2,3,4-tri-*O*-acetyl-β-D-fucopyranosyl)-1-thio-β-D-glucopyranoside (25β)

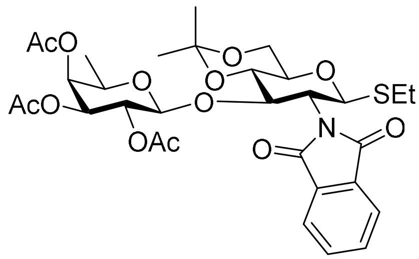

[α]D[23] +20.5 (c=1.0, $CHCl_3$), R_f = 0.48 in 30% ethyl acetate in hexane. White Solid. ^{1}H NMR (900 MHz, $CDCl_3$, β-isomer) δ 7.90 (d, J = 7.3 Hz, 1H, Ar), 7.80 (d, J = 7.2 Hz, 1H, Ar), 7.76-7.73 (m, 2H, Ar), 5.23 (d, J = 10.7 Hz, 1H, H1′), 5.09 (dd, J = 3.5, 1.1 Hz, 1H, H4), 4.88 (dd, J = 10.2, 7.8 Hz, 1H, H2), 4.82 (dd, J = 10.3, 3.5 Hz, 1H, H3), 4.67 (d, J = 7.9 Hz, 1H, H1), 4.55 (dd, J = 9.9, 8,6 Hz, 1H, H3′), 4.35 (t, J = 10.3 Hz, 1H, H2′), 3.97 (dd, J = 11.1, 5.3 Hz, 1H, H6a′), 3.87 (t, J = 9.2 Hz, 1H, H4′), 3.81 (t, J = 10.7 Hz, 1H, H6b′), 3.66 (q, J = 6.3 Hz, 1H, H5), 3.50 (td, J = 10.0, 5.3 Hz, 1H, H5′), 2.62 (ddq, J = 53.4, 12.4, 7.5 Hz, 2H, CH_2′-SEt), 2.11 (s, 3H, CH_3), 1.83 (s, 3H, CH_3), 1.54 (s, 3H, CH_3), 1.54 (s, 3H, CH_3), 1.40 (s, 3H, CH_3), 1.19 (d, J = 6.4 Hz, 3H, CH_3), 1.13 (t, J = 7.5 Hz, 3H, CH_3). ^{13}C NMR (226 MHz, $CDCl_3$) δ 170.9, 170.2, 169.1, 168.5, 167.0, 134.5, 134.0, 132.0, 131.9, 124.1, 123.1, 100.3 (C1), 99.5, 81.7 (C1′), 74.7, 71.7, 71.4, 70.2, 70.0, 69.1, 62.1, 54.2, 29.4, 23.9, 20.8, 20.6, 20.4, 19.2, 16.5, 14.9. ESI-HRMS: m/z calcd for $C_{31}H_{39}O_{13}NSNa$ $[M+Na]^+$ 688.2034, found 688.2026.

2,3,4-Tri-*O*-acetyl-α-L-fucopyranosyl bromide (24) was prepared according to literature procedure.[2b]

Glycosylation

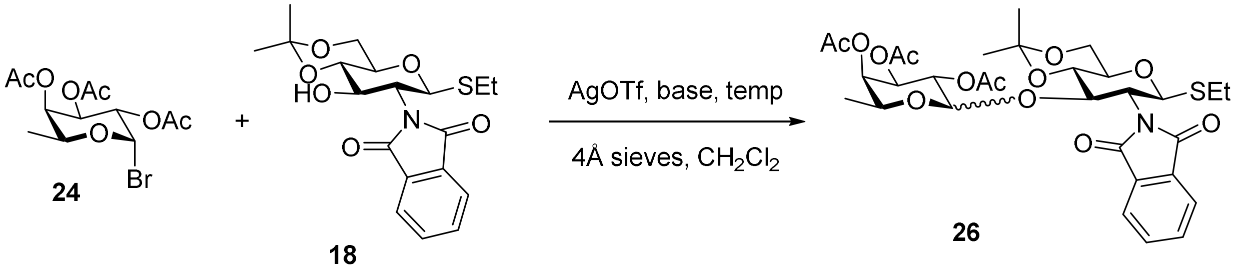

A mixture of donor 2,3,4-tri-*O*-acetyl-α-L-fucopyranosyl bromide **24** and acceptor ethyl 2-deoxy-2-phthalimido-4,6-*O*-isopropylidene-thio-β-D-glucopyranoside **18**, in the specified ratios (see Table), was co-evaporated with toluene three times and then maintained under high vacuum in the presence of activated 4 Å molecular sieves (3 g/mmol) for 0.5 h. Anhydrous CH_2Cl_2 was added to reach the desired concentration (see Table), and the mixture was stirred for an additional 0.5 h at room temperature under an argon atmosphere. The reaction mixture was cooled to -25 °C/-45 °C (bath temp.) and stirred continuously for 0.25 h. 2,4,6-Tri-*tert*-butylpyrimidine (TTBP, 1.5 equiv relative to the donor) followed by silver trifluoromethanesulfonate (AgOTf, 1.1 equiv relative to the donor) were introduced at the same temperature, and the reaction mixture was stirred at -25 °C/-45 °C for 6 h before being quenched with triethylamine (12 equivalents, 0.25 mL). The reaction progress was monitored using TLC. After that, the reaction was diluted with dichloromethane (10 mL), filtered through P8 filter paper, and washed with saturated aqueous $NaHCO_3$ (10 mL X 2) and brine (10 mL). The organic layer was then separated, dried with Na_2SO_4, filtered, and concentrated under reduced pressure. The resulting products were purified by flash column chromatography on silica gel using a hexane/ethyl acetate gradient (40-60%), producing pure β-glycoside **26** as a white solid. The anomeric ratio of the products was assessed from the crude reaction mixtures by analyzing the anomeric signals in the ^{1}H NMR spectra.

Entry	Donor (mmol)	Acceptor (mmol)	Donor Conc (M)	Donor:Acceptor Stoichiometry	CH_2Cl_2 (mL)	Temp. (°C)	Yield (%) **26**	1,2-*cis*:1,2-*trans* Ratio
1	0.15	0.15	0.033	1:1	4.5	-25	89	>95% 1,2-*trans*
2	0.15	0.15	0.2	1:1	0.75	-25	83	>95% 1,2-*trans*
3	0.15	0.15	0.033	1:1	4.5	-45	76	>95% 1,2-*trans*
4	0.15	0.15	0.2	1:1	0.75	-45	73	>95% 1,2-*trans*

Ethyl 2-deoxy-2-phthalimido-4,6-*O*-isopropylidene-3-*O*-(2,3,4-tri-*O*-acetyl-β-L-fucopyranosyl)-1-thio-β-D-glucopyranoside (26β)

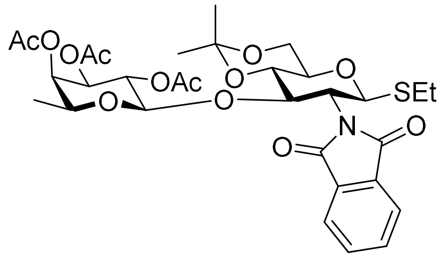

[α]$_D^{23}$ +37.2 (*c*=1.0, $CHCl_3$), R_f= 0.48 in 50% ethyl acetate in hexane. White Solid. ^{1}H NMR (600 MHz, $CDCl_3$, β-isomer) δ 7.91-7.85 (m, 1H, Ar), 7.79-7.77 (m, 1H, Ar), 7.72-7.67 (m, 2H, Ar), 5.40 (d, *J* = 10.7 Hz, 1H, H1′), 4.90 (d, *J* = 2.0 Hz, 1H, H4), 4.87-4.80 (m, 2H, H2, H3), 4.50 (d, *J* = 7.8 Hz, 1H, H1), 4.46 (dd, *J* = 9.9, 9.0 Hz, 1H, H3′), 4.27 (dd, *J* = 10.7, 9.9 Hz, 1H, H2′), 3.98 (dd, *J* = 10.9, 5.3 Hz, 1H, H6a′), 3.77 (t, *J* = 10.6 Hz, 1H, H6b′), 3.71 (t, *J* = 10.6 Hz, 1H, H4′), 3.54-3.46 (m, 2H, H5, H5′), 2.71-2.60 (m, 2H, CH_2′-SEt), 1.99 (s, 3H, CH_3), 1.88 (s, 3H, CH_3), 1.81 (s, 3H, CH_3), 1.46 (s, 3H, CH_3), 1.41 (s, 3H, CH_3), 1.17 (t, *J* = 7.4 Hz, 3H, CH_3′-SEt), 0.50 (d, *J* = 6.4 Hz, 3H, CH_3). ^{13}C NMR (151 MHz, $CDCl_3$) δ 170.4, 170.3, 169.2, 168.5, 167.3, 133.8, 132.6, 132.2, 123.5, 123.4, 100.8 (C1), 99.6, 81.7 (C1′), 75.6, 74.5, 71.6, 71.4, 70.1, 69.6, 68.6, 62.3, 54.5, 29.3, 24.0, 21.0, 20.7, 20.6, 19.4, 15.6, 15.0. ESI-HRMS: *m*/*z* calcd for $C_{31}H_{39}O_{13}NSNa$ $[M+Na]^+$ 688.2034, found 688.2016.

4′-Methylphenyl 2-*O*-Benzoyl-3-*O*-benzyl-4,6-*O*-isopropylidene-1-thio-α-L-idopyranoside (27)[4]

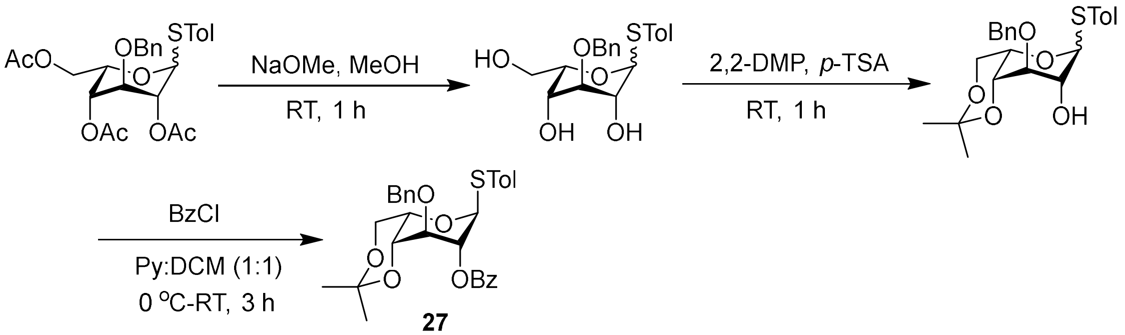

4′-Methylphenyl 2,4,6-tri-*O*-acetyl-3-*O*-benzyl-1-thio-L-idopyranoside[4a] (2.7 g, 5.4 mmol, 1 equiv) was dissolved in dry methanol (25 mL) followed by addition of NaOMe (25 wt% solution in methanol, 350 μL, 1.62 mmol, 0.3 equiv). The reaction mixture was stirred at room temperature under an inert atmosphere. After completion, the reaction was neutralized with Amberlyst 15 (H) resin. After filtration, the crude reaction mixture was evaporated under reduced pressure and directly subjected to the next step.

The crude mixture was dissolved in 2,2-dimethoxypropane (25 mL), under an inert atmosphere. To this solution *p*-TSA (186 mg, 1.08 mmol, 0.2 equiv) was added and the reaction mixture was stirred at room temperature. After 1 h, the reaction was quenched with saturated aqueous $NaHCO_3$ solution (20 mL), the aqueous layer was extracted with EtOAc (3 x 30 mL). The combined organic phases were washed with brine, dried over Na_2SO_4,

filtered, and evaporated under reduced pressure. The crude mixture was dried and subjected to benzoylation without further purification.

The crude mixture was dissolved in dry DCM and Pyridine (1:1, 20 mL) under inert atmosphere and cooled to 0 °C in an ice bath. Benzoyl chloride (1.25 mL, 10.8 mmol, 2 equiv) was added dropwise at 0 °C and the reaction temperature was gradually increased to room temperature. After the consumption of starting material, the reaction was quenched with saturated aqueous $NaHCO_3$ solution (20 mL), the aqueous layer was extracted with EtOAc (3 x 30 mL). The combined organic phases were further washed with saturated aqueous $NaHCO_3$ (to remove benzoic acid) and brine, dried over Na_2SO_4, filtered, and evaporated under reduced pressure. The product, 4′-methylphenyl 2-*O*-benzoyl-3-*O*-benzyl-4,6-*O*-isopropylidene-1-thio-*α*-L-idopyranoside (**27**) was purified by flash column chromatography on silica gel using a hexane/ethyl acetate gradient (10-20%) as a white solid in 49% (1.4 g) overall yield. The experimental data was in accordance with the literature values.[4b]

Glycosylation[4b]

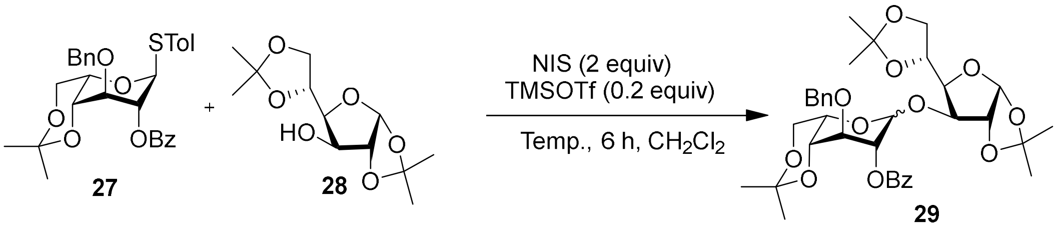

A mixture of donor **27** and acceptor **28** in the specified ratios (see Table), was co-evaporated with toluene three times and then kept under high vacuum with activated 4 Å molecular sieves (3 g/mmol) for 0.5 h. Anhydrous CH_2Cl_2 was added to achieve the desired concentration (see Table), and the mixture was stirred for 0.5 h at room temperature under an argon atmosphere. The reaction mixture was then cooled to -20 °C/-5 °C (bath temperature) and stirred continuously for 0.25 h. NIS (2 equiv, wrt donor) and TMSOTf (0.2 equiv, wrt donor) were added respectively, and the reaction was allowed to proceed at -20 °C/-5 °C for 6 h. Progress of the reaction was monitored by TLC then it was quenched with triethylamine (12 equiv, 0.25 mL). The reaction mixture was diluted with dichloromethane (10 mL), filtered through P8 filter paper, and washed with saturated aqueous $NaHCO_3$ (10 mL x 2) and brine (10 mL). The organic layer was separated, dried with Na_2SO_4, filtered, and concentrated under reduced pressure. The product was purified by flash column chromatography on silica gel using a hexane/ethyl acetate gradient (20-30%) to yield glycoside **29** as an α/β mixture. The anomeric ratio of the product was determined by analyzing the anomeric signals in the ^{1}H NMR spectra of the crude reaction mixture.

Entry	Donor (mmol)	Acceptor (mmol)	Donor Conc (M)	Donor:Acceptor Stoichiometry	CH_2Cl_2 (mL)	Temp (°C)	Yield (%) **29**		1,2-*cis*:1,2-*trans* Ratio	
							Set-1	Set-2	Set-1	Set-2
1	0.15	0.038	0.033	1:0.25	4.5	-20	61	73	1:1.9	1:1.84
2	0.15	0.075	0.033	1:0.5	4.5	-20	73	75	1:1.3	1:1.26
3	0.15	0.15	0.033	1:1	4.5	-20	83	87	1:1.0	1:1.08
4	0.15	0.038	0.2	1:0.25	0.75	-20	69	76	1:1.5	1:1.32
5	0.15	0.075	0.2	1:0.5	0.75	-20	71	82	1:1.3	1:1.2
6	0.15	0.125	0.2	1:0.83	0.75	-20	76	86	1:1.2	1:1.1
7	0.15	0.15	0.2	1:1	0.75	-20	76	82	1:1.0	1:1
8	0.15	0.30	0.2	1:2	0.75	-20	77	77	1:1.0	1:1.1
9	0.15	0.075	0.3	1:0.5	0.5	-20	84	82	1:1.2	1:1.12
10	0.15	0.15	0.3	1:1	0.5	-20	69	84	1:1	1:1
11	0.15	0.075	0.033	1:0.5	4.5	-5	77	75	1:1.37	1:1.36
12	0.15	0.15	0.033	1:1	4.5	-5	83	87	1:1.3	1:1.23
13	0.15	0.075	0.2	1:0.5	0.75	-5	82	86	1:1.3	1:1.26
14	0.15	0.15	0.2	1:1	0.75	-5	73	82	1:1.0	1:1.08
15	0.15	0.075	0.3	1:0.5	0.5	-5	83	86	1:1.2	1:1.2

1,2:5,6-Di-*O*-isopropylidene-3-*O*-(2-*O*-benzoyl-3-*O*-benzyl-4,6-*O*-isopropylidene-α-L-idopyranosyl)-α-D-glucopyranoside (29α)

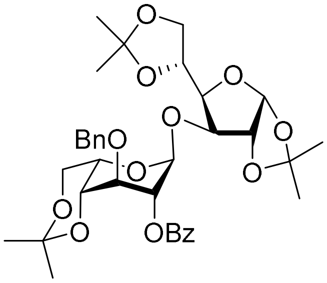

$[\alpha]_D^{23}$ +31.7 (c=1.0, $CHCl_3$); R_f= 0.53 in 30% ethyl acetate in hexane. Yellowish Solid. ^1H NMR (600 MHz, $CDCl_3$, α-isomer) δ 8.14-8.12 (m, 2H, Ar), 7.59-7.56 (m, 1H, Ar), 7.43 (t, J = 7.8 Hz, 2H, Ar), 7.34-7.27 (m, 5H, Ar), 5.91 (d, J = 3.6 Hz, 1H, H1′), 5.20-5.19 (m, 1H, H2), 5.18 (s, 1H, H1), 4.81 (d, J = 11.2 Hz, 1H, CH_2Ph), 4.60 (d, J = 11.2 Hz, 1H, CH_2Ph), 4.52 (d, J = 3.4 Hz, 1H, H2′), 4.41 (d, J = 3.4 Hz, 1H, H3′), 4.30-4.28 (m, 1H, H6a), 4.21 (d, J = 2.1 Hz, 1H, H4′), 4.08-4.03 (m, 2H, H5, H6b), 4.01 (t, J = 2.4 Hz, 1H, H4), 3.95-3.82 (m, 3H, H5′, H6a′, H6b′), 3.72 (t, J = 3.1 Hz, 1H, H3), 1.50 (s, 3H, CH_3), 1.48 (s, 3H, CH_3), 1.47 (s, 3H, CH_3), 1.36 (s, 3H, CH_3), 1.29 (s, 3H, CH_3), 1.18 (s,

3H, CH_3). ^{13}C NMR (151 MHz, CDCl3) δ 165.9, 137.8, 133.5, 130.2, 129.8, 128.5, 128.5, 128.2, 128.1, 112.2, 109.0, 105.5 (C1′), 98.8, 96.2 (C1), 82.4, 81.2, 77.2, 74.8, 72.4, 71.9, 67.8, 67.3, 66.7, 63.0, 60.2, 29.4, 27.1, 27.0, 26.4, 25.6, 18.9. ESI-HRMS: *m*/*z* calcd for $C_{35}H_{44}O_{12}Na$ [M+Na]$^+$ 679.2725, found 679.2701.

1,2:5,6-Di-*O*-isopropylidene-3-*O*-(2-*O*-benzoyl-3-*O*-benzyl-4,6-*O*-isopropylidene-β-L-idopyranosyl)-α-D-glucopyranoside (29β)

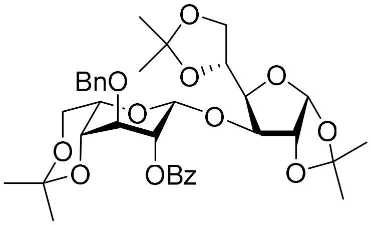

$[\alpha]_D^{23}$ -14.0 (*c*=1.0, $CHCl_3$); R_f= 0.46 in 30% ethyl acetate in hexane, Pale Yellow Solid. ^{1}H NMR (600 MHz, $CDCl_3$, β-isomer) δ 8.18-8.10 (m, 2H, Ar), 7.65-7.55 (m, 1H, Ar), 7.44 (t, *J* = 7.8 Hz, 2H, Ar), 7.39-7.29 (m, 5H, Ar), 6.00 (d, *J* = 3.7 Hz, 1H, H1′), 5.34 (dd, *J* = 3.9, 2.2 Hz, 1H, H2), 5.13 (d, *J* = 2.2 Hz, 1H, H1), 4.87 (d, *J* = 11.8 Hz, 1H, CH_2Ph), 4.66 (d, *J* = 11.8 Hz, 1H, CH_2Ph), 4.58 (d, *J* = 3.7 Hz, 1H, H2′), 4.28 (dd, *J* = 7.4, 3.8 Hz, 1H, H4′), 4.19 (d, *J* = 3.8 Hz, 1H, H3′), 4.12 (dd, *J* = 12.7, 2.7 Hz, 1H, H6a), 4.04-4.03 (m, 1H, H4), 4.00 (d, *J* = 2.4 Hz, 1H, H5), 3.97-3.94 (m, 2H, H6b, H6a′), 3.77 (td, *J* = 7.5, 2.6 Hz, 1H, H5′), 3.72 (t, *J* = 3.9 Hz, 1H, H3), 3.64 (dd, *J* = 11.2, 7.6 Hz, 1H, H6b′), 1.49 (s, 3H, CH_3), 1.47 (d, *J* = 1.7 Hz, 6H, CH_3), 1.33 (s, 3H, CH_3), 1.32 (s, 3H, CH_3), 1.30 (s, 3H, CH_3). ^{13}C NMR (151 MHz, $CDCl_3$) δ 165.6, 138.1, 133.3, 130.2, 130.1, 128.5, 128.4, 127.8, 127.8, 112.3, 106.5 (C1′), 101.1, 99.4, 98.8 (C1), 84.1, 79.7, 75.2, 75.1, 72.0, 71.9, 68.7, 68.1, 67.1, 62.9, 60.5, 29.1, 27.3, 26.7, 24.2, 24.0, 19.1. ESI-HRMS: *m*/*z* calcd for $C_{35}H_{44}O_{12}Na$ [M+Na]$^+$ 679.2725, found 679.2700.

2,4,6-Tri-*O*-acetyl-3-*O*-benzyl-D-glucopyranosyl *N*-phenyltrichloroacetmidate (30)

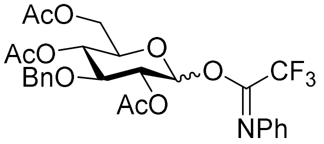

The corresponding hemiacetal was obtained from 1,2:5,6-di-*O*-isopropylidene-D-glucopyranose by adapting previously published procedures.[5] A solution of 2,4,6-tri-*O*-acetyl-3-*O*-benzyl-α-D-glucopyranose (396 mg, 1 mmol, 1 equiv) in DCM (5 mL), maintained under an inert atmosphere, was cooled to 0 °C. DBU (152 µL, 1 mmol, 1 equiv) and 2,2,2-trifluoro-*N*-phenylacetimidoyl chloride (323 µL, 2 mmol, 2 equiv) were then added. The reaction mixture was allowed to warm gradually to room temperature and stirred for an hour. DCM was evaporated under reduced pressure, and the resulting crude reaction mixture was subjected directly to flash chromatography over silica gel to obtain *N*-phenyltrifluoroacetimidoyl 2,4,6-tri-*O*-acetyl-3-*O*-benzyl-*α*-D-glucopyranoside **(30)** using a hexane/ethyl acetate gradient (30-40%).as a white solid as a α/β-mixture in 61% yield (346 mg).

2,4,6-Tri-*O*-acetyl-3-*O*-benzyl-α-D-glucopyranosyl *N*-phenyltrichloroacetmidate (30α)

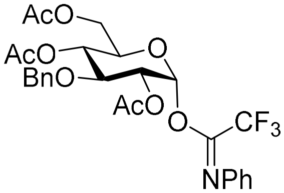

[α]$_D$^{23} +69.8 (*c*=1.0, $CHCl_3$), R_f= 0.5 in 30% ethyl acetate in hexane. Colorless liquid. ^{1}H NMR (600 MHz, C_6D_6) δ 7.26-7.18 (m, 2H, Ar), 7.13 (t, *J* = 7.6 Hz, 2H, Ar), 7.06-7.01 (m, 3H, Ar), 6.87-6.85 (m, 1H, Ar), 6.79 (br s, 1H, H1) 6.68 (d, *J* = 7.8 Hz, 2H, Ar), 5.38 (t, *J* = 9.8 Hz, 1H, H4), 5.17 (br s, 1H, H2), 4.57 (d, *J* = 12.1 Hz, 1H, CH_2Ph), 4.49 (d, *J* = 12.0 Hz, 1H, CH_2Ph), 4.25 (dd, *J* = 12.9, 5.2 Hz, 1H, H6), 4.19 – 3.96 (m, 3H, H3, H5, H6), 1.73 (s, 3H), 1.60 (s, 3H), 1.58 (s, 3H). ^{13}C NMR (151 MHz, C_6D_6) δ 170.4, 169.5, 169.3, 143.9, 139.0, 129.5, 129.0, 128.7, 128.5, 128.3, 127.9, 125.2, 120.0, 93.9, 77.9, 75.2, 72.5, 71.8, 69.6, 62.3, 20.6, 20.6, 20.4. ^{19}F NMR (564 MHz, C_6D_6) δ -65.5, -82.3. ESI-HRMS: *m/z* calcd for $C_{27}H_{28}O_9NF_3Na$ $[M+Na]^+$ 590.1608, found 590.1603.

2,4,6-Tri-*O*-acetyl-3-*O*-benzyl-β-D-glucopyranosyl *N*-phenyltrichloroacetmidate (30β)

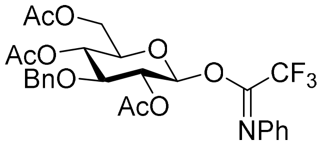

[α]$_D$^{23} +27.2 (*c*=1.0, $CHCl_3$), R_f= 0.42 in 30% ethyl acetate in hexane. White Solid. ^{1}H NMR (600 MHz, C_6D_6) δ 7.23-7.22 (m, 2H, Ar), 7.13 (t, *J* = 7.6 Hz, 2H, Ar), 7.07-7.02 (m, 3H, Ar), 6.87-6.84 (m, 1H, Ar), 6.77 (d, *J* = 7.7 Hz, 2H, Ar), 5.79 (s, 1H, H1), 5.53 (t, *J* = 8.3 Hz, 1H, H2), 5.32 (t, *J* = 9.4 Hz, 1H, H4), 4.48 (d, *J* = 3.4 Hz, 2H, CH_2Ph), 4.20 (dd, *J* = 12.4, 5.0 Hz, 1H, H6), 4.10 (d, *J* = 12.5 Hz, 1H, H6), 3.58 (br s, 1H, H3), 3.26 (br s, 1H, H5), 1.71 (s, 3H), 1.65 (s, 3H), 1.57 (s, 3H). ^{13}C NMR (151 MHz, C_6D_6) δ 170.5, 169.2, 168.7, 144.1, 138.7, 129.5, 129.0, 128.7, 128.5, 128.4, 128.3, 125.2, 119.9, 95.9 (C1), 80.2, 73.9, 73.6, 73.6, 71.7, 69.2, 62.2, 20.6, 20.6, 20.5. ^{19}F NMR (564 MHz, C_6D_6) δ -65.7, -75.6. ESI-HRMS: *m/z* calcd for $C_{27}H_{28}O_9NF_3Na$ $[M+Na]^+$ 590.1608, found 590.1598.

1,2,4,6-Tetra-*O*-acetyl-β-D-glucopyranose (31β)

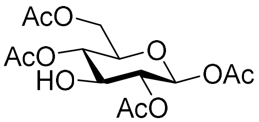

To a solution of 1,2,4,6-tetra-*O*-acetyl-3-*O*-benzyl-β-D-glucopyranose[5a] (4.3 g, 10 mmol, 1 equiv.) in methanol (50 mL) was added Pd/C (10% Pd, 1 equiv by mass) and the suspension was stirred vigorously under 1 atm of H_2 (balloon) for 5 h. The palladium on carbon was filtered through a Celite pad and the filtrate was concentrated under reduced pressure. The desired compound, acetyl 2,4,6-tri-*O*-acetyl-β-D-glucopyranoside (**31)** was obtained by flash chromatography over silica gel using a hexane/ethyl acetate (60-70%) as a white solid and pure β anomer in 74% yield (2.6g). **[α]$_D$^{23}** -2.0 (*c*=1.0, $CHCl_3$), R_f= 0.2 in 50% ethyl acetate in hexane. White Solid. ^{1}H NMR (600 MHz, $CDCl_3$) δ 5.64 (dd, *J* = 8.3, 1.2 Hz, 1H, H1), 5.00-4.95 (m, 2H, H2, H4), 4.26 (ddd, *J* = 12.5, 4.8, 1.4 Hz, 1H, H6a), 4.11-4.08 (m, 1H, H6b), 3.77-3.73 (m, 2H, H3, H5), 2.94 (Br S, 1H, OH), 2.09 (s, 6H, CH_3), 2.08 (s, 3H, CH_3), 2.06 (s, 3H, CH_3). ^{13}C NMR (151 MHz, $CDCl_3$) δ 170.9, 170.8, 170.5, 169.4, 91.8 (C1), 73.9,

73.0, 72.9, 70.4, 61.9, 21.0, 20.9, 20.9, 20.9. ESI-HRMS: *m/z* calcd for $C_{14}H_{20}O_{10}Na$ $[M+Na]^+$ 371.0948, found 371.0941.

Glycosylation[6]

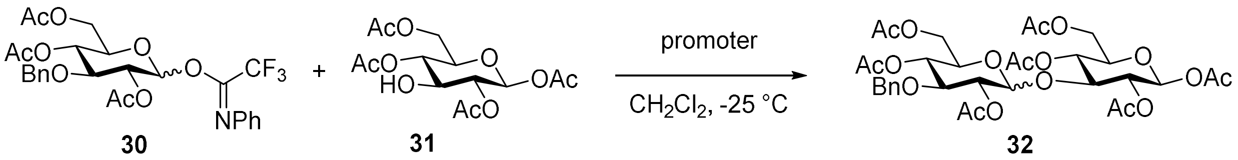

A mixture of the donor **30** and acceptor **31** in the specified ratios (See Table) was co-evaporated with toluene three times and then kept under high vacuum with activated 4 Å molecular sieves (3 g/mmol) for 0.5 h. Anhydrous CH_2Cl_2 was subsequently added to give the required concentration (see Table), and the mixture was stirred for an additional 0.5 h at room temperature under an argon atmosphere. The reaction mixture was then cooled to -25 °C (bath temp.) and stirred continuously for 0.25 h. TMSOTf (0.2 equiv, wrt donor) / $BF_3.OEt_2$ (1.5 equiv, wrt donor) was added at the same temperature, and the reaction mixture was stirred for 6 h at -25 °C before being quenched with triethylamine (12 equiv, 0.2 mL). Progress of the reaction was monitored by TLC. The mixture was then diluted with dichloromethane (10 mL), filtered through P8 filter paper, and washed with saturated aqueous $NaHCO_3$ (10 mL x 2) and brine (10 mL). The organic layer was separated, dried over Na_2SO_4, filtered, and concentrated under reduced pressure. The products were purified by flash column chromatography on silica gel eluting with a hexane/ethyl acetate gradient (60-70%), yielding the glycoside **32** as a white solid in the form of α/β-mixture. The anomeric ratio of the products was determined from the crude reaction mixtures by integrating the anomeric signals in the ^1H NMR spectra.

Entry	Donor (mmol)	Acceptor (mmol)	Donor Conc (M)	Donor:Acceptor Stoichiometry	CH_2Cl_2 (mL)	Promoter (equiv)	Yield (%) **32**		1,2-*cis*:1,2-*trans* Ratio	
							Set-1	Set-2	Set-1	Set-2
1	0.12	0.12	0.033	1:1	3.6	TMSOTf (0.2)	87	92	1:1.3	1:1.43
2	0.12	0.12	0.2	1:1	0.6	TMSOTf (0.2)	81	83	1:1.25	1:1.34
3	0.12	0.12	0.3	1:1	0.4	TMSOTf (0.2)	85	87	1:1.2	1:1.28
4	0.12	0.12	0.033	1:1	3.6	$BF_3.Et_2O$ (1.5)	70	68	1:3.0	1:3.0
5	0.12	0.12	0.2	1:1	0.6	$BF_3.Et_2O$ (1.5)	75	78	1:2.9	1:2.86
6	0.12	0.12	0.25	1:1	0.48	$BF_3.Et_2O$ (1.5)	72	76	1:2.2	1:2.01
7	0.12	0.12	0.3	1:1	0.4	$BF_3.Et_2O$ (1.5)	66	80	1:2.4	1:2.65

8	0.12	0.12	0.35	1:1	0.34	$BF_3.Et_2O$ (1.5)	75	71	1:2.45	1:2.79
9	0.12	0.12	0.4	1:1	0.3	$BF_3.Et_2O$ (1.5)	68	72	1:2.7	1:2.85

1,2,4,6-Tetra-*O*-acetyl-3-*O*-(2,4,6-tri-*O*-acetyl-3-*O*-benzyl-α-D-glucopyranosyl)-β-D-glucpoyranose (32α)

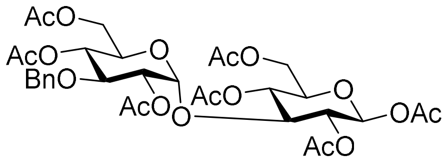

$[\alpha]_D^{23}$ +42.1 (*c*=1.0, $CHCl_3$), R_f= 0.28 in 50% ethyl acetate in hexane. White Solid. ^{1}H NMR (600 MHz, $CDCl_3$) δ 7.34-7.31 (m, 2H, Ar), 7.28 (d, *J* = 7.3 Hz, 1H, Ar), 7.25-7.23 (m, 2H, Ar), 5.61 (d, *J* = 8.3 Hz, 1H, H1′), 5.25 (d, *J* = 3.5 Hz, 1H, H1), 5.21 (t, *J* = 9.7 Hz, 1H, H4′), 5.18 (dd, *J* = 9.6, 8.3 Hz, 1H, H2′), 5.04 (dd, *J* = 10.3, 9.3 Hz, 1H, H4), 4.75 (dd, *J* = 10.2, 3.5 Hz, 1H, H2), 4.66 (d, *J* = 11.8 Hz, 1H, CH_2Ph), 4.56 (d, *J* = 11.8 Hz, 1H, CH_2Ph), 4.24 (dd, *J* = 12.5, 4.6 Hz, 1H, H6a′), 4.15 (dd, *J* = 12.5, 4.0 Hz, 1H, H6a), 4.08 (ddd, *J* = 14.5, 12.6, 2.3 Hz, 2H, H6b′, H6b), 3.96 (t, *J* = 9.4 Hz, 1H, H3′), 3.92 (ddd, *J* = 10.4, 4.0, 2.4 Hz, 1H, H5), 3.85 (t, *J* = 9.8 Hz, 1H, H3), 3.72 (ddd, *J* = 10.0, 4.6, 2.3 Hz, 1H, H5′), 2.11 (s, 3H, CH_3), 2.09 (s, 6H, CH_3), 2.07 (s, 3H, CH_3), 2.05 (s, 3H, CH_3), 2.04 (s, 3H, CH_3), 1.88 (s, 3H, CH_3). ^{13}C NMR (151 MHz, $CDCl_3$) δ 170.9, 170.8, 170.7, 169.6, 169.4, 169.2, 138.2, 128.6, 127.9, 127.8, 96.2 (C1), 92.0 (C1′), 76.6, 76.5, 75.3, 73.4, 73.0, 71.1, 69.5, 69.4, 68.9, 61.7, 61.6, 21.1, 21.1, 21.0, 20.9, 20.9, 20.9, 20.8. ESI-HRMS: *m*/*z* calcd for $C_{33}H_{42}O_{18}Na$ $[M+Na]^+$ 749.2263, found 749.2239.

1,2,4,6-Tetra-*O*-acetyl-3-*O*-(2,4,6-tri-*O*-acetyl-3-*O*-benzyl-β-D-glucopyranosyl)-β-D-glucopyranose (32β)

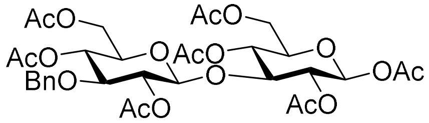

$[\alpha]_D^{23}$ -11.3 (*c*=1.0, $CHCl_3$), R_f= 0.23 in 50% ethyl acetate in hexane. White Solid. ^{1}H NMR (600 MHz, $CDCl_3$) ^{1}H NMR (600 MHz, CDCl3) δ 7.32-7.28 (m, 2H, Ar), 7.28-7.24 (m, 1H, Ar), 7.21-7.19 (m, 2H, Ar), 5.61 (d, *J* = 8.3 Hz, 1H, H1′), 5.11-5.08 (m, 2H, H4, H2′), 4.99 (t, *J* = 9.6 Hz, 1H, H4′), 4.94 (dd, *J* = 9.6, 8.1 Hz, 1H, H2), 4.55 (s, 2H, CH_2Ph), 4.50 (d, *J* = 8.1 Hz, 1H, H1), 4.28 (dd, *J* = 12.4, 4.8 Hz, 1H, H6a), 4.19 (dd, *J* = 12.5, 4.7 Hz, 1H, H6a′), 4.13 (dd, *J* = 12.4, 2.3 Hz, 1H, H6b′), 4.04 (dd, *J* = 12.3, 2.4 Hz, 1H, H6b), 3.92 (t, *J* = 9.3 Hz, 1H, H3′), 3.79 (ddd, *J* = 10.2, 5.0, 2.2 Hz, 1H, H5′), 3.64 (t, *J* = 9.4 Hz, 1H, H3), 3.58 (ddd, *J* = 10.0, 4.9, 2.4 Hz, 1H, H5), 2.09 (s, 6H, CH_3), 2.07 (s, 6H, CH_3), 2.03 (s, 3H, CH_3), 1.97 (s, 3H, CH_3), 1.96 (s, 3H, CH_3). ^{13}C NMR (151 MHz, CDCl3) δ 170.9, 170.8, 169.4, 169.4, 169.3, 169.3, 168.9, 137.7, 128.6, 128.0, 101.4 (C1), 91.9 (C1′), 80.1, 78.7, 73.6, 73.0, 72.3, 72.2, 72.2, 69.4, 67.9, 62.2, 61.9, 21.0, 20.9, 20.9, 20.9, 20.8, 20.6. ESI-HRMS: *m*/*z* calcd for $C_{33}H_{42}O_{18}Na$ $[M+Na]^+$ 749.2263, found 749.2230.

References

1. (a) Kurfirt, M.; Dracinsky, M.; Stastna, L. C.; Curinova, P.; Hamala, V.; Hovorkova, M.; Bojarova, P.; Karban, J. Selectively Deoxyfluorinated *N*-acetyllactosamine analogues as ^{19}F NMR probes to study carbohydrate-galectin interactions. *Chem. Eur. J.* **2021**, *27*, 13040-13051. (b) Peters, T.; Weimar, T. Improved synthesis of a-L-Fuc(1→4)-P-D-GlcNAc and a-L-fuc(1→6)-P-DGlcNAc building blocks: a convergent strategy employing 4-*O*→6-*O* acetyl migration; NOE data of the protected α-1,4-linked disaccharide. *Liebigs Ann. Chem.* **1991**, 237-242.
2. (a) Doyle, L. M.; O'Sullivan, S.; Salvo, C. D.; McKinney, M.; McArdle, P.; Murphy, P. V. Stereoselective epimerizations of glycosyl thiols. *Org. Lett.* **2017**, *19*, 5802-5805. (b) Zhao, G.; Yao, W.; Kevlishvili, I.; Mauro, J. N.; Liu, P.; Ngai, M.-Y. Nickel-catalyzed radical migratory coupling enables C-2 arylation of carbohydrates. *J. Am. Chem. Soc.* **2021**, *143*, 8590-8596.
3. Spijker, N. M.; van Boeckel, C. A. A. Double stereodifferentiation in carbohydrate coupling reactions: the mismatched interaction of donor and acceptor as an unprecedented factor governing the α/β ratio of glycoside formation. *Angew. Chem. Int. Ed. Engl.* **1991**, *30*, 180-183.
4. (a) Hung, S.-C.; Lu, X.-A.; Lee, J.-C.; Chang, M. D.-T.; Fang, S.-L.; Fan, T.-C.; Zulueta, M. M. L.; Zhong, Y.-Q.; Synthesis of heparin oligosaccharides and their interaction with eosinophil-derived neurotoxin. *Org. Biomol. Chem.* **2012**, *10*, 760-772. (b) Mohamed, S.; He, Q. Q.; Lepage, R. J.; Krenske, E. H.; Ferro, V. Glycosylations of simple acceptors with 2-*O*-acyl L-idose or L-iduronic acid donors reveal only a minor role for neighbouring-group participation. *Eur. J. Org. Chem.* **2018**, 2214-2227.
5. (a) Virlouvet, M.; Gartner, M.; Koroniak, K.; Sleeman, J. P.; Braese, S. Multi-gram synthesis of a hyaluronic acid subunit and synthesis of fully protected oligomers. *Adv. Synth. & Catal.* **2010**, *352*, 2657-2662. (b) Cai, T. B.; Lu, D.; Tang, X.; Zhang, Y.; Landerholm, M.; Wang, P. G. New glycosidase activated nitric oxide donors: glycose and 3-morphorlinosydnonimine conjugates. *J. Org. Chem.* **2005**, *70*, 3518-3524.
6. Yu, H.; Williams, D. L.; Ensley, H. E. 4-Acetoxy-2,2-dimethylbutanoate: a useful carbohydrate protecting group for the selective formation of β-(1→3)-D-glucans. *Tet. Lett.* **2005**, *46*, 3417–3421.

^{1}H NMR (600 MHz, CDCl$_3$) spectrum of ethyl 2-deoxy-4,6-*O*-isopropylidene-2-phthalimido-1-thio-β-D-glucopyranose (**18**)

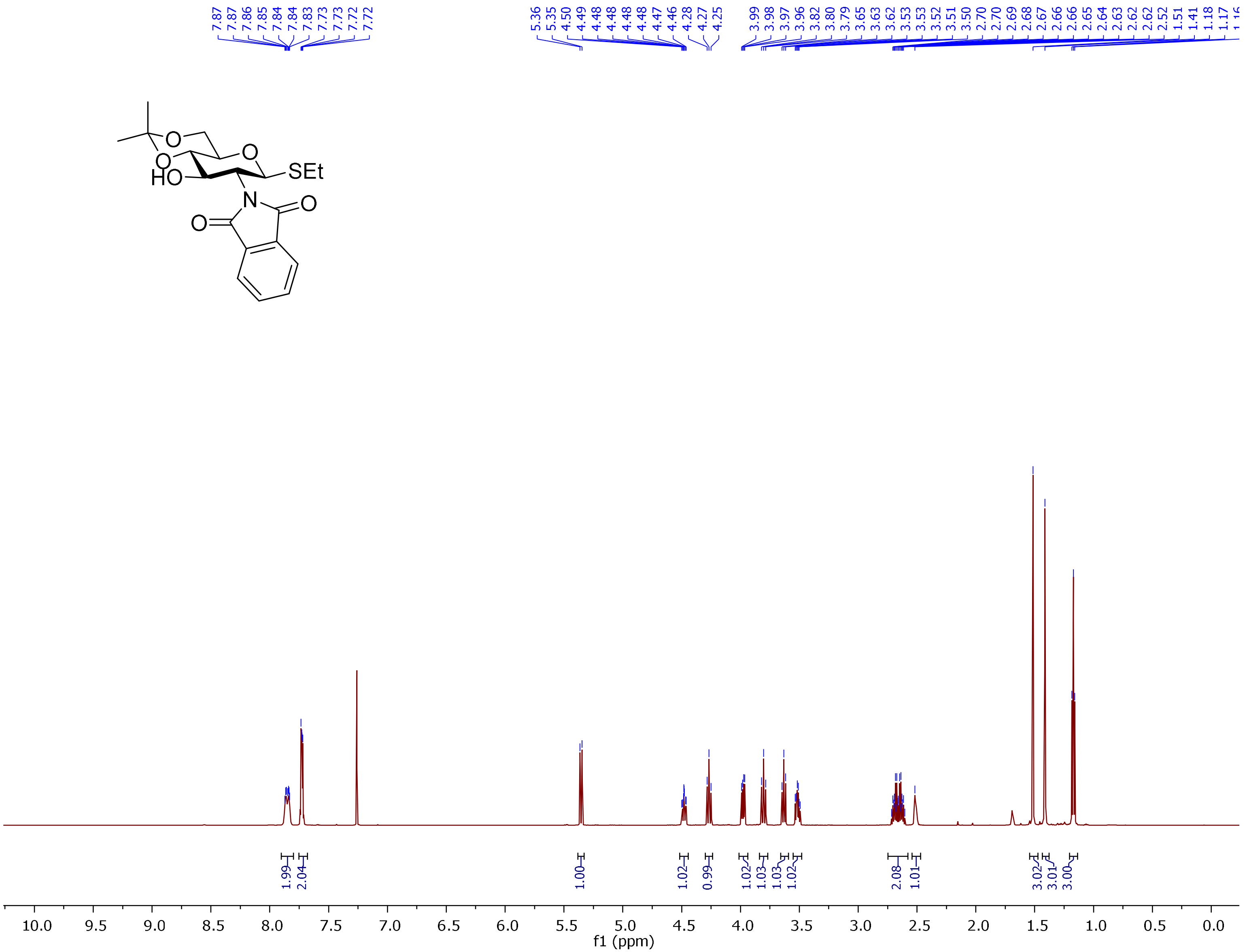

$^{13}C\{^1H\}$ NMR (151 MHz, $CDCl_3$) spectrum of ethyl 2-deoxy-4,6-*O*-isopropylidene-2-phthalimido-1-thio-β-D-glucopyranose (**18**)

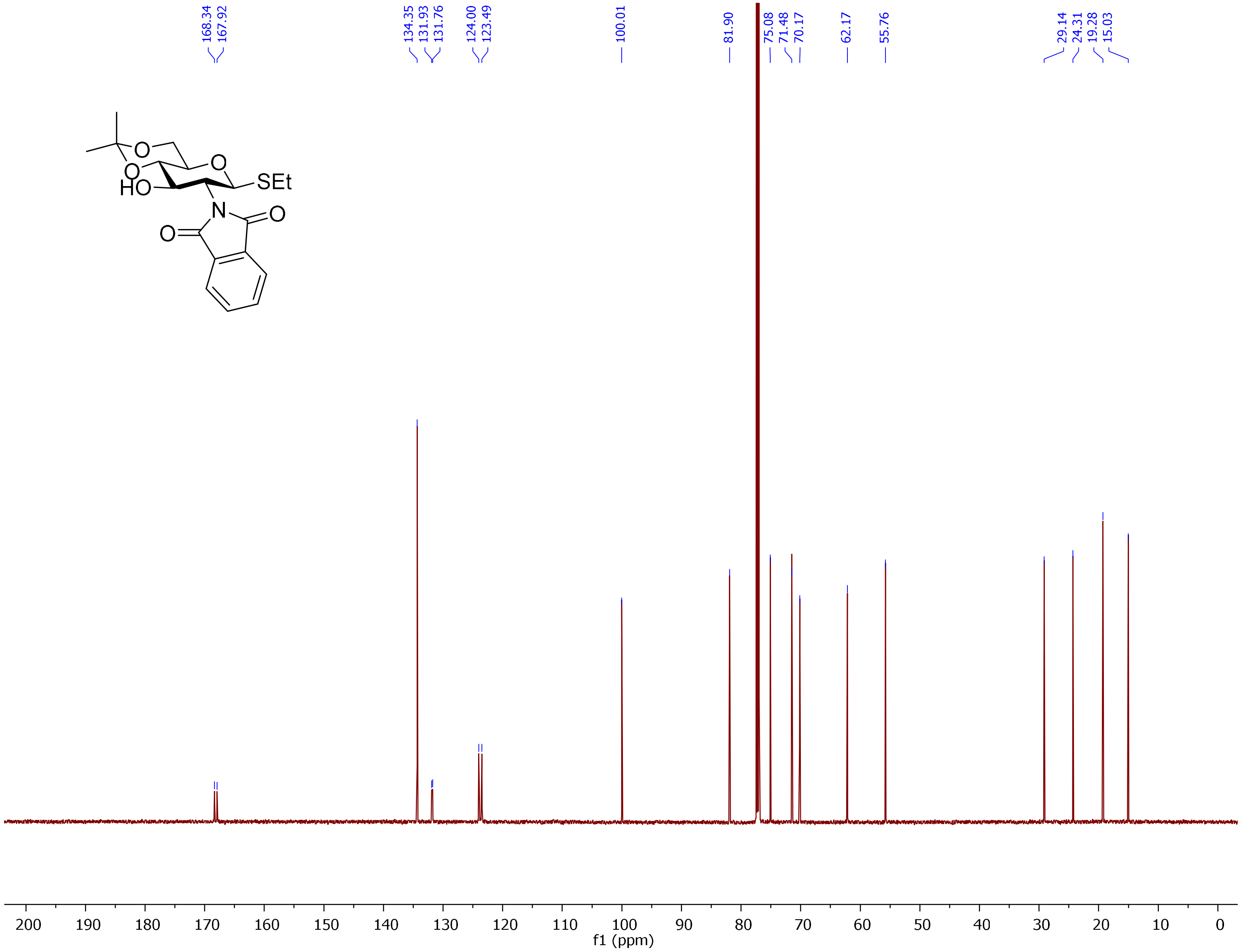

COSY NMR (600 MHz, $CDCl_3$) spectrum of ethyl 2-deoxy-4,6-*O*-isopropylidene-2-phthalimido-1-thio-β-D-glucopyranose (**18**)

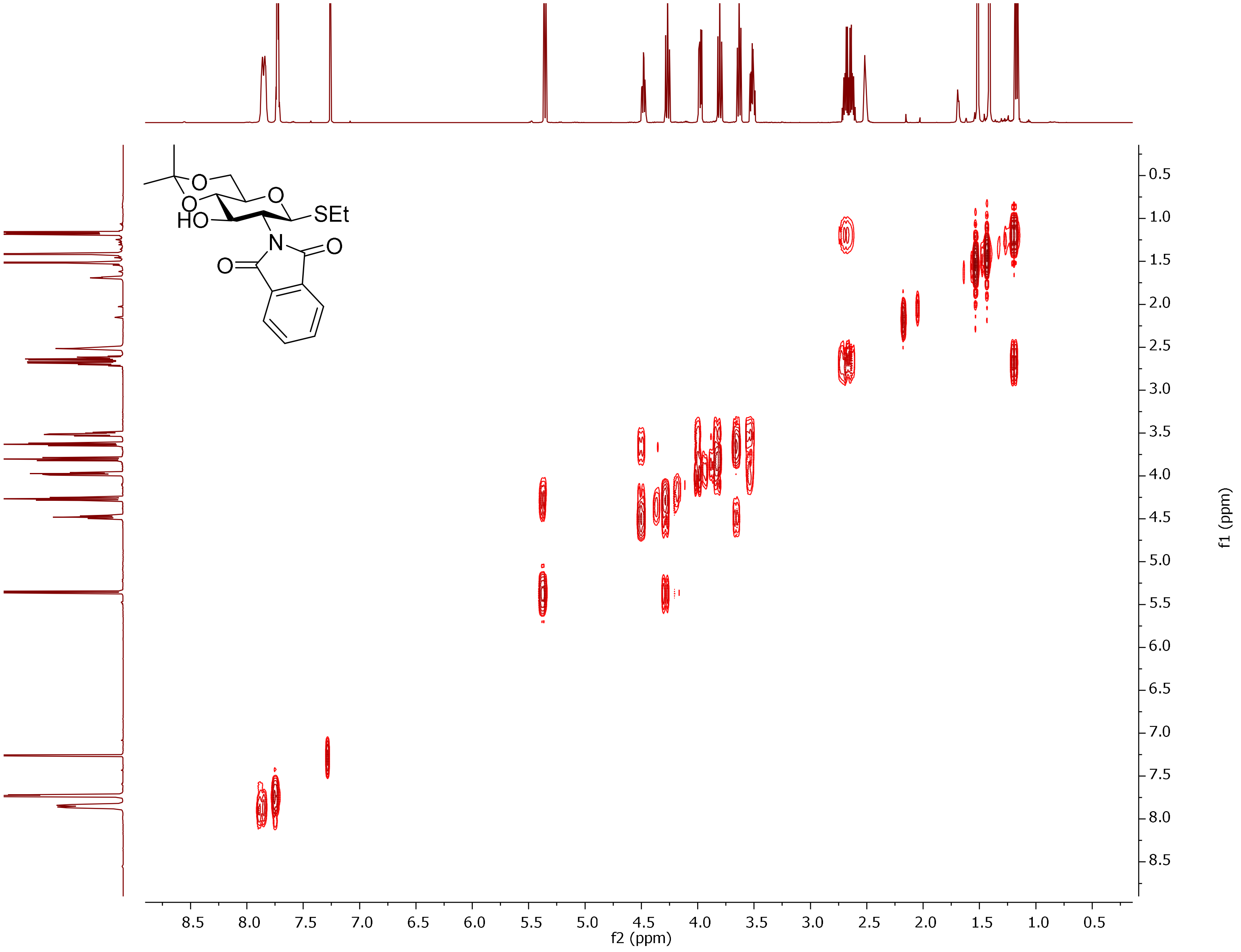

HSQC NMR (600 MHz, $CDCl_3$) spectrum of ethyl 2-deoxy-4,6-*O*-isopropylidene-2-phthalimido-1-thio-β-D-glucopyranose (**18**)

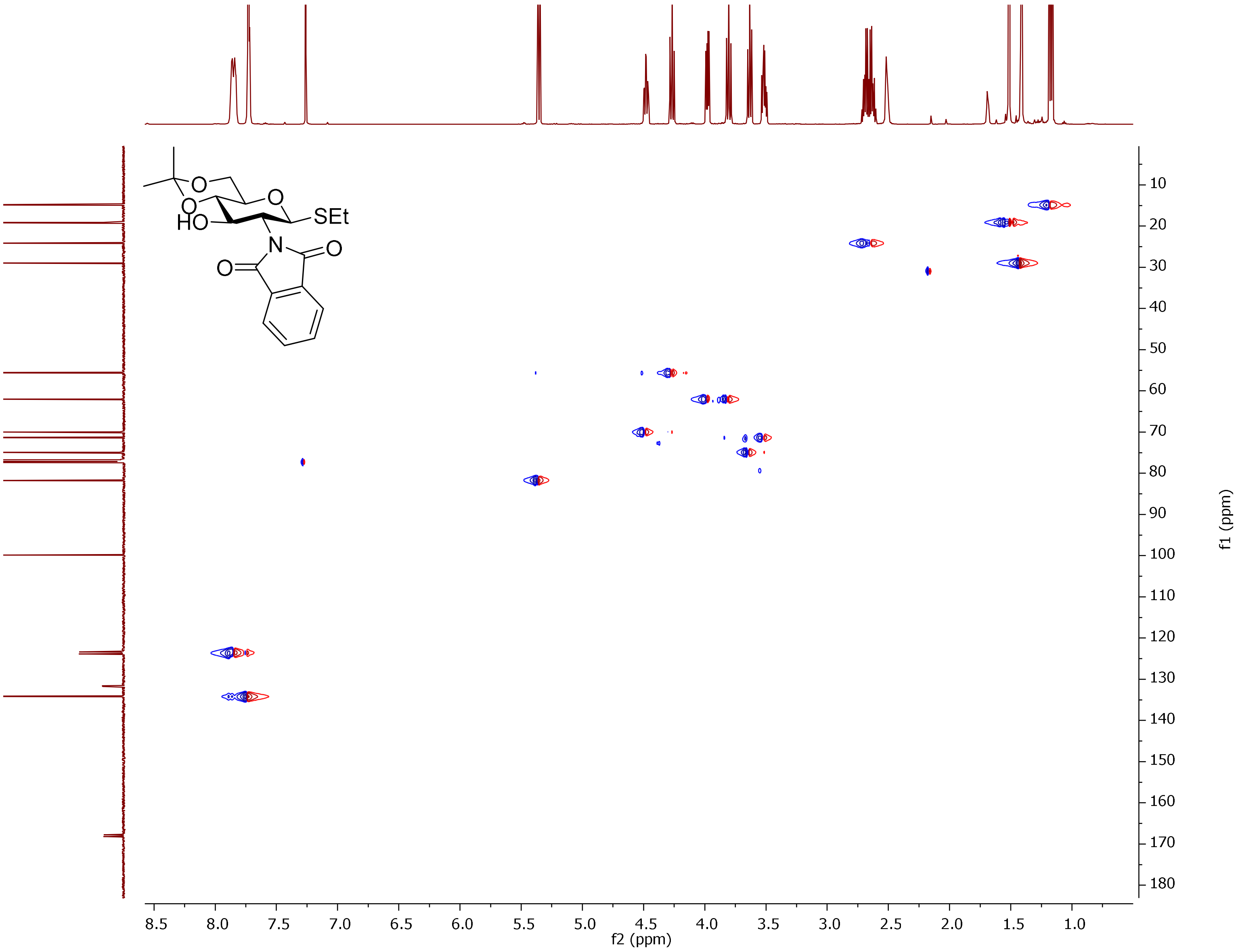

^{1}H NMR (600 MHz, $CDCl_3$) spectrum of ethyl 2-deoxy-2-phthalimido-4,6-*O*-isopropylidene-3-*O*-(2,3,4-tri-*O*-benzoyl-α-D-fucopyranosyl)-1-thio-β-D-glucopyranoside (**21α**)

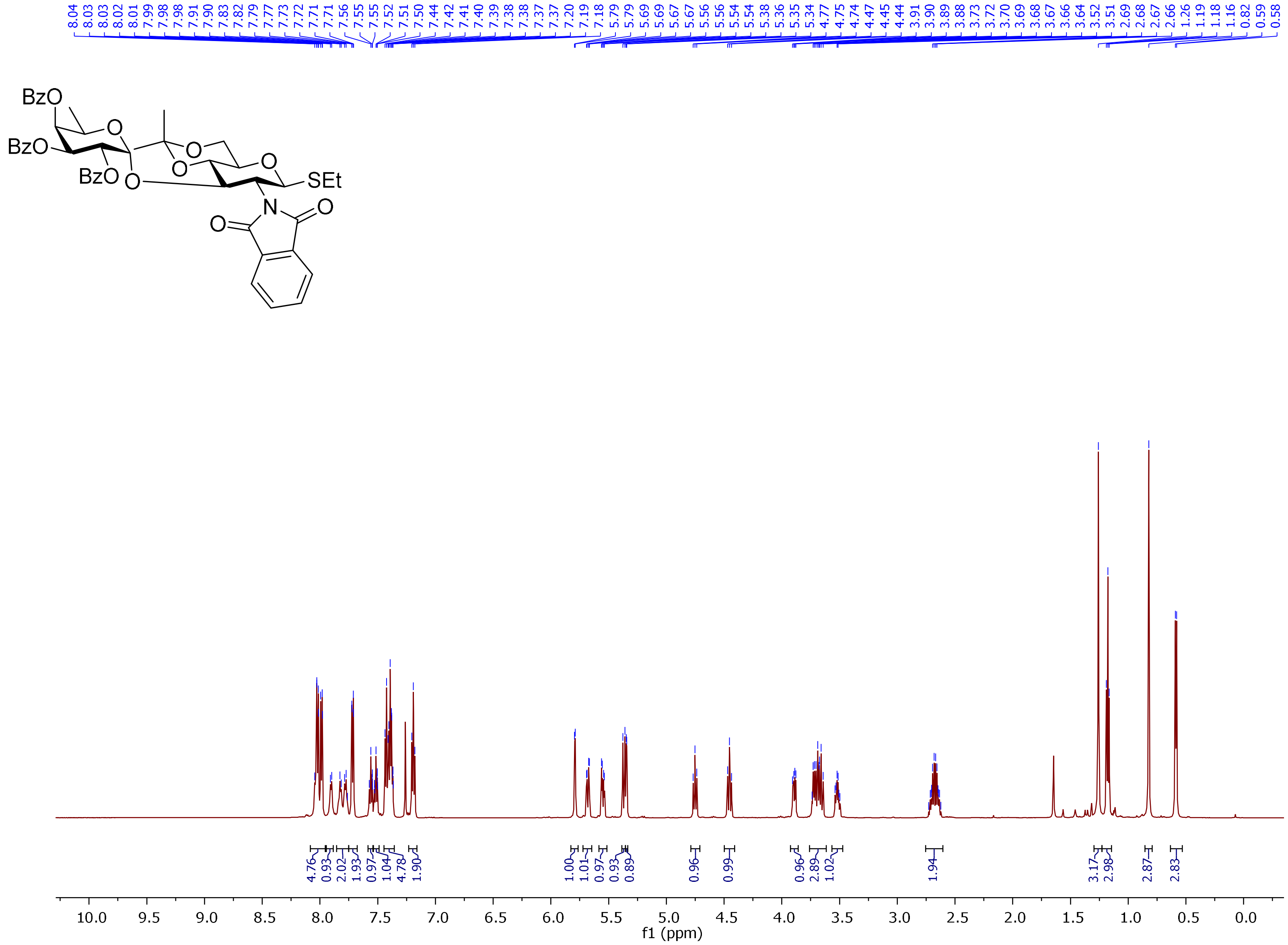

$^{13}C\{^{1}H\}$ NMR (151 MHz, $CDCl_3$) spectrum of ethyl 2-deoxy-2-phthalimido-4,6-*O*-isopropylidene-3-*O*-(2,3,4-tri-*O*-benzoyl-α-D-fucopyranosyl)-1-thio-β-D-glucopyranoside (**21α**)

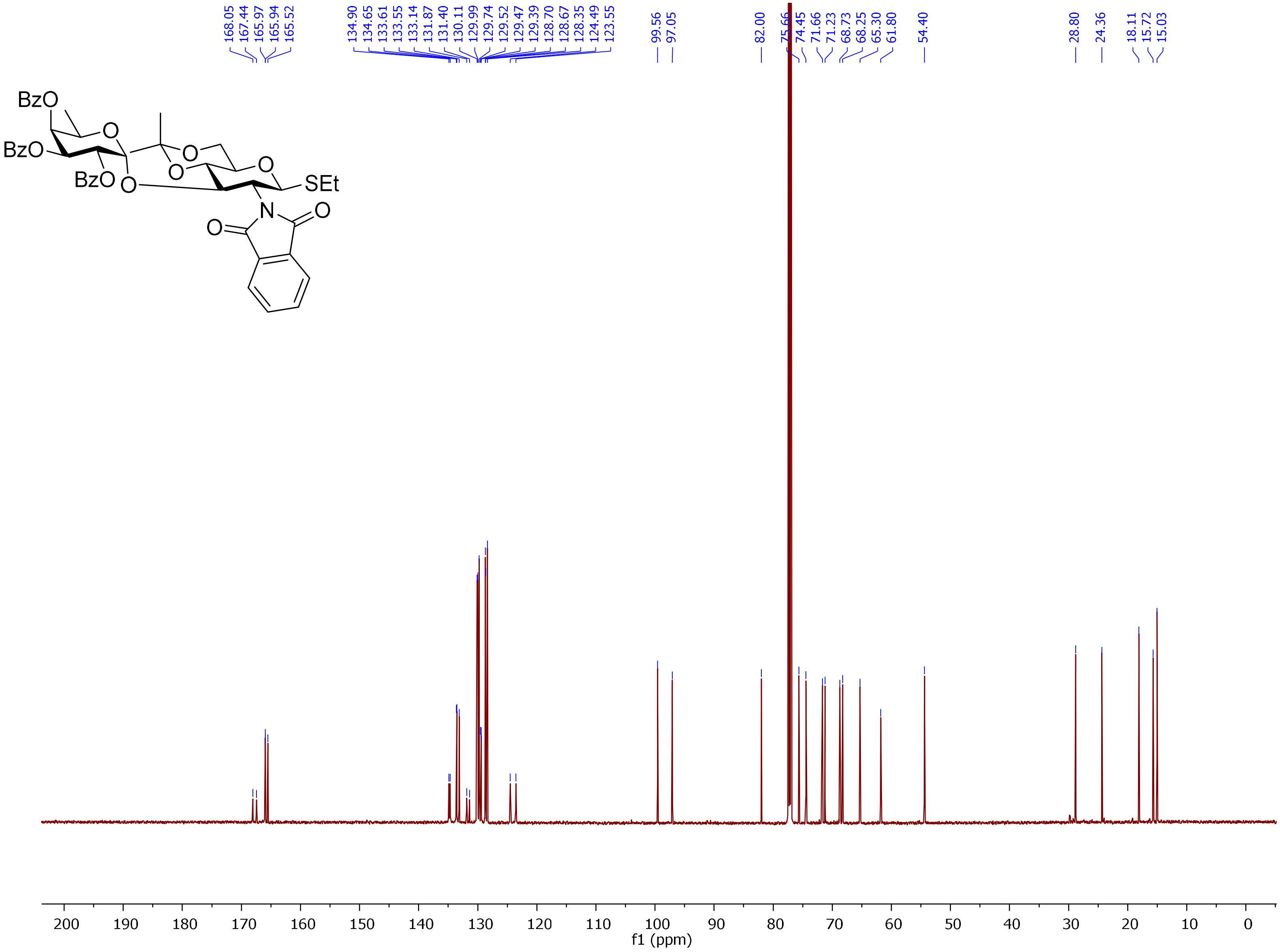

COSY NMR (600 MHz, $CDCl_3$) spectrum of ethyl 2-deoxy-2-phthalimido-4,6-*O*-isopropylidene-3-*O*-(2,3,4-tri-*O*-benzoyl-α-D-fucopyranosyl)-1-thio-β-D-glucopyranoside (**21α**)

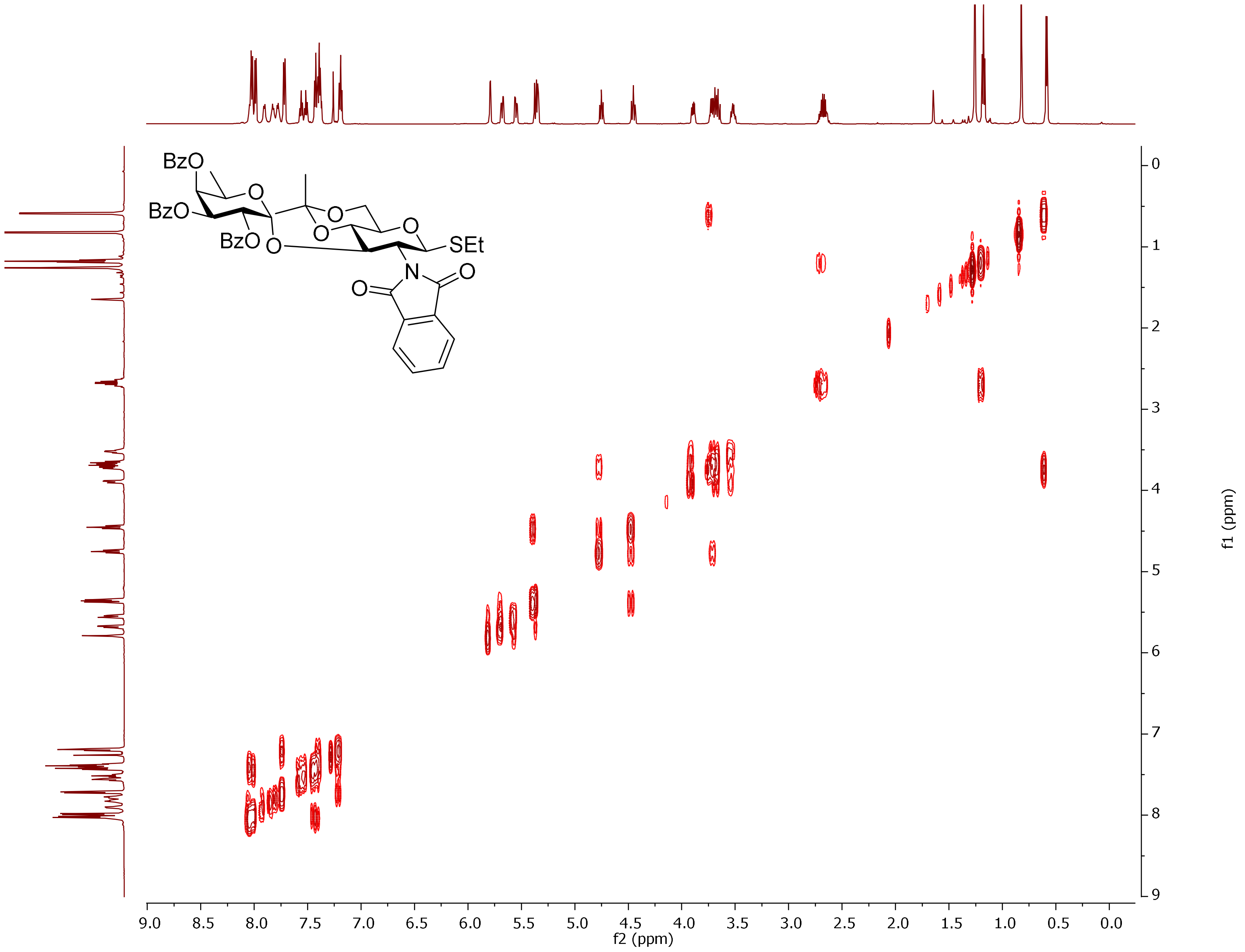

HSQC NMR (600 MHz, $CDCl_3$) spectrum of ethyl 2-deoxy-2-phthalimido-4,6-*O*-isopropylidene-3-*O*-(2,3,4-tri-*O*-benzoyl-α-D-fucopyranosyl)-1-thio-β-D-glucopyranoside (**21α**)

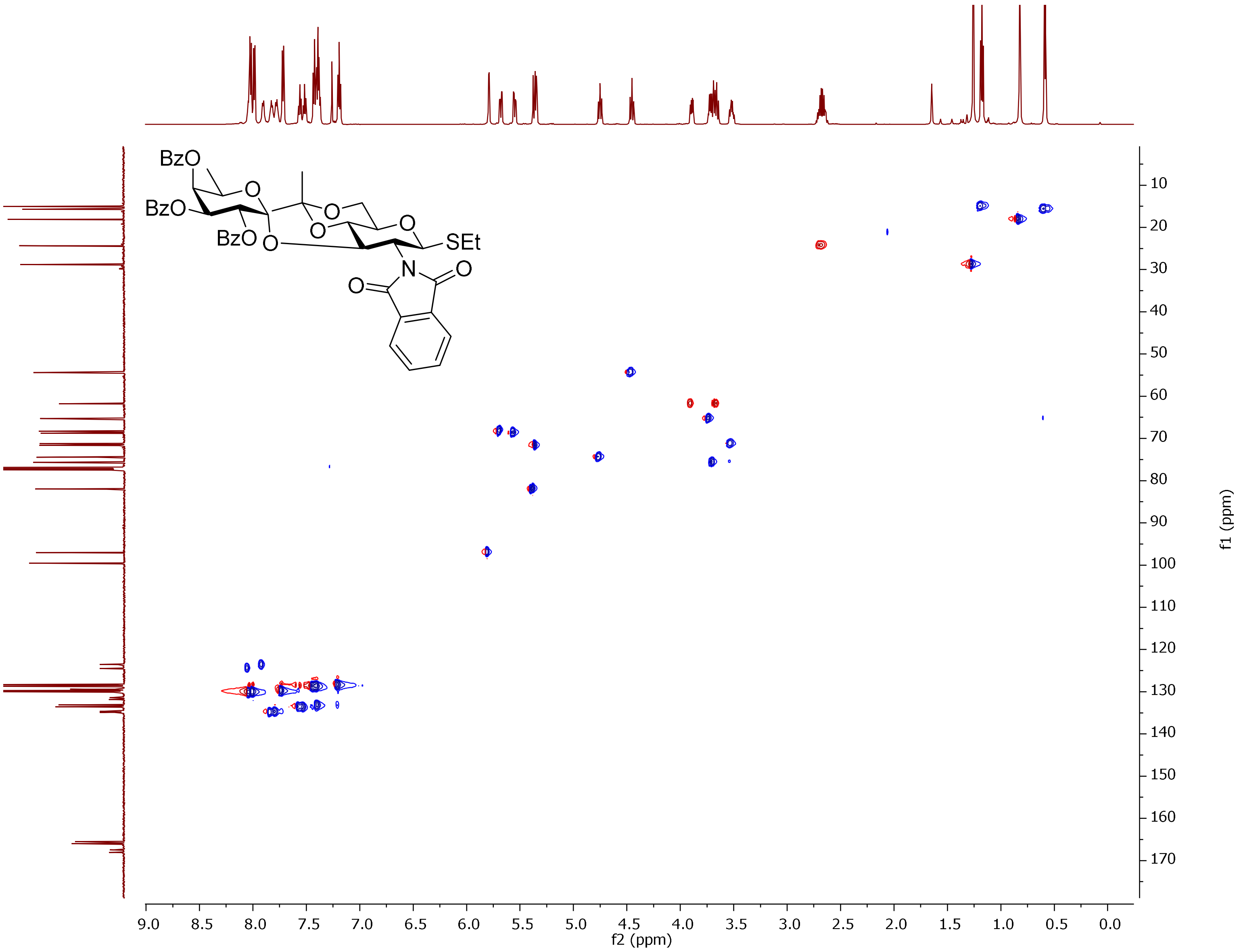

HMBC NMR (600 MHz, $CDCl_3$) spectrum of ethyl 2-deoxy-2-phthalimido-4,6-*O*-isopropylidene-3-*O*-(2,3,4-tri-*O*-benzoyl-α-D-fucopyranosyl)-1-thio-β-D-glucopyranoside (**21α**)

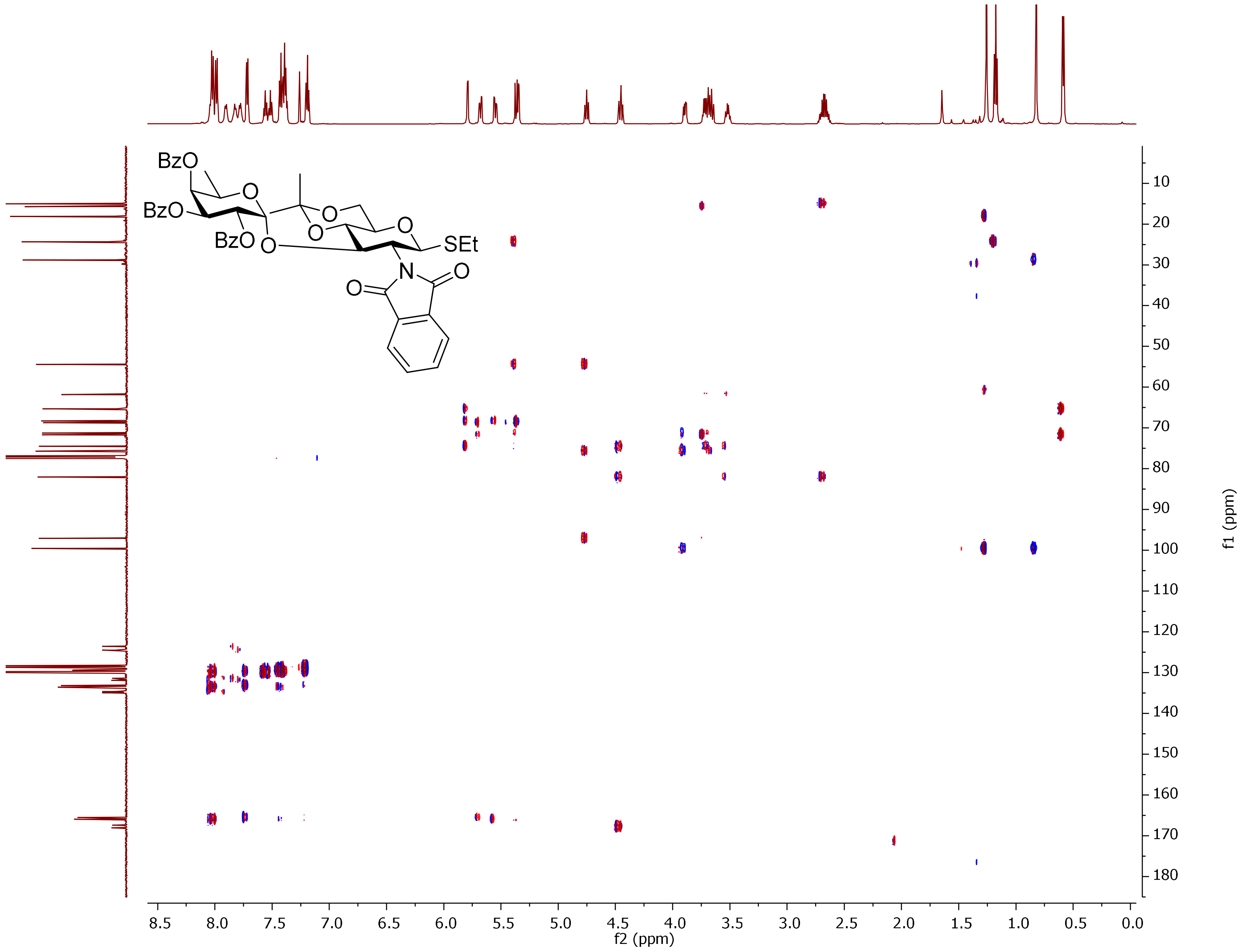

^{1}H NMR (600 MHz, $CDCl_3$) spectrum of ethyl 2-deoxy-2-phthalimido-4,6-*O*-isopropylidene-3-*O*-(2,3,4-tri-*O*-benzoyl-β-D-fucopyranosyl)-1-thio-β-D-glucopyranoside (**21β**)

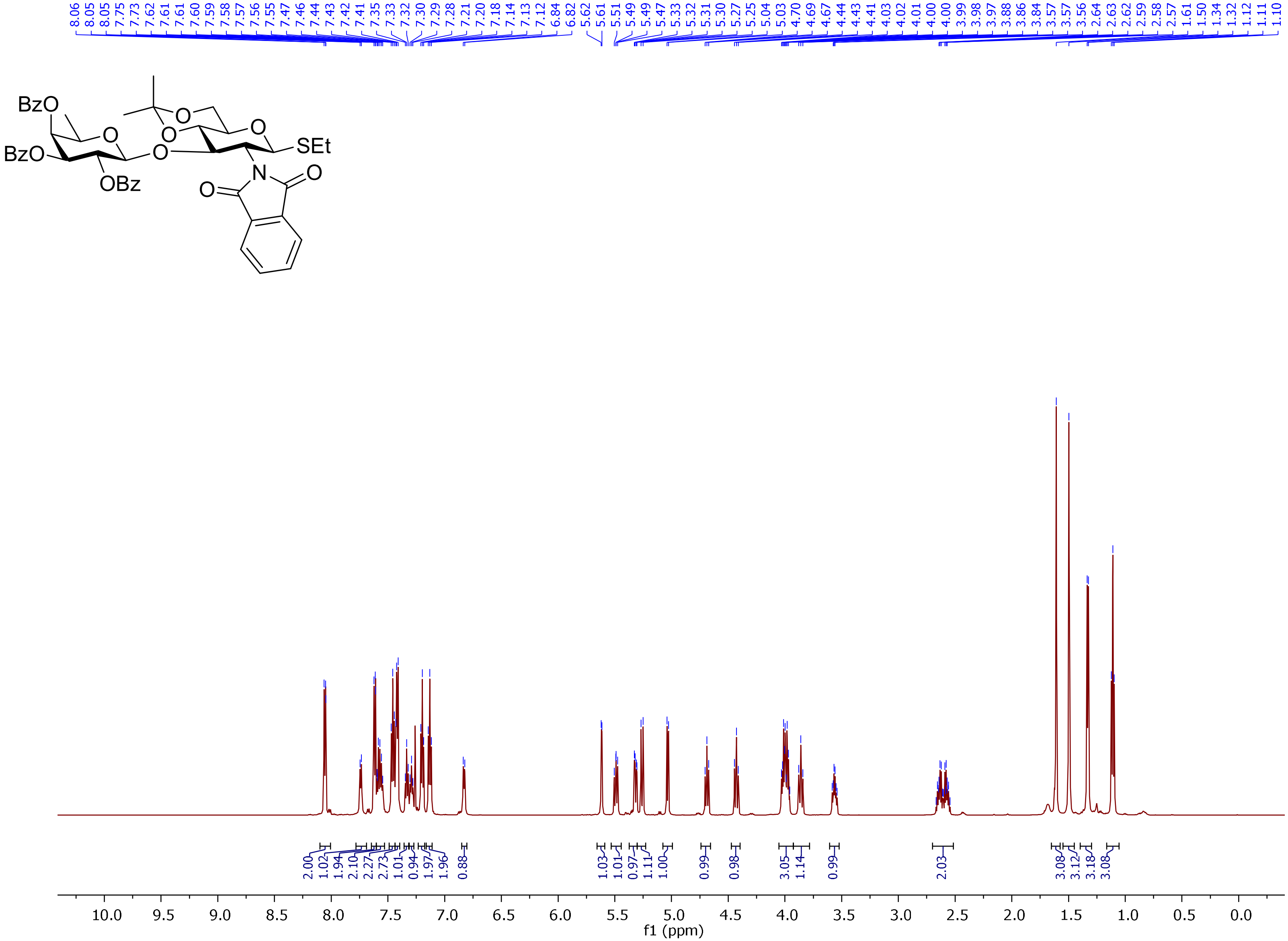

$^{13}C\{^{1}H\}$ NMR (151 MHz, $CDCl_3$) spectrum of ethyl 2-deoxy-2-phthalimido-4,6-*O*-isopropylidene-3-*O*-(2,3,4-tri-*O*-benzoyl-β-D-fucopyranosyl)-1-thio-β-D-glucopyranoside (**21β**)

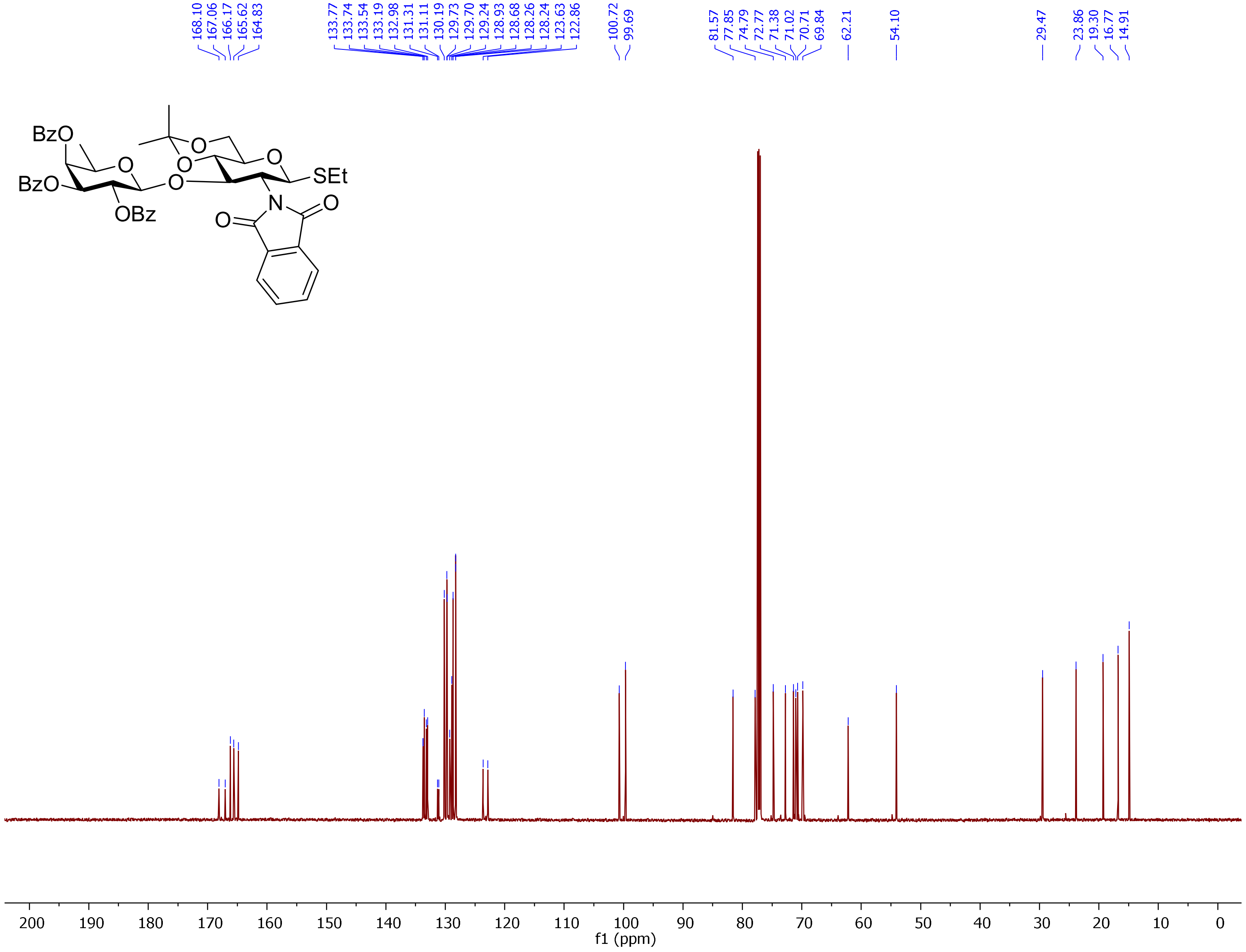

COSY NMR (600 MHz, $CDCl_3$) spectrum of ethyl 2-deoxy-2-phthalimido-4,6-*O*-isopropylidene-3-*O*-(2,3,4-tri-*O*-benzoyl-β-D-fucopyranosyl)-1-thio-β-D-glucopyranoside (**21β**)

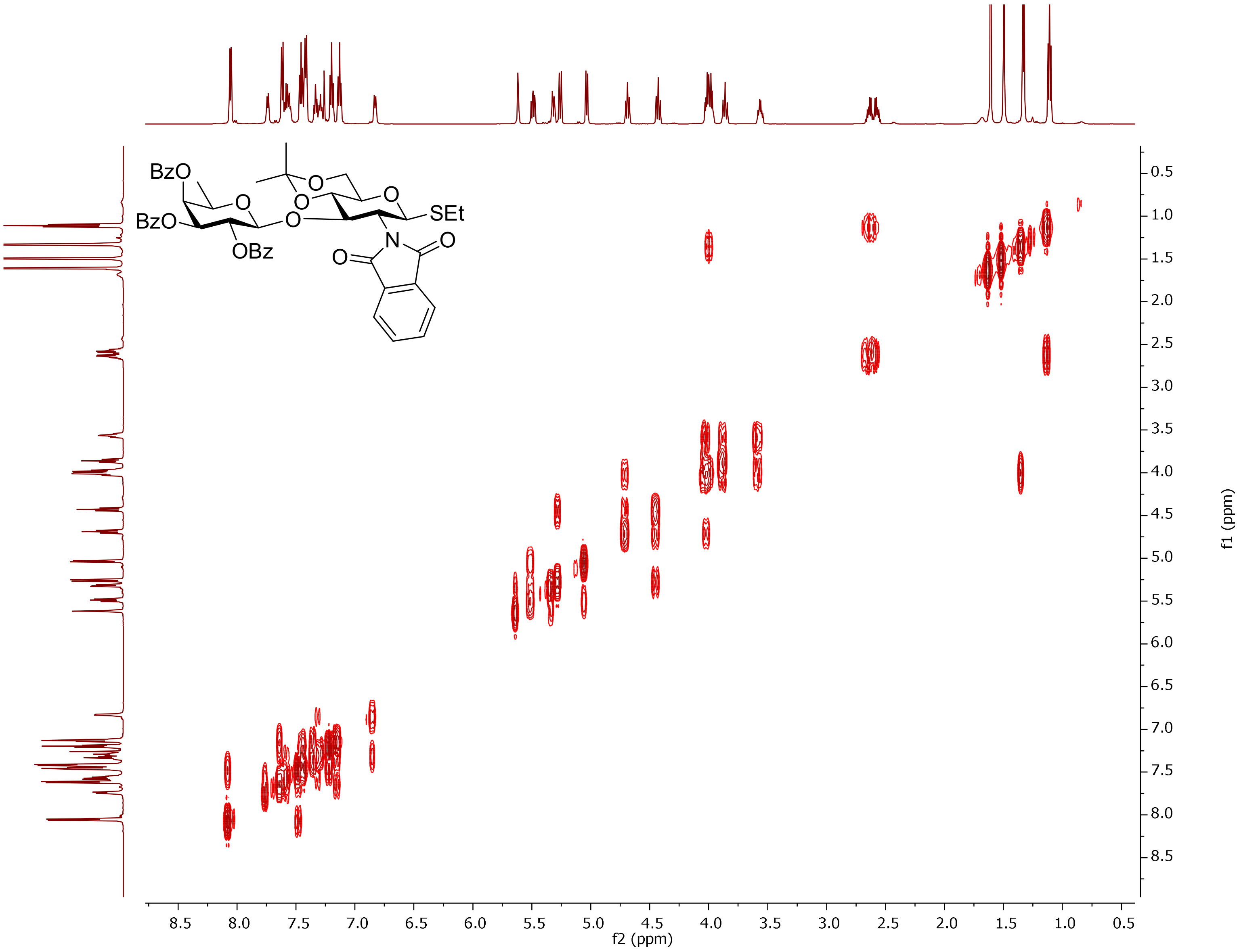

HSQC NMR (600 MHz, $CDCl_3$) spectrum of ethyl 2-deoxy-2-phthalimido-4,6-*O*-isopropylidene-3-*O*-(2,3,4-tri-*O*-benzoyl-β-D-fucopyranosyl)-1-thio-β-D-glucopyranoside (**21β**)

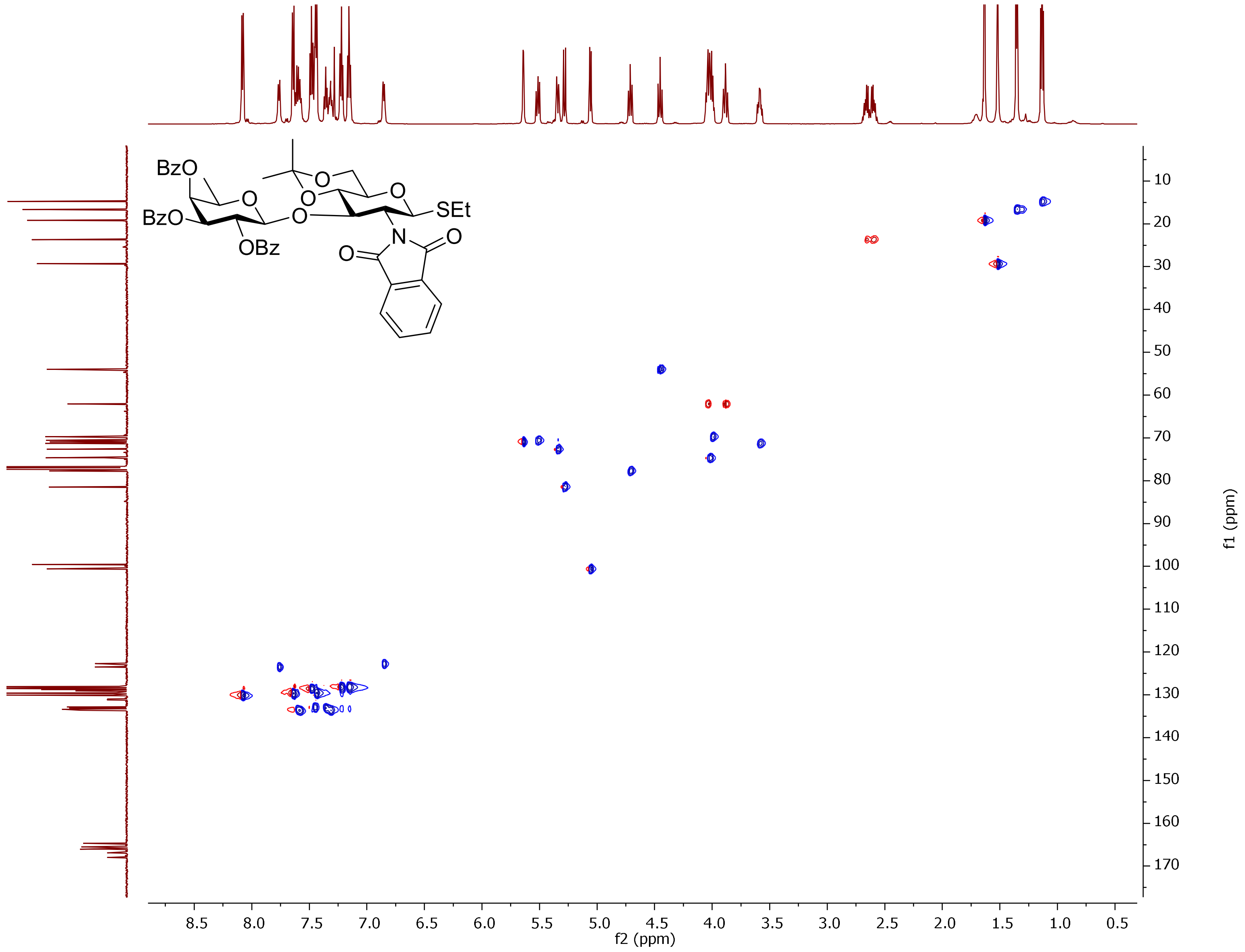

HMBC NMR (600 MHz, $CDCl_3$) spectrum of ethyl 2-deoxy-2-phthalimido-4,6-*O*-isopropylidene-3-*O*-(2,3,4-tri-*O*-benzoyl-β-D-fucopyranosyl)-1-thio-β-D-glucopyranoside (**21β**)

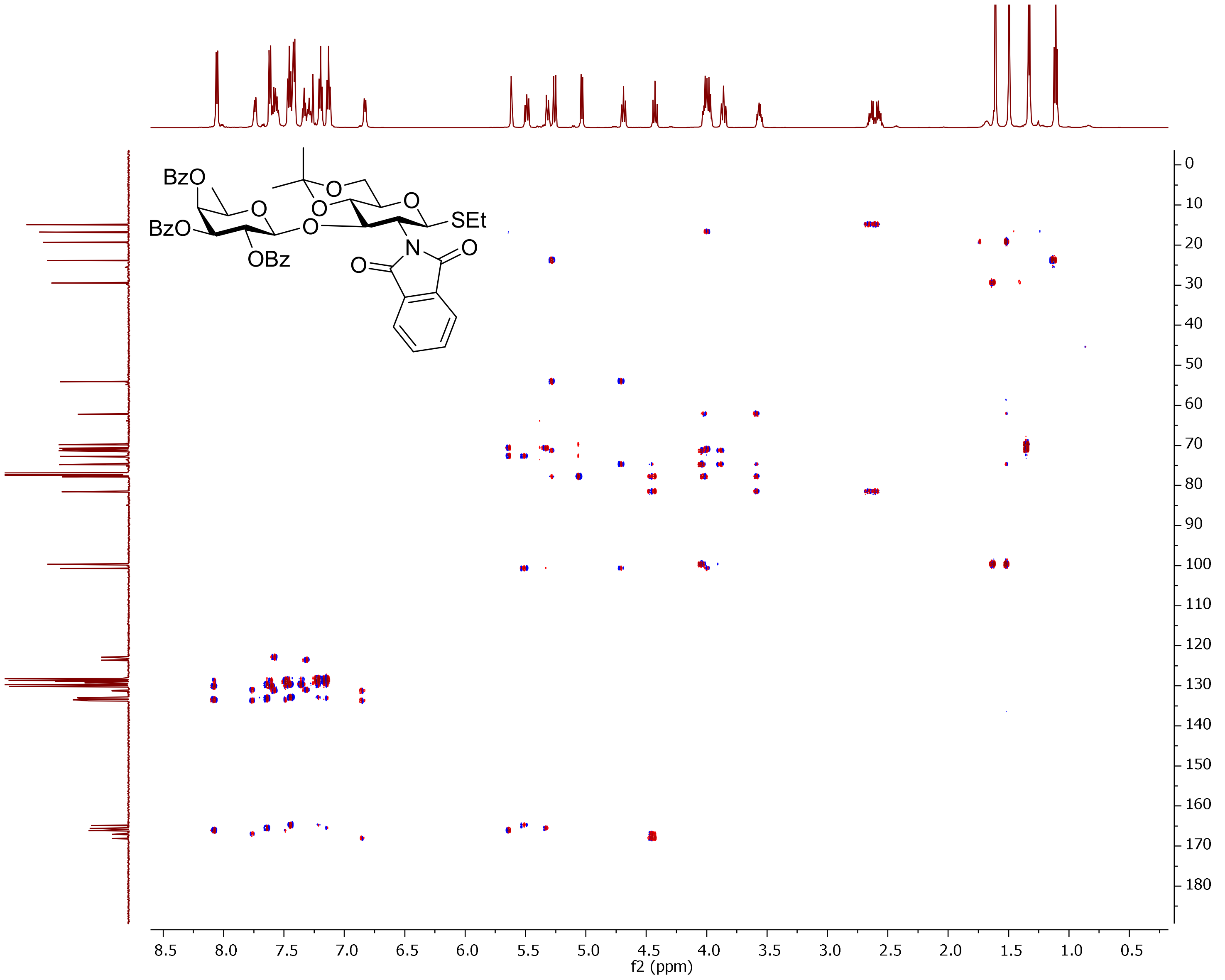

Crude ^{1}H NMR (600 MHz, $CDCl_3$) spectrum of **21** (Donor:Acceptor 1:1, 0.033 M, -25 ºC)

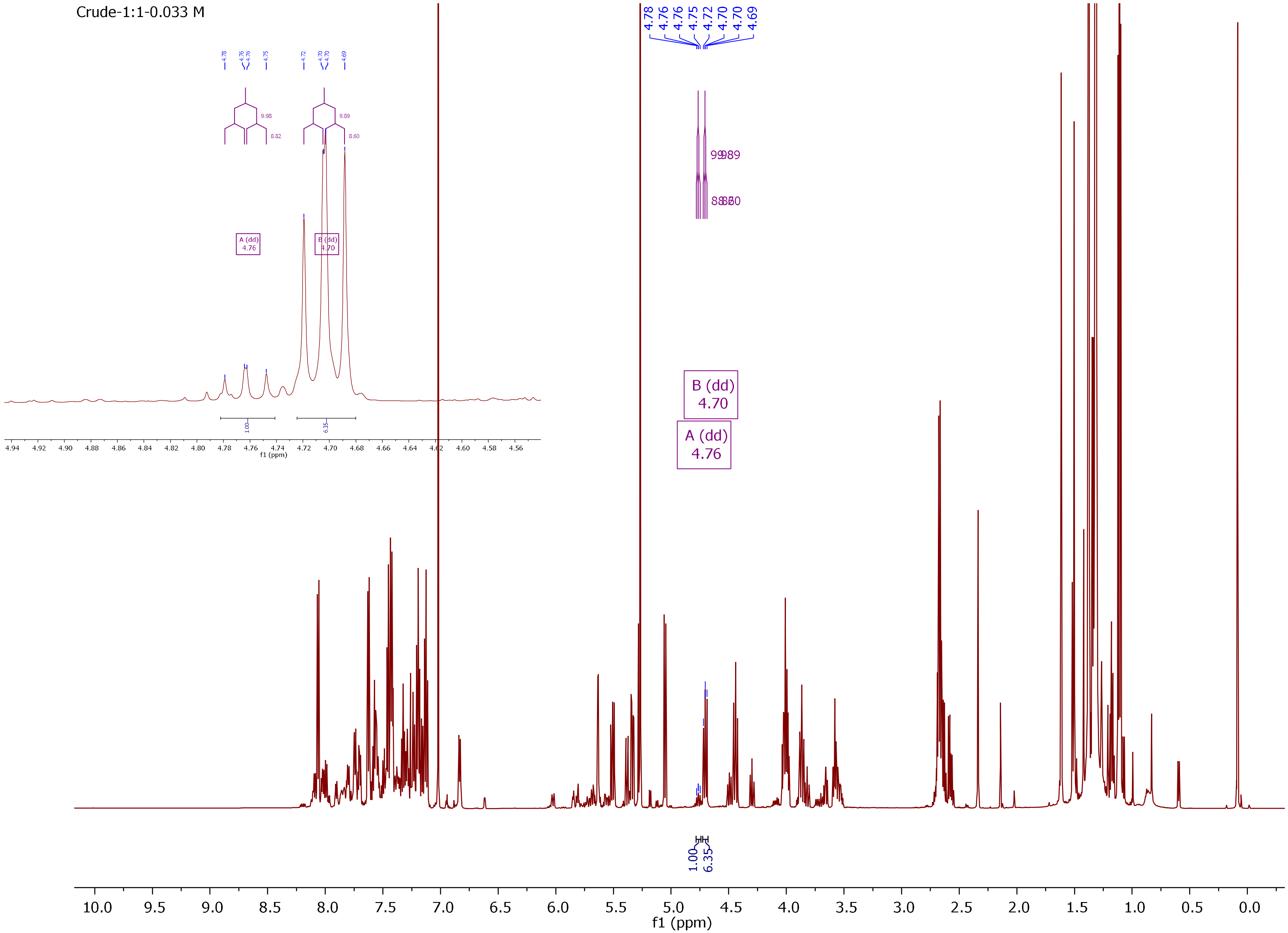

Crude ^{1}H NMR (600 MHz, $CDCl_3$) spectrum of **21** (Donor:Acceptor 1:1, 0.066 M, -25 ºC)

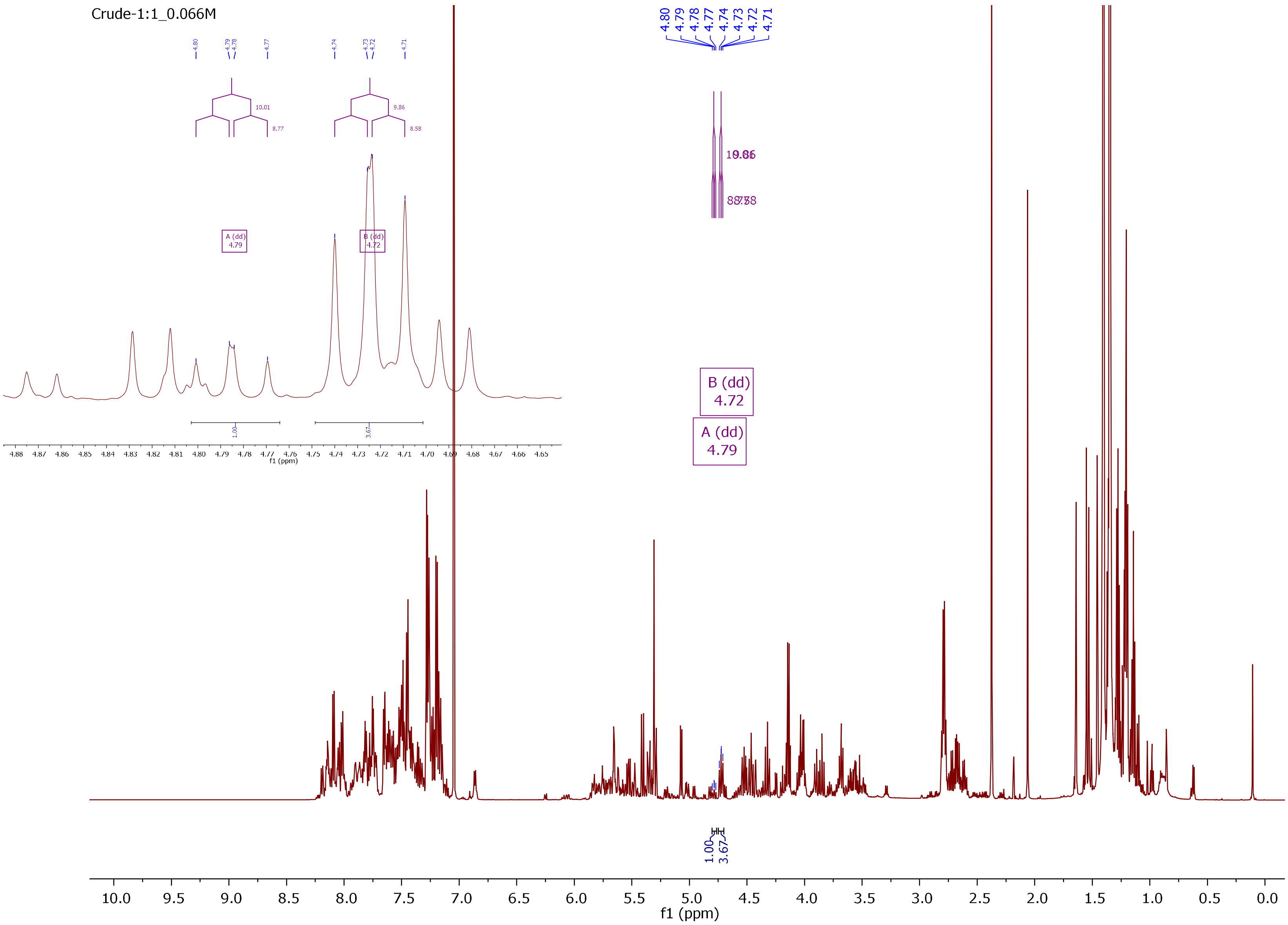

Crude ^{1}H NMR (600 MHz, $CDCl_3$) spectrum of **21** (Donor:Acceptor 1:1, 0.1 M, -25 ºC)

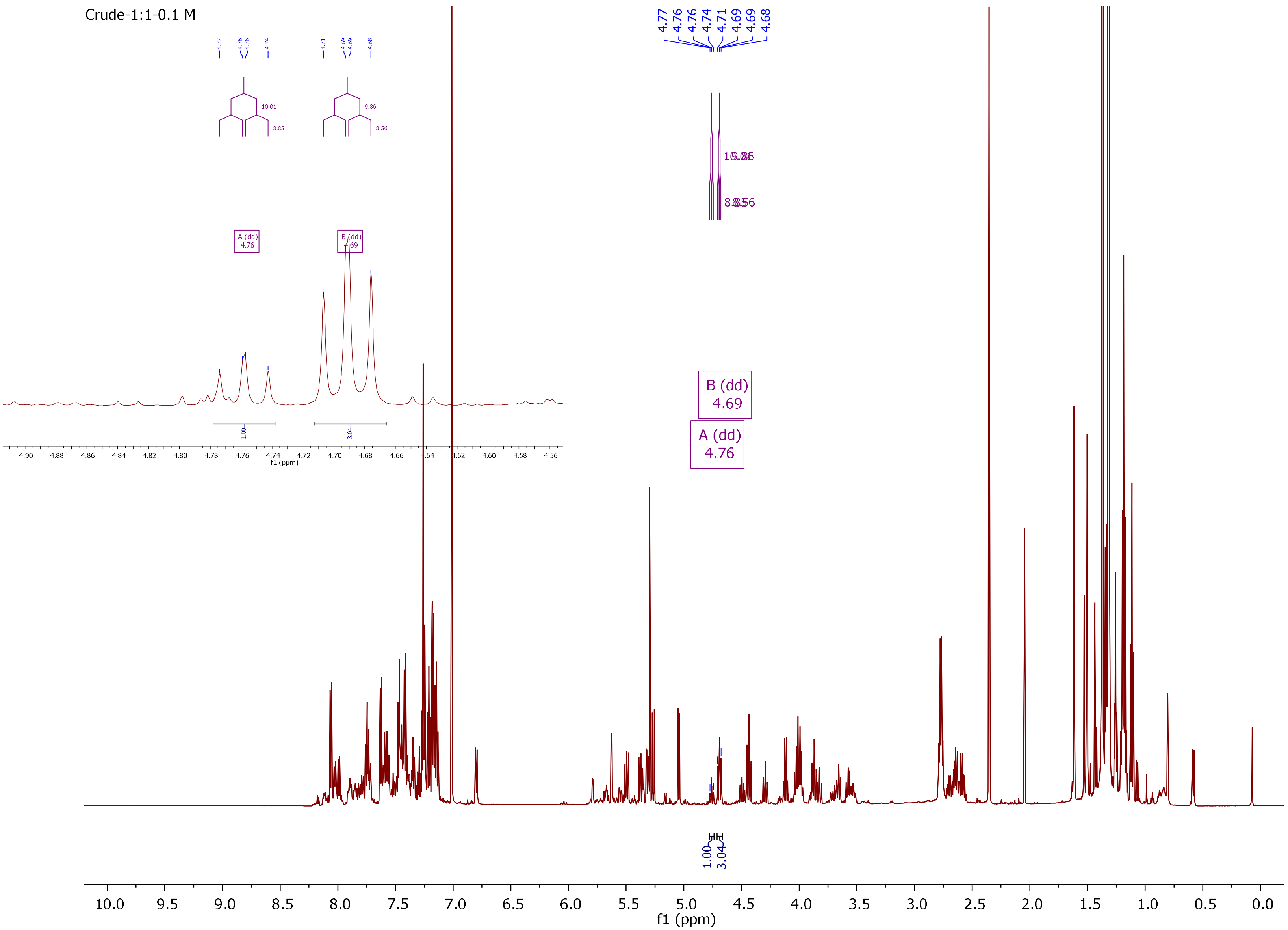

Crude ^{1}H NMR (600 MHz, $CDCl_3$) spectrum of **21** (Donor:Acceptor 1:1, 0.2 M, -25 ºC)

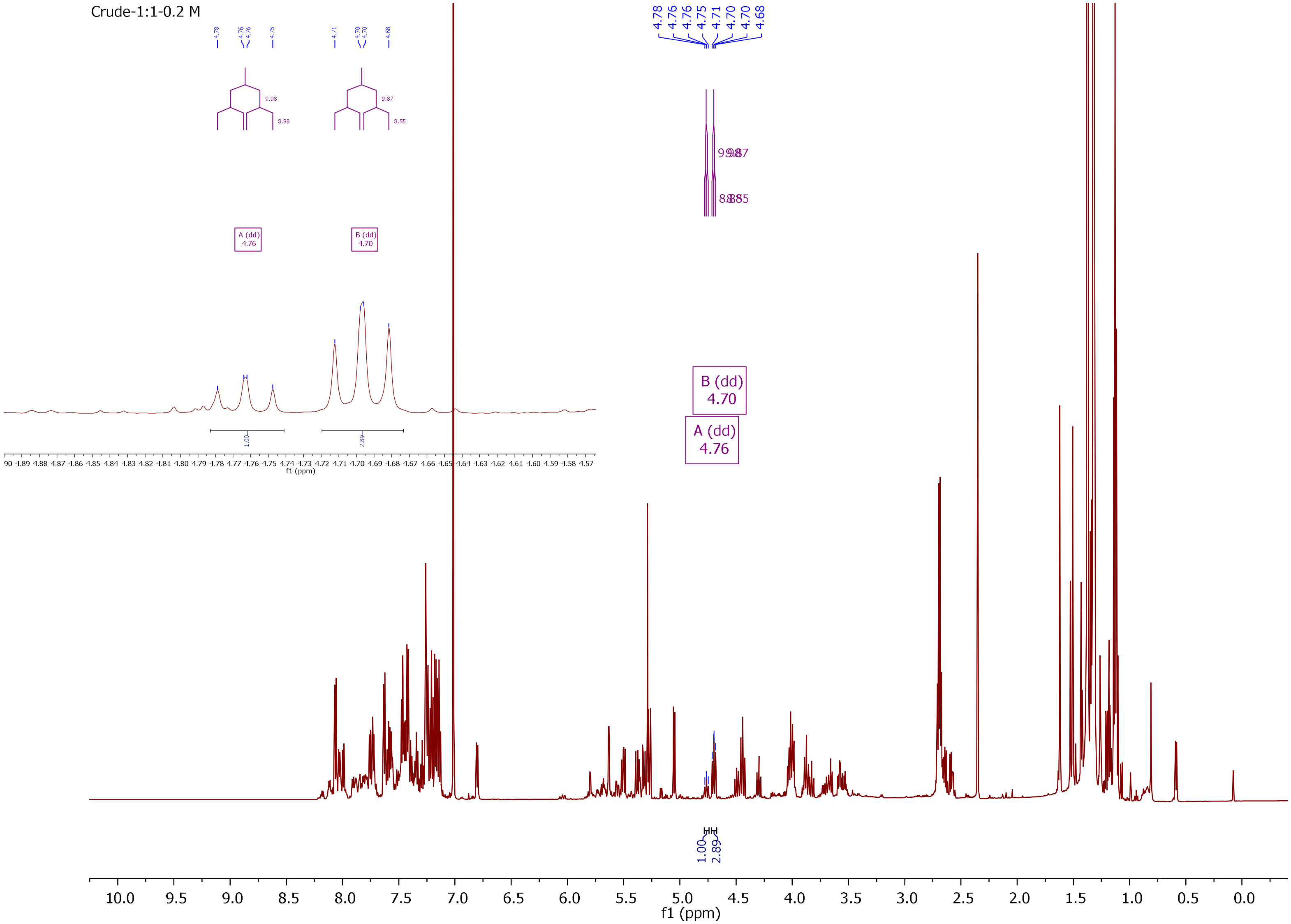

Crude ^{1}H NMR (600 MHz, $CDCl_3$) spectrum of **21** (Donor:Acceptor 1:1, 0.3 M, -25 ºC)

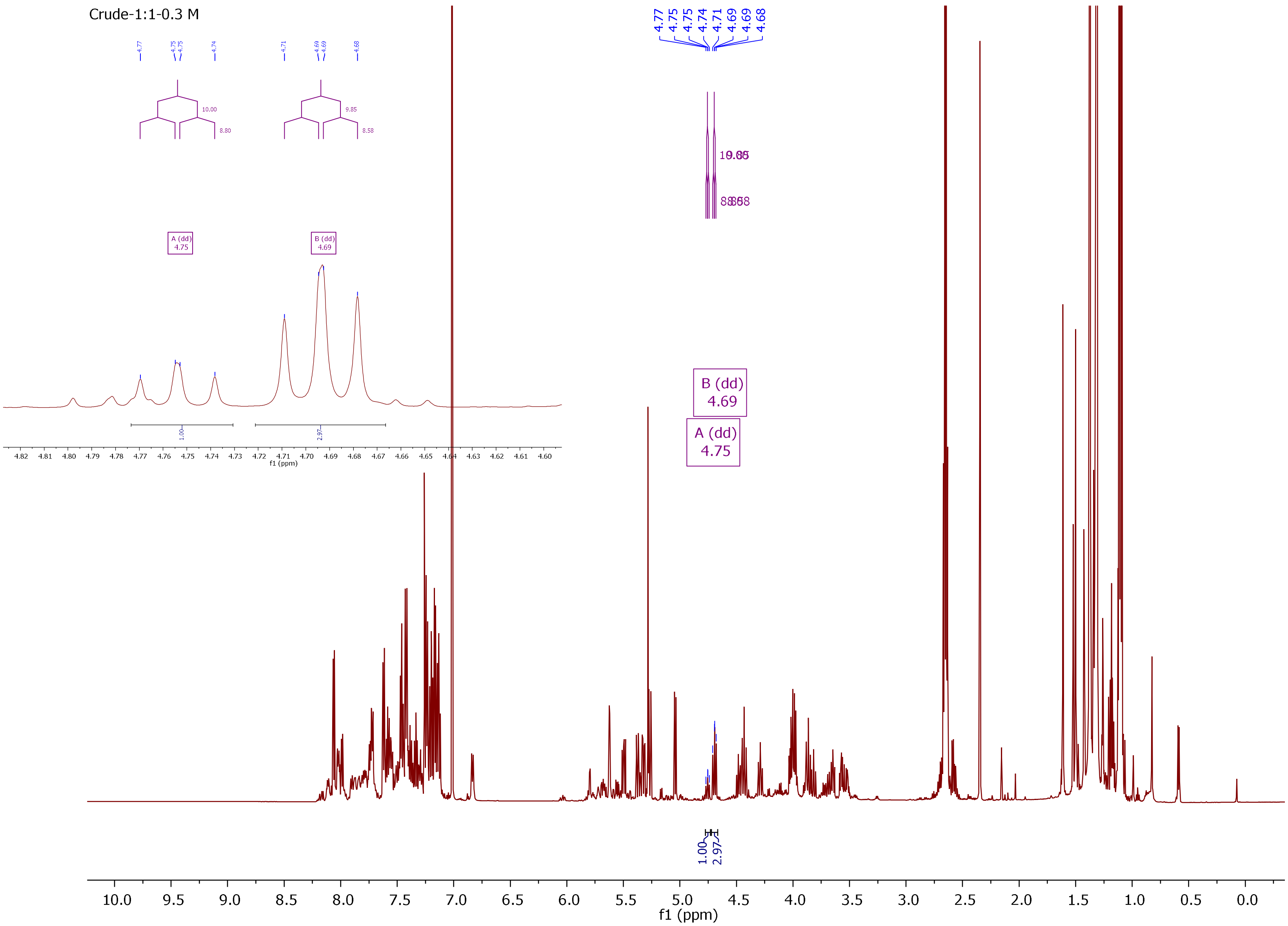

^{1}H NMR (600 MHz, $CDCl_3$) spectrum of ethyl 2-deoxy-2-phthalimido-4,6-*O*-isopropylidene-3-*O*-(2,3,4-tri-*O*-benzoyl-β-L-fucopyranosyl)-1-thio-β-D-glucopyranoside (**22β**)

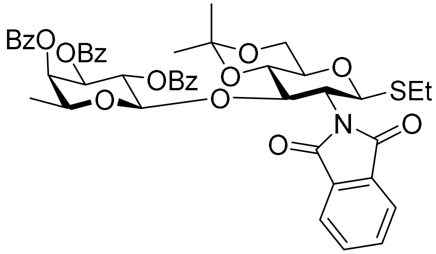

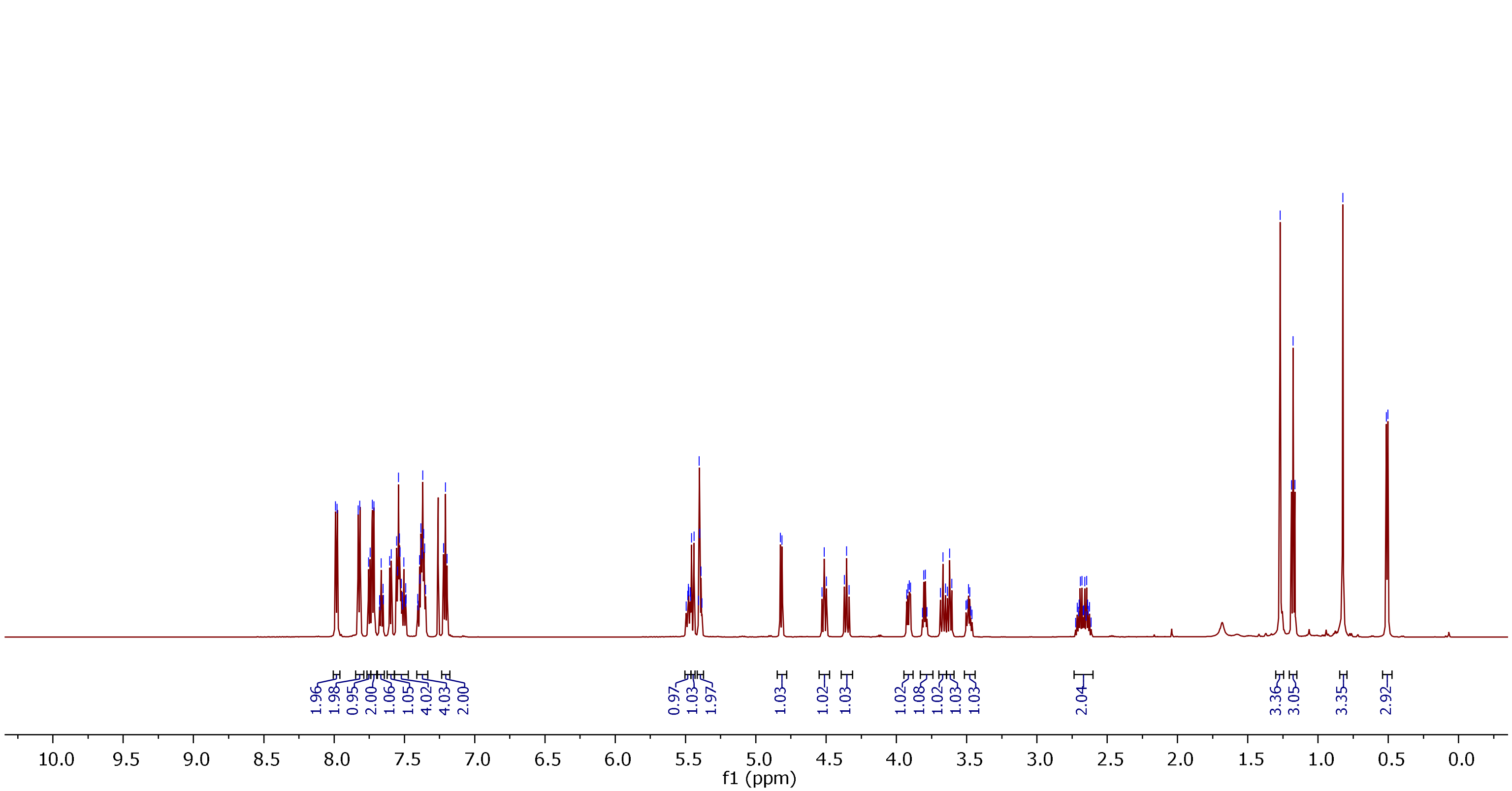

$^{13}C\{^{1}H\}$ NMR (151 MHz, $CDCl_3$) spectrum of ethyl 2-deoxy-2-phthalimido-4,6-*O*-isopropylidene-3-*O*-(2,3,4-tri-*O*-benzoyl-β-L-fucopyranosyl)-1-thio-β-D-glucopyranoside (**22β**)

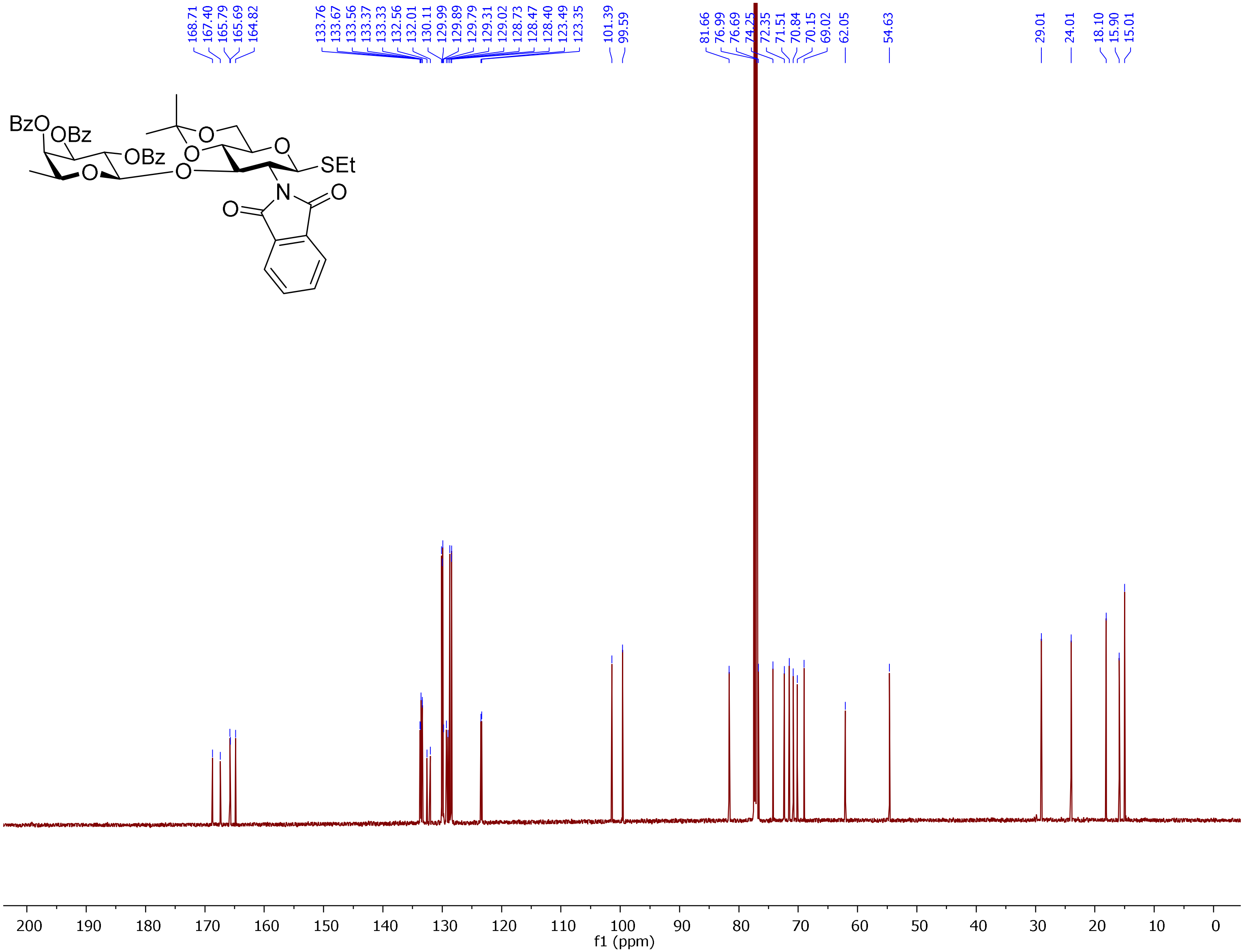

COSY NMR (600 MHz, $CDCl_3$) spectrum of ethyl 2-deoxy-2-phthalimido-4,6-*O*-isopropylidene-3-*O*-(2,3,4-tri-*O*-benzoyl-β-L-fucopyranosyl)-1-thio-β-D-glucopyranoside (**22β**)

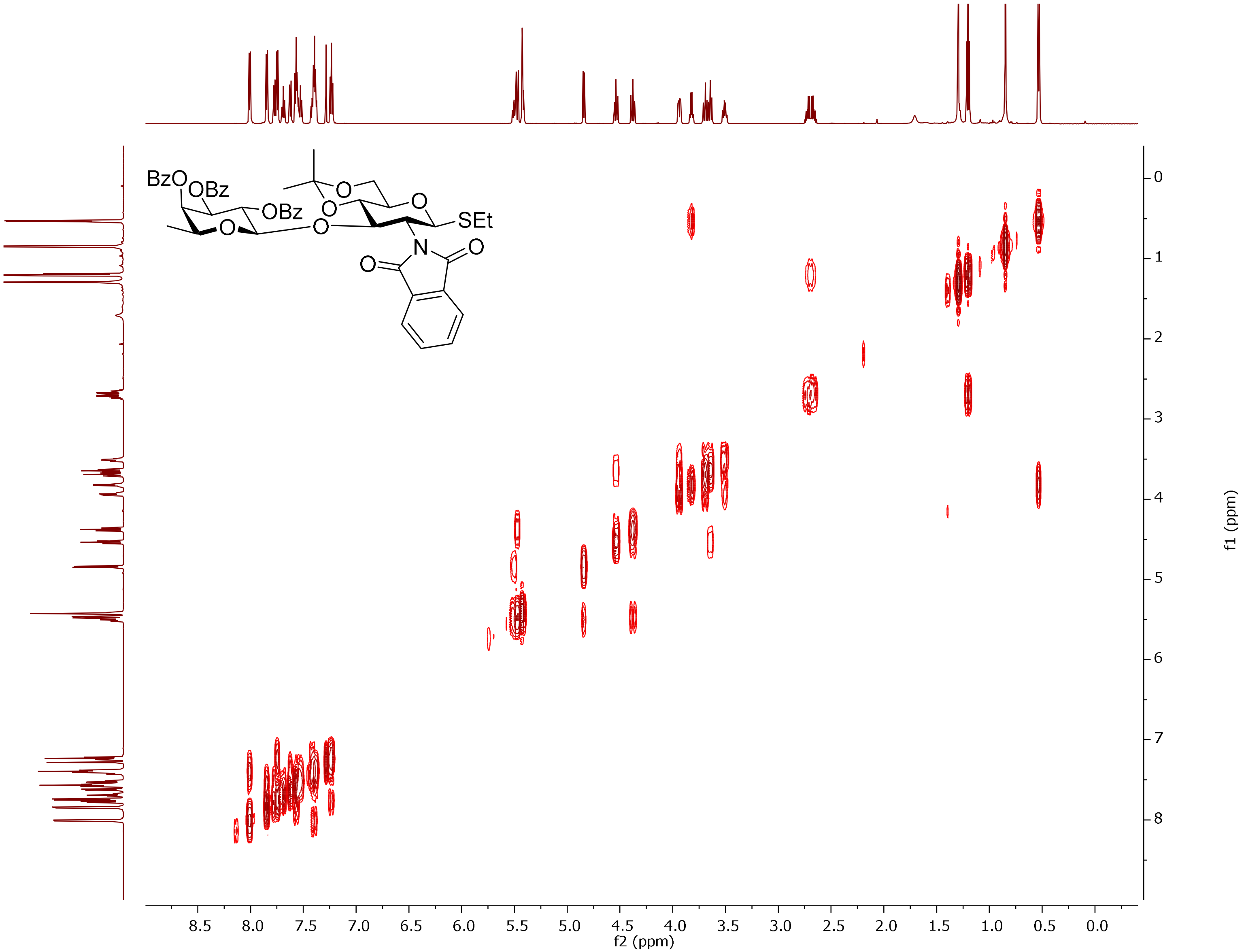

HSQC NMR (600 MHz, $CDCl_3$) spectrum of ethyl 2-deoxy-2-phthalimido-4,6-*O*-isopropylidene-3-*O*-(2,3,4-tri-*O*-benzoyl-β-L-fucopyranosyl)-1-thio-β-D-glucopyranoside (**22β**)

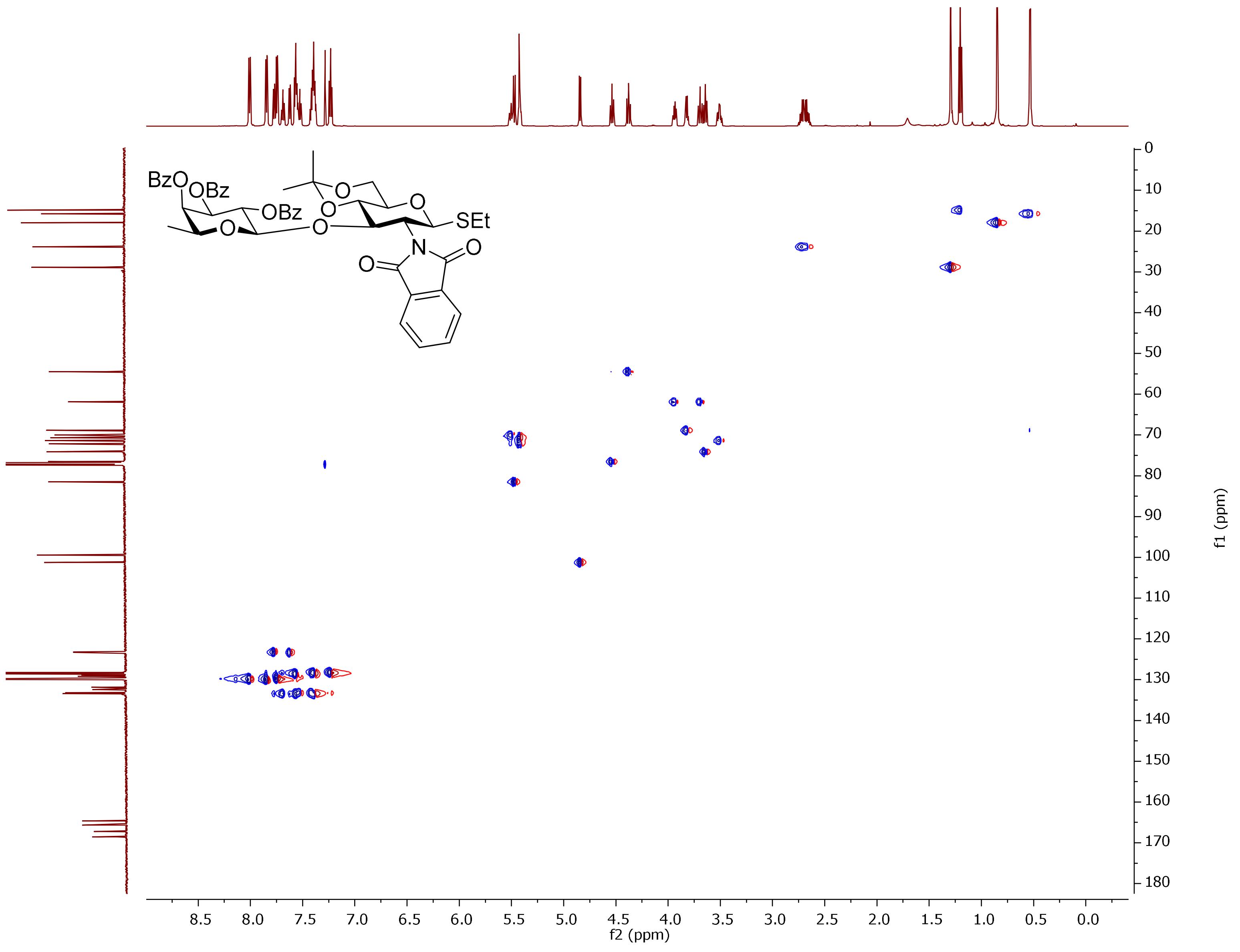

HMBC NMR (600 MHz, $CDCl_3$) spectrum of ethyl 2-deoxy-2-phthalimido-4,6-*O*-isopropylidene-3-*O*-(2,3,4-tri-*O*-benzoyl-β-L-fucopyranosyl)-1-thio-β-D-glucopyranoside (**22β**)

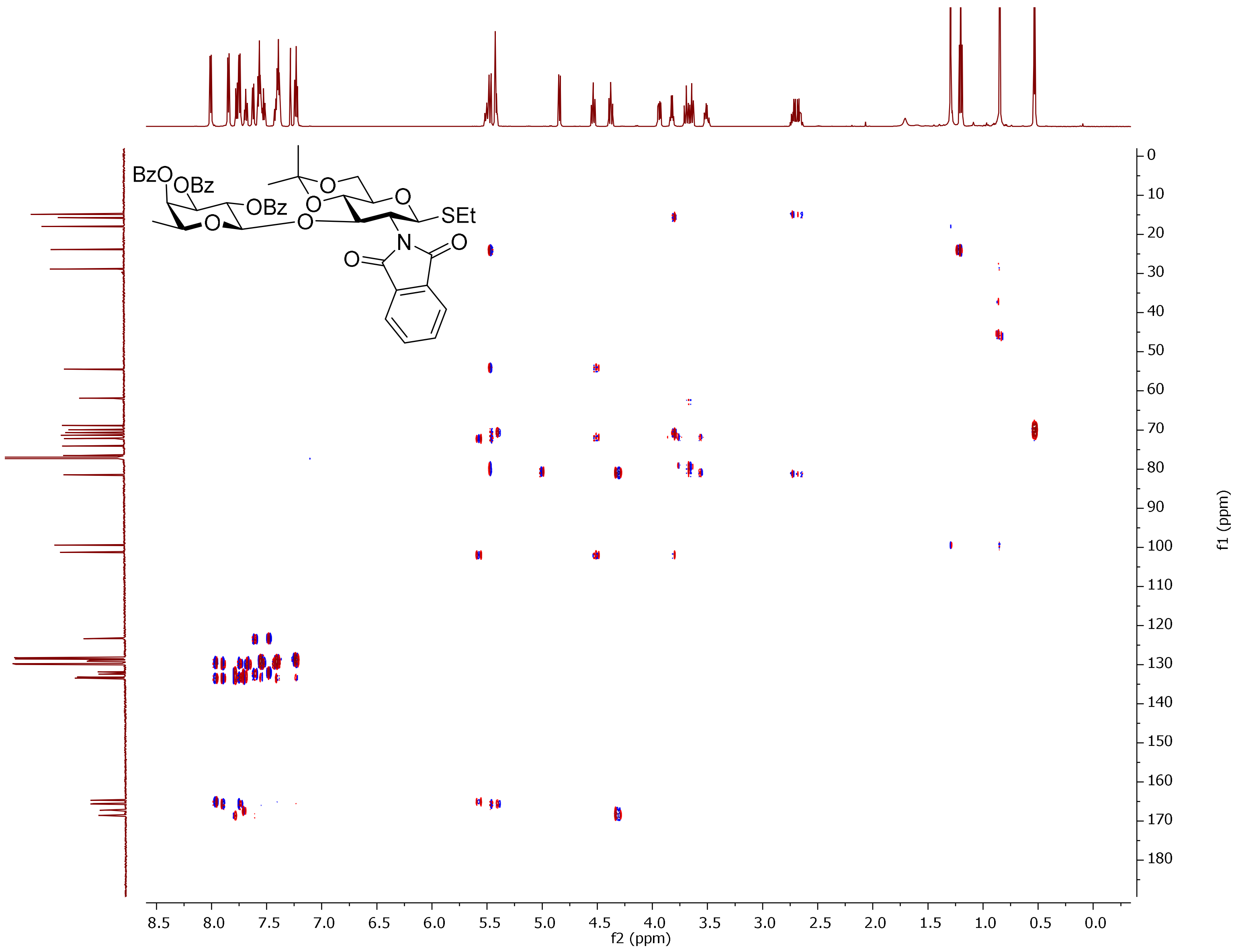

Crude ^{1}H NMR (600 MHz, $CDCl_3$) spectrum of **22** (Donor:Acceptor 1:1, 0.033 M, -25 ºC)

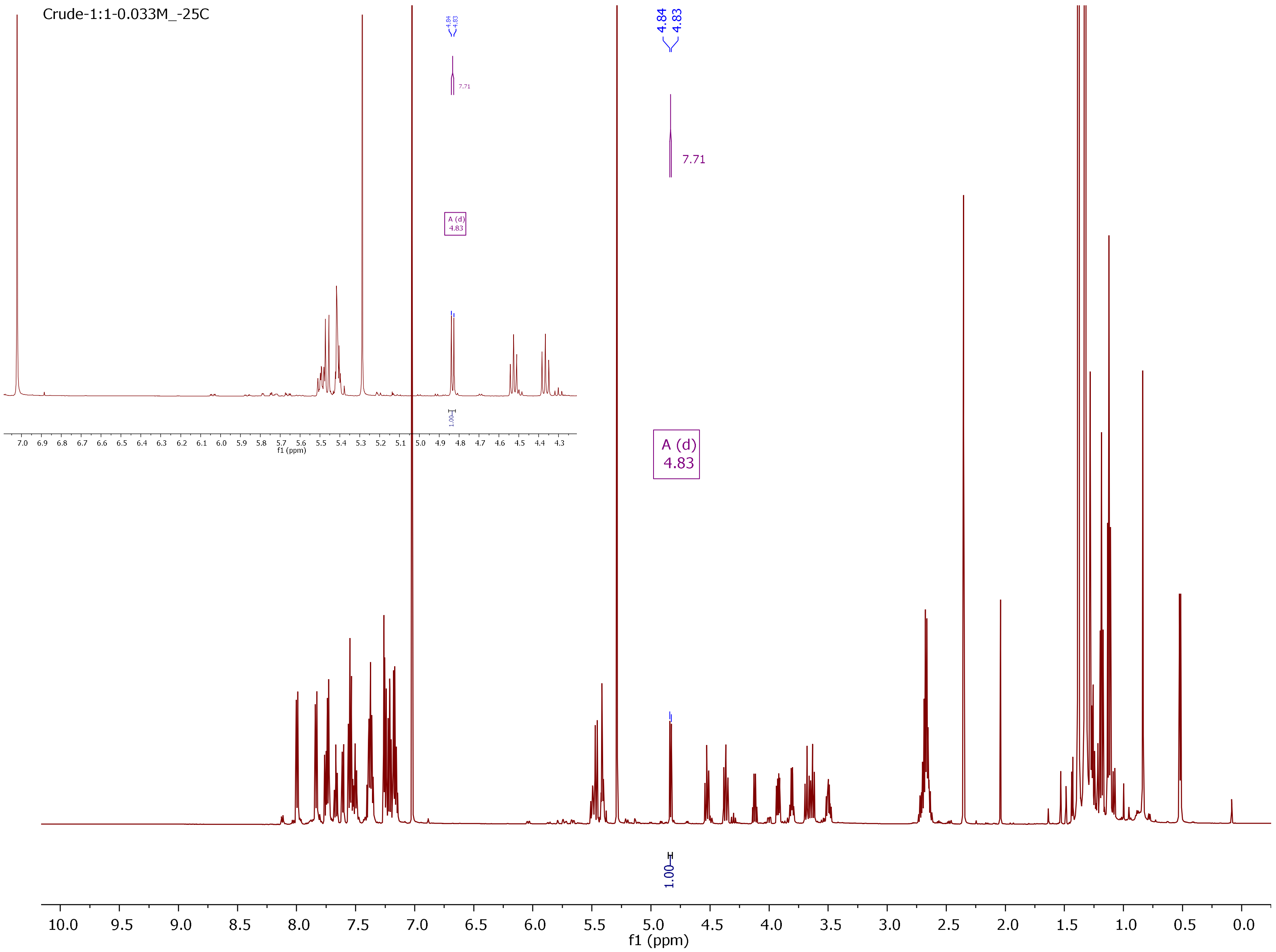

Crude ^{1}H NMR (600 MHz, $CDCl_3$) spectrum of **22** (Donor:Acceptor 1:1, 0.2 M, -25 ºC)

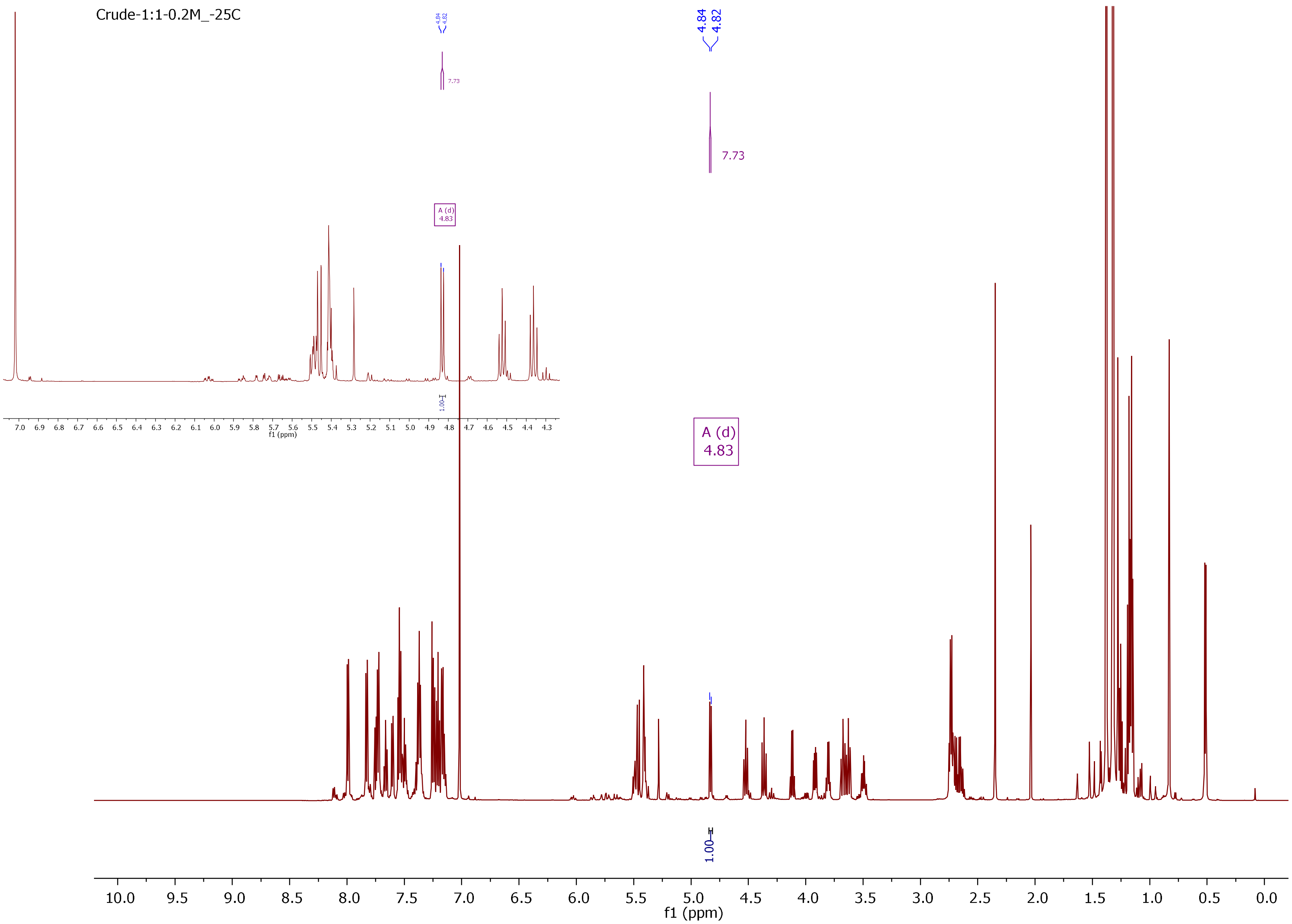

Crude ^{1}H NMR (600 MHz, $CDCl_3$) spectrum of **22** (Donor:Acceptor 1:1, 0.033 M, -45 ºC)

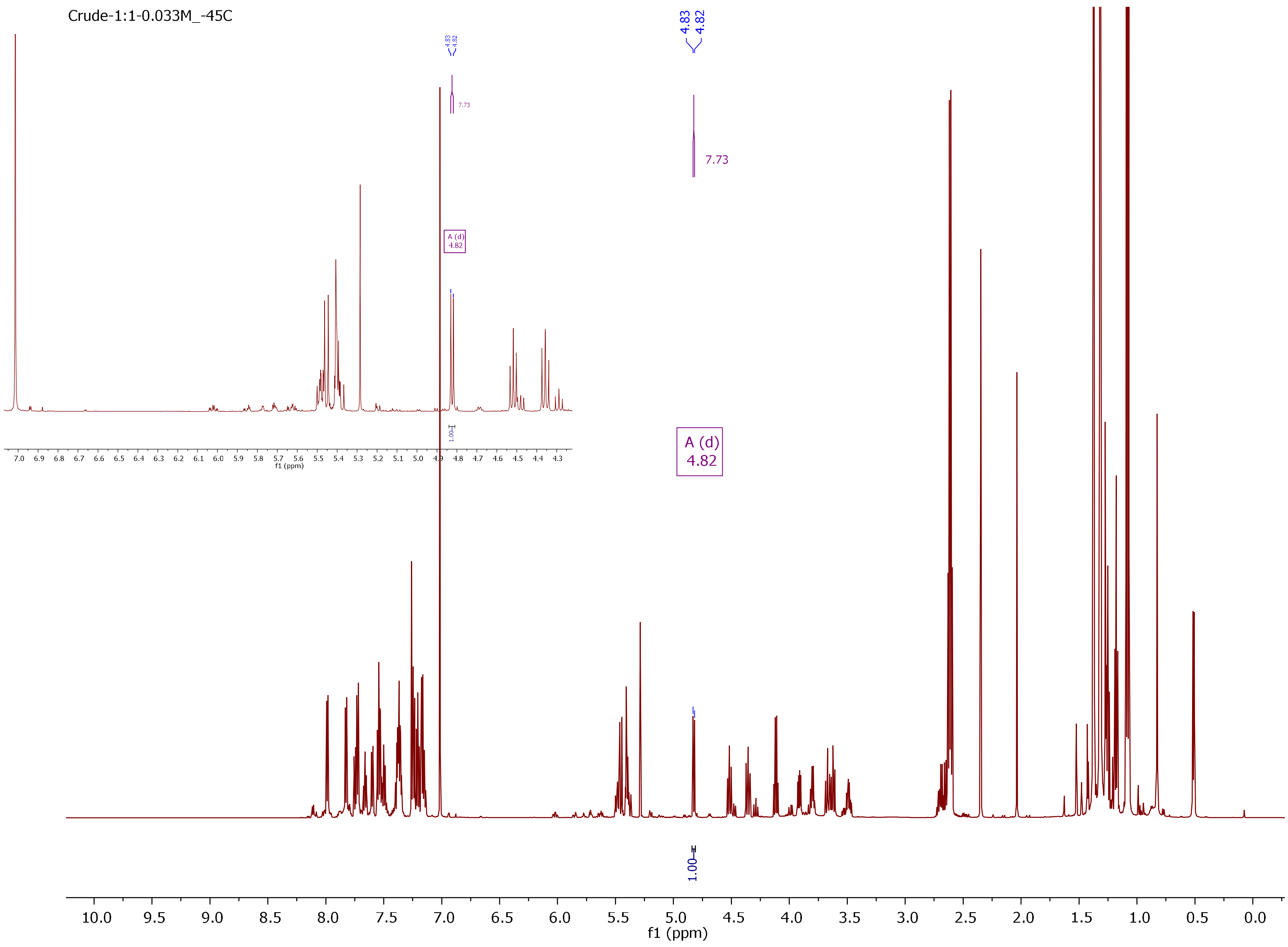

Crude ^{1}H NMR (600 MHz, $CDCl_3$) spectrum of **22** (Donor:Acceptor 1:1, 0.2 M, -45 ºC)

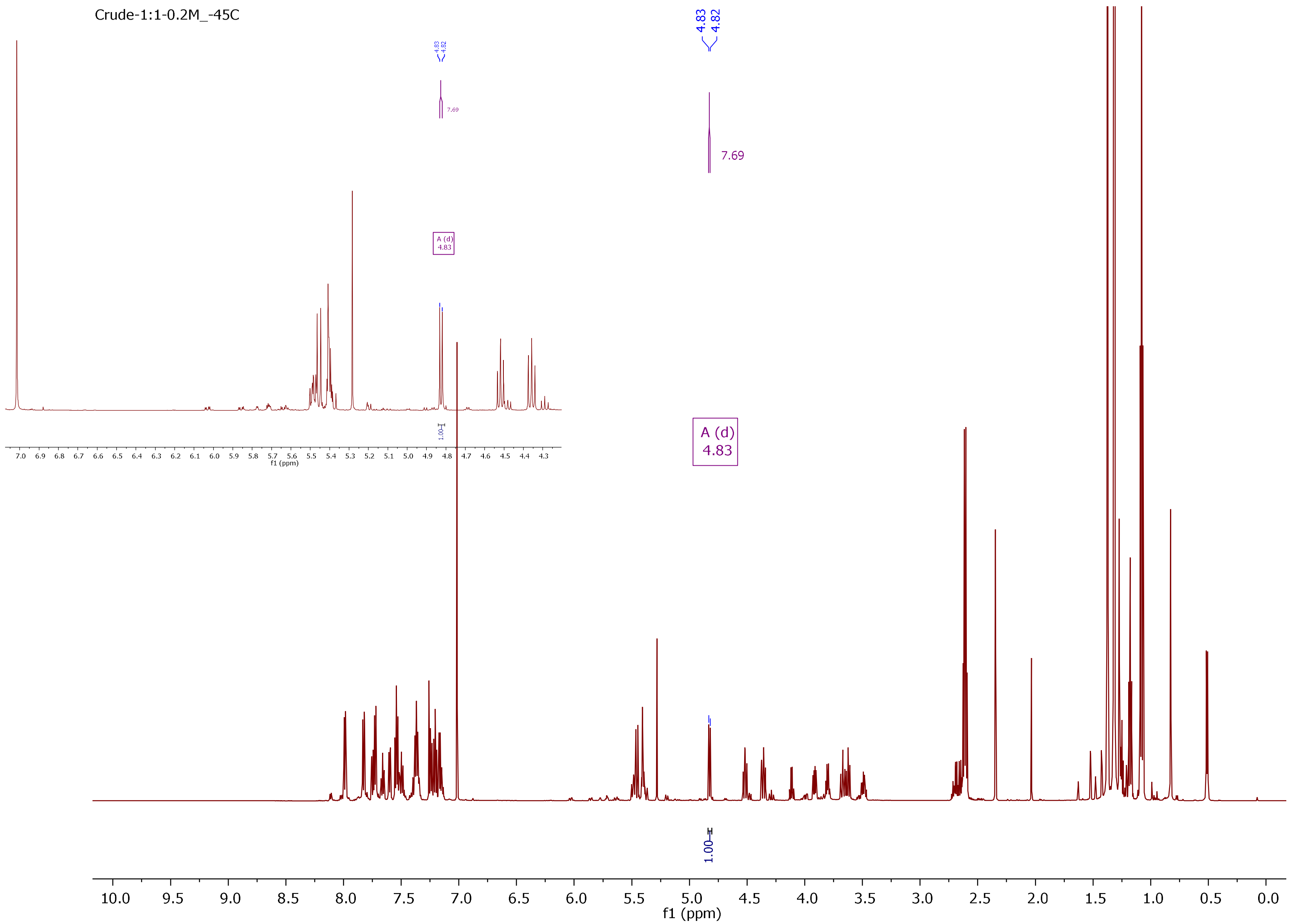

^{1}H NMR (600 MHz, $CDCl_3$) spectrum of ethyl 2-deoxy-2-phthalimido-4,6-*O*-isopropylidene-3-*O*-(2,3,4-tri-*O*-acetyl-α-D-fucopyranosyl)-1-thio-β-D-glucopyranoside (**25α**)

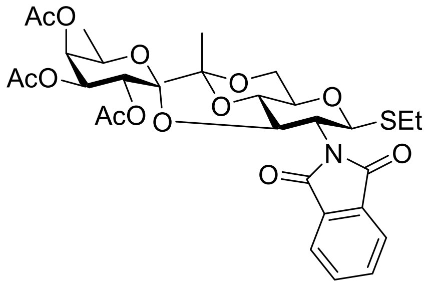

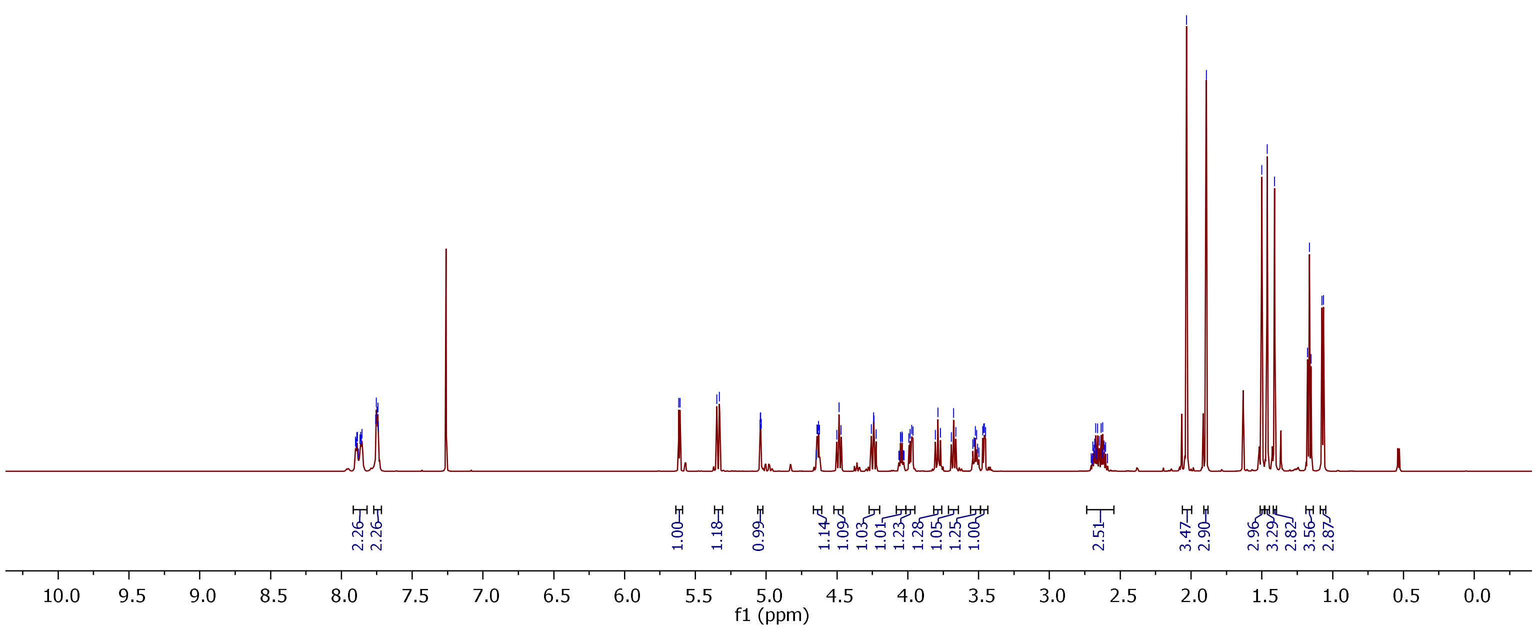

$^{13}C\{^{1}H\}$ NMR (151 MHz, $CDCl_3$) spectrum of ethyl 2-deoxy-2-phthalimido-4,6-*O*-isopropylidene-3-*O*-(2,3,4-tri-*O*-acetyl-α-D-fucopyranosyl)-1-thio-β-D-glucopyranoside (**25α**)

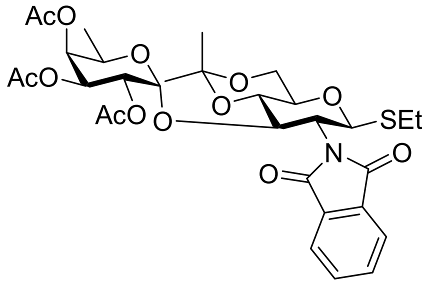

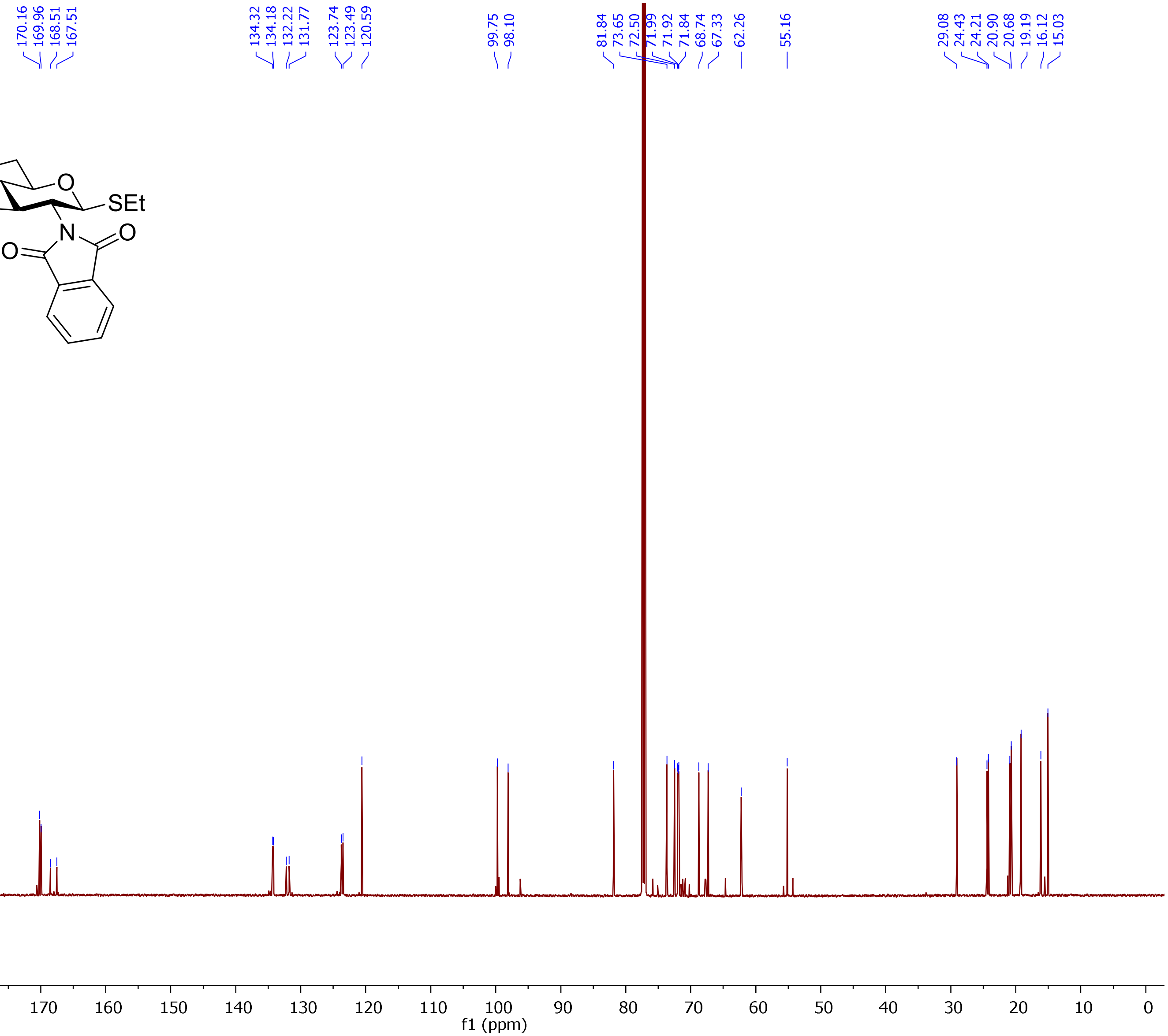

COSY NMR (600 MHz, $CDCl_3$) spectrum of ethyl 2-deoxy-2-phthalimido-4,6-*O*-isopropylidene-3-*O*-(2,3,4-tri-*O*-acetyl-α-D-fucopyranosyl)-1-thio-β-D-glucopyranoside (**25α**)

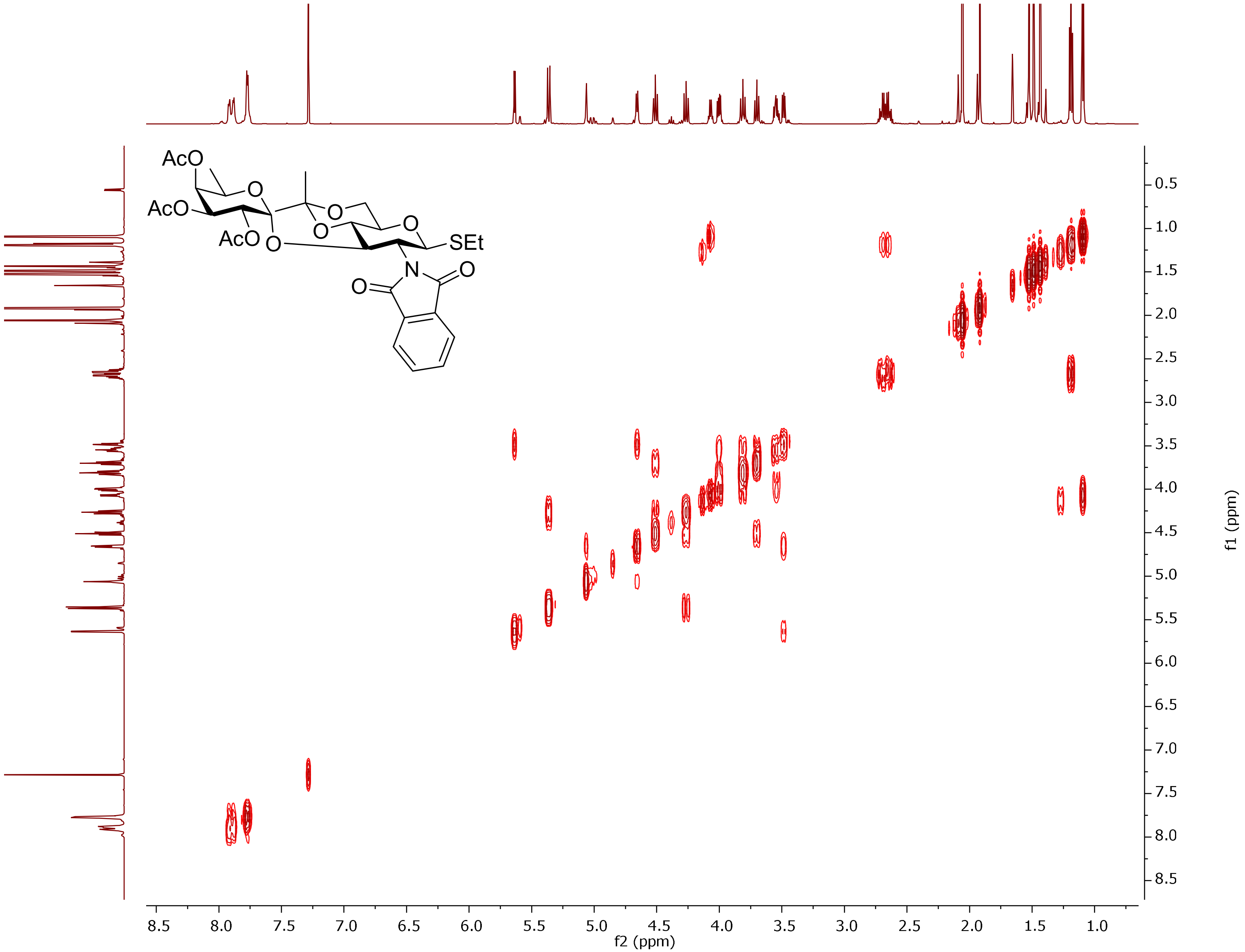

HSQC NMR (600 MHz, $CDCl_3$) spectrum of ethyl 2-deoxy-2-phthalimido-4,6-*O*-isopropylidene-3-*O*-(2,3,4-tri-*O*-acetyl-α-D-fucopyranosyl)-1-thio-β-D-glucopyranoside (**25α**)

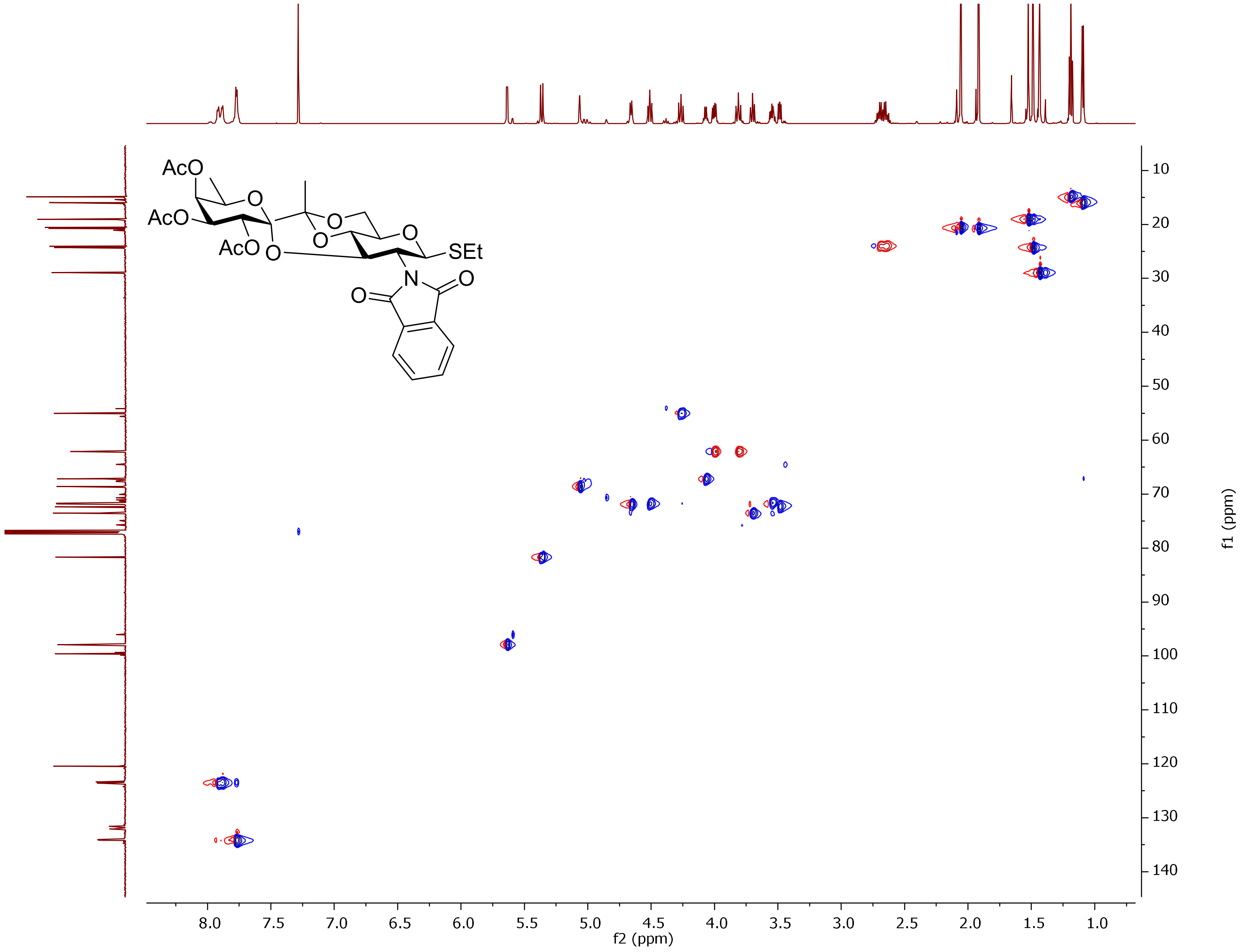

HMBC NMR (600 MHz, $CDCl_3$) spectrum of ethyl 2-deoxy-2-phthalimido-4,6-*O*-isopropylidene-3-*O*-(2,3,4-tri-*O*-acetyl-α-D-fucopyranosyl)-1-thio-β-D-glucopyranoside (**25α**)

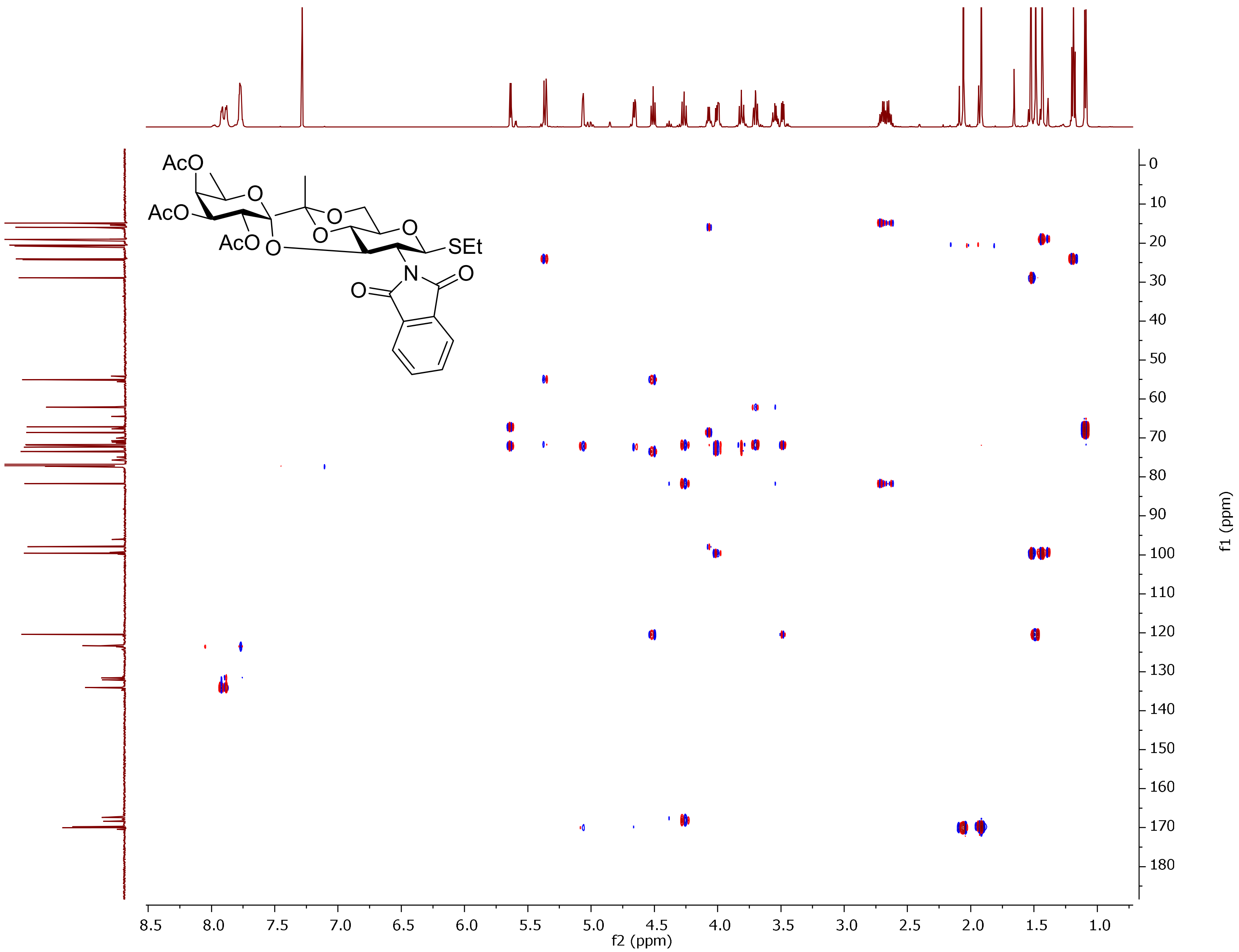

^{1}H NMR (900 MHz, $CDCl_3$) spectrum of ethyl 2-deoxy-2-phthalimido-4,6-*O*-isopropylidene-3-*O*-(2,3,4-tri-*O*-acetyl-β-D-fucopyranosyl)-1-thio-β-D-glucopyranoside (**25β**)

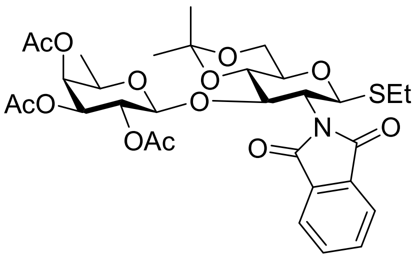

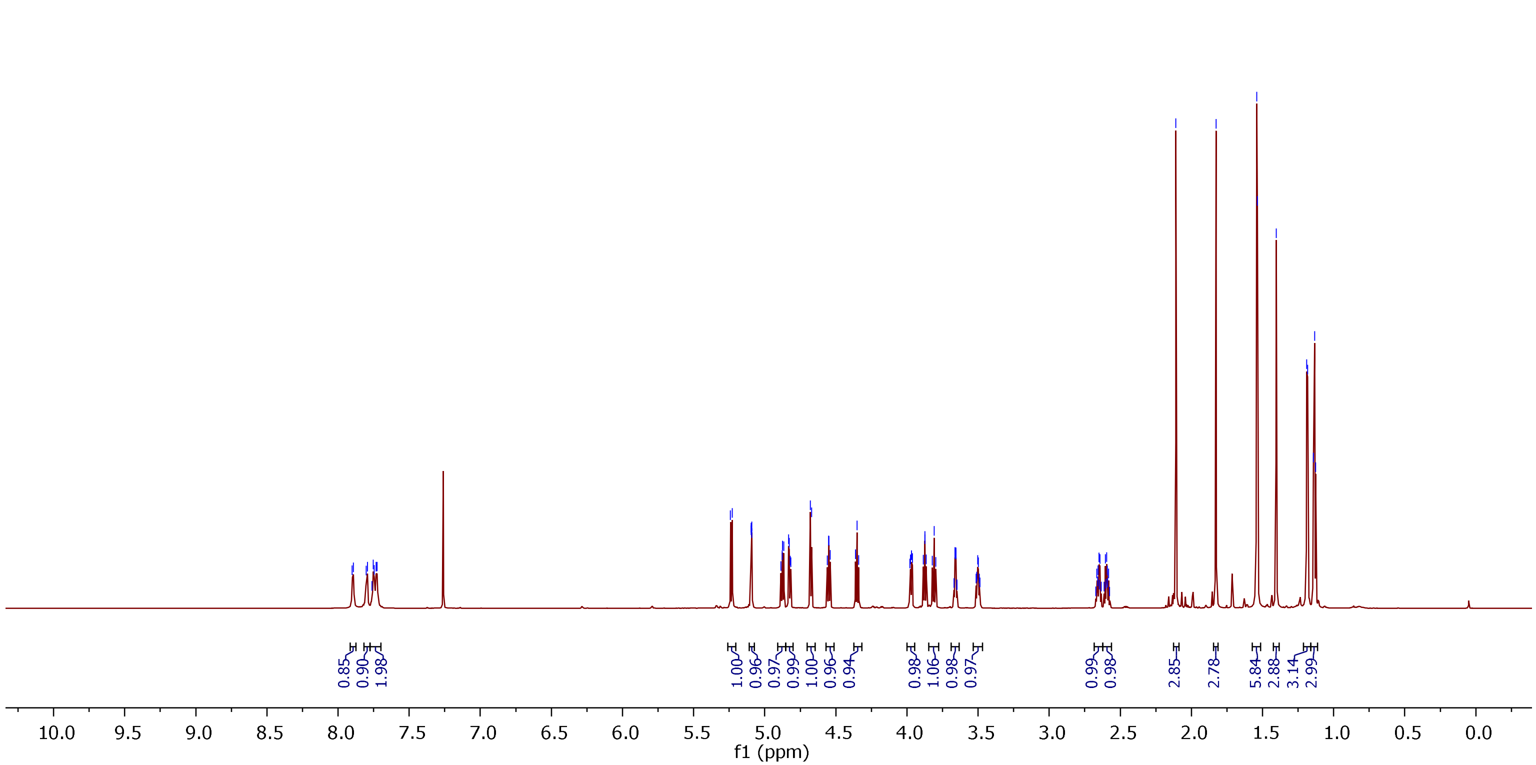

$^{13}C\{^{1}H\}$ NMR (226 MHz, $CDCl_3$) spectrum of ethyl 2-deoxy-2-phthalimido-4,6-*O*-isopropylidene-3-*O*-(2,3,4-tri-*O*-acetyl-β-D-fucopyranosyl)-1-thio-β-D-glucopyranoside (**25β**)

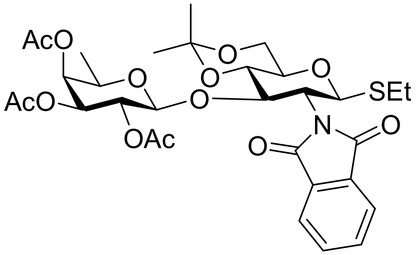

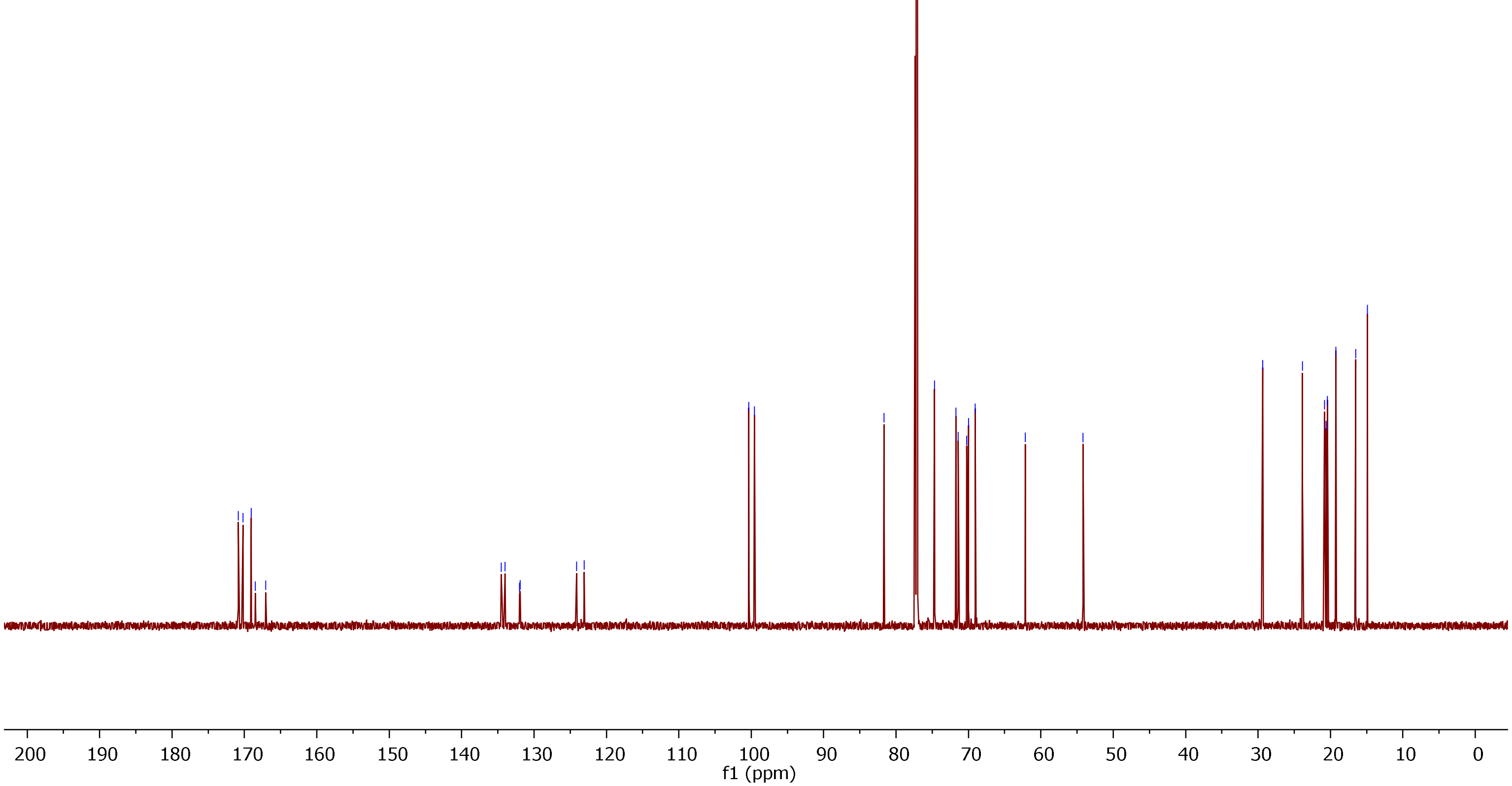

COSY NMR (900 MHz, $CDCl_3$) spectrum of ethyl 2-deoxy-2-phthalimido-4,6-*O*-isopropylidene-3-*O*-(2,3,4-tri-*O*-acetyl-β-D-fucopyranosyl)-1-thio-β-D-glucopyranoside (**25β**)

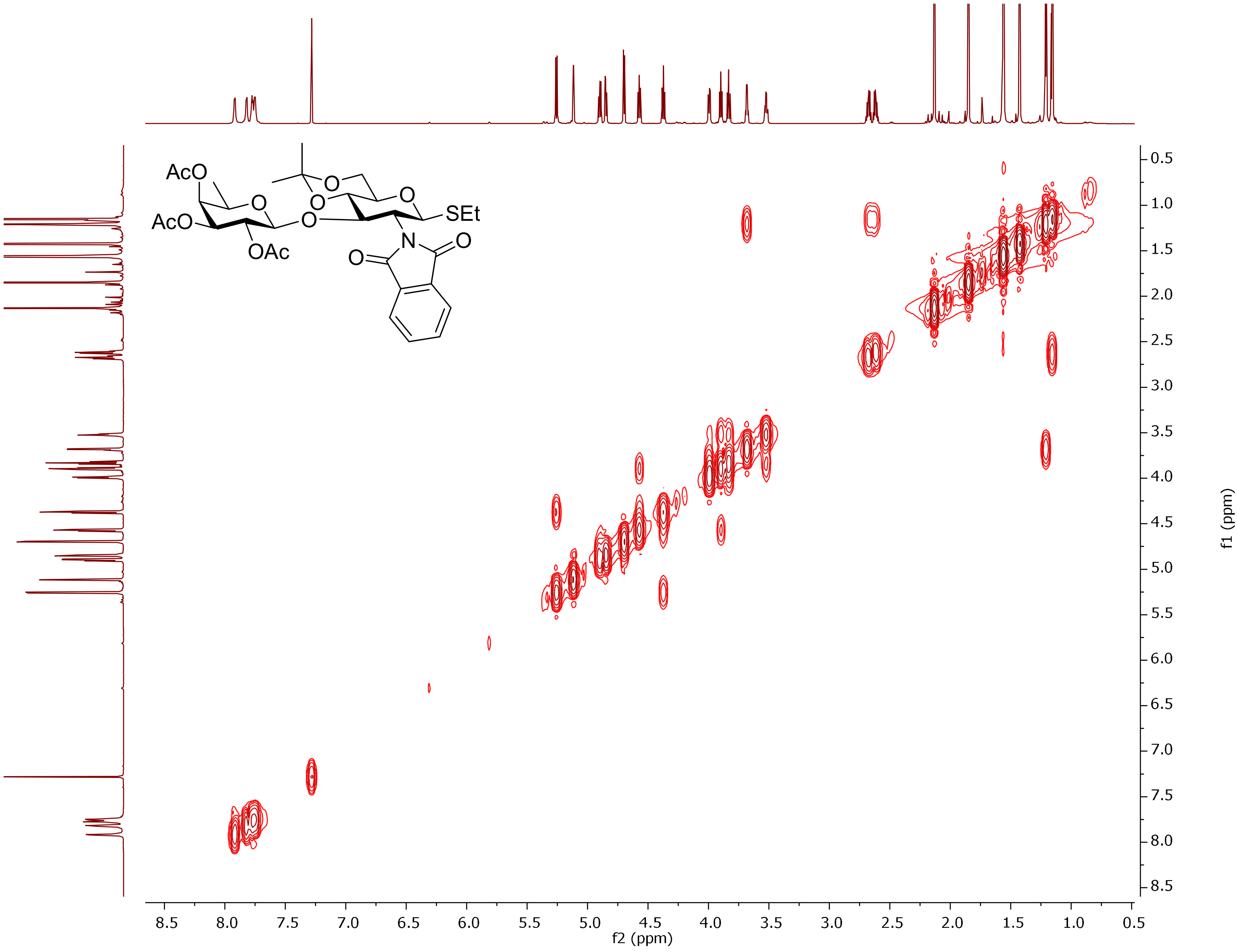

HSQC NMR (900 MHz, $CDCl_3$) spectrum of ethyl 2-deoxy-2-phthalimido-4,6-*O*-isopropylidene-3-*O*-(2,3,4-tri-*O*-acetyl-β-D-fucopyranosyl)-1-thio-β-D-glucopyranoside (**25β**)

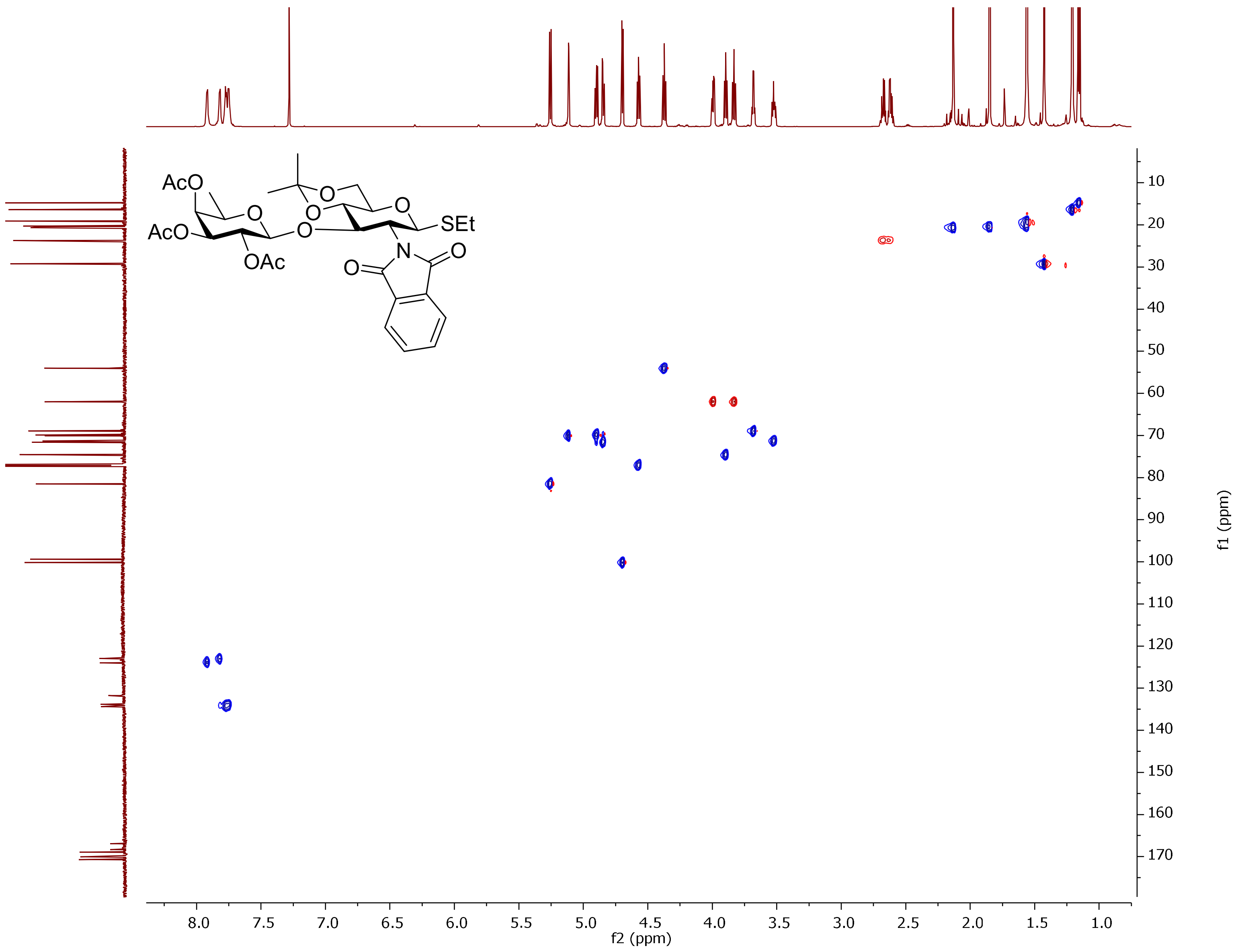

HMBC NMR (600 MHz, $CDCl_3$) spectrum of ethyl 2-deoxy-2-phthalimido-4,6-*O*-isopropylidene-3-*O*-(2,3,4-tri-*O*-acetyl-β-D-fucopyranosyl)-1-thio-β-D-glucopyranoside (**25β**)

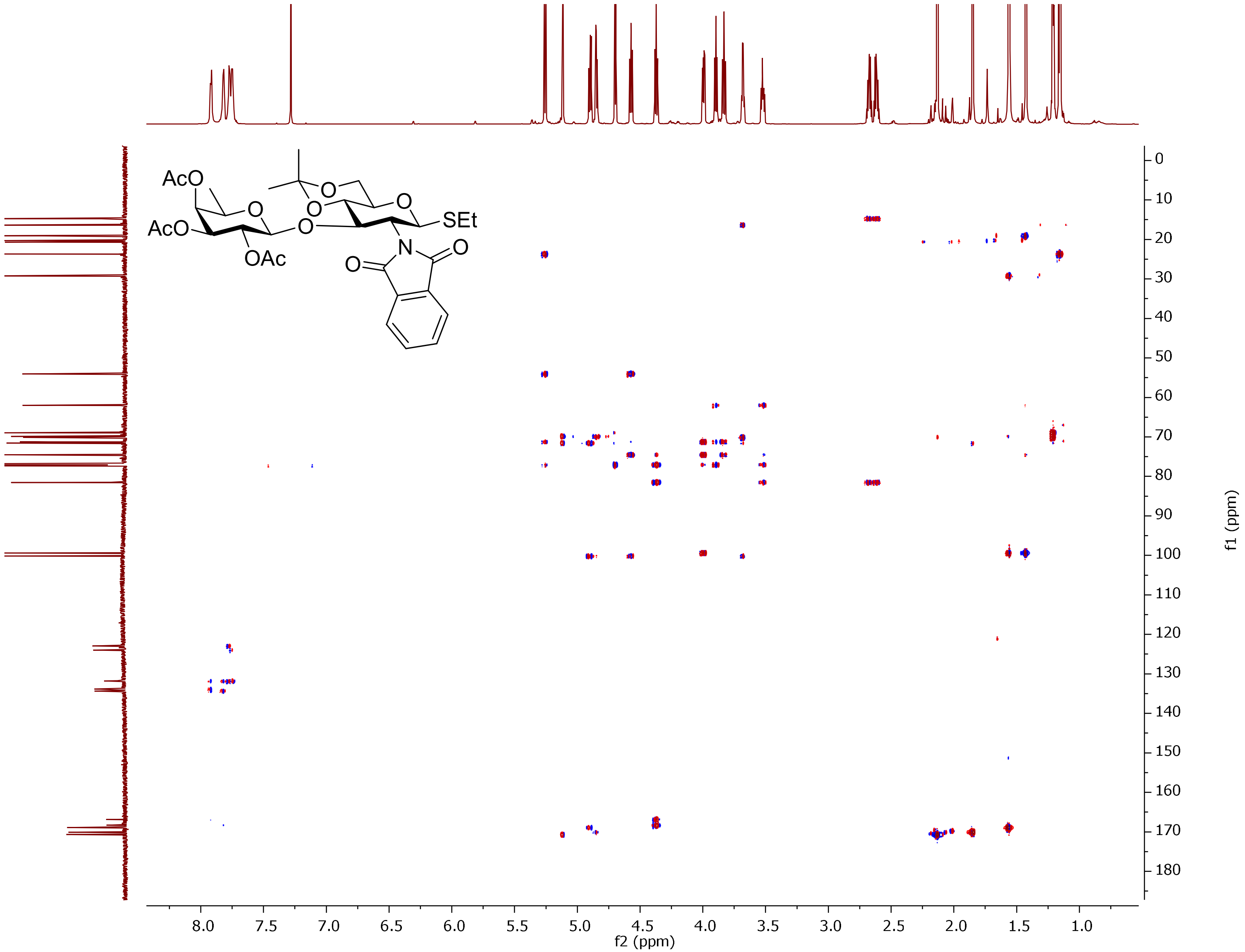

Crude ^{1}H NMR (600 MHz, $CDCl_3$) spectrum of **25** (Donor:Acceptor 1:1, 0.033 M, -25 ºC)

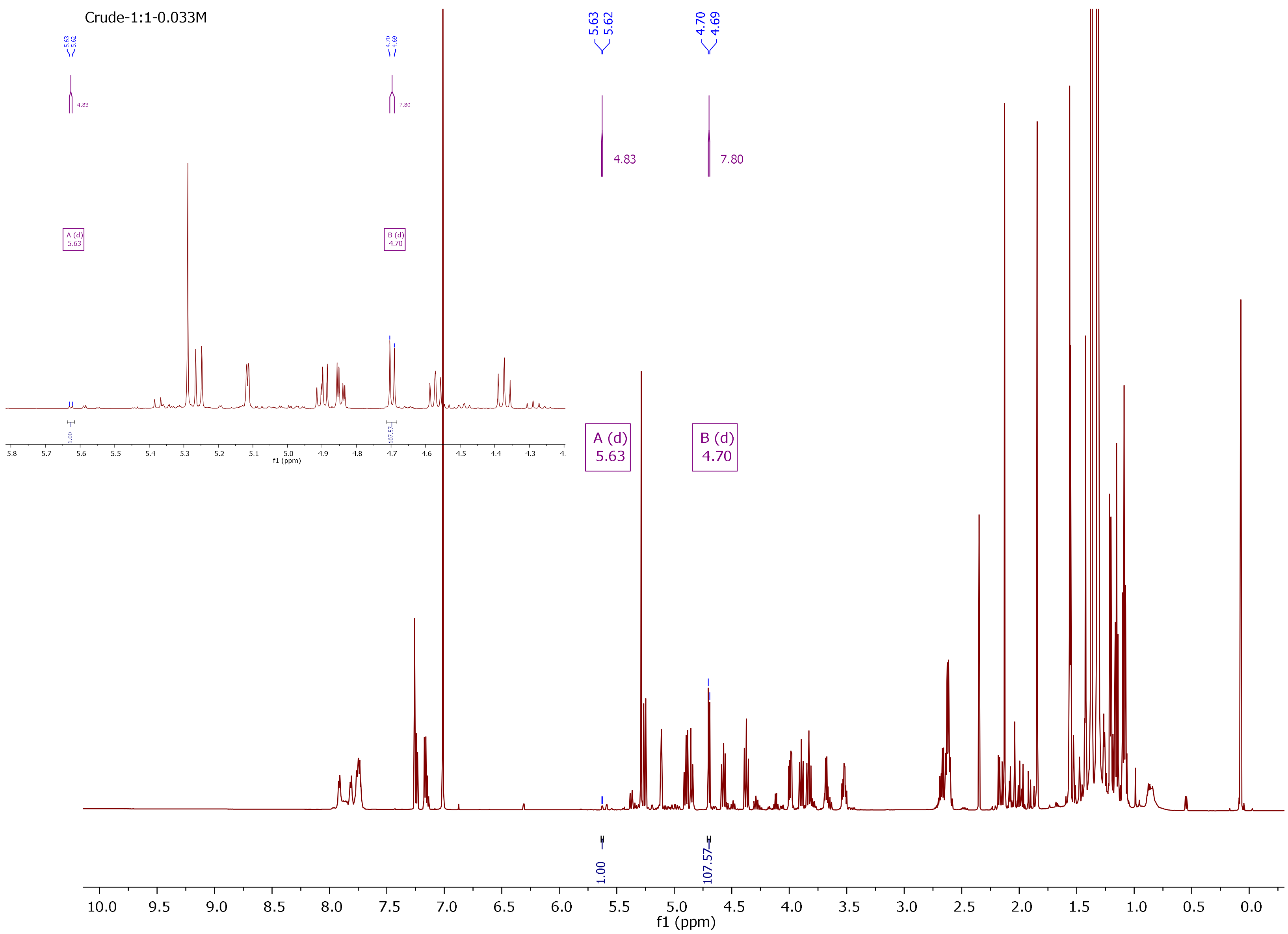

Crude ^{1}H NMR (600 MHz, $CDCl_3$) spectrum of **25** (Donor:Acceptor 1:1, 0.066 M, -25 ºC)

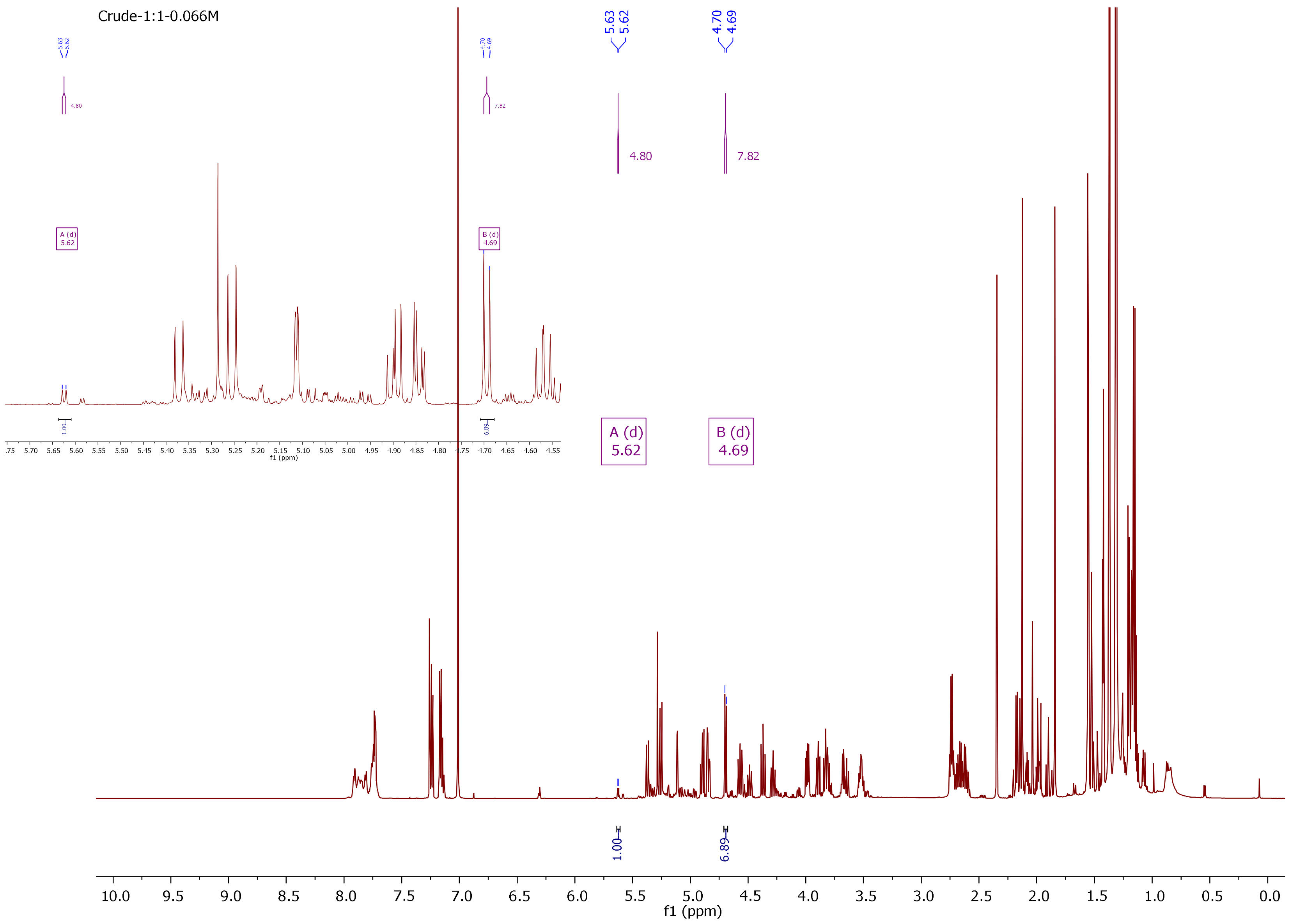

Crude ^{1}H NMR (600 MHz, $CDCl_3$) spectrum of **25** (Donor:Acceptor 1:1, 0.1 M, -25 ºC)

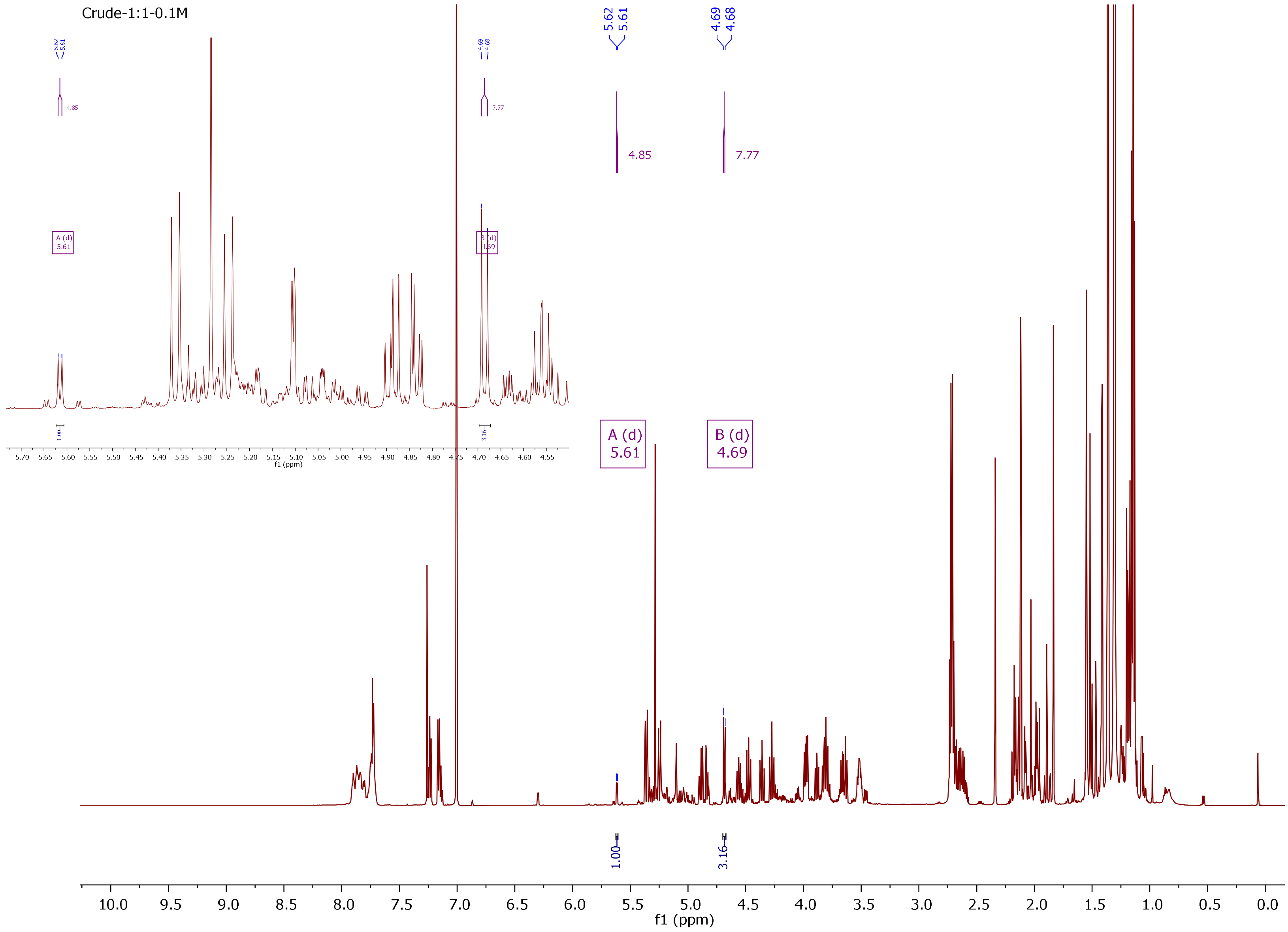

Crude ^{1}H NMR (600 MHz, $CDCl_3$) spectrum of **25** (Donor:Acceptor 1:1, 0.2 M, -25 ºC)

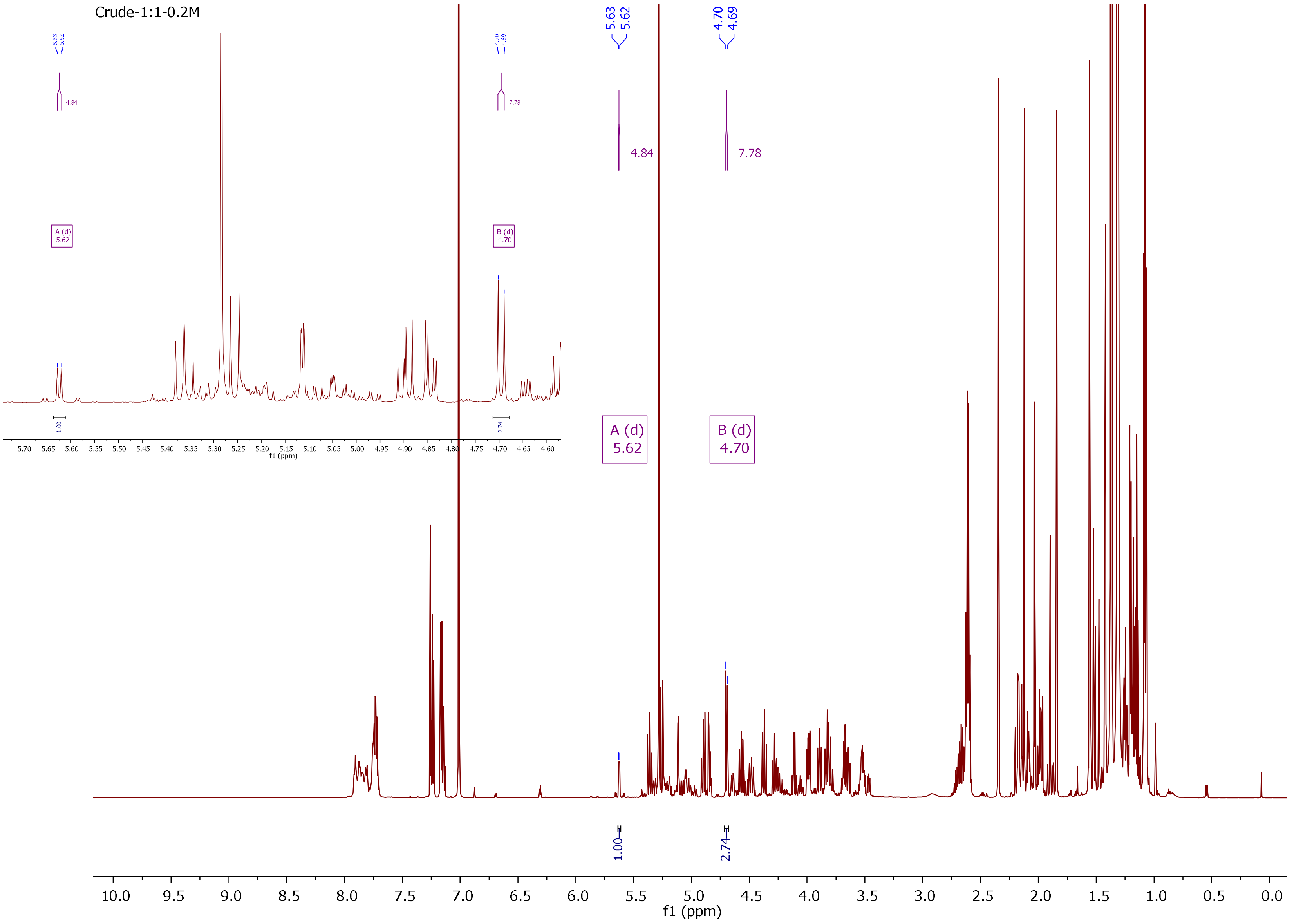

Crude ^{1}H NMR (600 MHz, $CDCl_3$) spectrum of **25** (Donor:Acceptor 1:1, 0.3 M, -25 ºC)

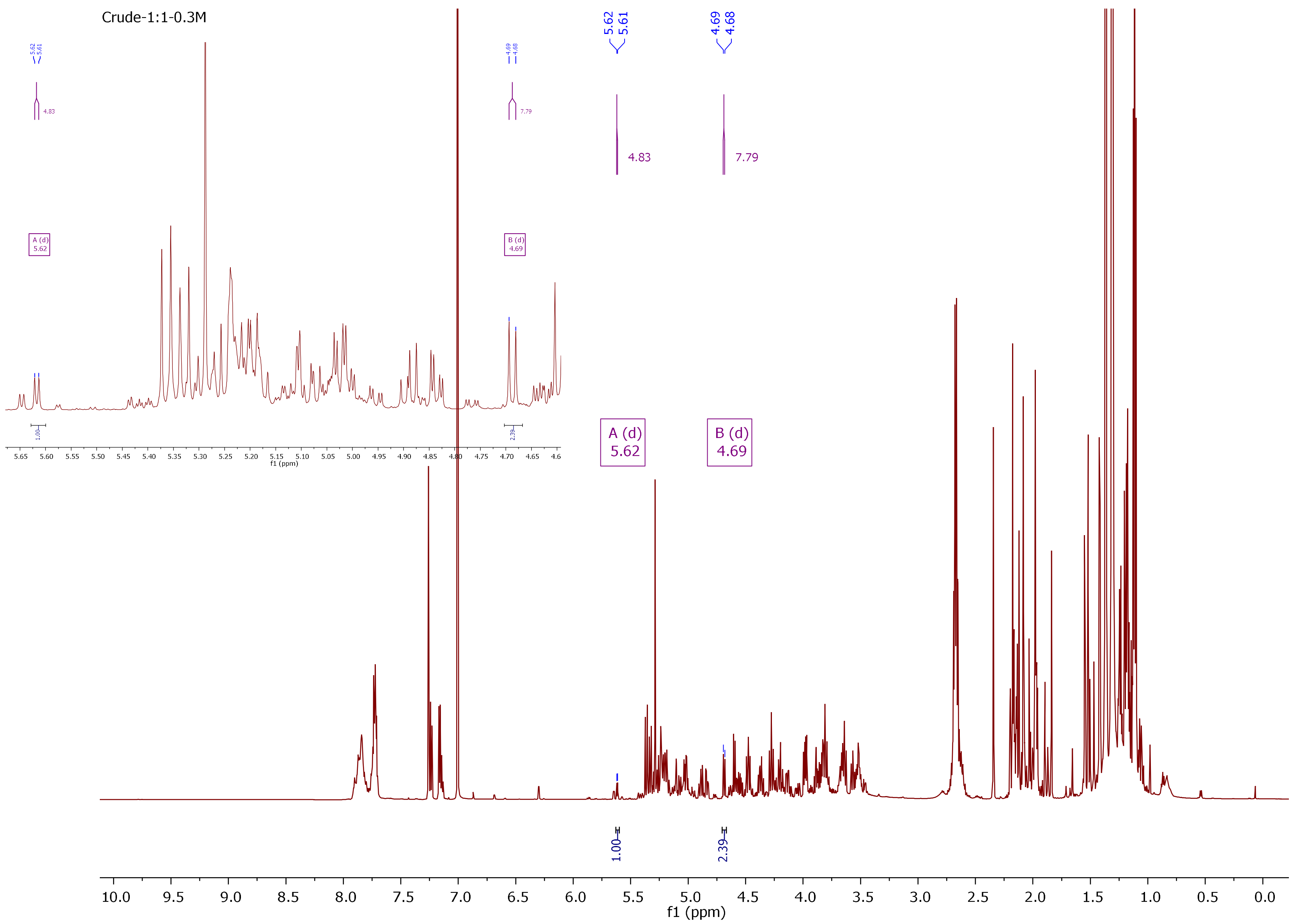

^{1}H NMR (600 MHz, $CDCl_3$) spectrum of ethyl 2-deoxy-2-phthalimido-4,6-*O*-isopropylidene-3-*O*-(2,3,4-tri-*O*-acetyl-β-L-fucopyranosyl)-1-thio-β-D-glucopyranoside (**26β**)

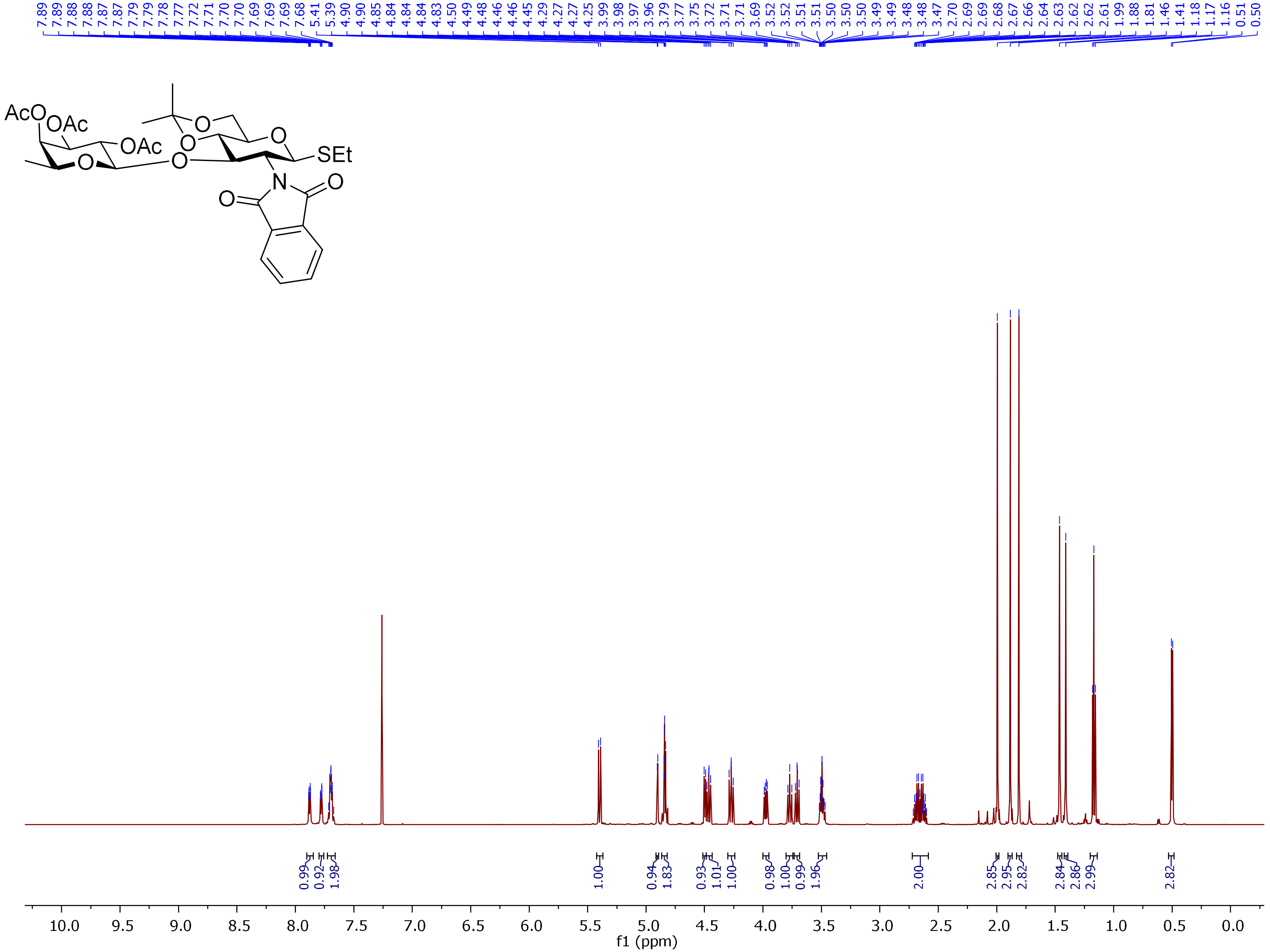

$^{13}C\{^{1}H\}$ NMR (151 MHz, $CDCl_3$) spectrum of ethyl 2-deoxy-2-phthalimido-4,6-*O*-isopropylidene-3-*O*-(2,3,4-tri-*O*-acetyl-β-L-fucopyranosyl)-1-thio-β-D-glucopyranoside (**26β**)

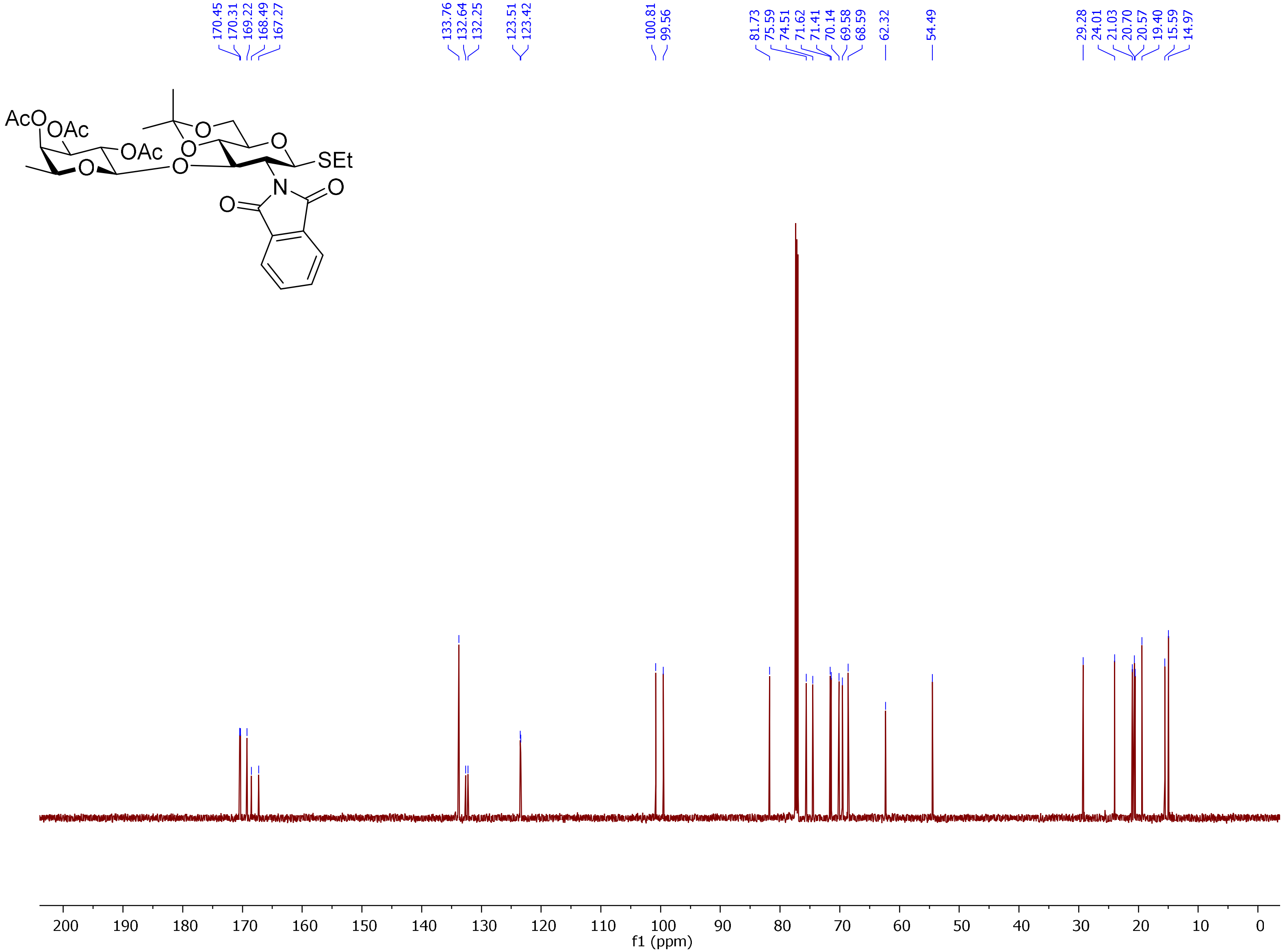

COSY NMR (600 MHz, $CDCl_3$) spectrum of ethyl 2-deoxy-2-phthalimido-4,6-*O*-isopropylidene-3-*O*-(2,3,4-tri-*O*-acetyl-β-L-fucopyranosyl)-1-thio-β-D-glucopyranoside (**26β**)

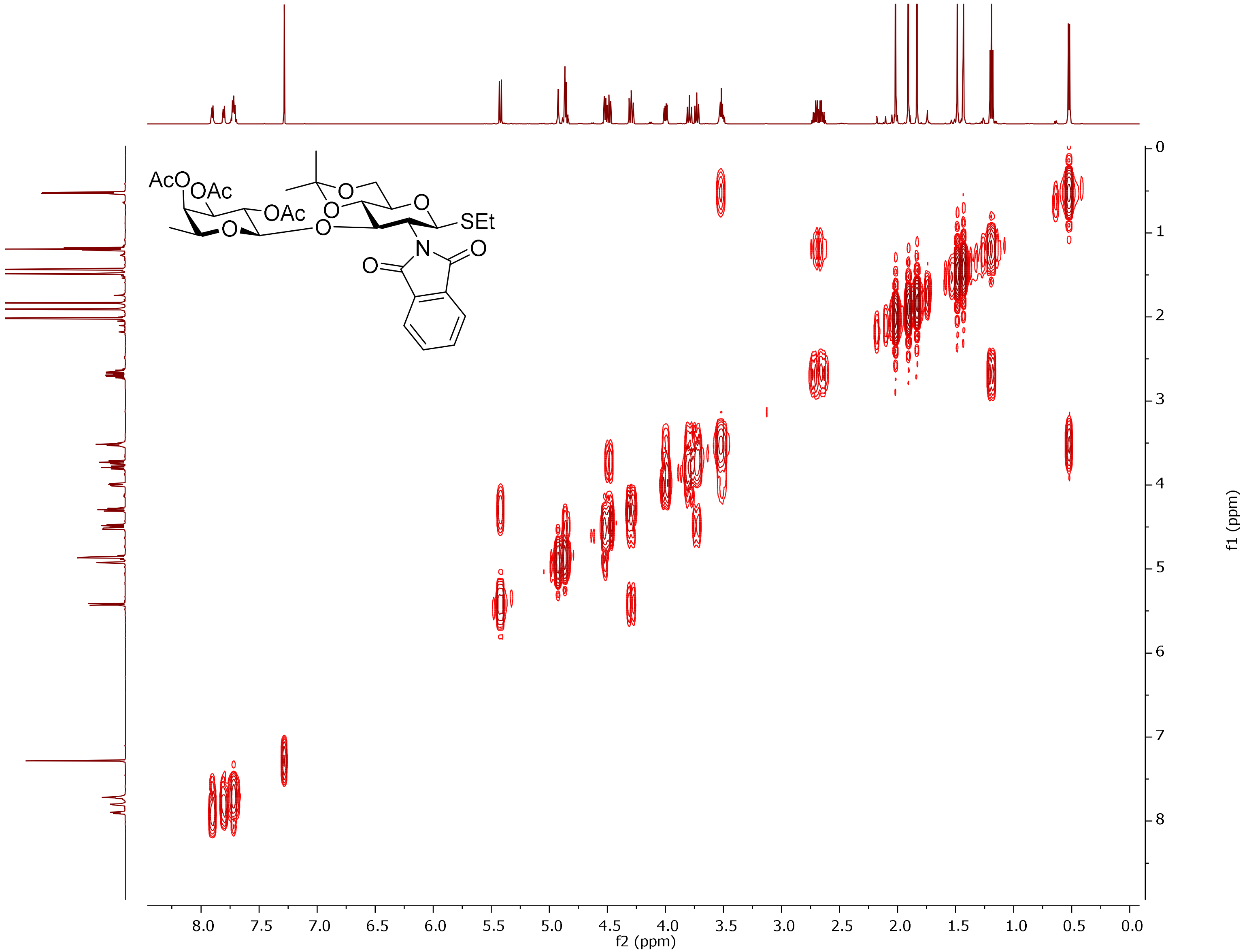

HSQC NMR (600 MHz, $CDCl_3$) spectrum of ethyl 2-deoxy-2-phthalimido-4,6-*O*-isopropylidene-3-*O*-(2,3,4-tri-*O*-acetyl-β-L-fucopyranosyl)-1-thio-β-D-glucopyranoside (**26β**)

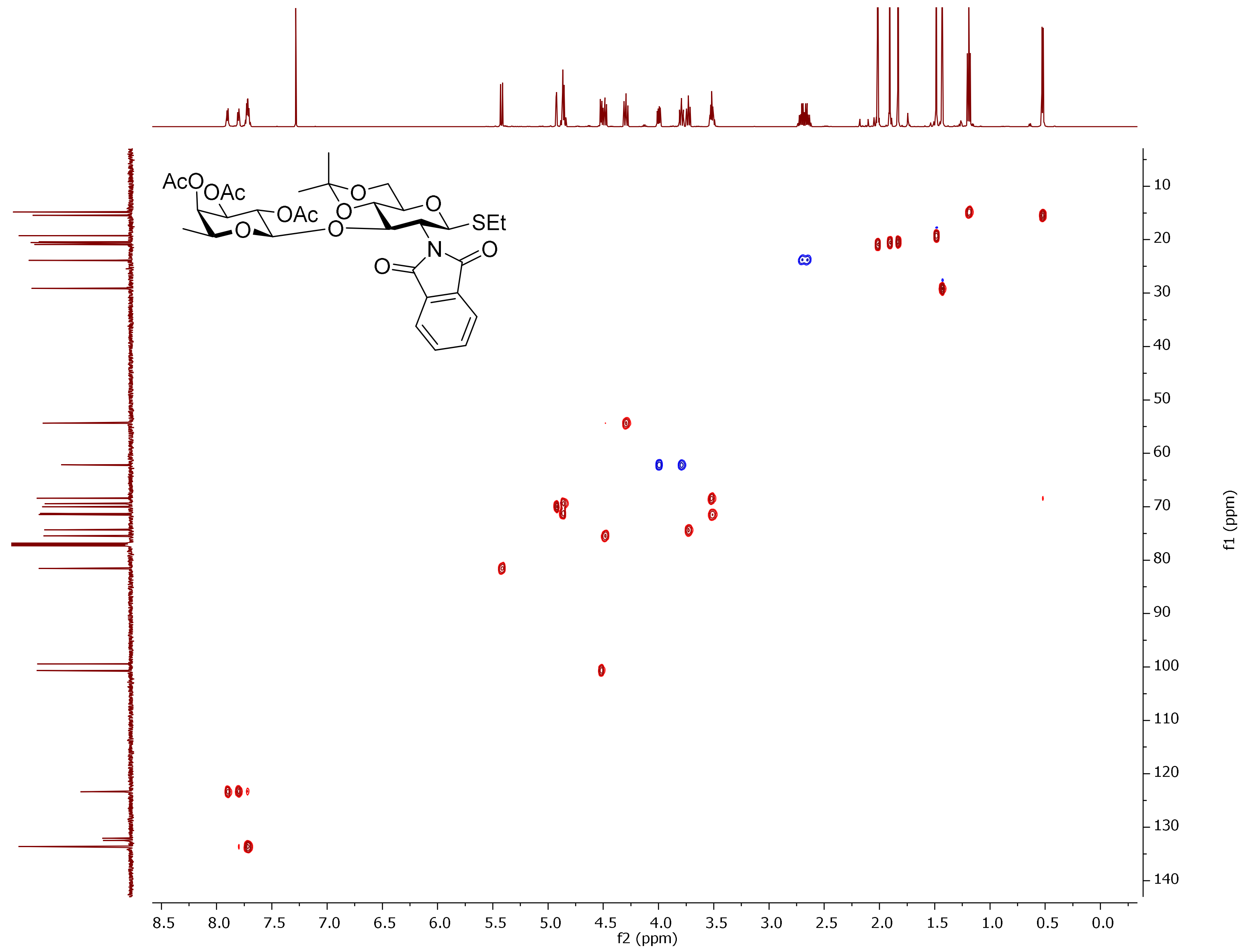

HMBC NMR (600 MHz, $CDCl_3$) spectrum of ethyl 2-deoxy-2-phthalimido-4,6-*O*-isopropylidene-3-*O*-(2,3,4-tri-*O*-acetyl-β-L-fucopyranosyl)-1-thio-β-D-glucopyranoside (**26β**)

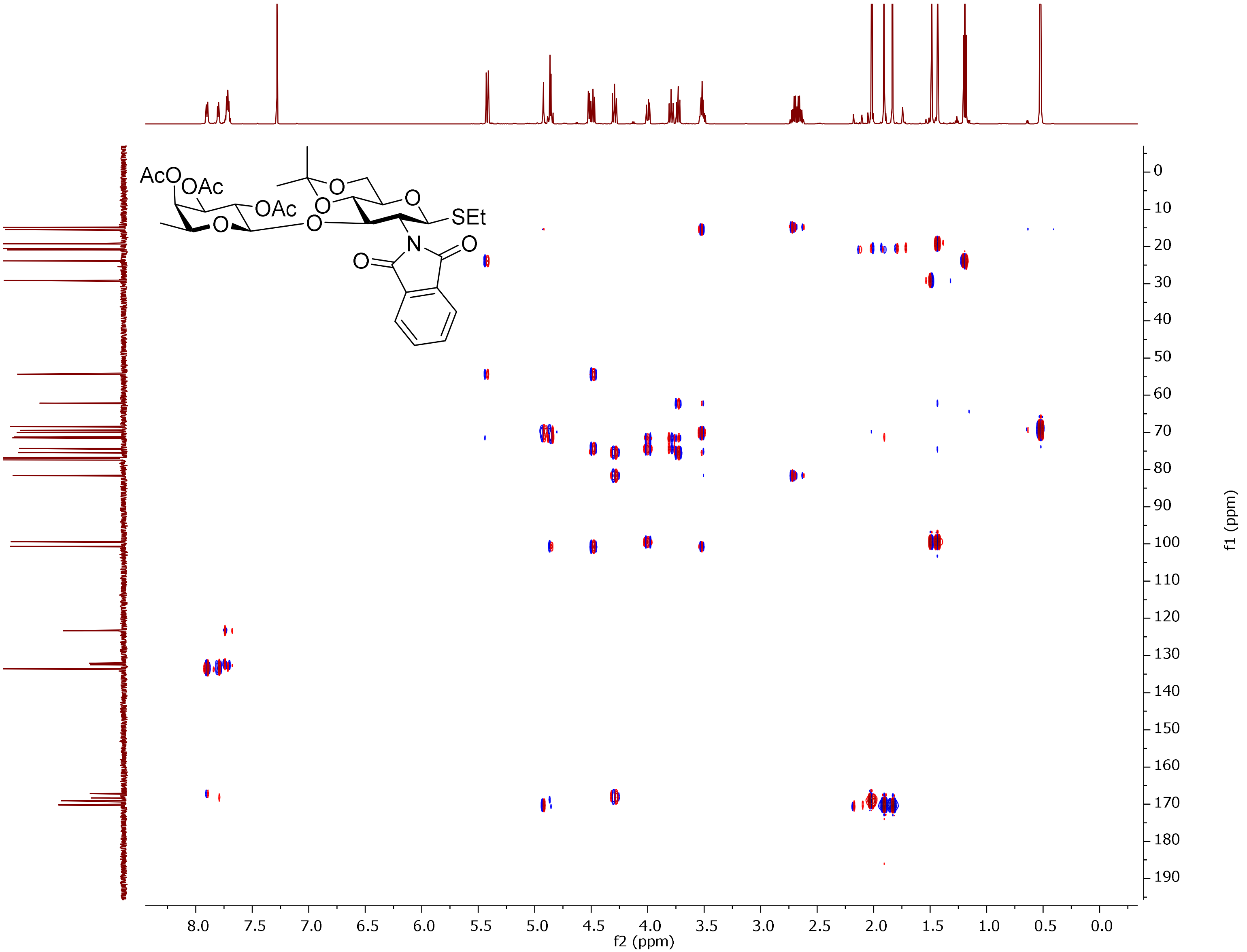

Crude ^{1}H NMR (600 MHz, $CDCl_3$) spectrum of **26** (Donor:Acceptor 1:1, 0.033 M, -25 ºC)

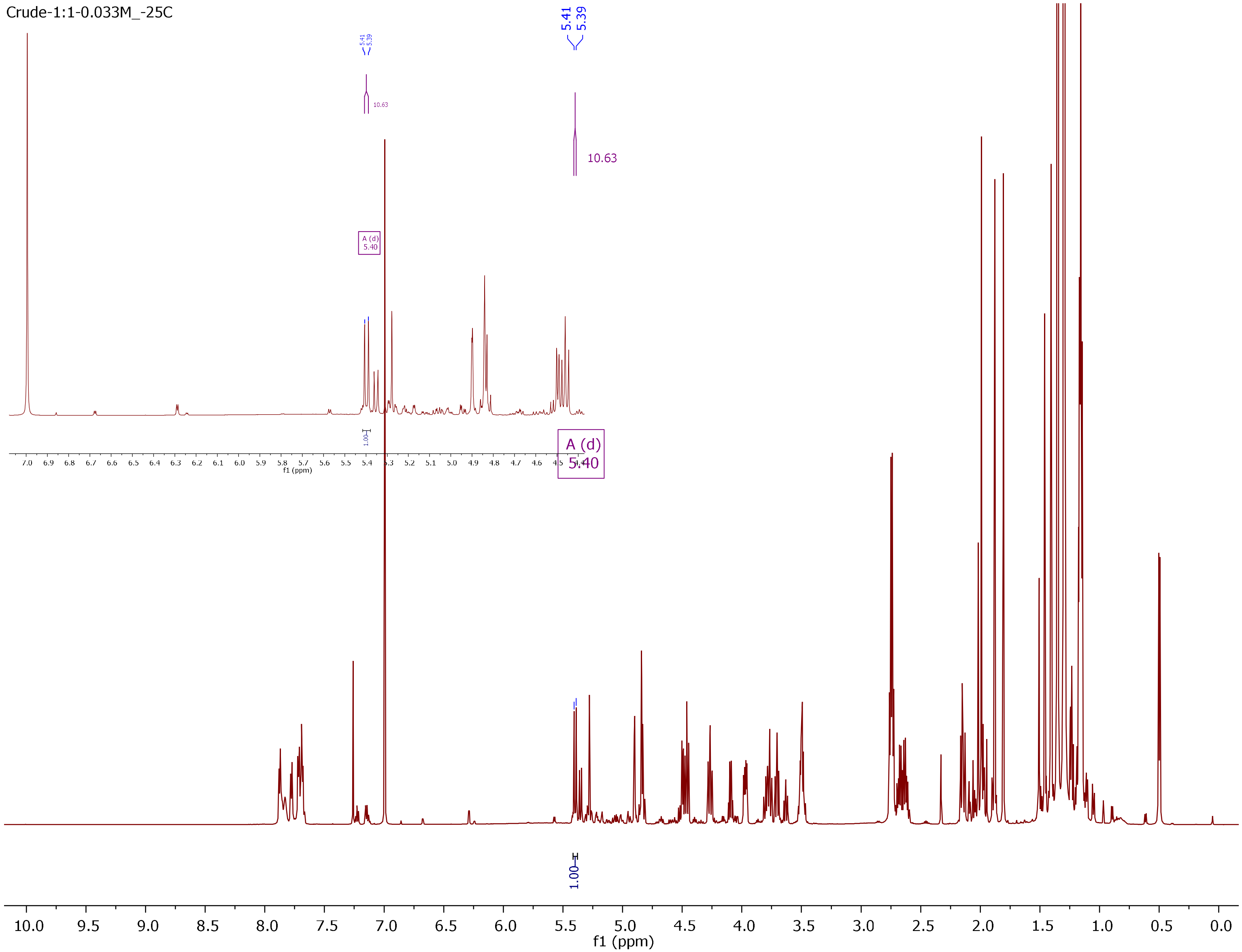

Crude ^{1}H NMR (600 MHz, $CDCl_3$) spectrum of **26** (Donor:Acceptor 1:1, 0.2 M, -25 ºC)

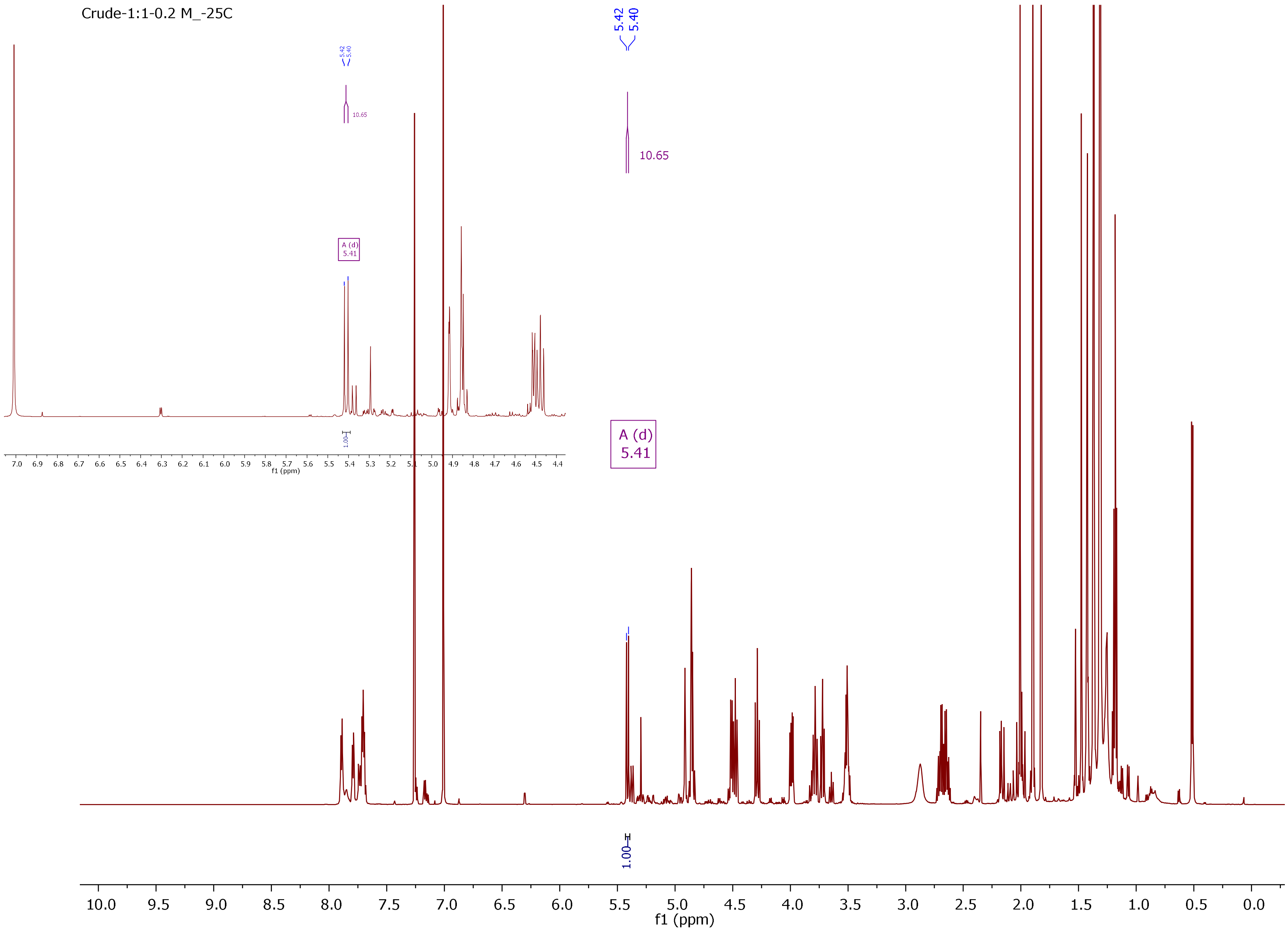

Crude ^{1}H NMR (600 MHz, $CDCl_3$) spectrum of **26** (Donor:Acceptor 1:1, 0.033 M, -45 ºC)

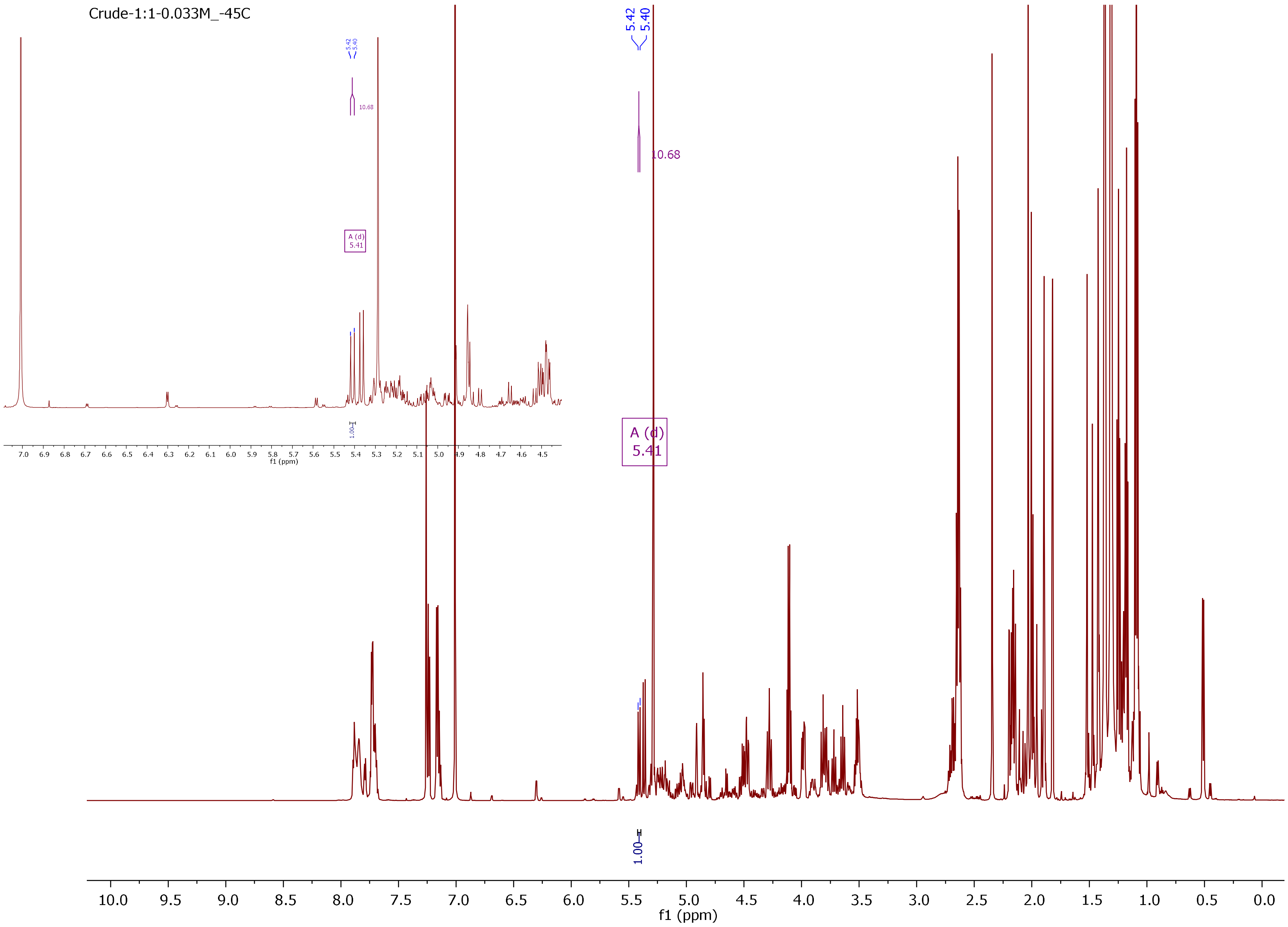

Crude ^{1}H NMR (600 MHz, $CDCl_3$) spectrum of **26** (Donor:Acceptor 1:1, 0.2 M, -45 ºC)

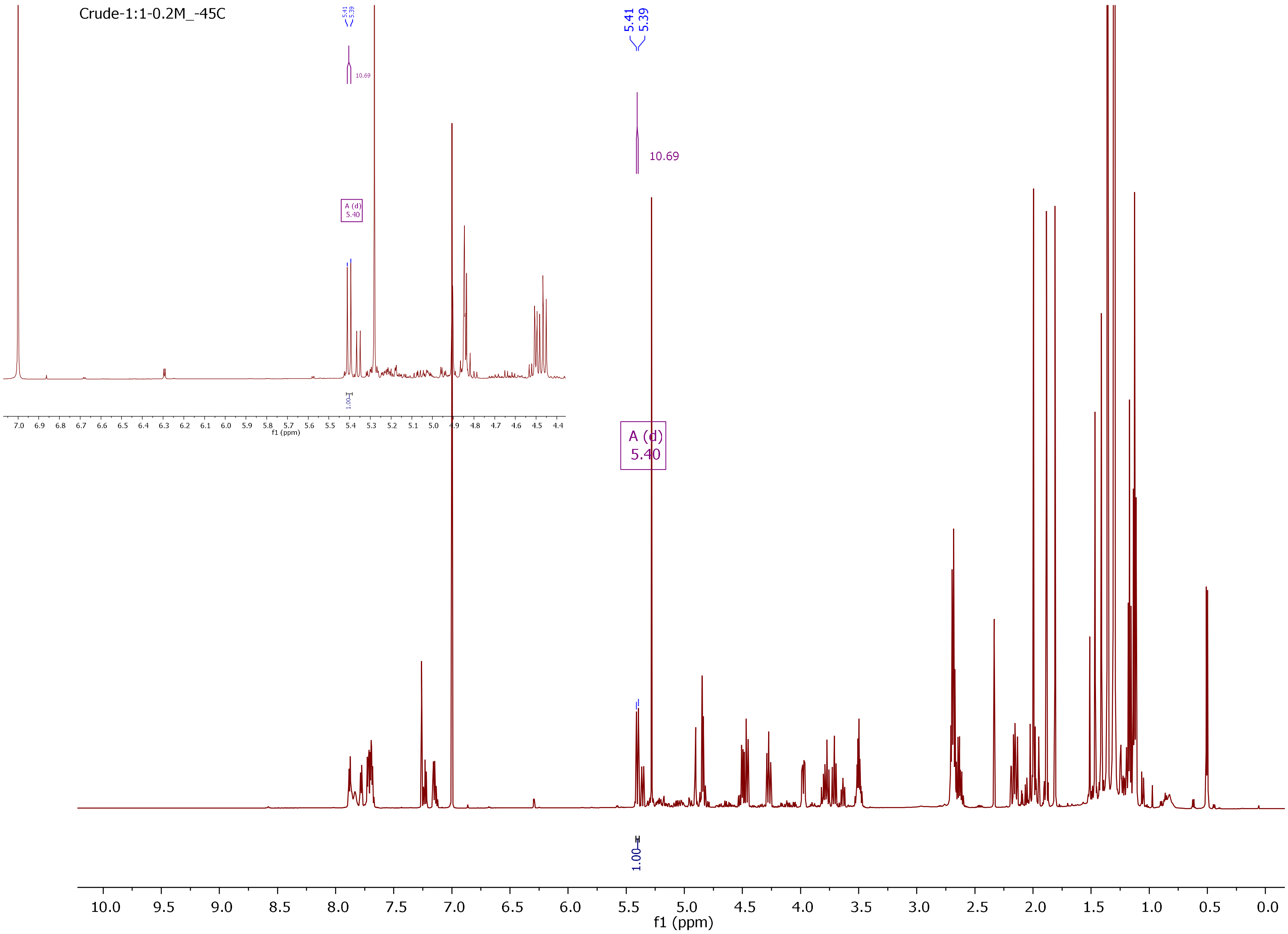

^{1}H NMR (600 MHz, $CDCl_3$) spectrum of 1,2:5,6-di-*O*-isopropylidene-3-*O*-(2-*O*-benzoyl-3-*O*-benzyl-4,6-*O*-isopropylidene-α-L-idopyranosyl)-α-D-glucopyranoside (**29α**)

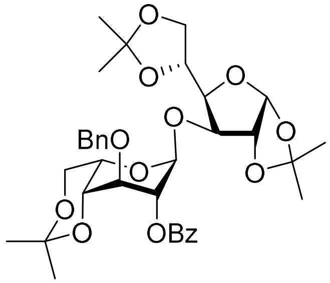

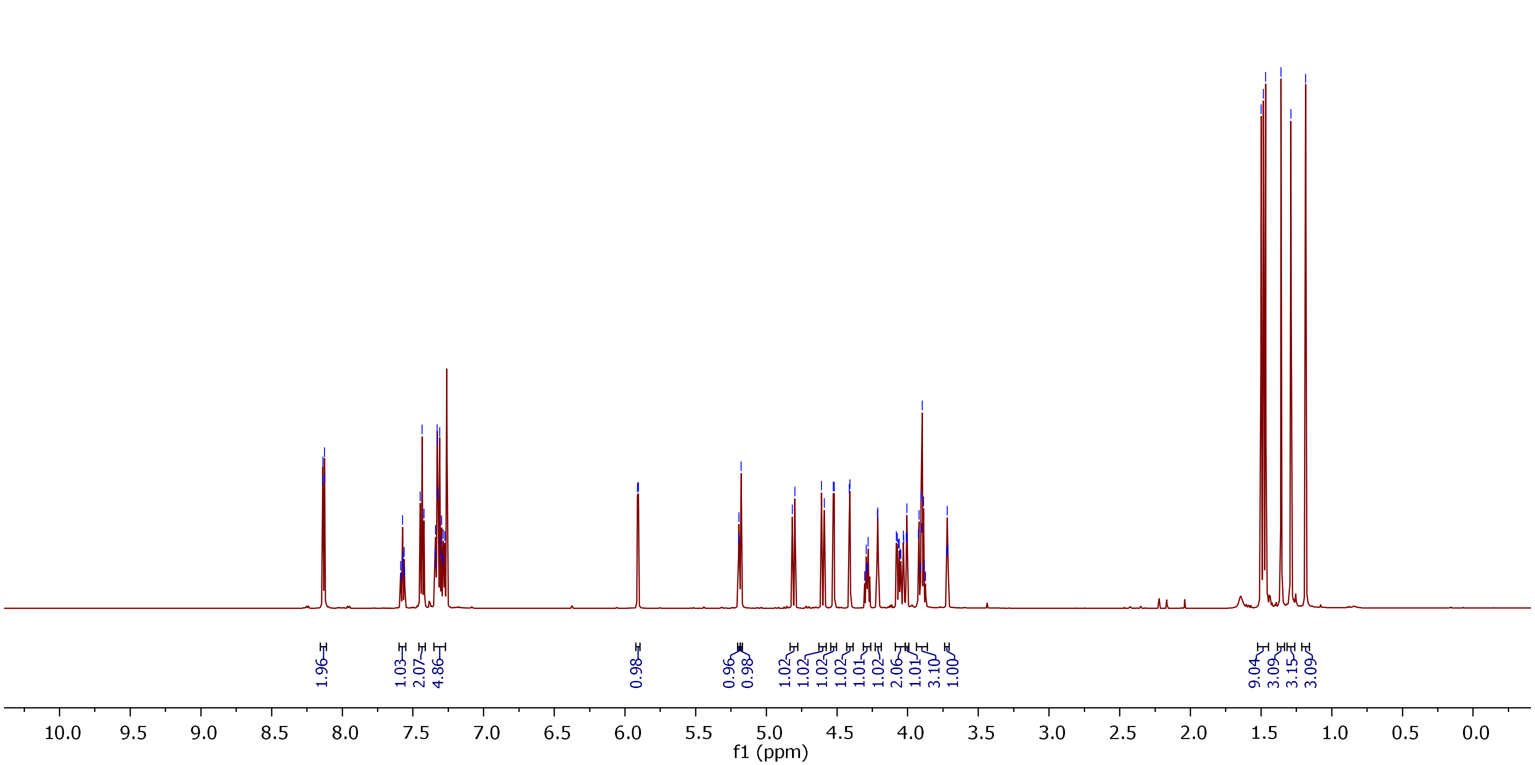

$^{13}C\{^{1}H\}$ NMR (151 MHz, $CDCl_3$) spectrum of 1,2:5,6-di-*O*-isopropylidene-3-*O*-(2-*O*-benzoyl-3-*O*-benzyl-4,6-*O*-isopropylidene-α-L-idopyranosyl)-α-D-glucopyranoside (**29α**)

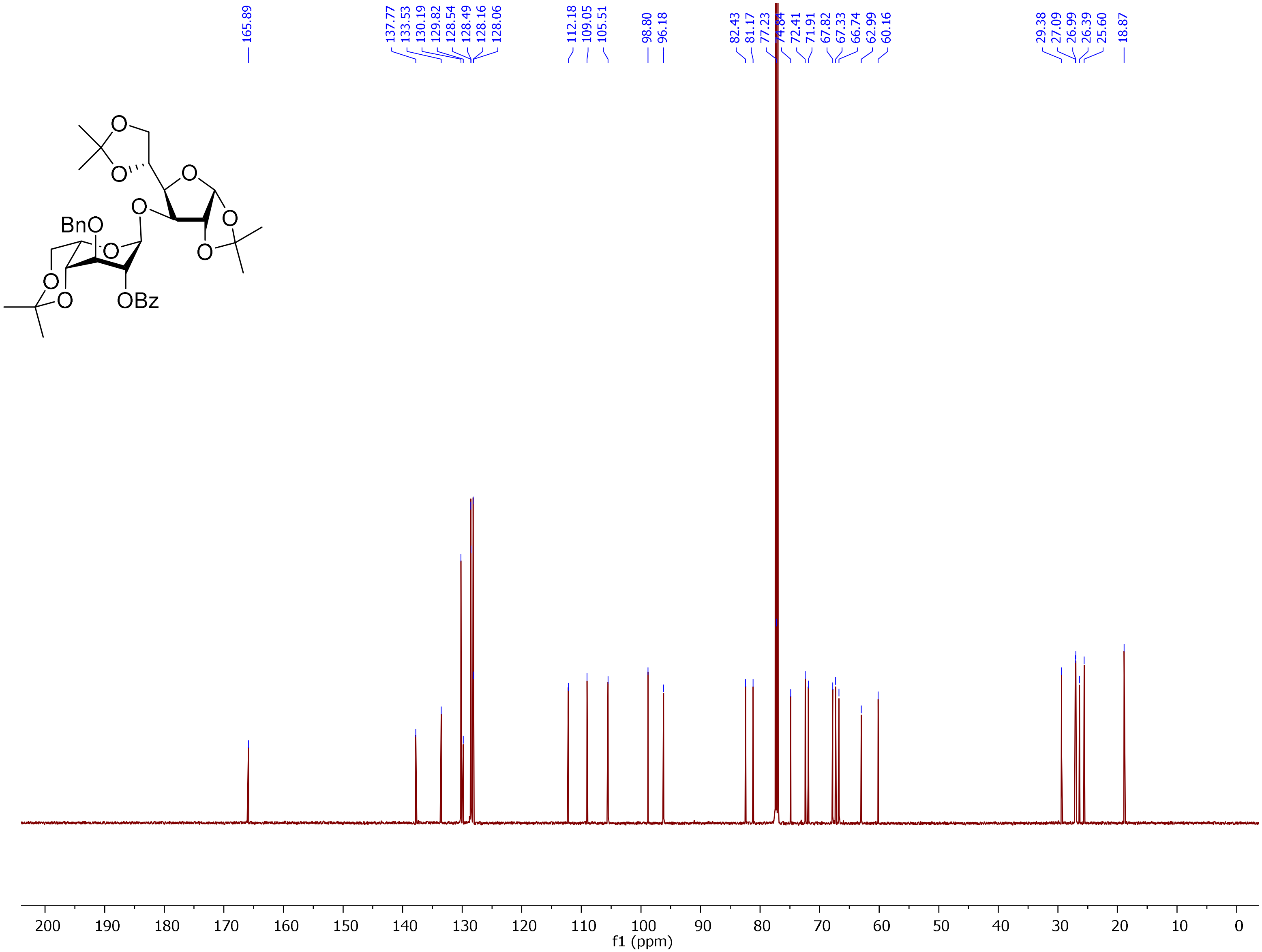

COSY NMR (600 MHz, $CDCl_3$) spectrum of 1,2:5,6-di-*O*-isopropylidene-3-*O*-(2-*O*-benzoyl-3-*O*-benzyl-4,6-*O*-isopropylidene-α-L-idopyranosyl)-α-D-glucopyranoside (**29α**)

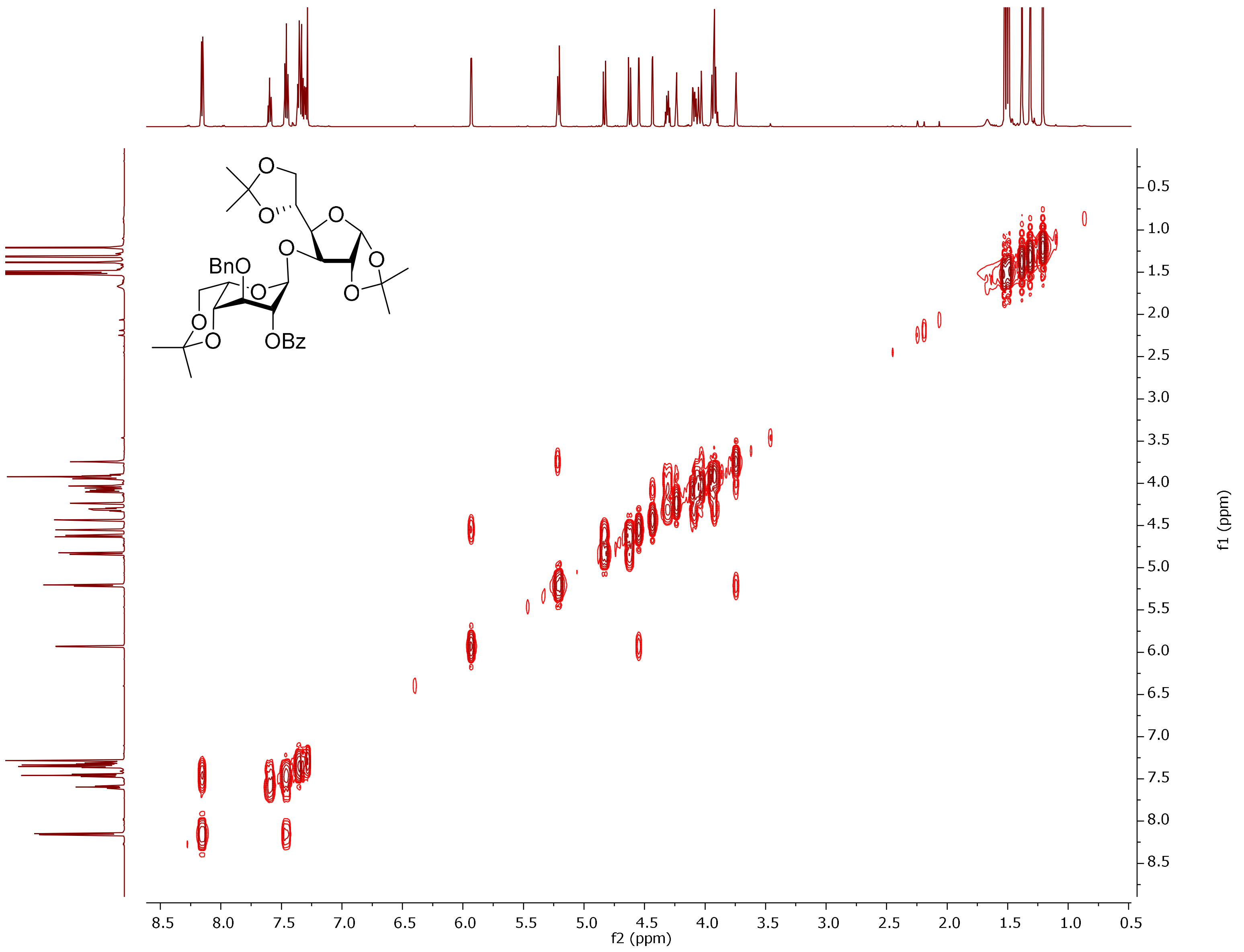

HSQC NMR (600 MHz, $CDCl_3$) spectrum of 1,2:5,6-di-*O*-isopropylidene-3-*O*-(2-*O*-benzoyl-3-*O*-benzyl-4,6-*O*-isopropylidene-α-L-idopyranosyl)-α-D-glucopyranoside (**29α**)

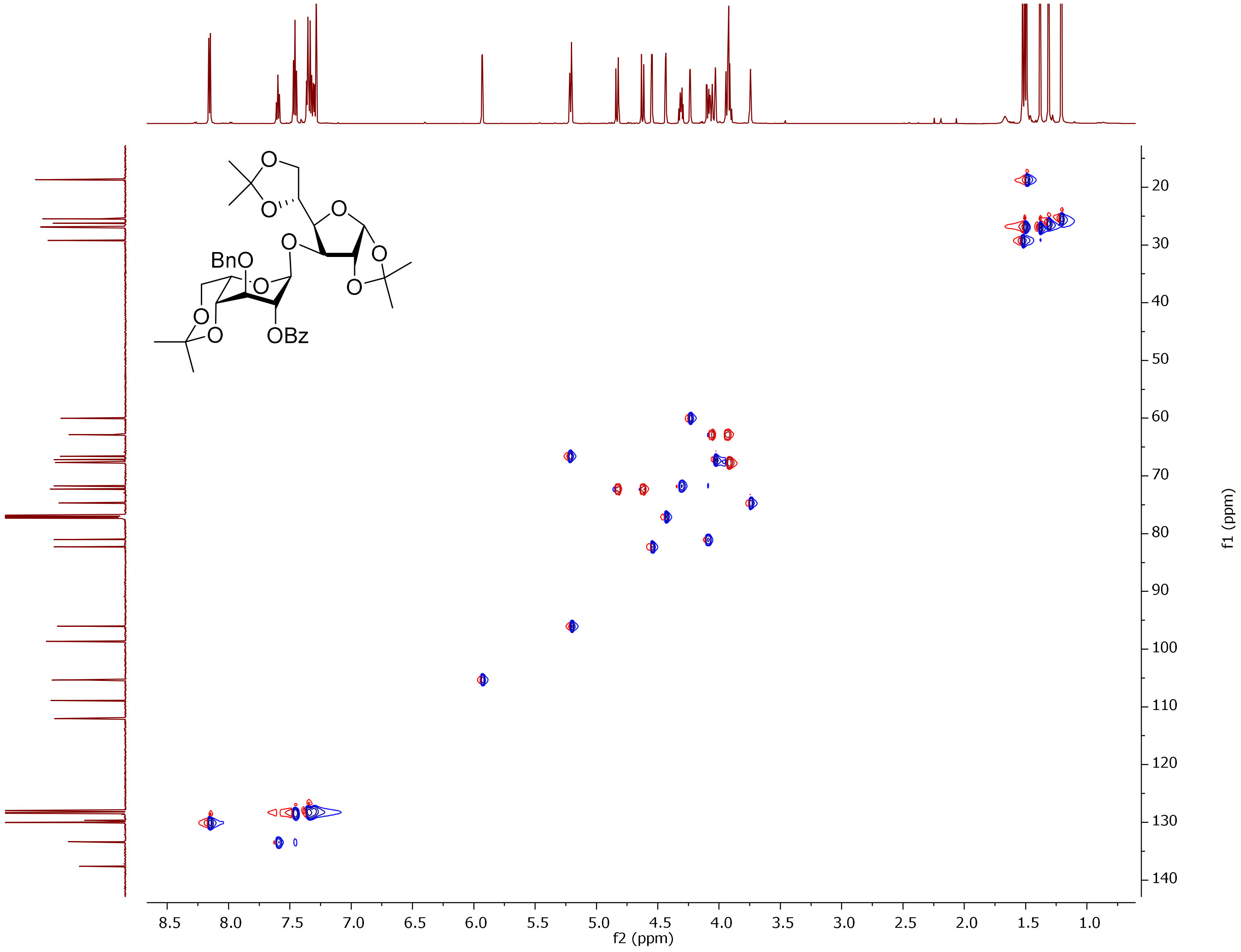

HMBC NMR (600 MHz, $CDCl_3$) spectrum of 1,2:5,6-di-*O*-isopropylidene-3-*O*-(2-*O*-benzoyl-3-*O*-benzyl-4,6-*O*-isopropylidene-α-L-idopyranosyl)-α-D-glucopyranoside (**29α**)

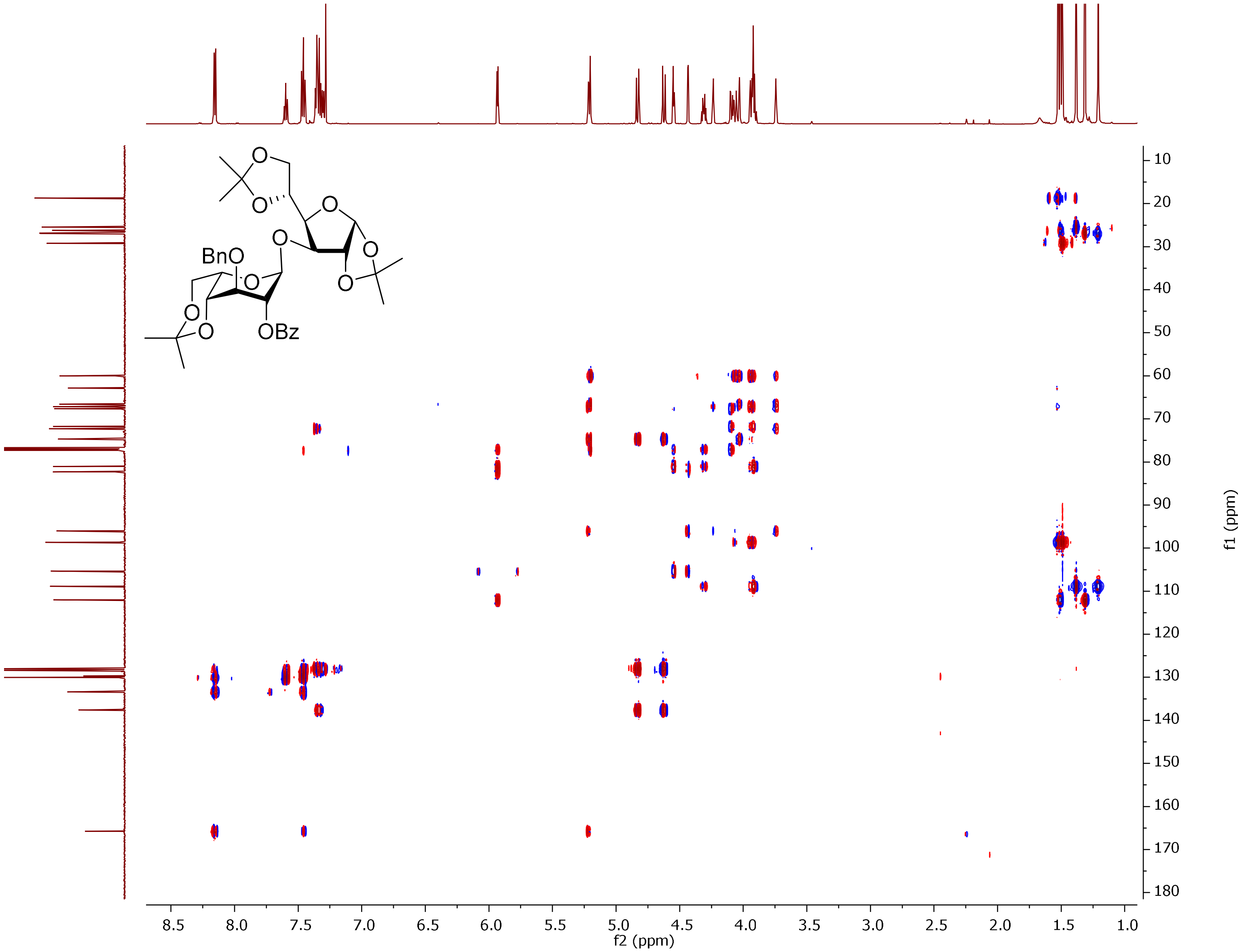

^{1}H NMR (600 MHz, $CDCl_3$) spectrum of 1,2:5,6-di-*O*-isopropylidene-3-*O*-(2-*O*-benzoyl-3-*O*-benzyl-4,6-*O*-isopropylidene-β-L-idopyranosyl)-α-D-glucopyranoside (**29β**)

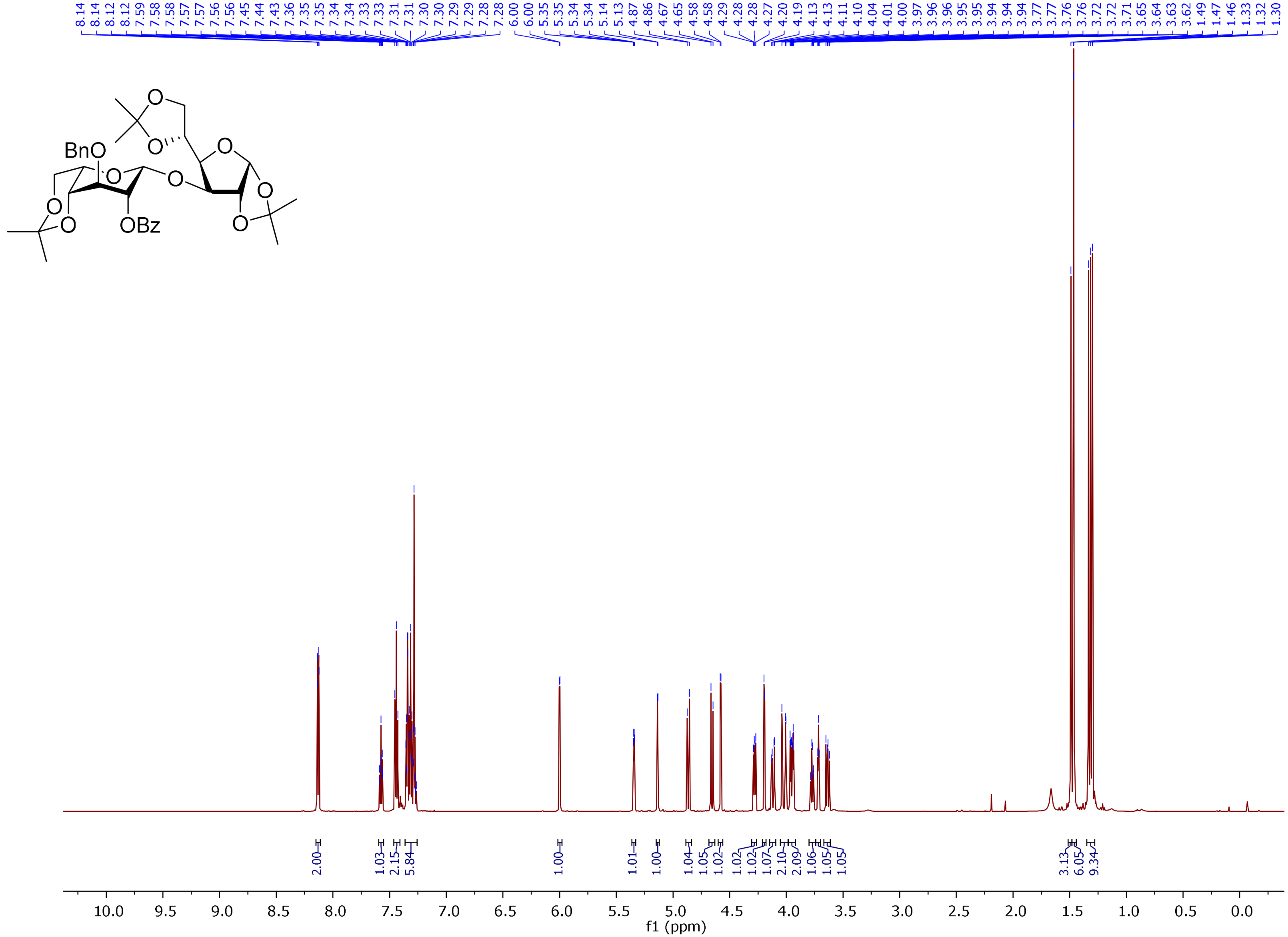

$^{13}C\{^{1}H\}$ NMR (151 MHz, $CDCl_3$) spectrum of 1,2:5,6-di-*O*-isopropylidene-3-*O*-(2-*O*-benzoyl-3-*O*-benzyl-4,6-*O*-isopropylidene-β-L-idopyranosyl)-α-D-glucopyranoside (**29β**)

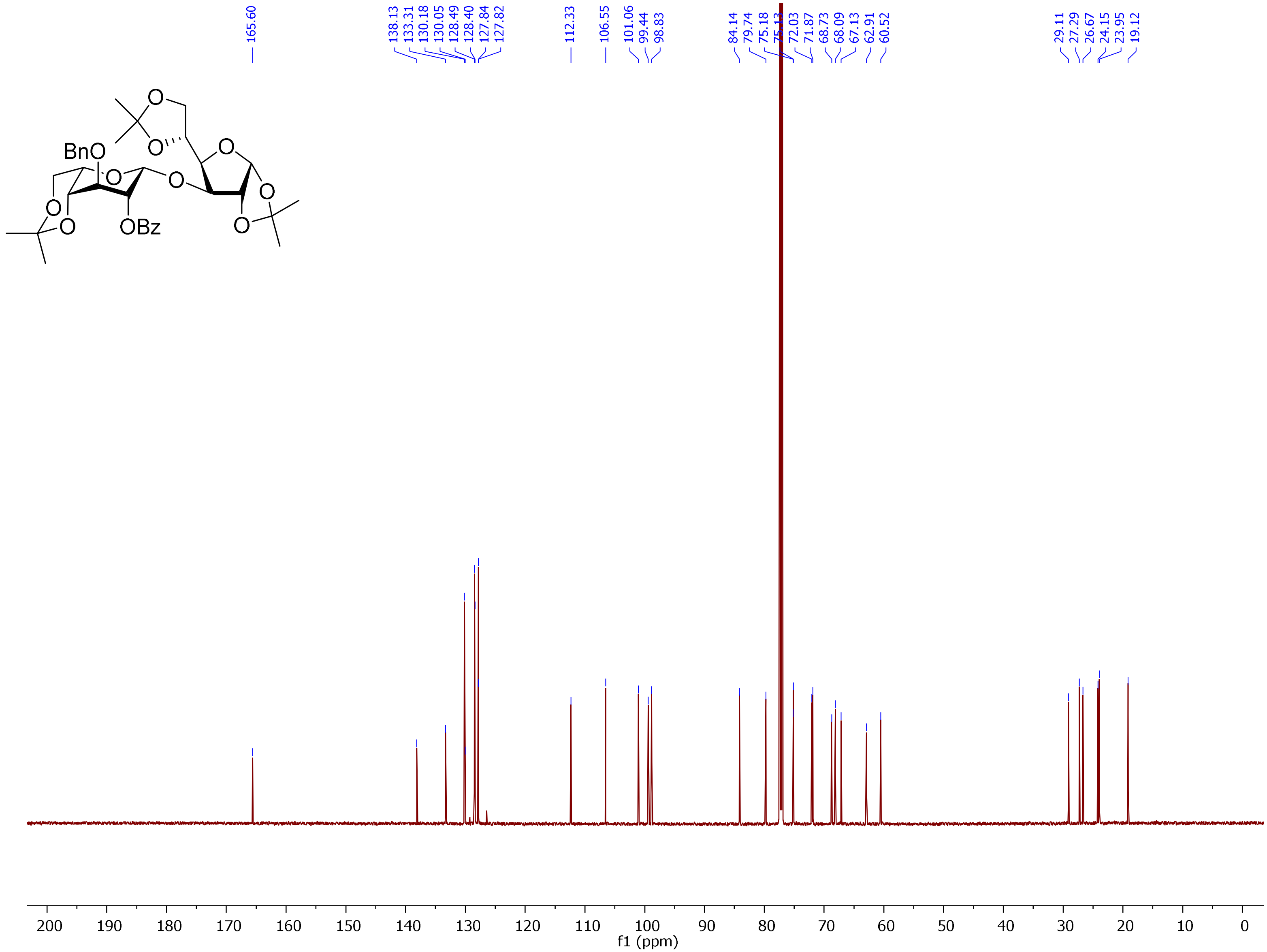

COSY NMR (600 MHz, $CDCl_3$) spectrum of 1,2:5,6-di-*O*-isopropylidene-3-*O*-(2-*O*-benzoyl-3-*O*-benzyl-4,6-*O*-isopropylidene-β-L-idopyranosyl)-α-D-glucopyranoside (**29β**)

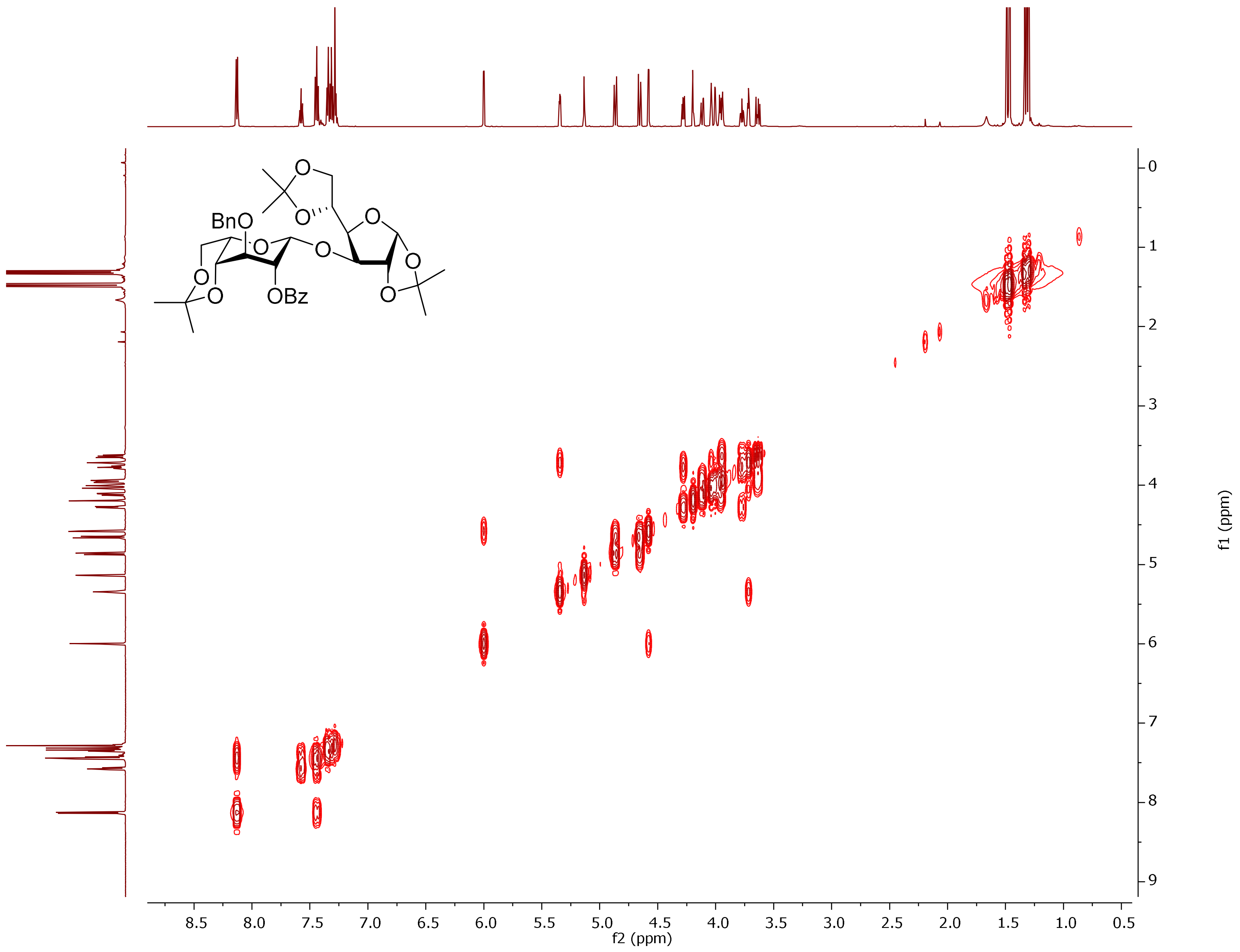

HSQC NMR (600 MHz, $CDCl_3$) spectrum of 1,2:5,6-di-*O*-isopropylidene-3-*O*-(2-*O*-benzoyl-3-*O*-benzyl-4,6-*O*-isopropylidene-β-L-idopyranosyl)-α-D-glucopyranoside (**29β**)

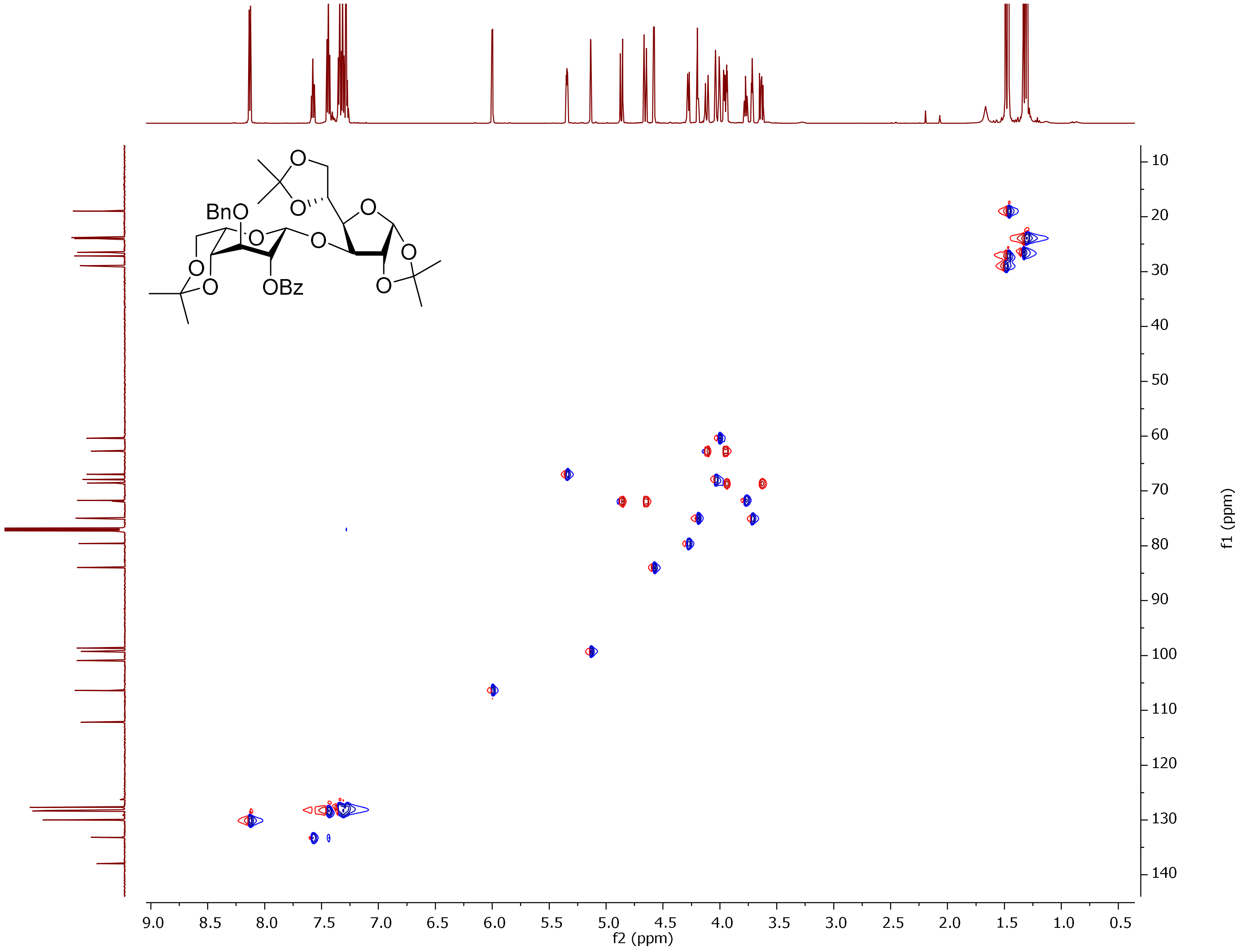

HMBC NMR (600 MHz, $CDCl_3$) spectrum of 1,2:5,6-di-*O*-isopropylidene-3-*O*-(2-*O*-benzoyl-3-*O*-benzyl-4,6-*O*-isopropylidene-β-L-idopyranosyl)-α-D-glucopyranoside (**29β**)

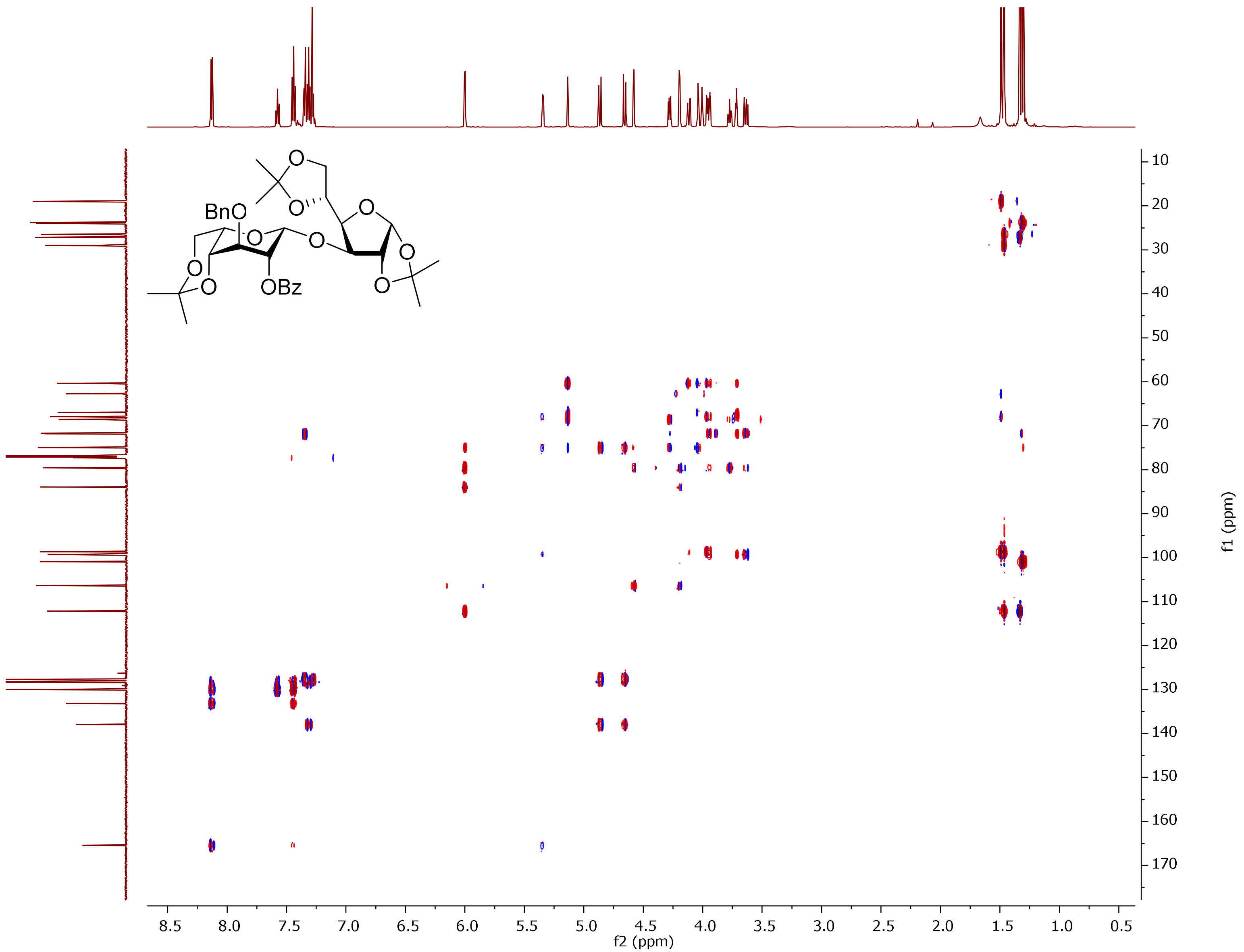

Crude ^{1}H NMR (600 MHz, $CDCl_3$) spectrum of **29** (Donor:Acceptor 1:0.25, 0.033 M, -20 ºC, Set-1)

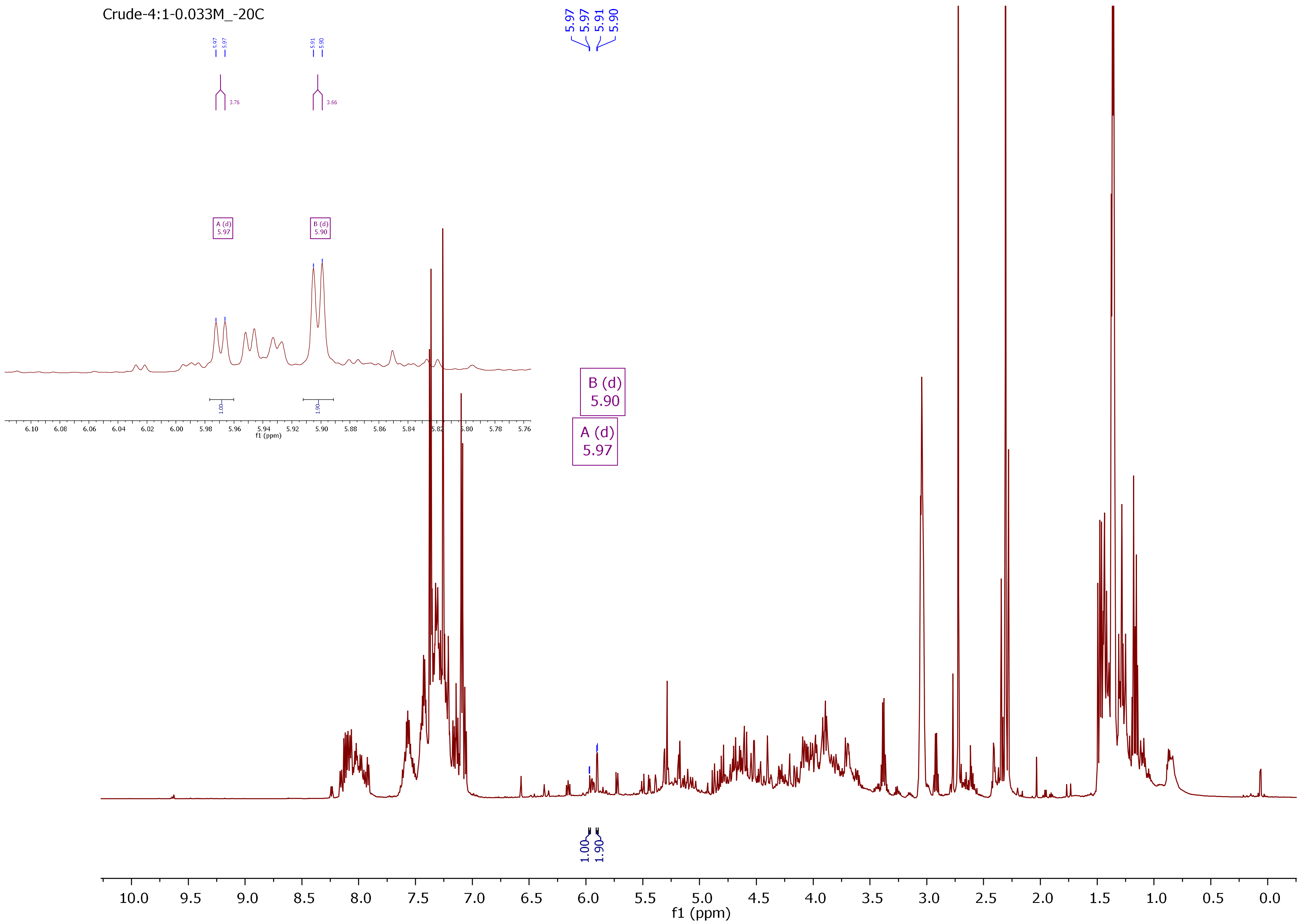

Crude ^{1}H NMR (600 MHz, $CDCl_3$) spectrum of **29** (Donor:Acceptor 1:0.25, 0.033 M, -20 ºC, Set-2)

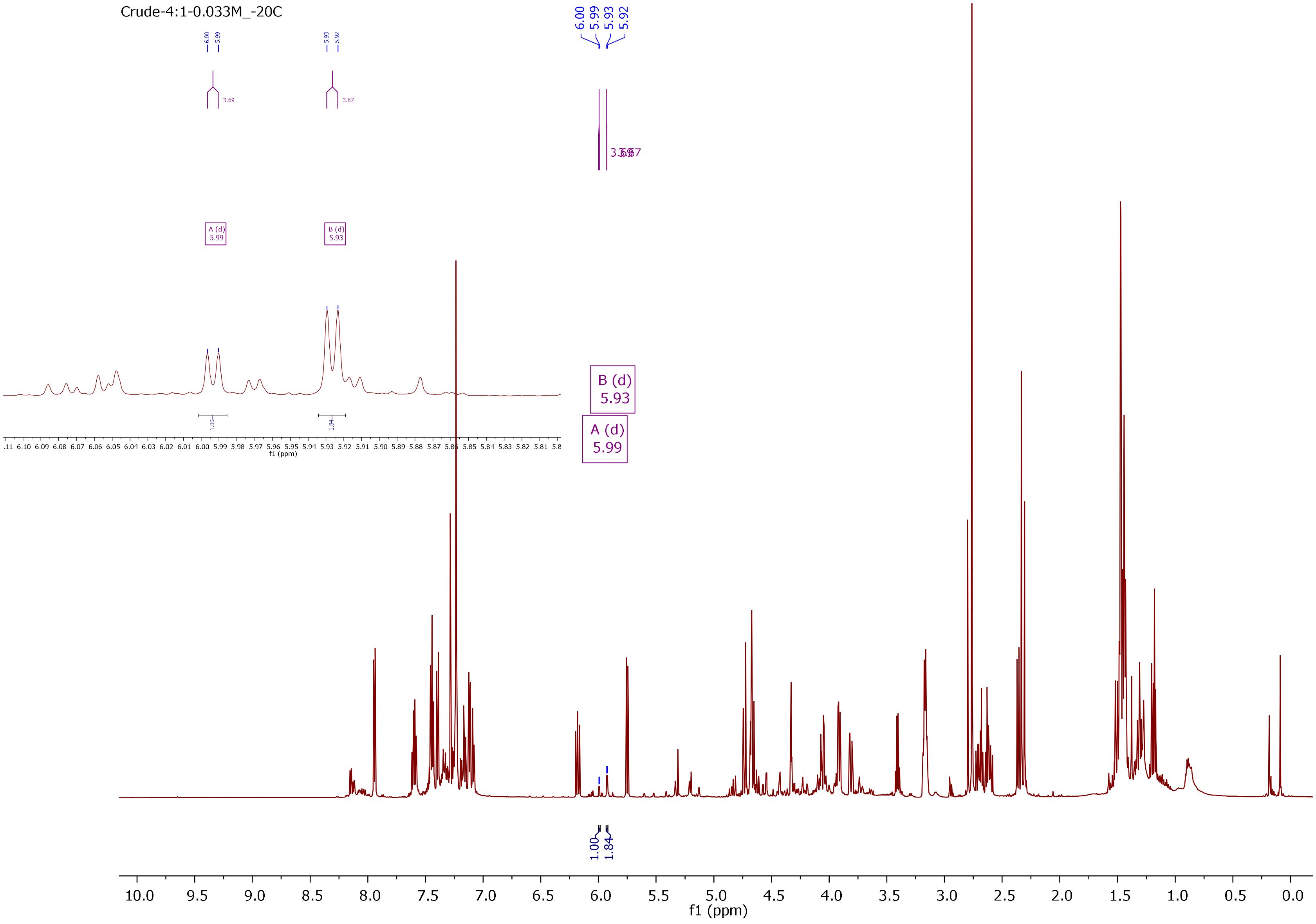

Crude ^{1}H NMR (600 MHz, $CDCl_3$) spectrum of **29** (Donor:Acceptor 1:0.5, 0.033 M, -20 ºC, Set-1)

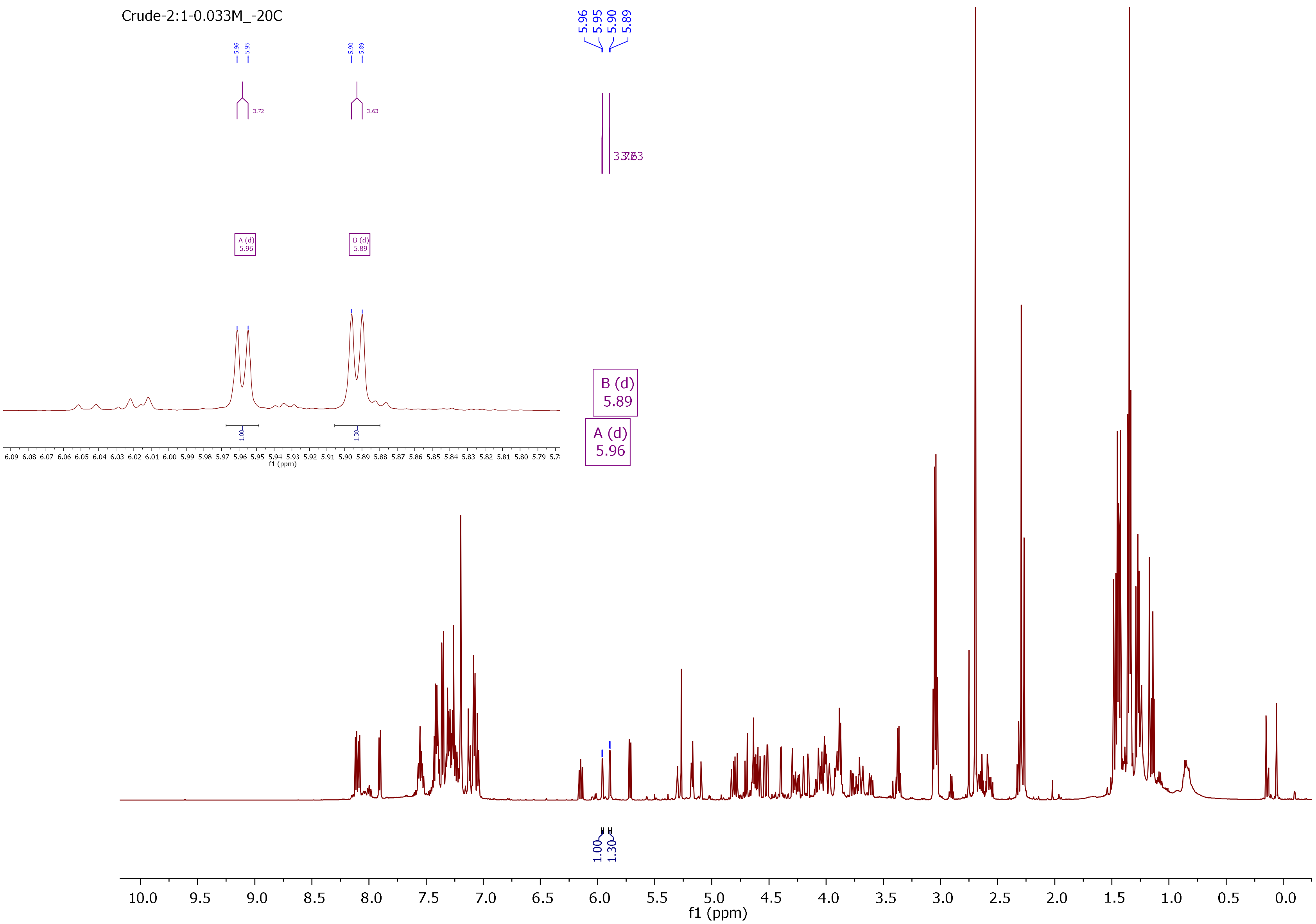

Crude ^{1}H NMR (600 MHz, $CDCl_3$) spectrum of **29** (Donor:Acceptor 1:0.5, 0.033 M, -20 ºC, Set-2)

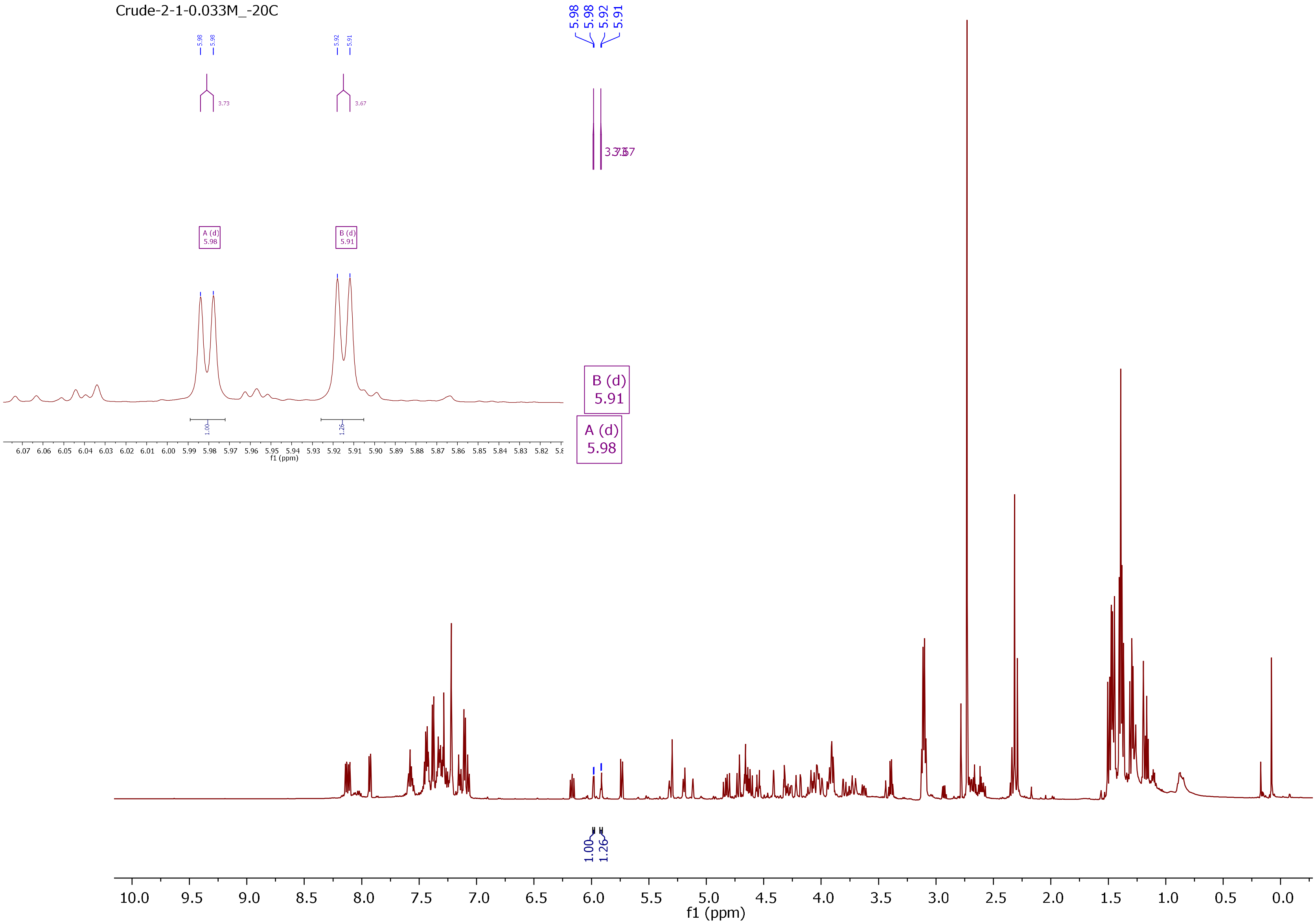

Crude ^{1}H NMR (600 MHz, $CDCl_3$) spectrum of **29** (Donor:Acceptor 1:1, 0.033 M, -20 ºC, Set-1)

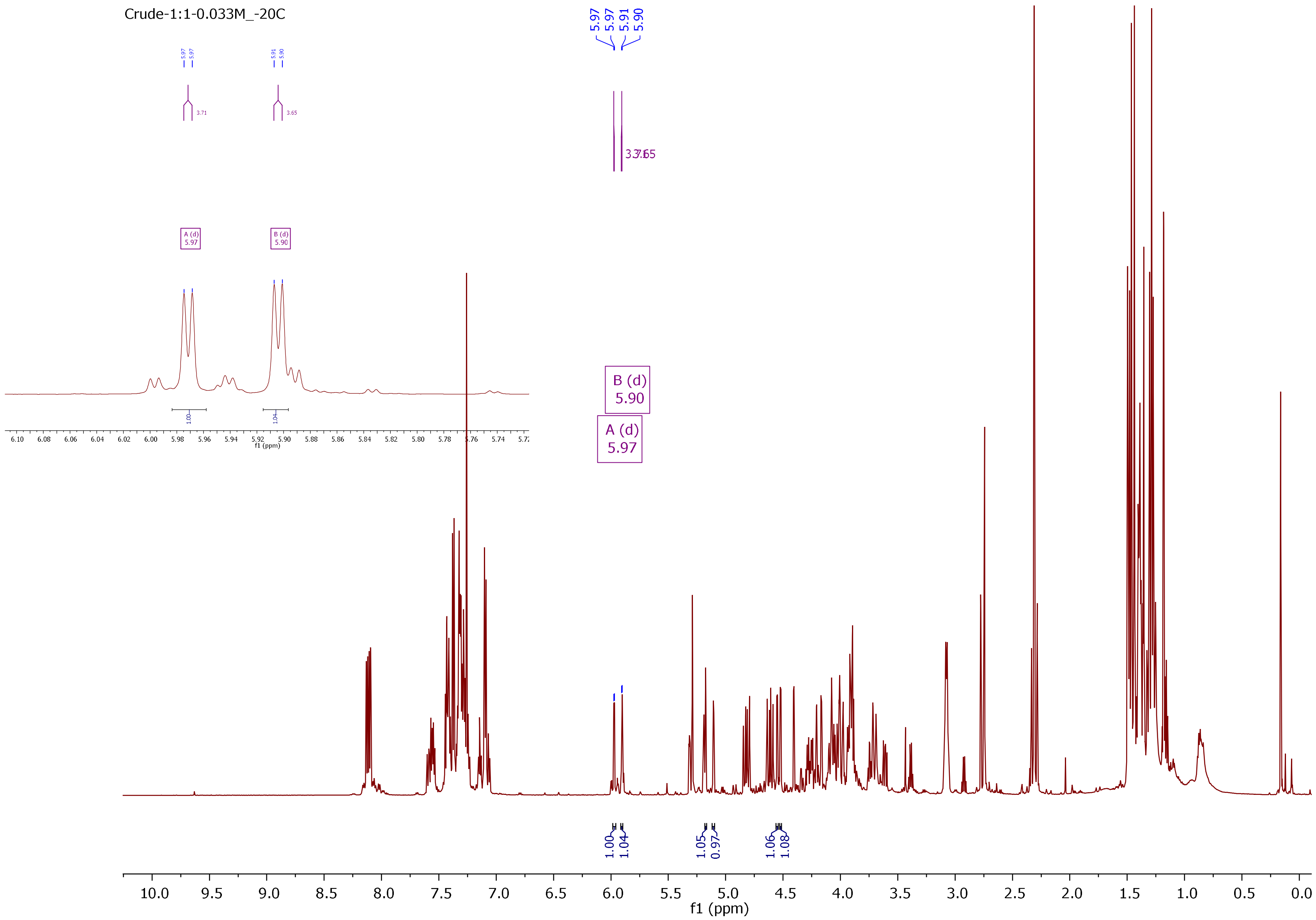

Crude ^{1}H NMR (600 MHz, $CDCl_3$) spectrum of **29** (Donor:Acceptor 1:1, 0.033 M, -20 ºC, Set-2)

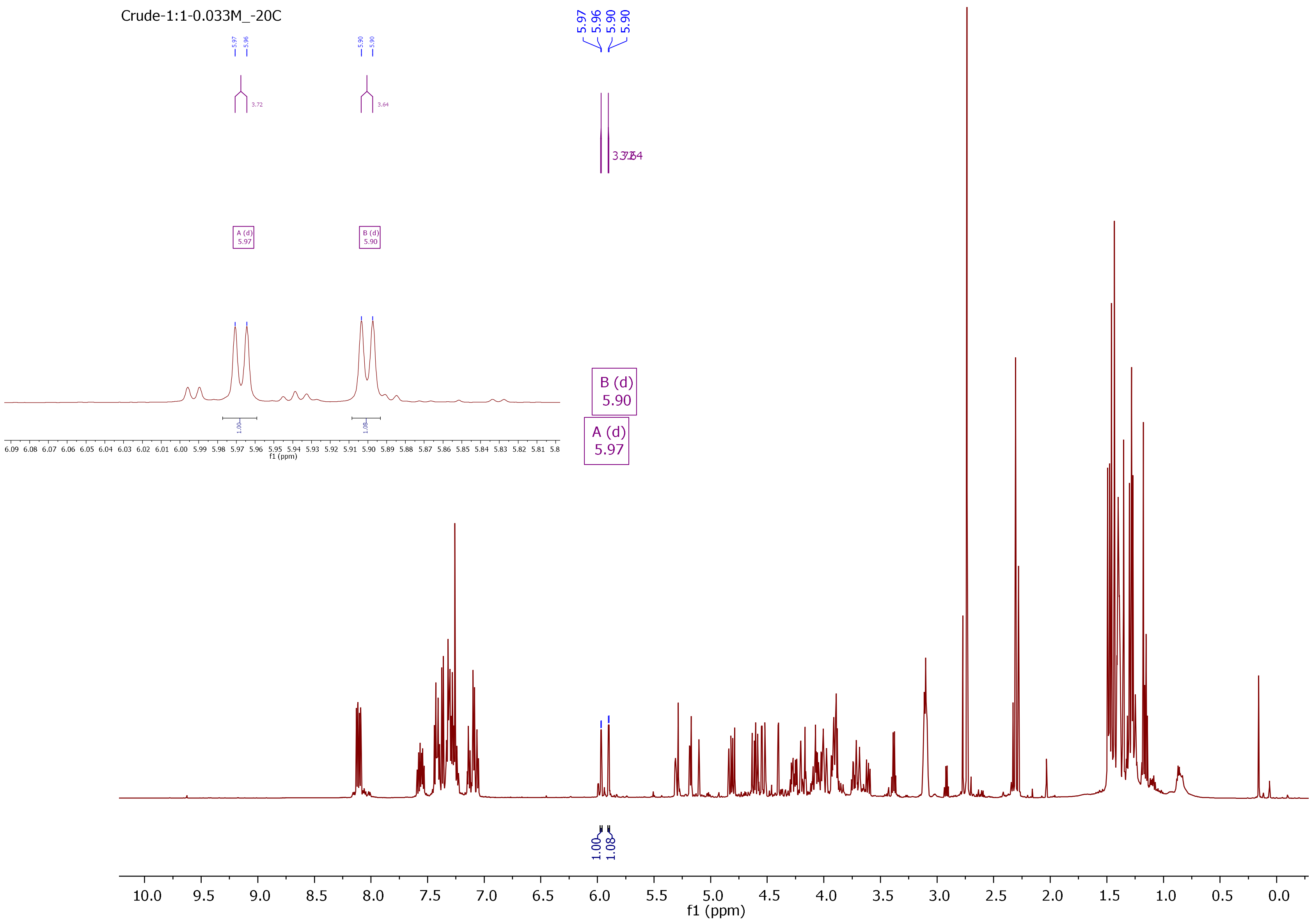

Crude ^{1}H NMR (600 MHz, $CDCl_3$) spectrum of **29** (Donor:Acceptor 1:0.25, 0.2 M, -20 ºC, Set-1)

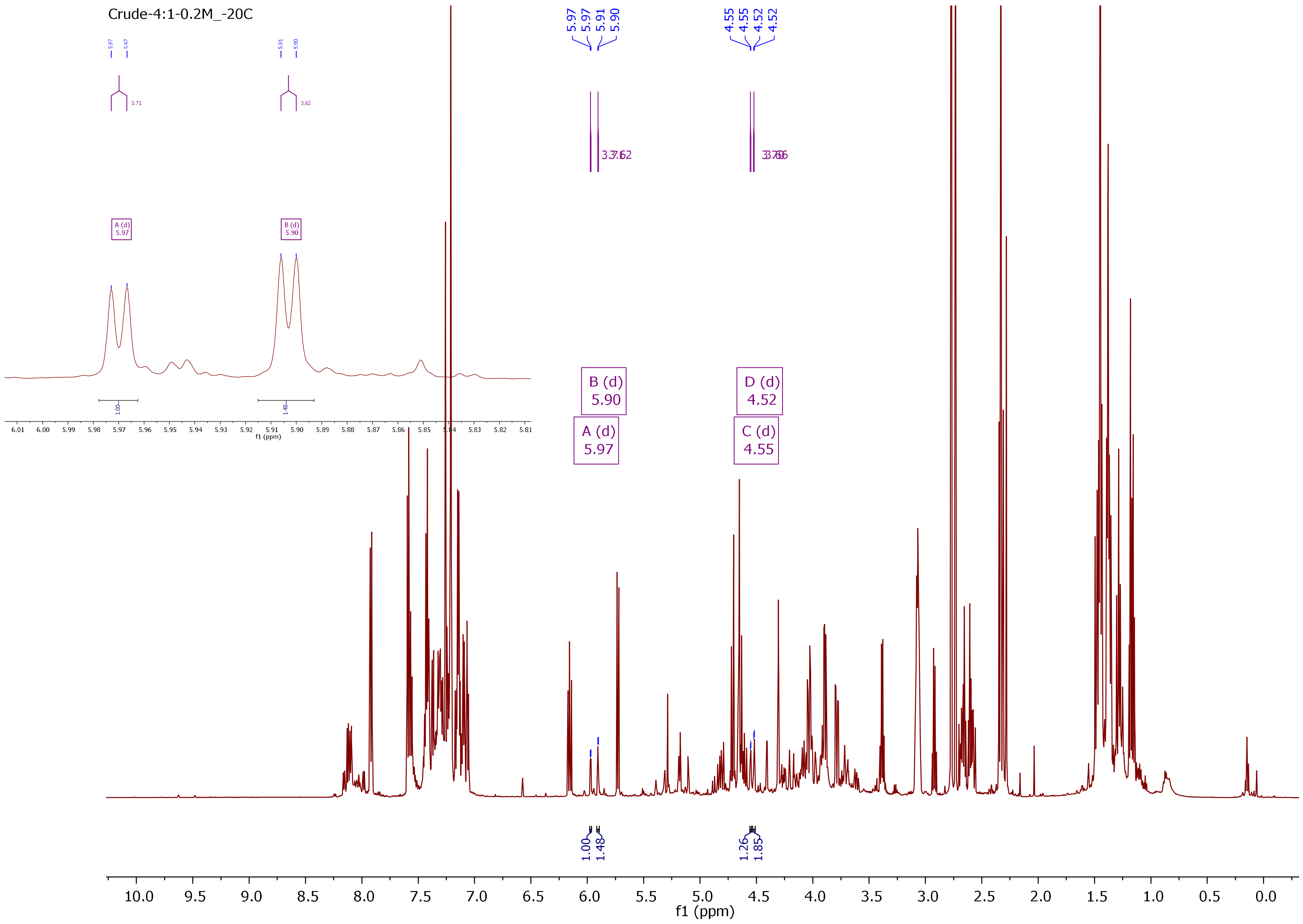

Crude ^{1}H NMR (600 MHz, $CDCl_3$) spectrum of **29** (Donor:Acceptor 1:0.25, 0.2 M, -20 ºC, Set-2)

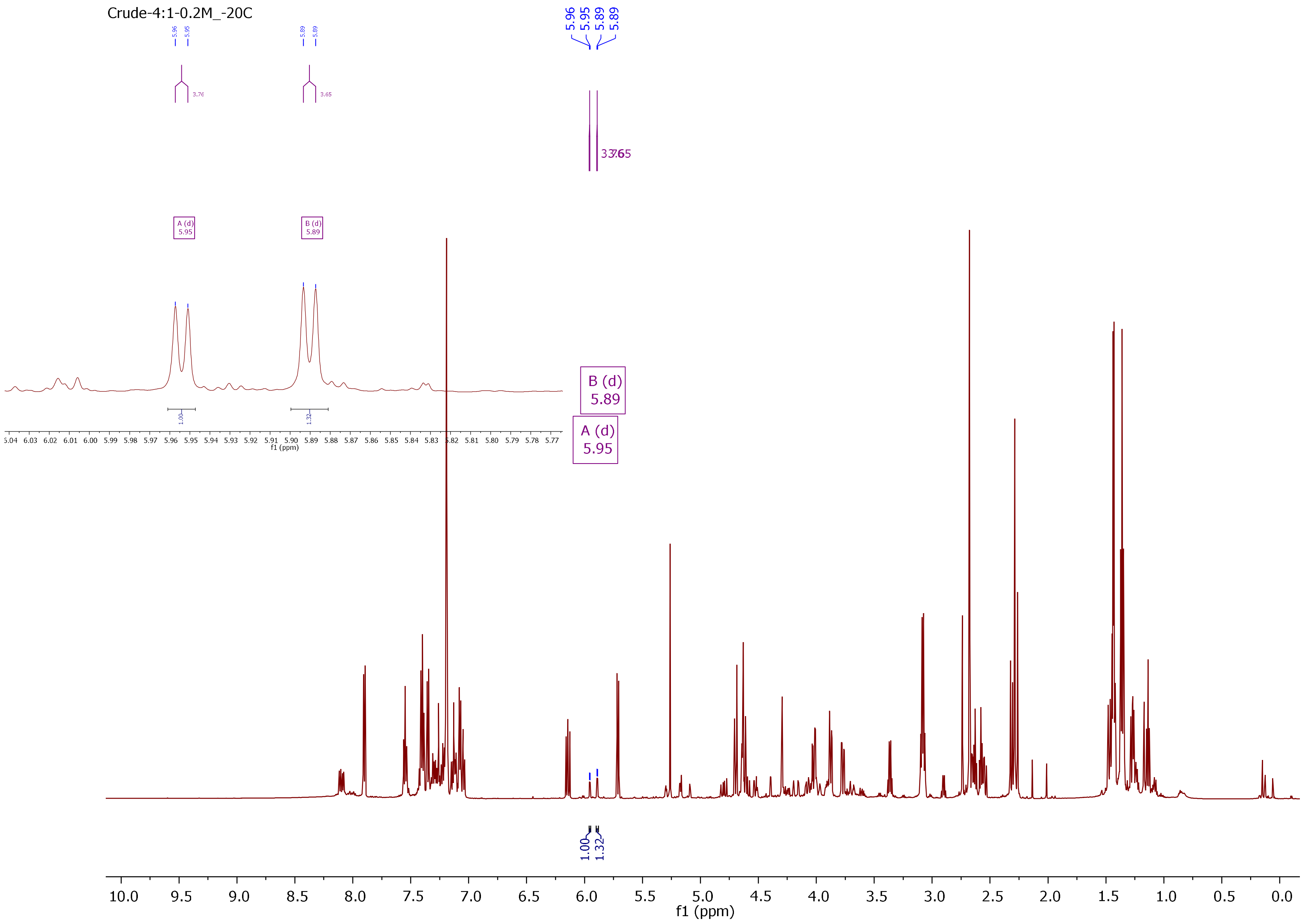

Crude ^{1}H NMR (600 MHz, $CDCl_3$) spectrum of **29** (Donor:Acceptor 1:0.5, 0.2 M, -20 ºC, Set-1)

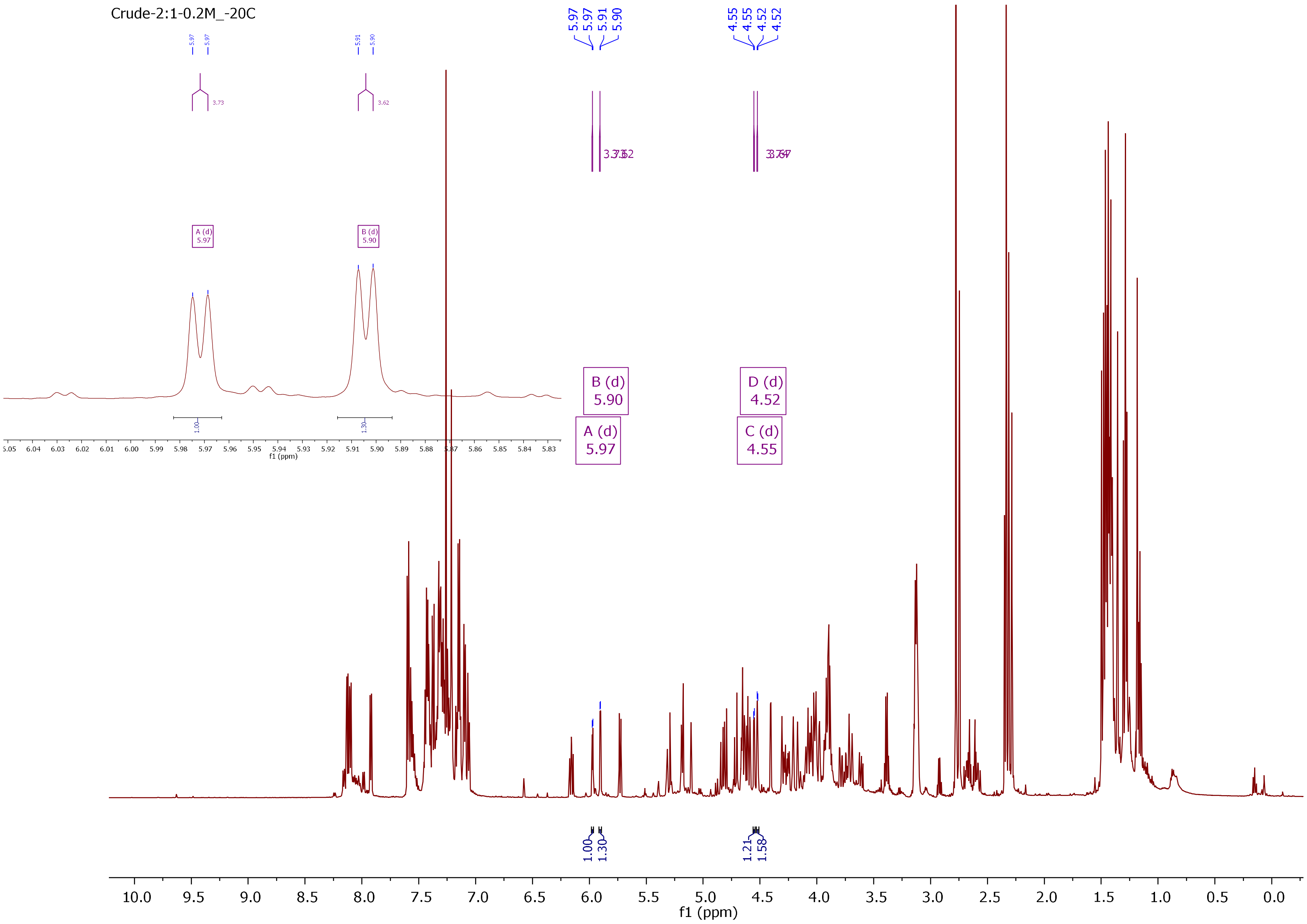

Crude ^{1}H NMR (600 MHz, $CDCl_3$) spectrum of **29** (Donor:Acceptor 1:0.5, 0.2 M, -20 ºC, Set-2)

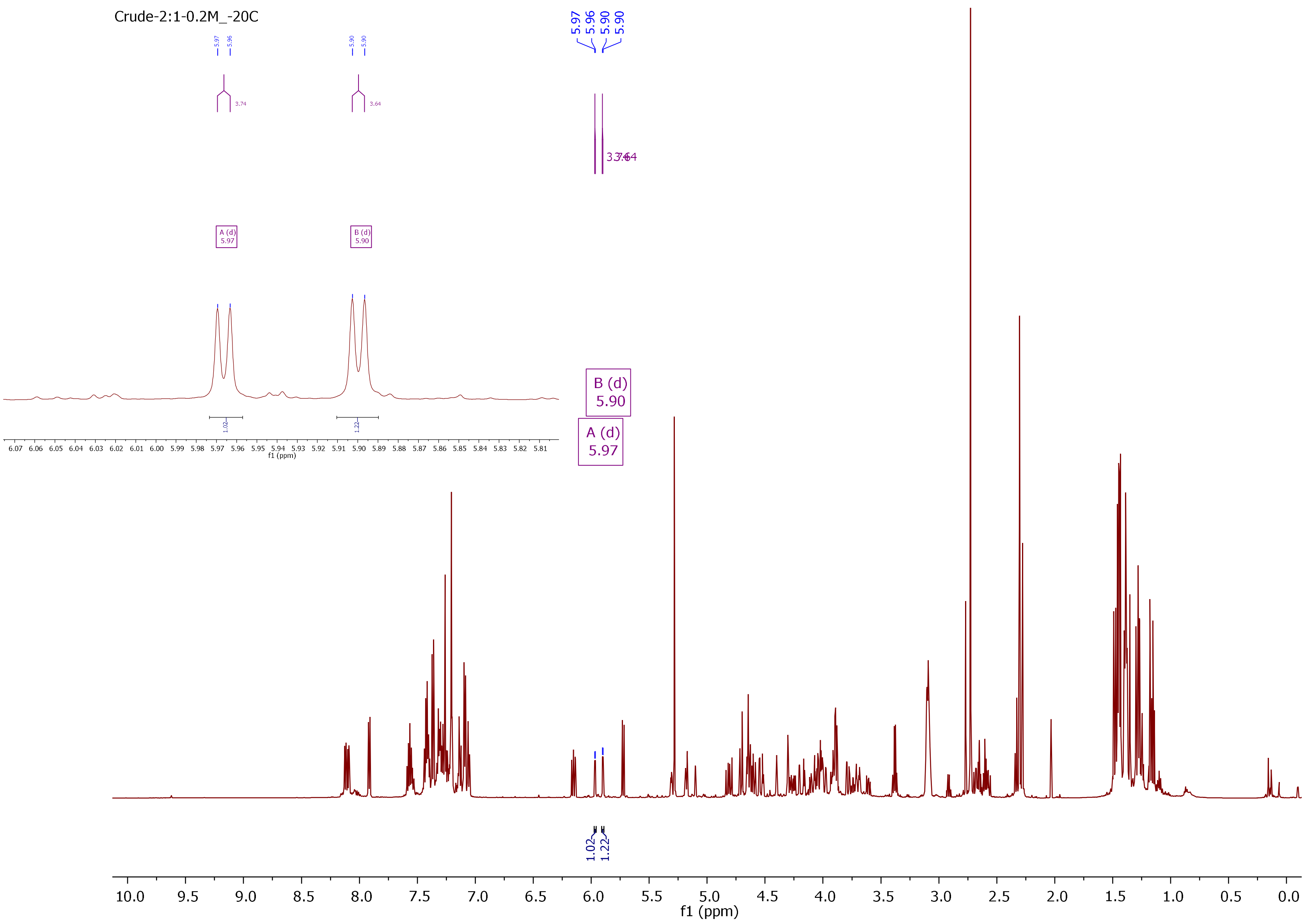

Crude ^{1}H NMR (600 MHz, $CDCl_3$) spectrum of **29** (Donor:Acceptor 1:0.83 0.2 M, -20 ºC, Set-1)

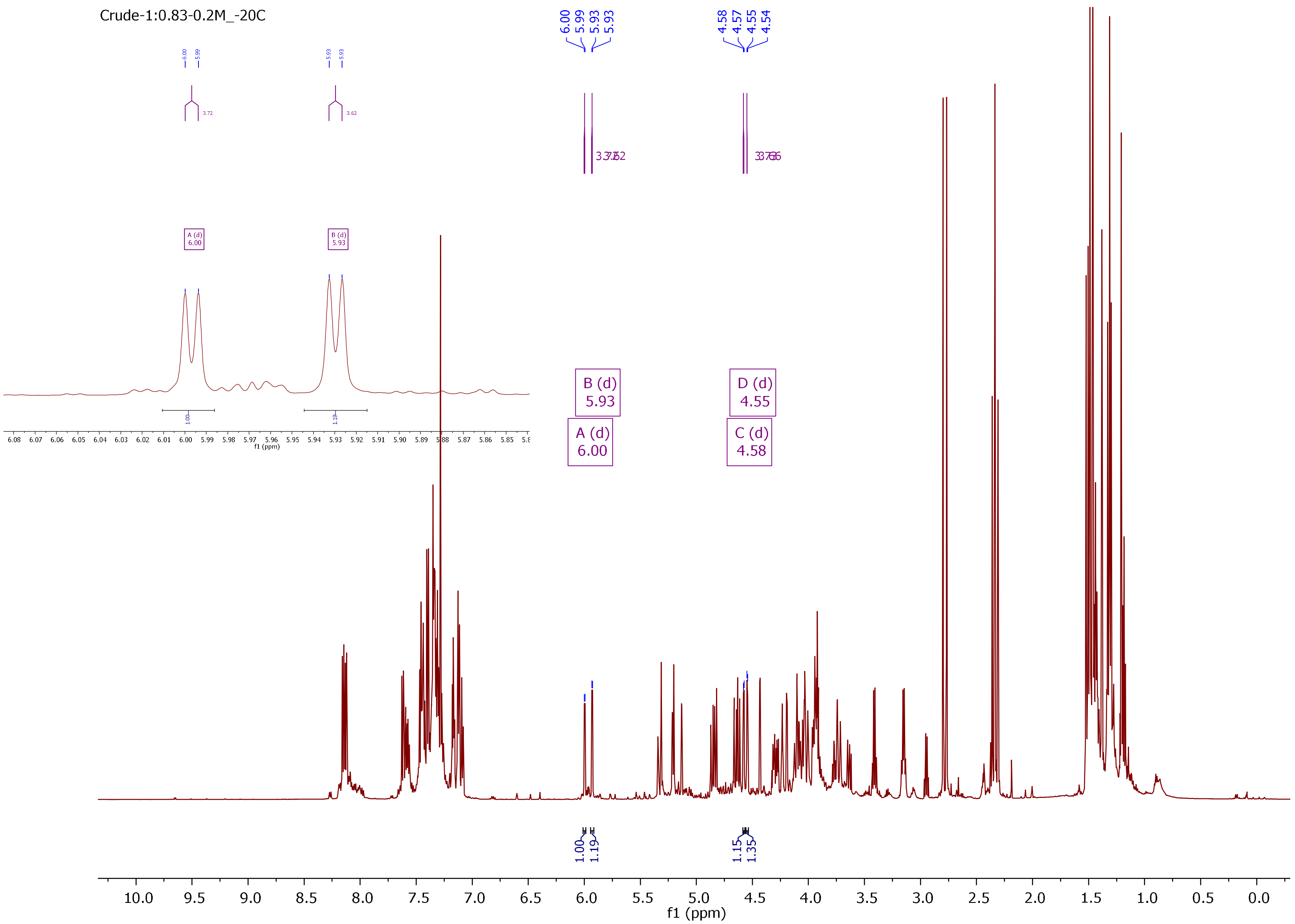

Crude ^{1}H NMR (600 MHz, $CDCl_3$) spectrum of **29** (Donor:Acceptor 1:0.83 0.2 M, -20 ºC, Set-2)

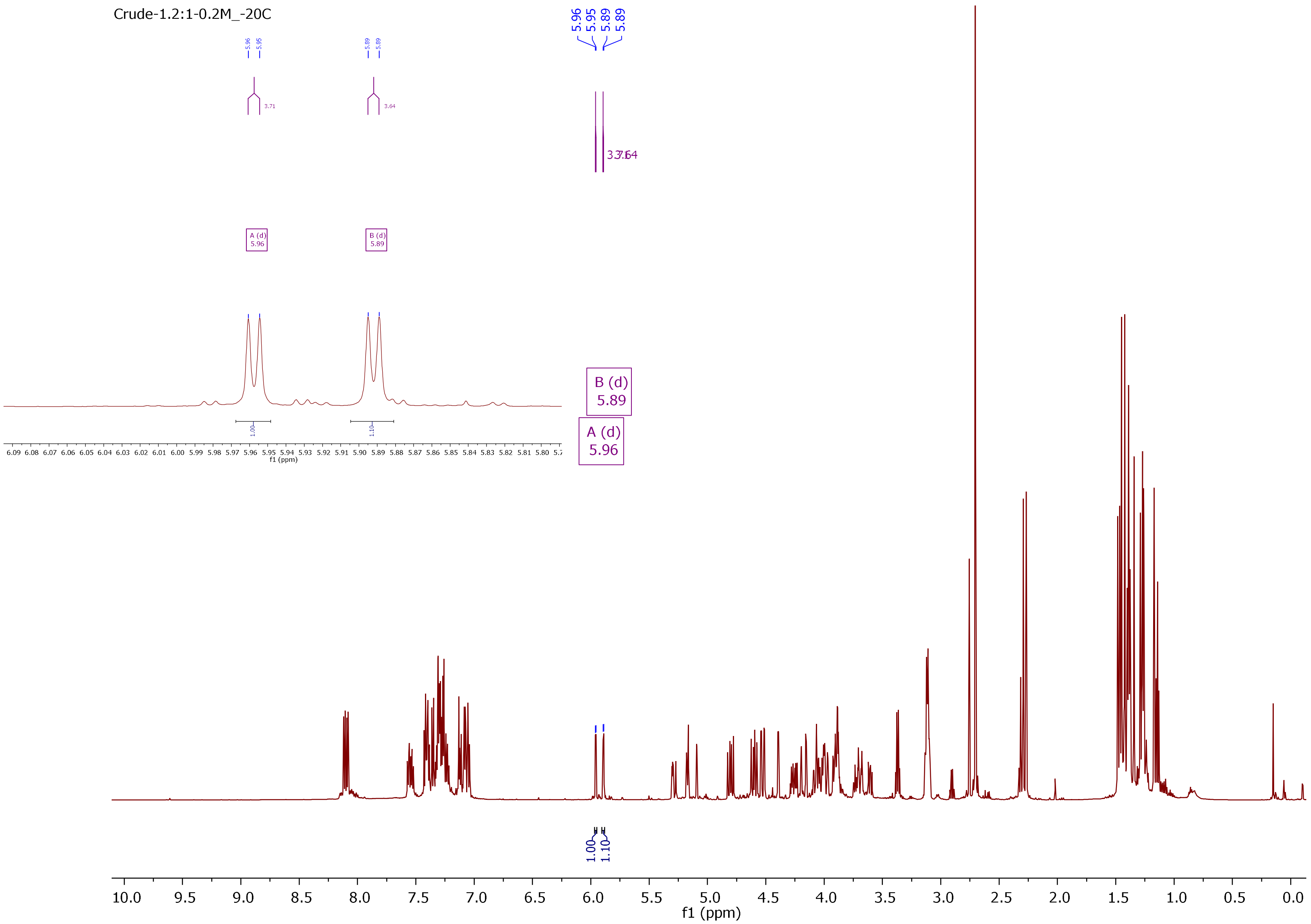

Crude ^{1}H NMR (600 MHz, $CDCl_3$) spectrum of **29** (Donor:Acceptor 1:1, 0.2 M, -20 ºC, Set-1)

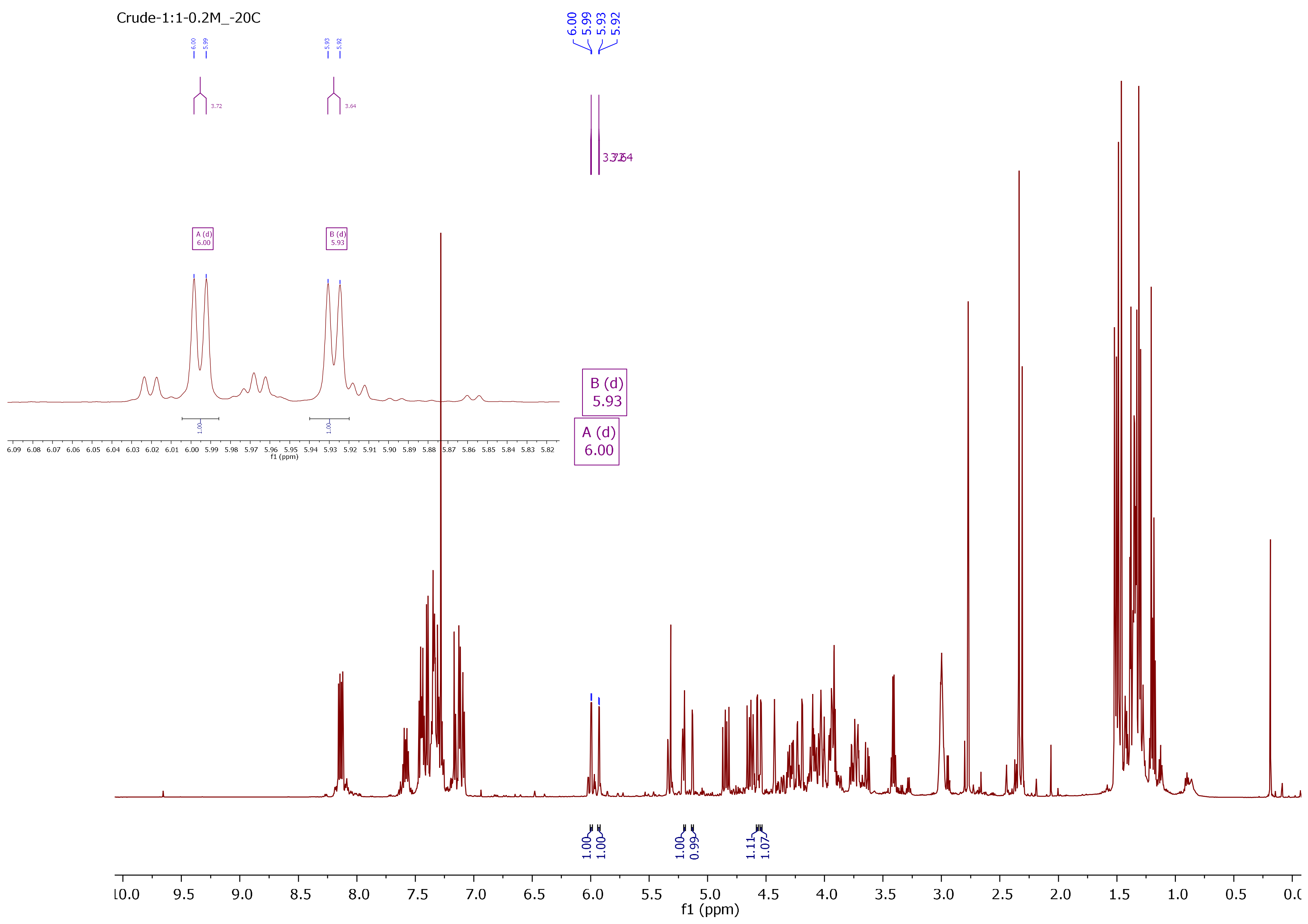

Crude ^{1}H NMR (600 MHz, $CDCl_3$) spectrum of **29** (Donor:Acceptor 1:1, 0.2 M, -20 ºC, Set-2)

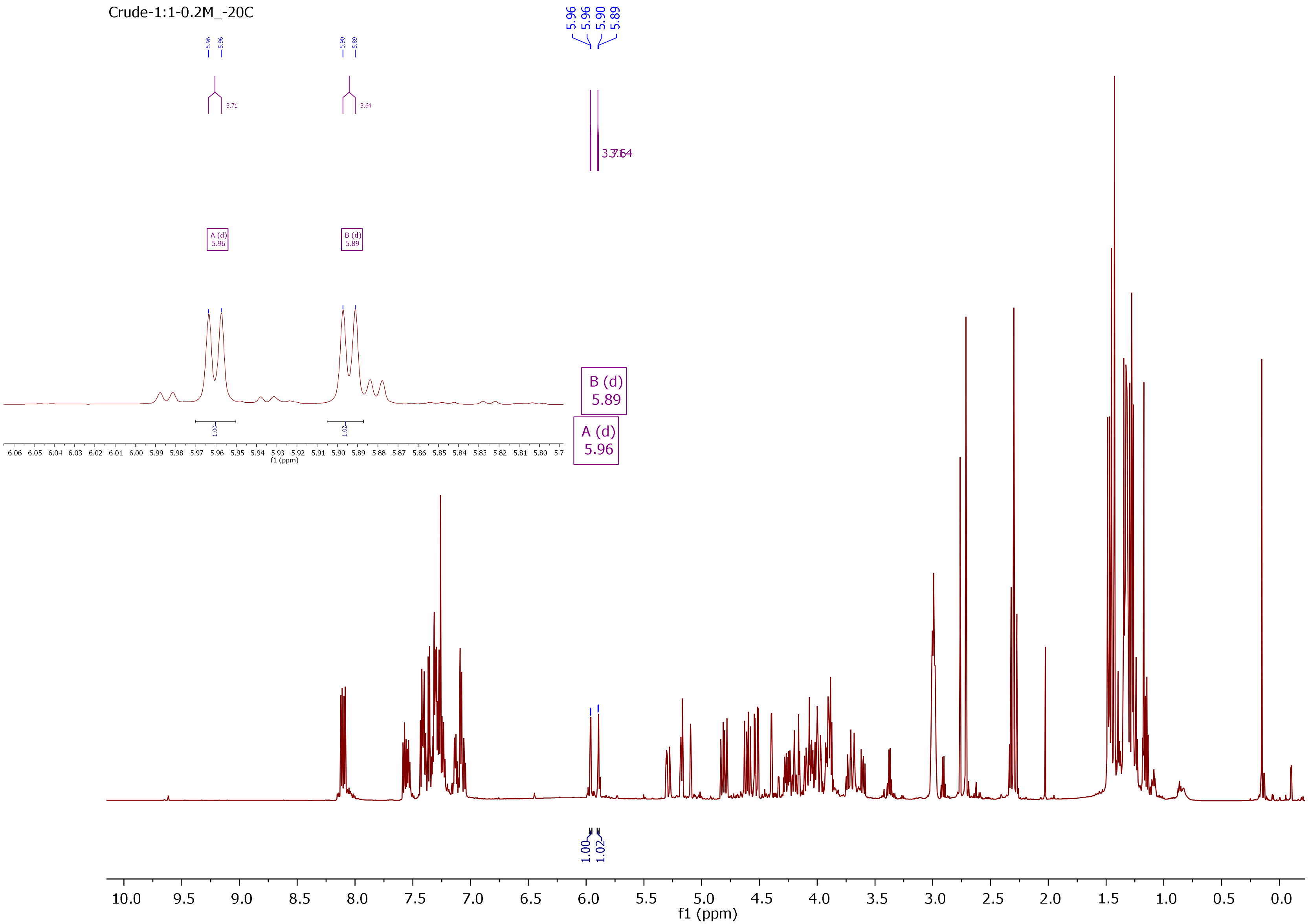

Crude ^{1}H NMR (600 MHz, $CDCl_3$) spectrum of **29** (Donor:Acceptor 1:2, 0.2 M, -20 ºC, Set-1)

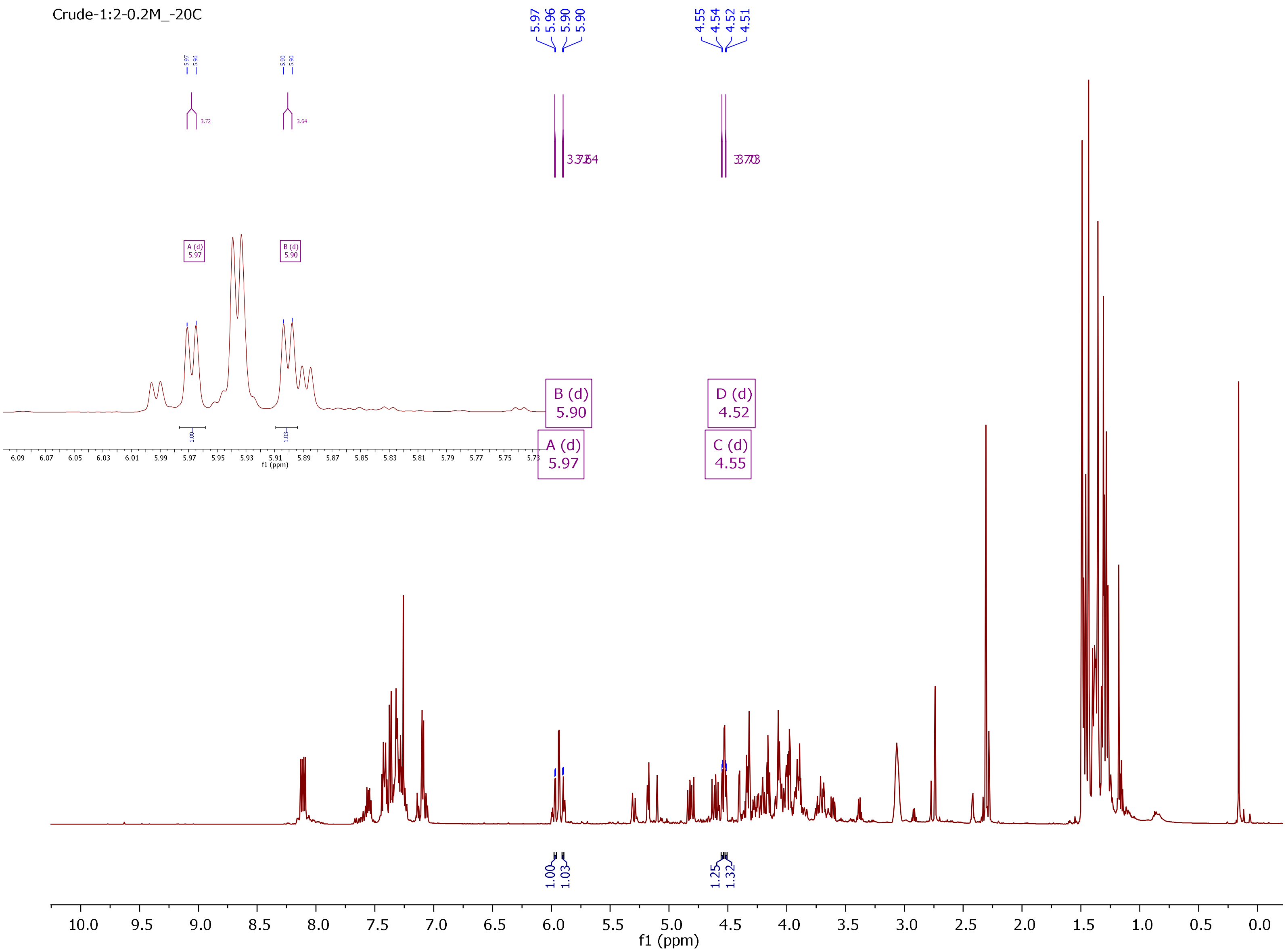

Crude ^{1}H NMR (600 MHz, $CDCl_3$) spectrum of **29** (Donor:Acceptor 1:2, 0.2 M, -20 ºC, Set-2)

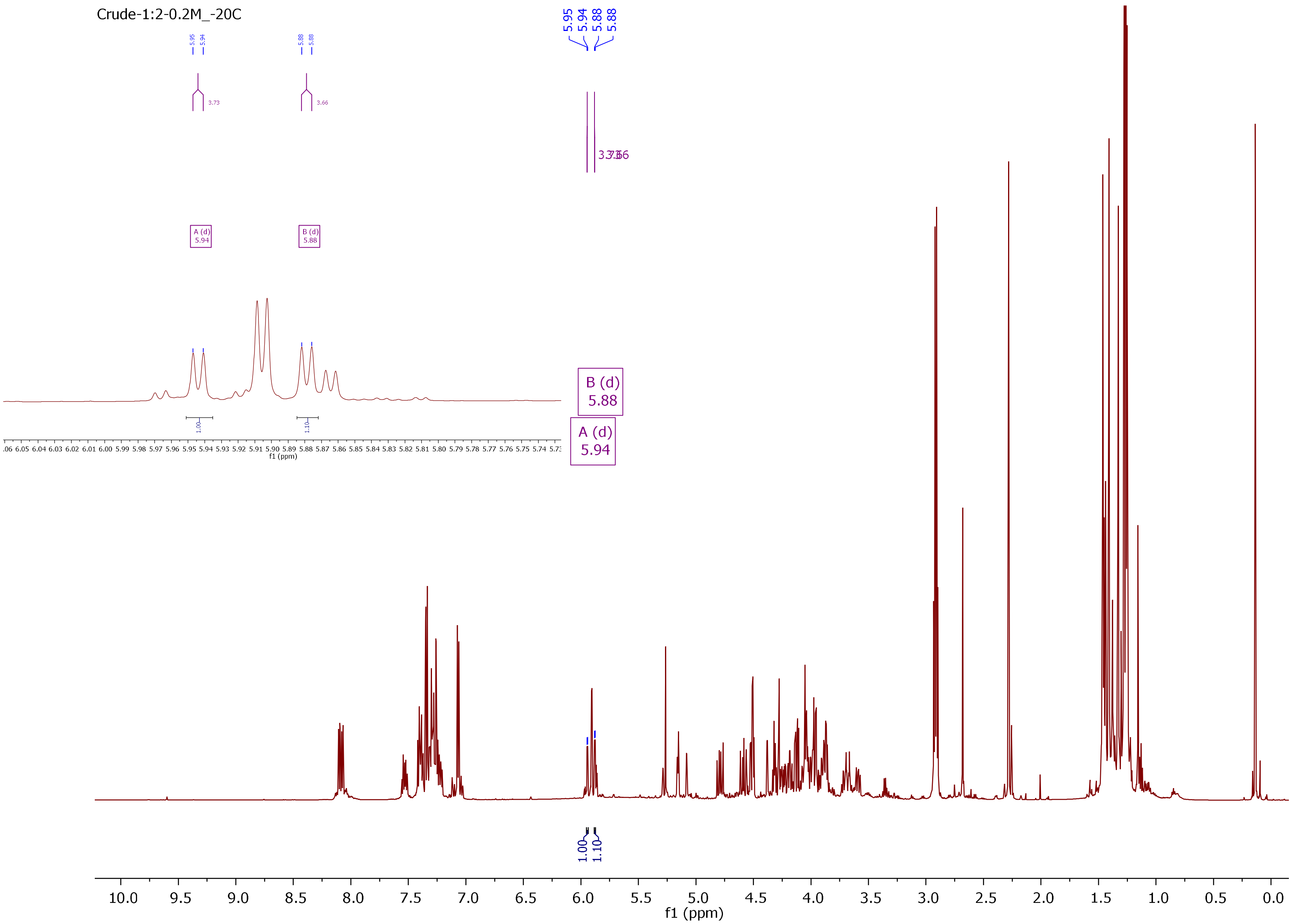

Crude ^{1}H NMR (600 MHz, $CDCl_3$) spectrum of **29** (Donor:Acceptor 1:0.5, 0.3 M, -20 ºC, Set-1)

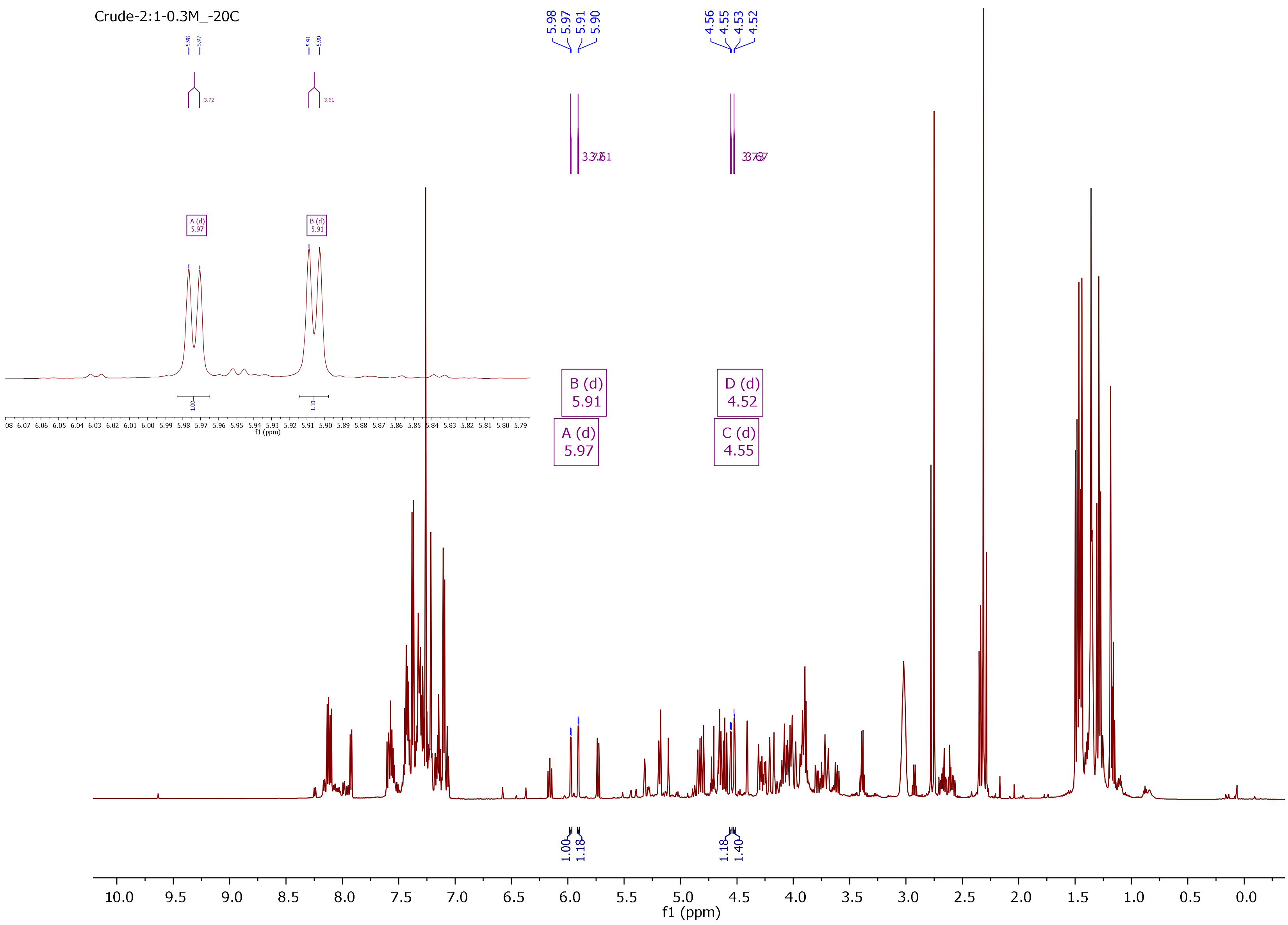

Crude ^{1}H NMR (600 MHz, $CDCl_3$) spectrum of **29** (Donor:Acceptor 1:0.5, 0.3 M, -20 ºC, Set-2)

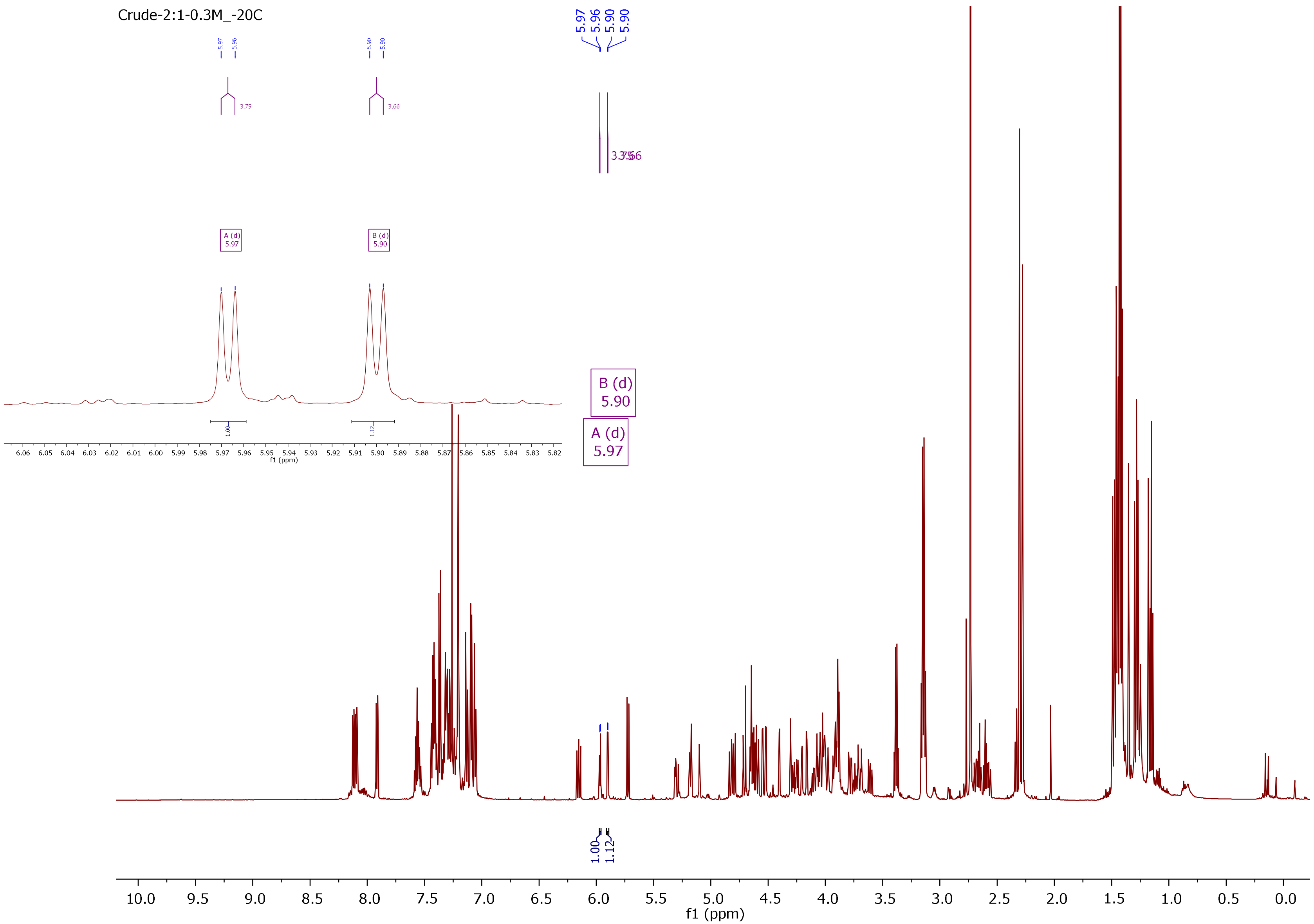

Crude ^{1}H NMR (600 MHz, $CDCl_3$) spectrum of **29** (Donor:Acceptor 1:1, 0.3 M, -20 ºC, Set-1)

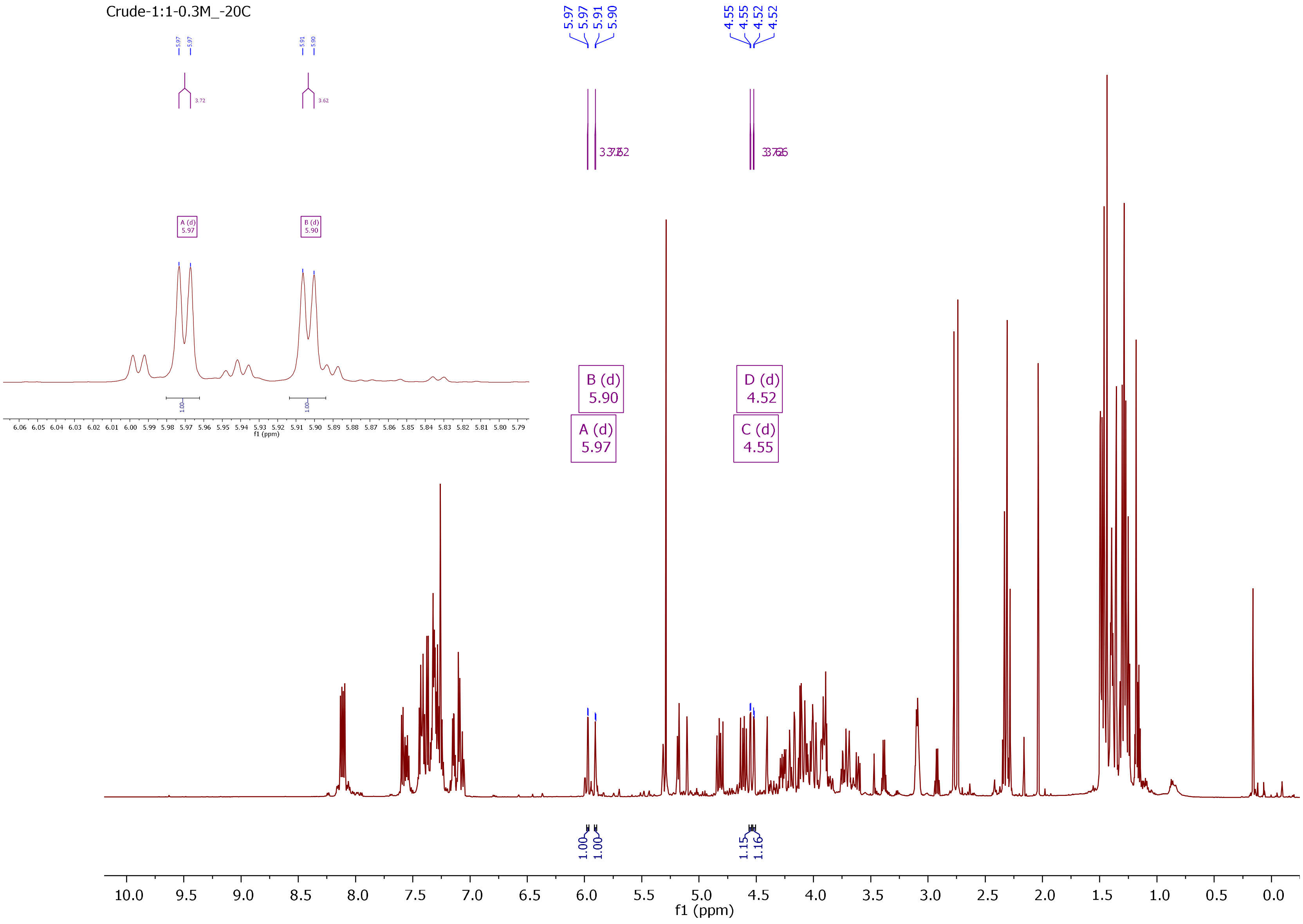

Crude ^{1}H NMR (600 MHz, $CDCl_3$) spectrum of **29** (Donor:Acceptor 1:1, 0.3 M, -20 ºC, Set-2)

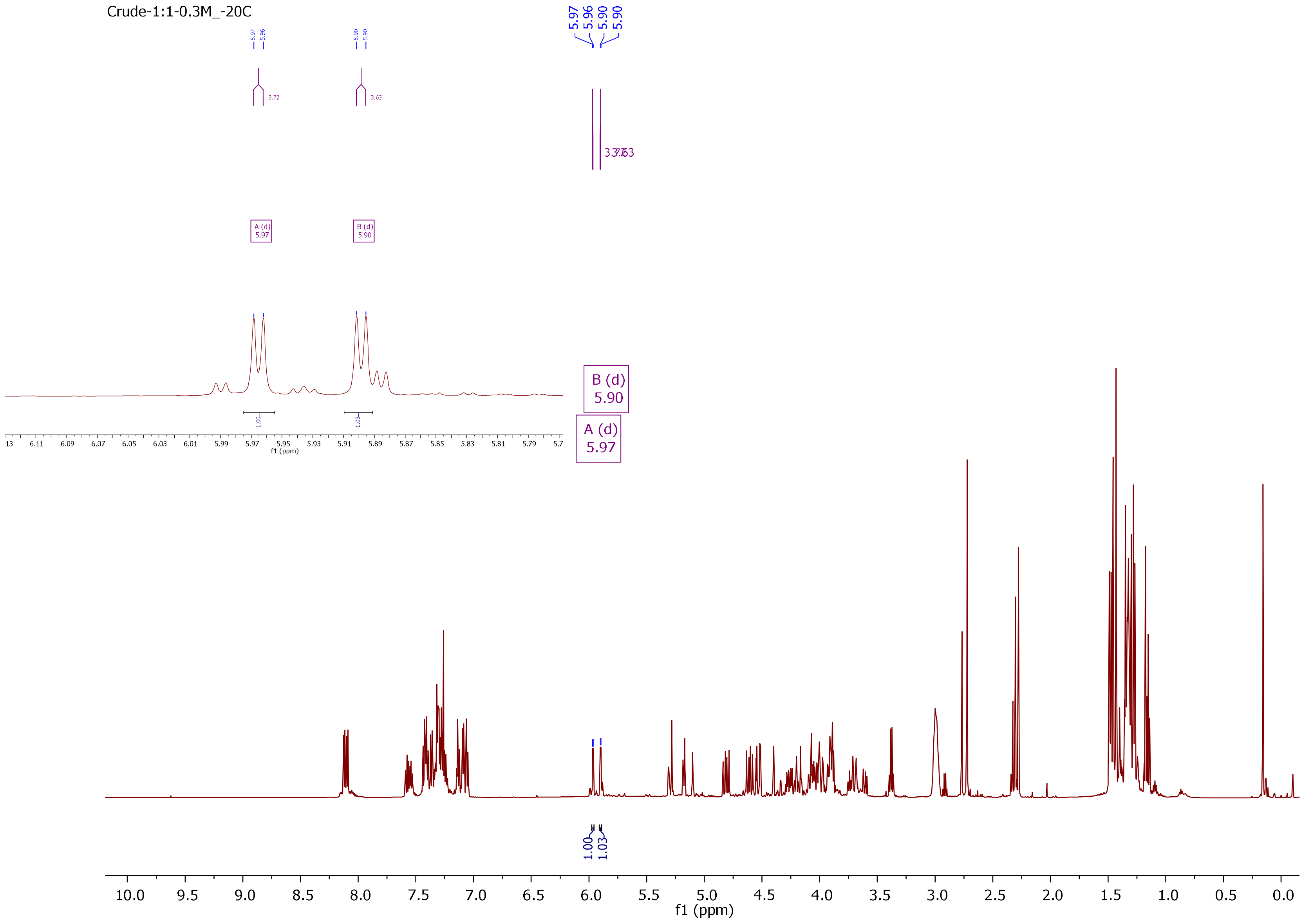

Crude ^{1}H NMR (600 MHz, $CDCl_3$) spectrum of **29** (Donor:Acceptor 1:0.5, 0.033 M, -5 ºC, Set-1)

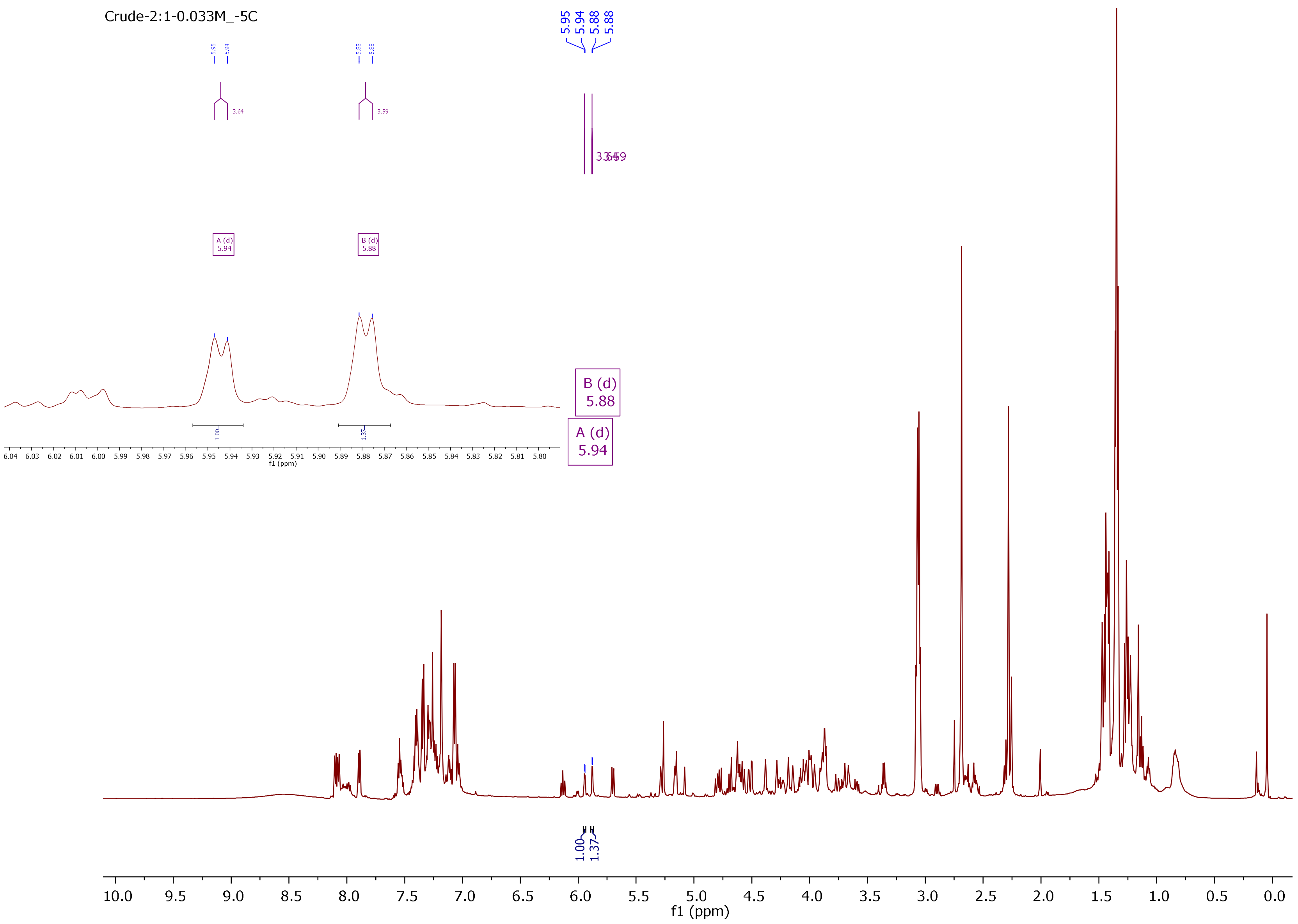

Crude ^{1}H NMR (600 MHz, $CDCl_3$) spectrum of **29** (Donor:Acceptor 1:0.5, 0.033 M, -5 ºC, Set-2)

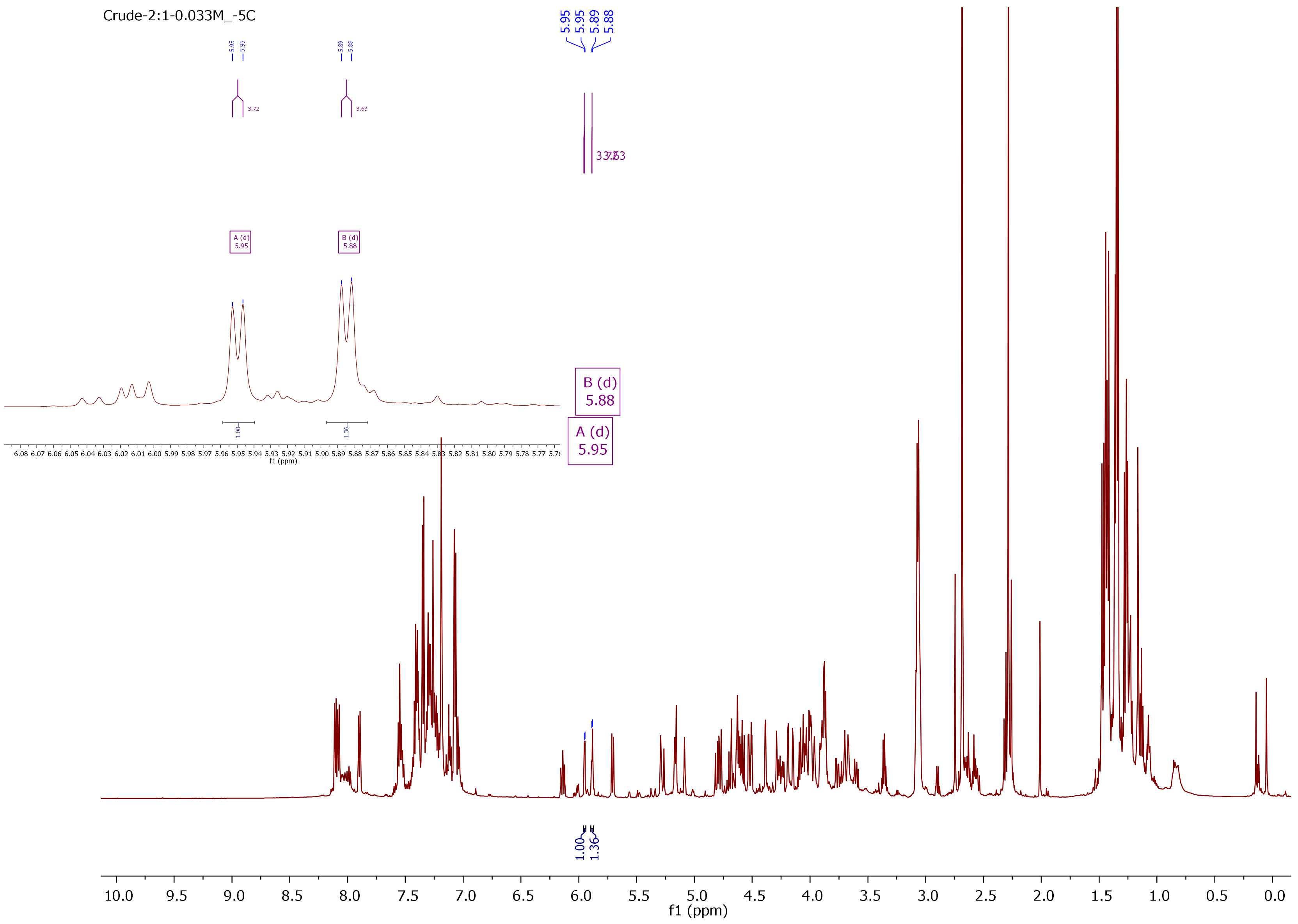

Crude ^{1}H NMR (600 MHz, $CDCl_3$) spectrum of **29** (Donor:Acceptor 1:1, 0.033 M, -5 ºC, Set-1)

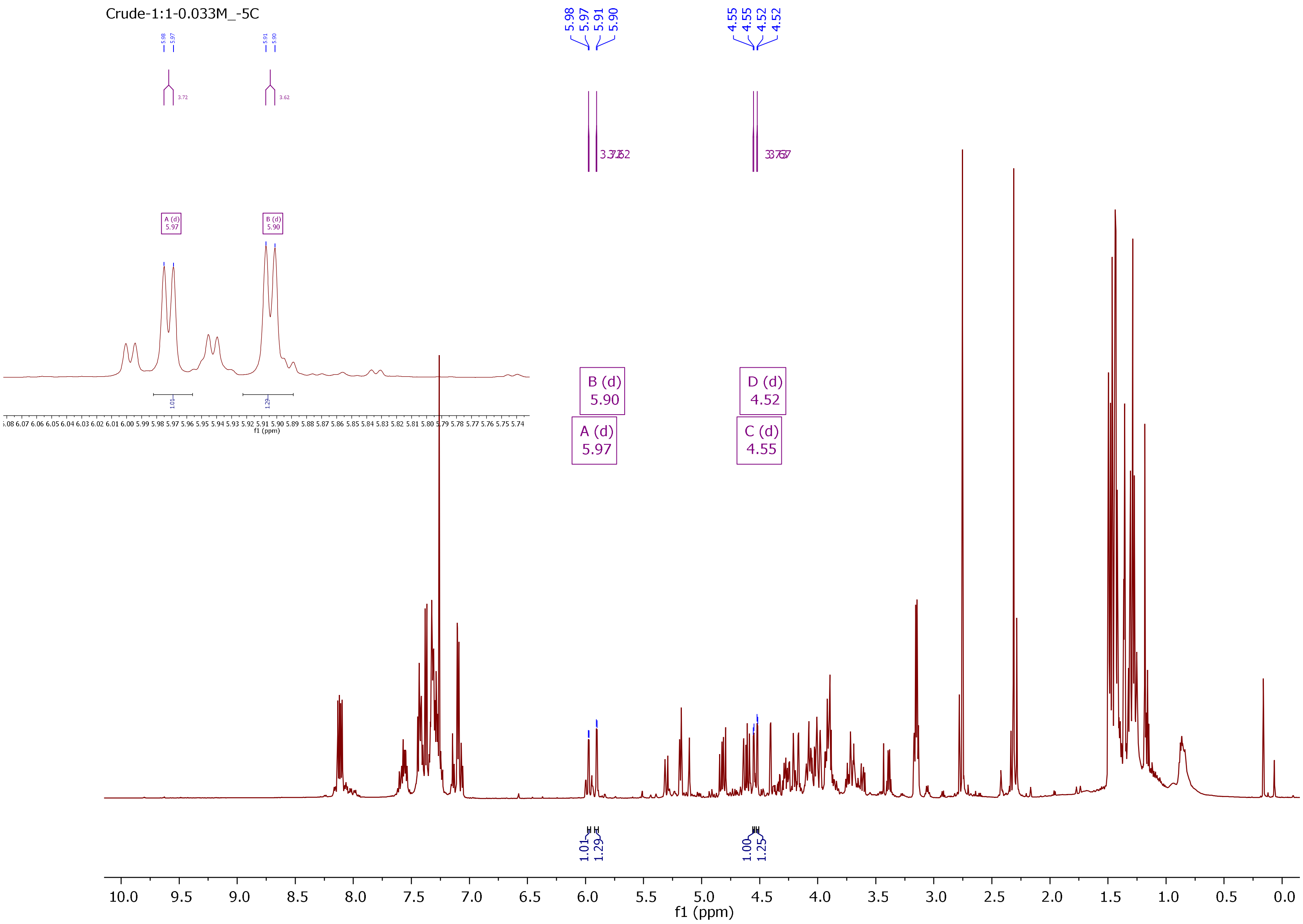

Crude ^{1}H NMR (600 MHz, $CDCl_3$) spectrum of **29** (Donor:Acceptor 1:1, 0.033 M, -5 ºC, Set-2)

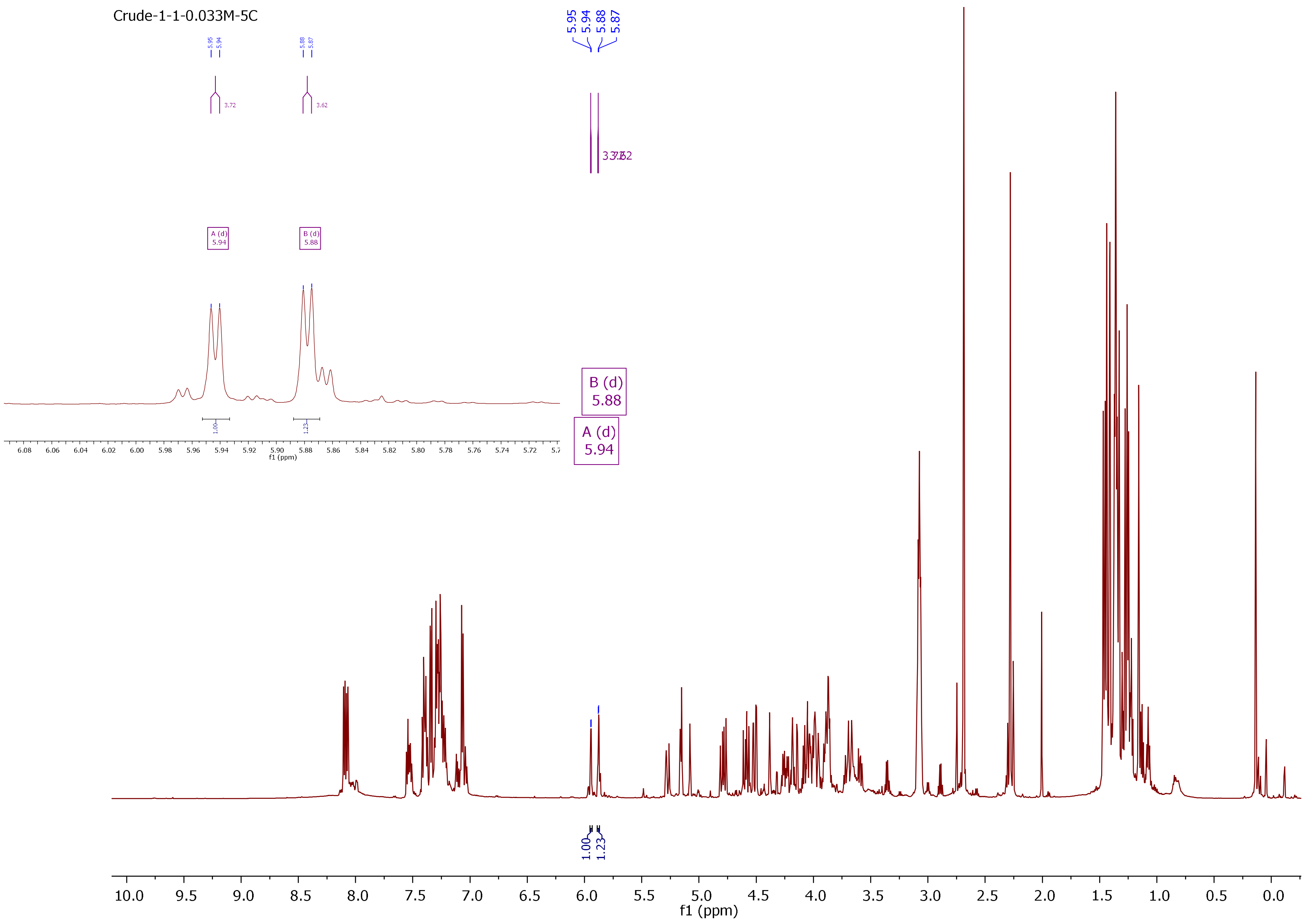

Crude ^{1}H NMR (600 MHz, $CDCl_3$) spectrum of **29** (Donor:Acceptor 1:0.5, 0.2 M, -5 ºC, Set-1)

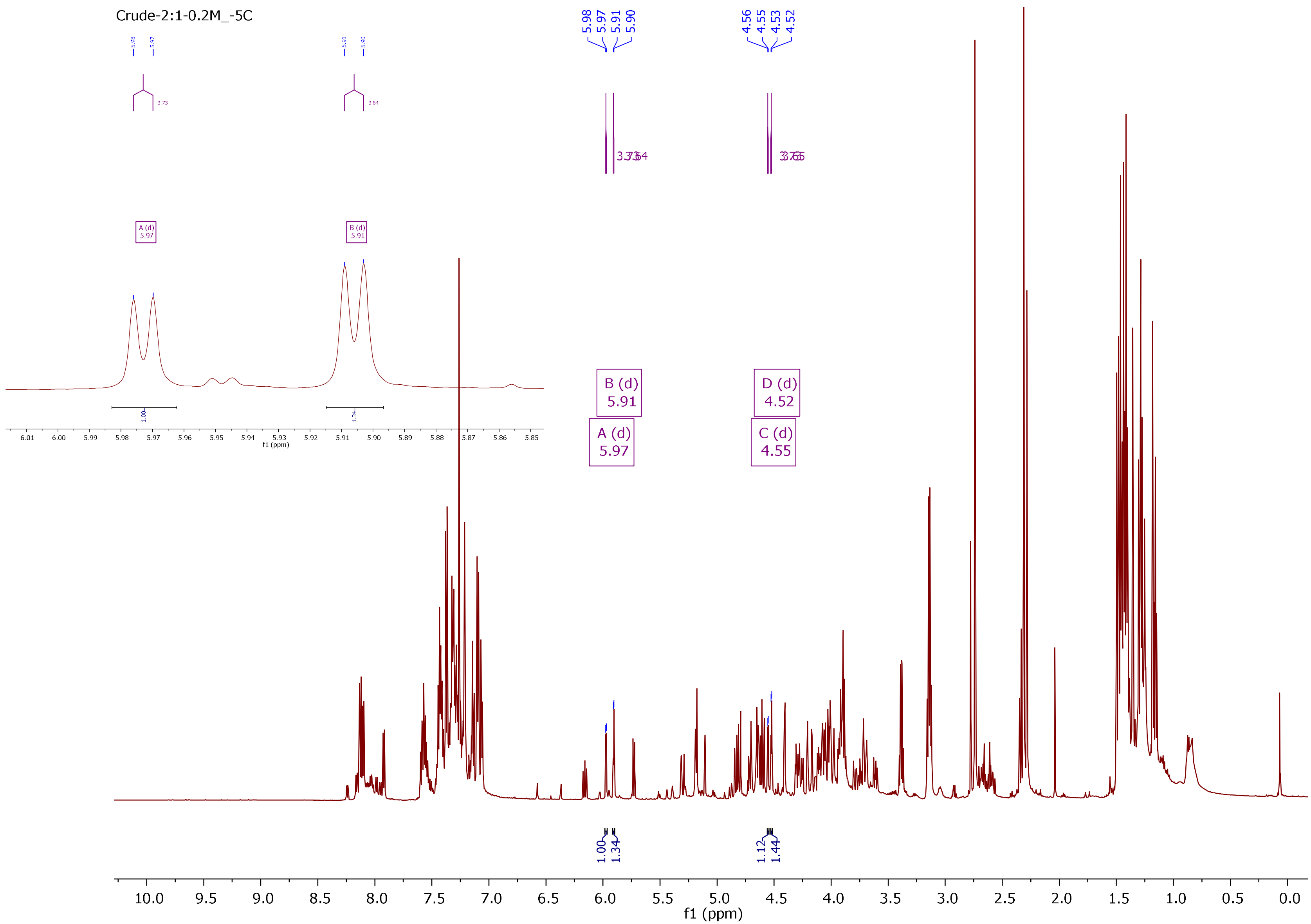

Crude ^{1}H NMR (600 MHz, $CDCl_3$) spectrum of **29** (Donor:Acceptor 1:0.5, 0.2 M, -5 ºC, Set-2)

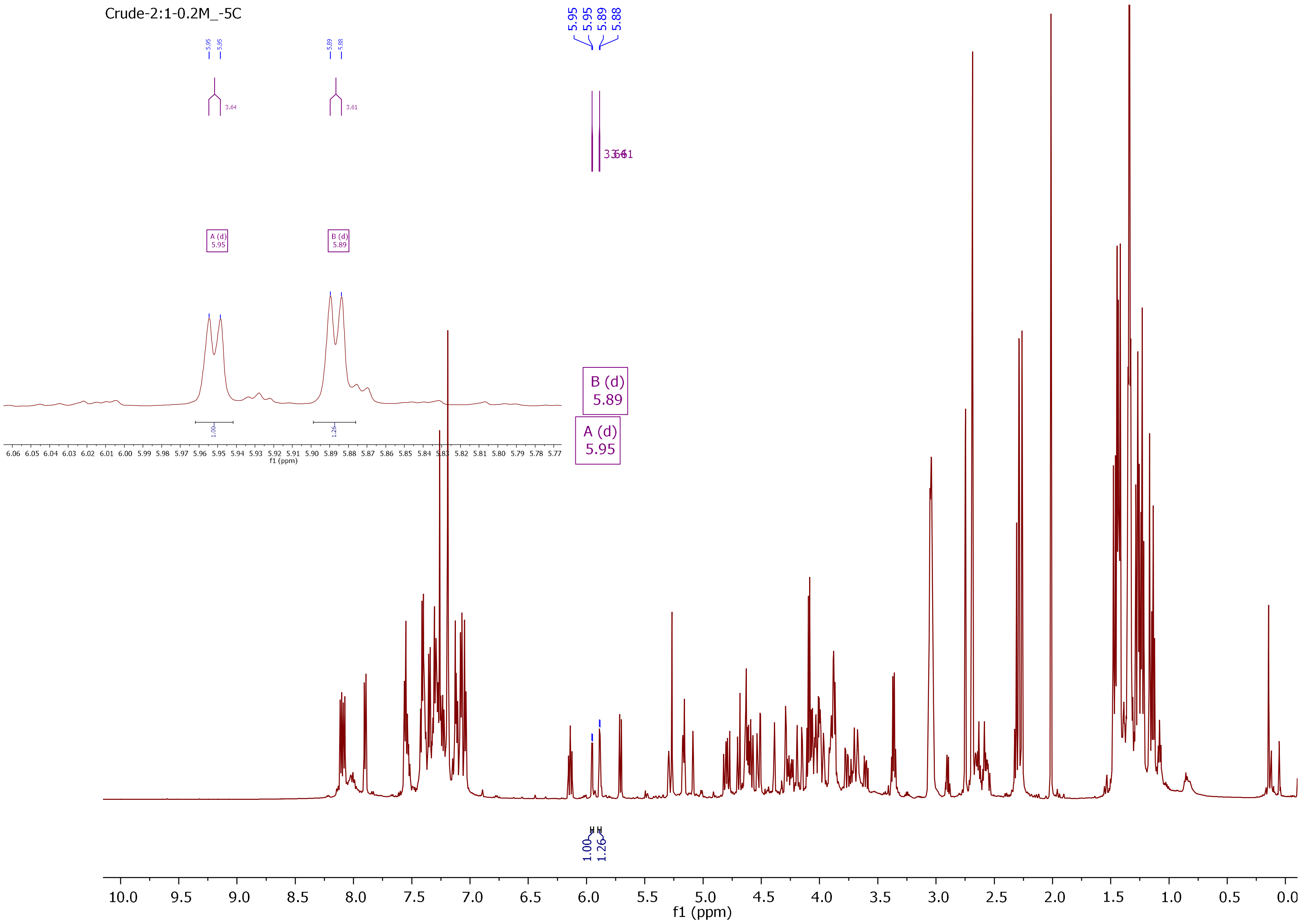

Crude ^{1}H NMR (600 MHz, $CDCl_3$) spectrum of **29** (Donor:Acceptor 1:1, 0.2 M, -5 ºC, Set-1)

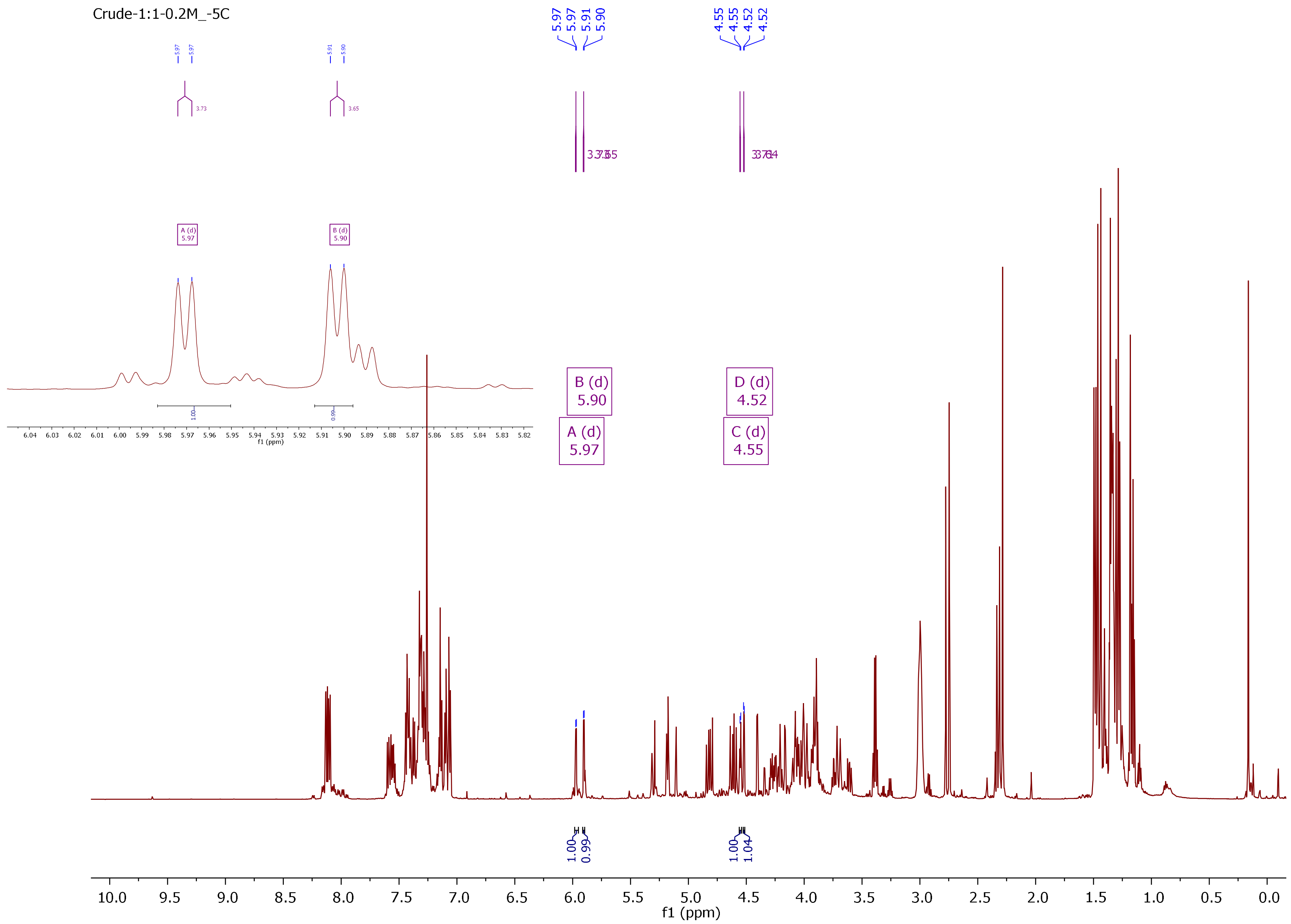

Crude ^{1}H NMR (600 MHz, $CDCl_3$) spectrum of **29** (Donor:Acceptor 1:1, 0.2 M, -5 ºC, Set-2)

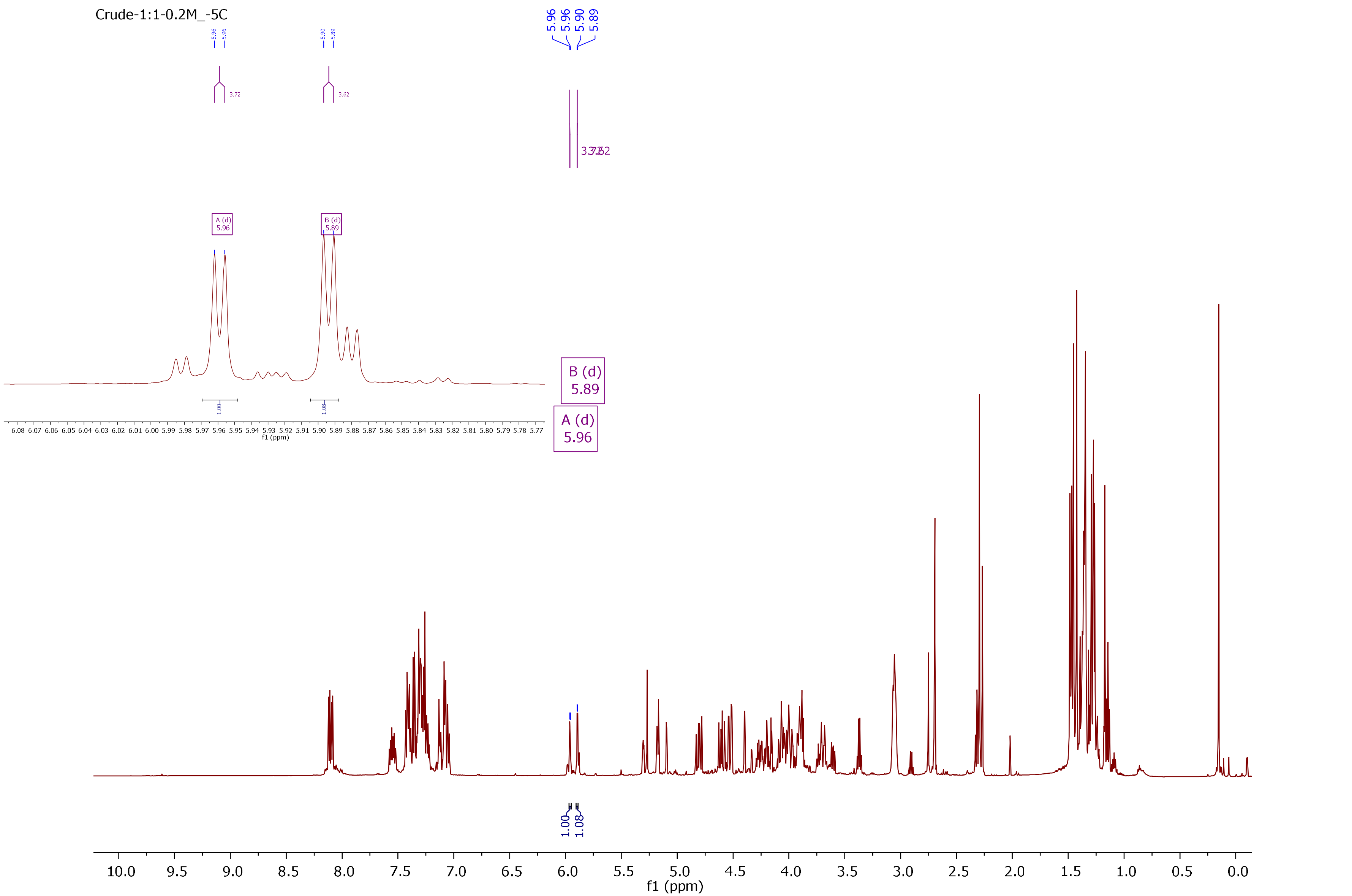

Crude ^{1}H NMR (600 MHz, $CDCl_3$) spectrum of **29** (Donor:Acceptor 1:0.5, 0.3 M, -5 ºC, Set-1)

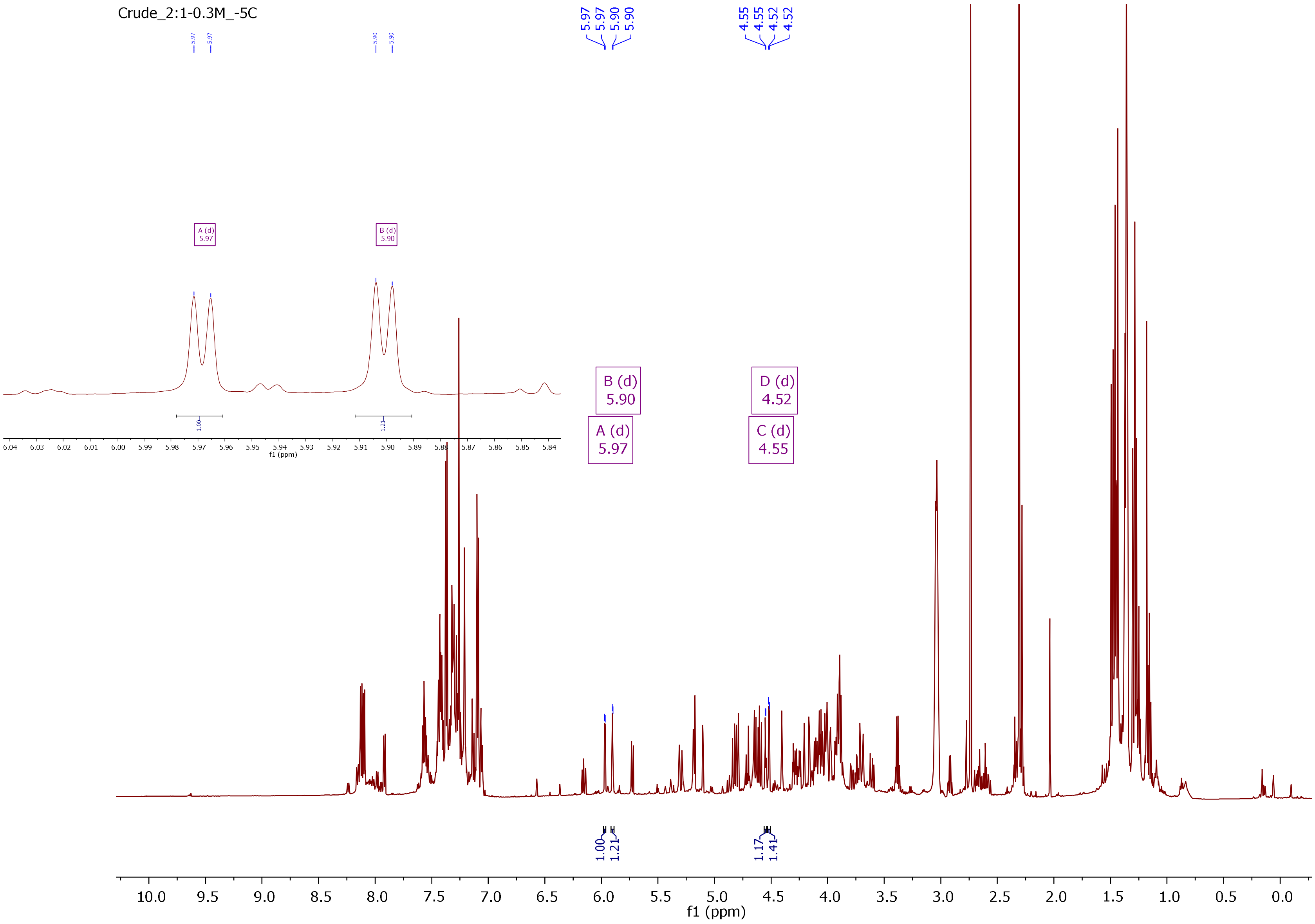

Crude ^{1}H NMR (600 MHz, $CDCl_3$) spectrum of **29** (Donor:Acceptor 1:0.5, 0.3 M, -5 ºC, Set-2)

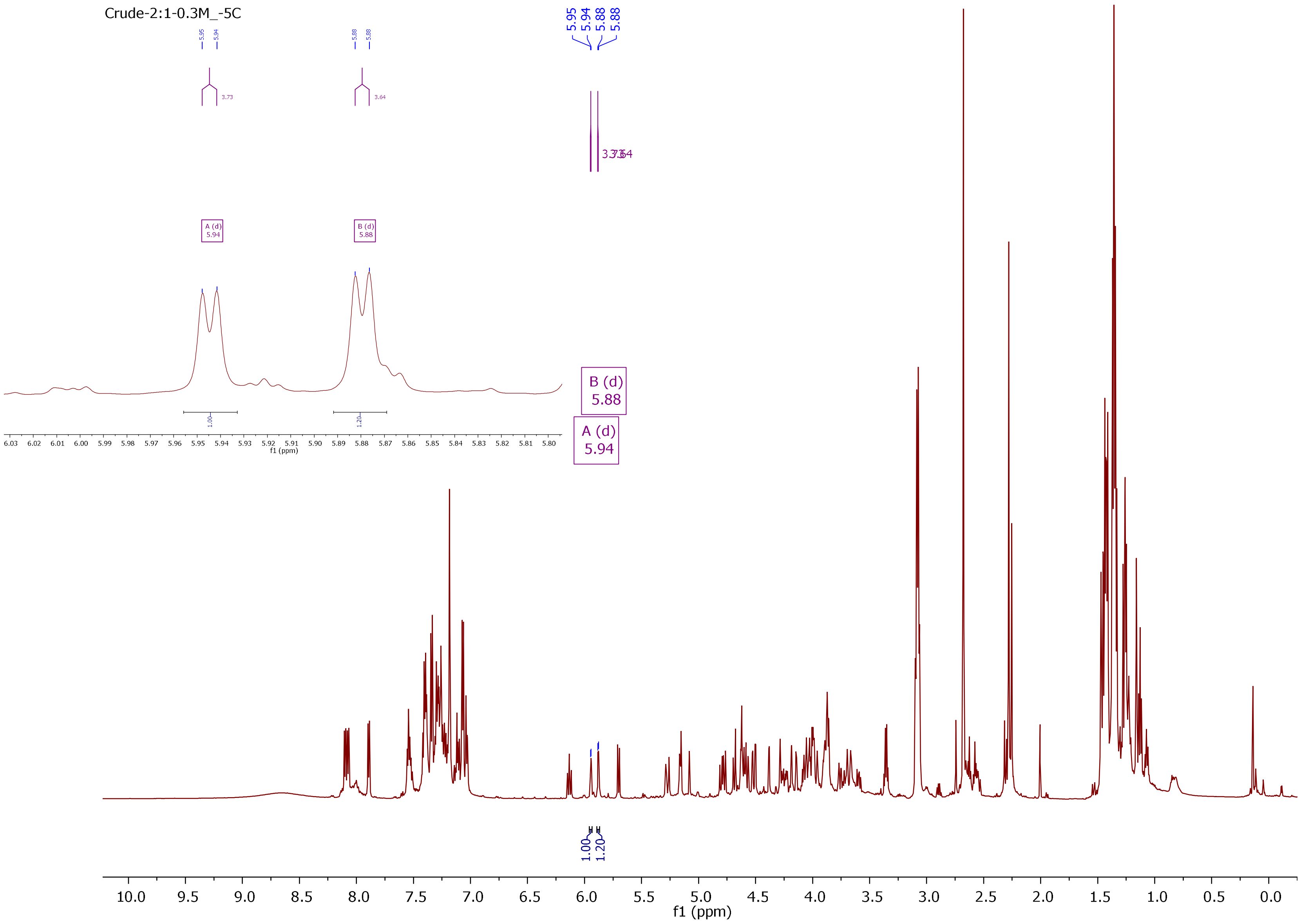

^{1}H NMR (600 MHz, C_6D_6) spectrum of 2,4,6-tri-*O*-acetyl-3-*O*-benzyl-α-D-glucopyranosyl *N*-phenyltrichloroacetmidate (**30α**)

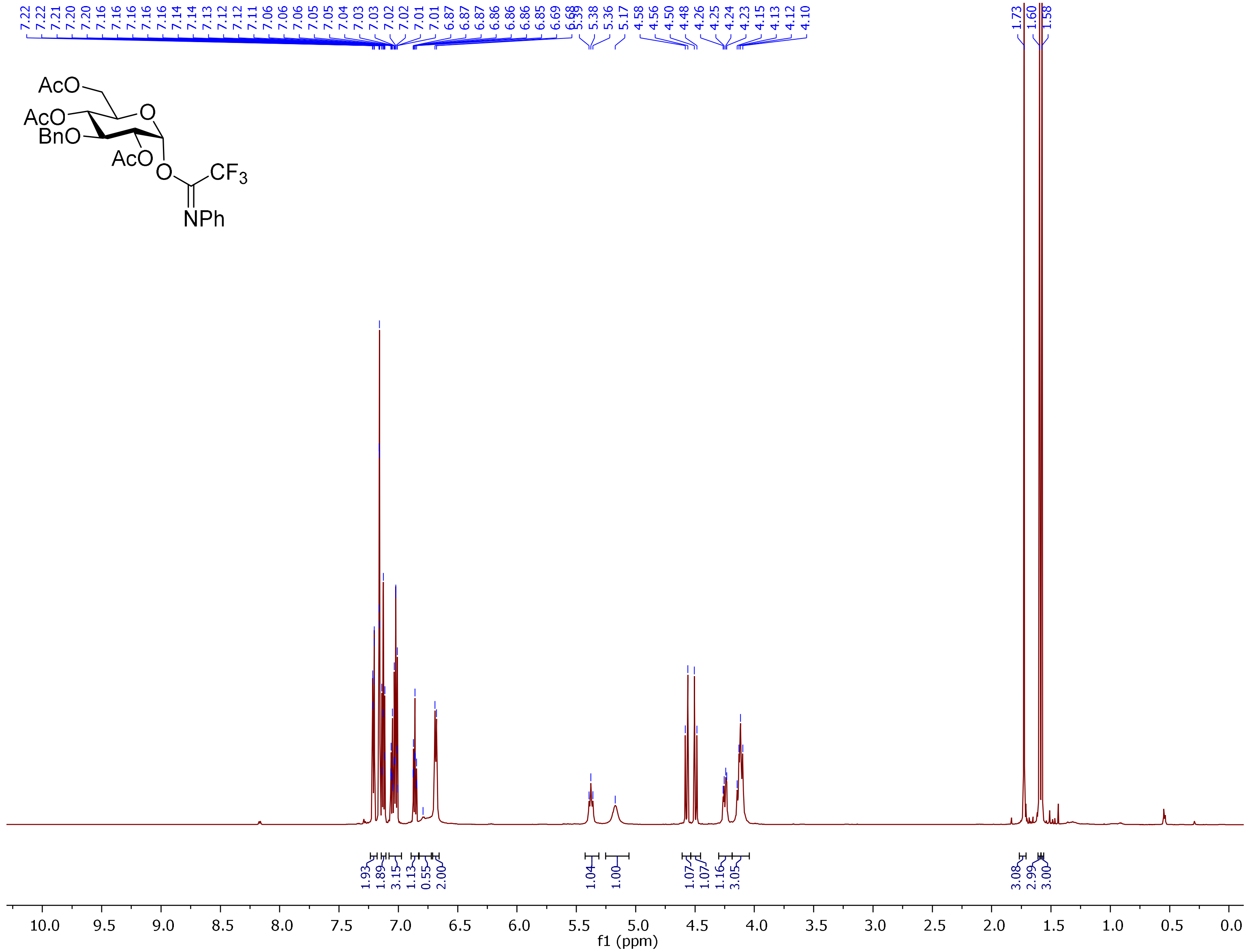

$^{13}C\{^{1}H\}$ NMR (151 MHz, C_6D_6) spectrum of 2,4,6-tri-*O*-acetyl-3-*O*-benzyl-α-D-glucopyranosyl *N*-phenyltrichloroacetmidate (**30α**)

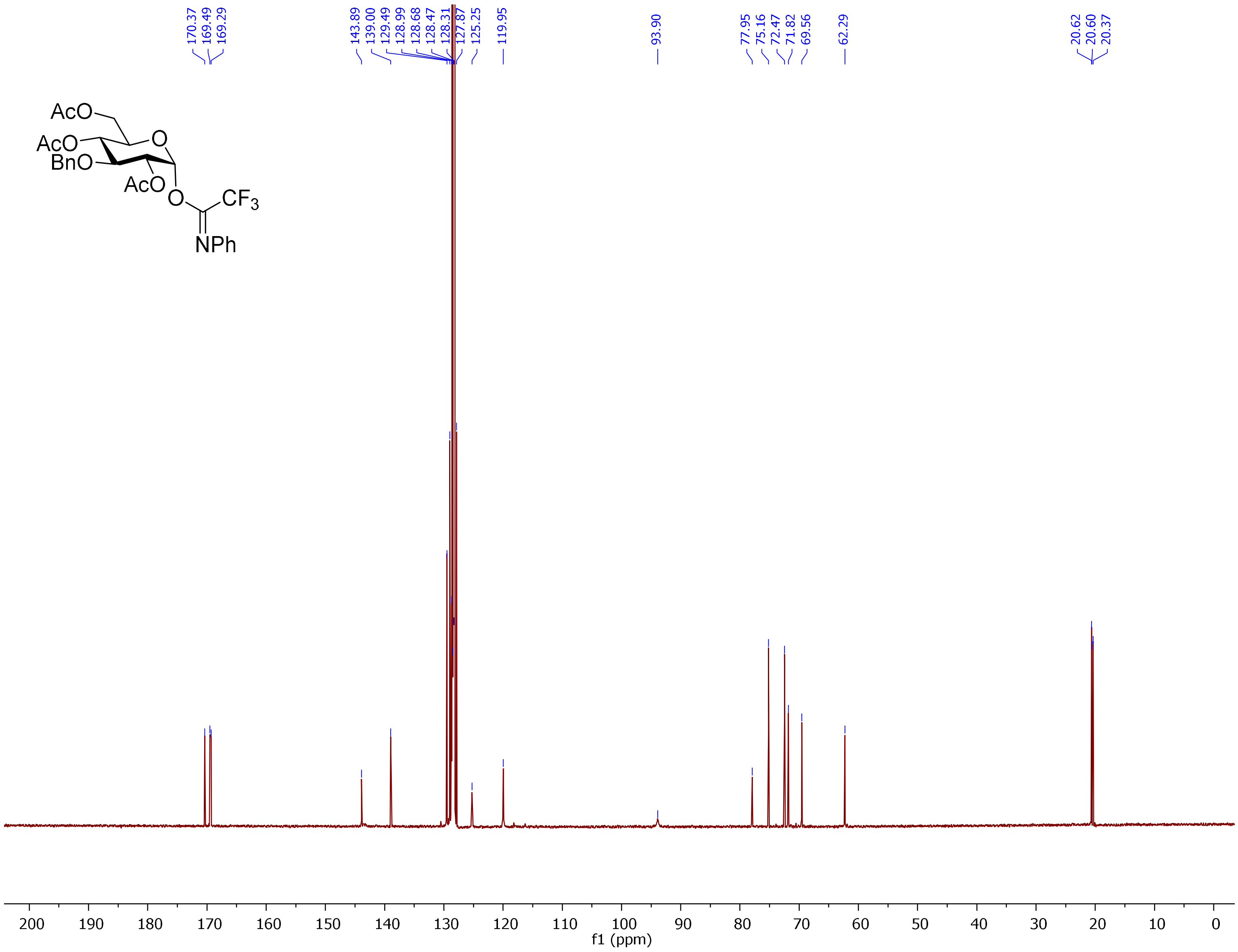

^{19}F NMR (564 MHz, C_6D_6) spectrum of 2,4,6-tri-*O*-acetyl-3-*O*-benzyl-α-D-glucopyranosyl *N*-phenyltrichloroacetmidate (**30α**)

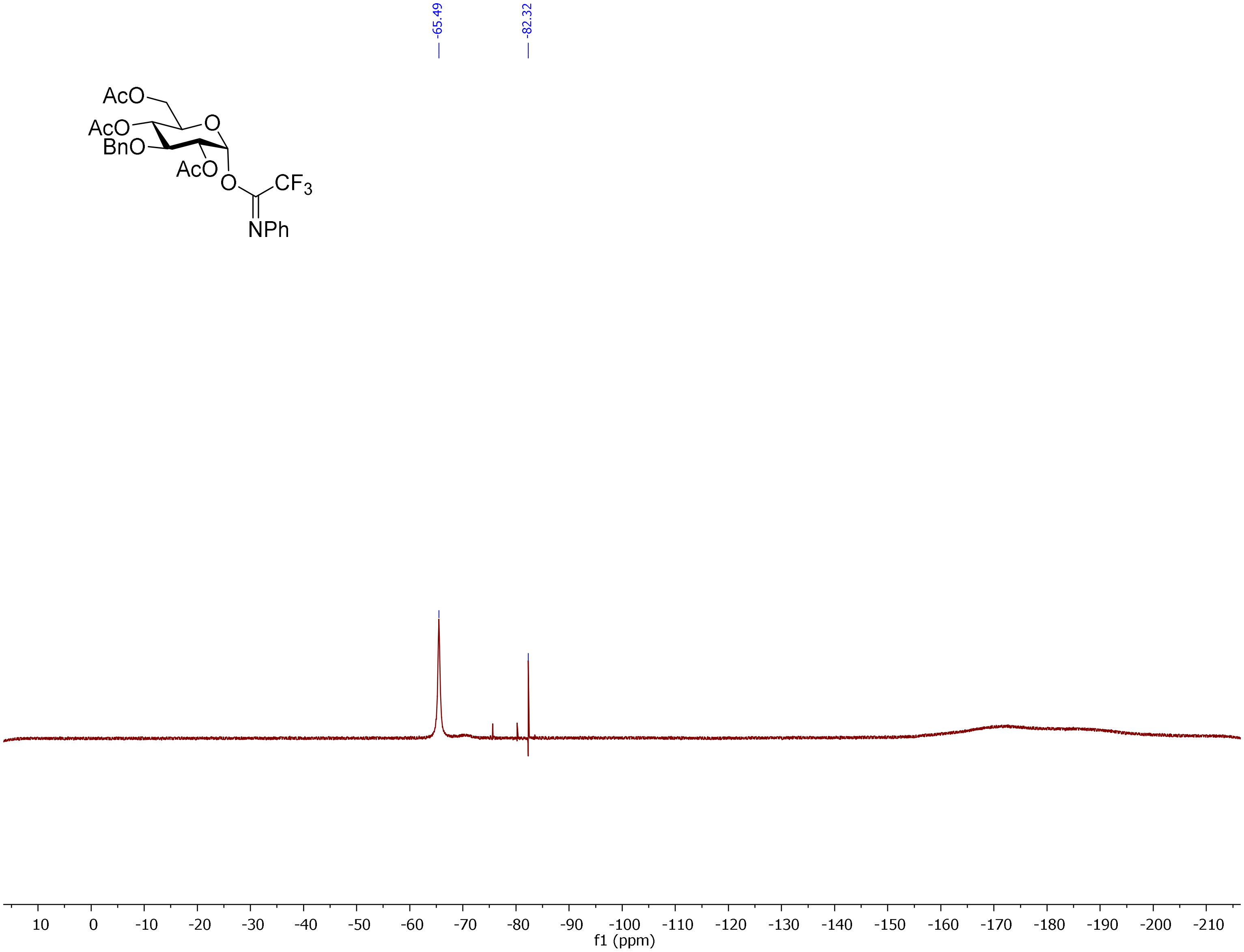

COSY NMR (600 MHz, C_6D_6) spectrum of 2,4,6-tri-*O*-acetyl-3-*O*-benzyl-α-D-glucopyranosyl *N*-phenyltrichloroacetmidate (**30α**)

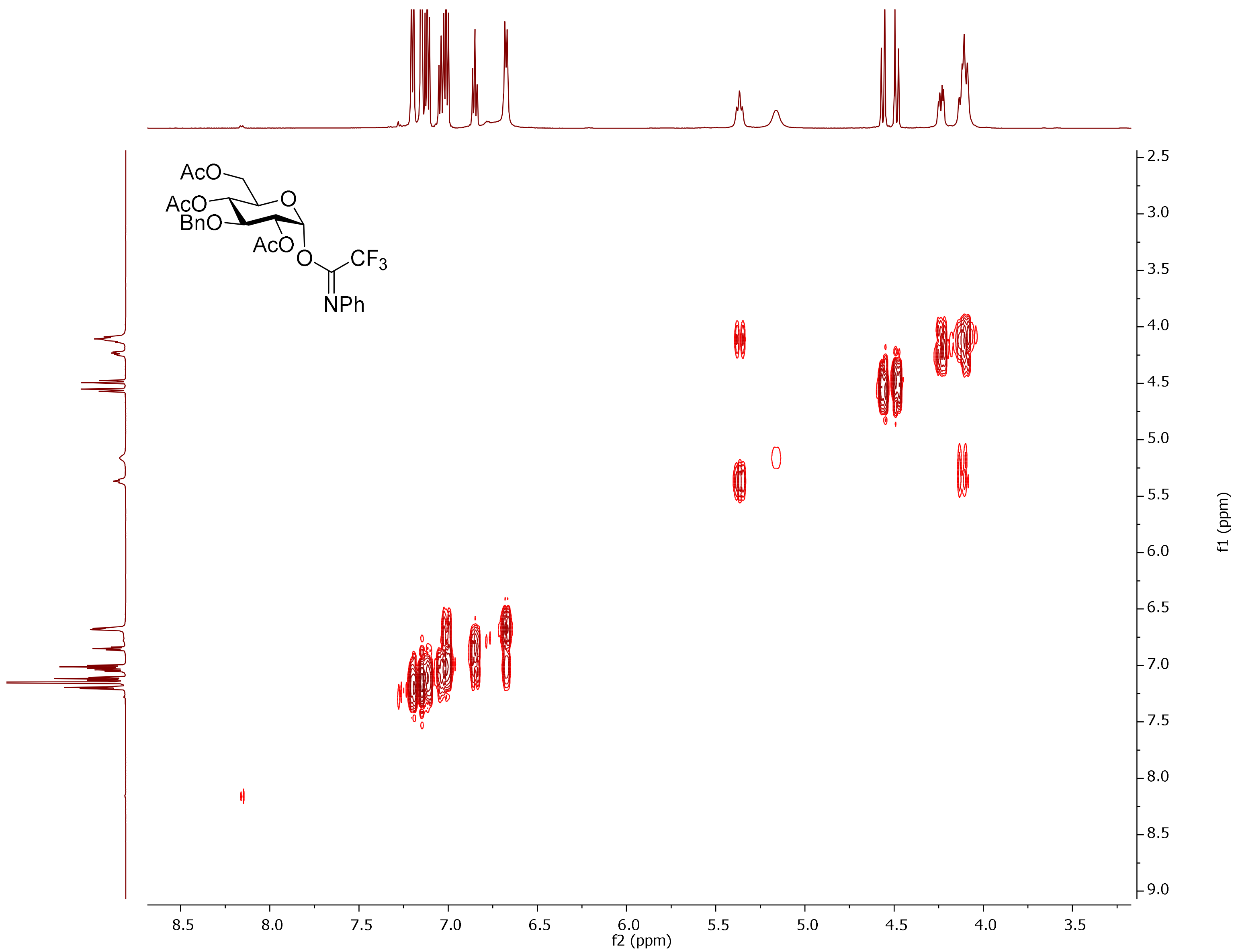

HSQC NMR (600 MHz, C_6D_6) spectrum of 2,4,6-tri-*O*-acetyl-3-*O*-benzyl-α-D-glucopyranosyl *N*-phenyltrichloroacetmidate (**30α**)

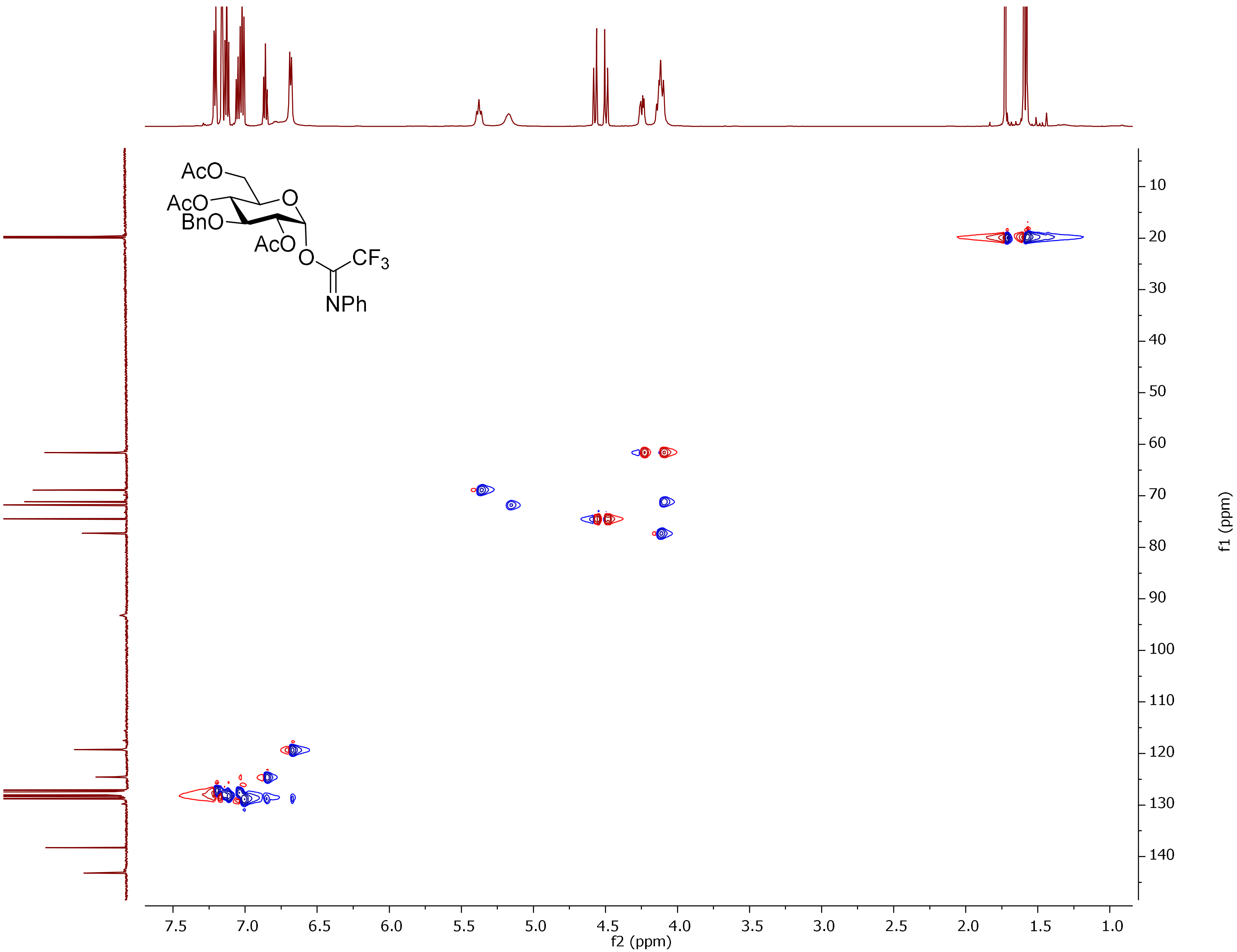

^{1}H NMR (600 MHz, C_6D_6) spectrum of 2,4,6-tri-*O*-acetyl-3-*O*-benzyl-β-D-glucopyranosyl *N*-phenyltrichloroacetmidate (**30β**)

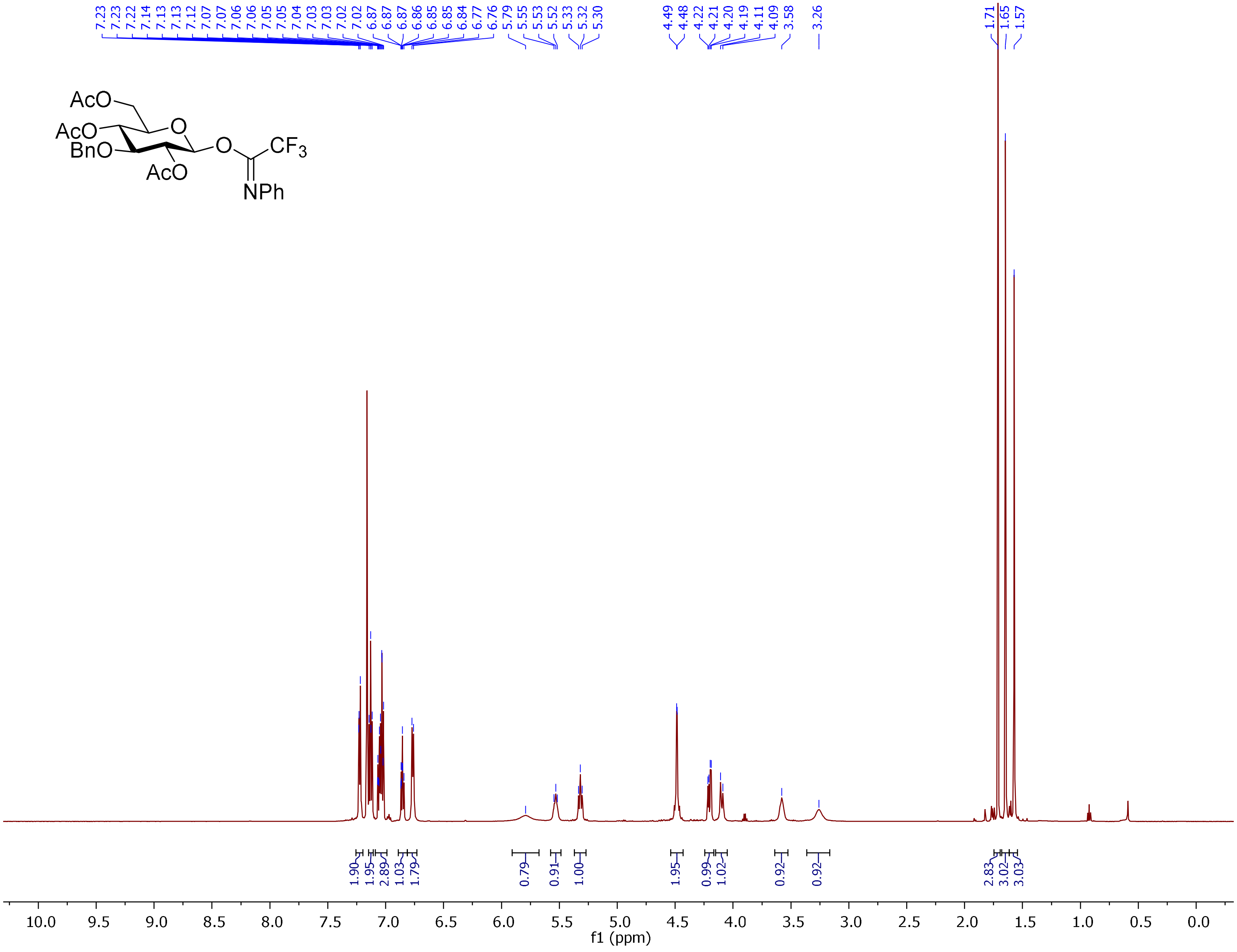

$^{13}C\{^{1}H\}$ NMR (151 MHz, C_6D_6) spectrum of 2,4,6-tri-*O*-acetyl-3-*O*-benzyl-β-D-glucopyranosyl *N*-phenyltrichloroacetmidate (**30β**)

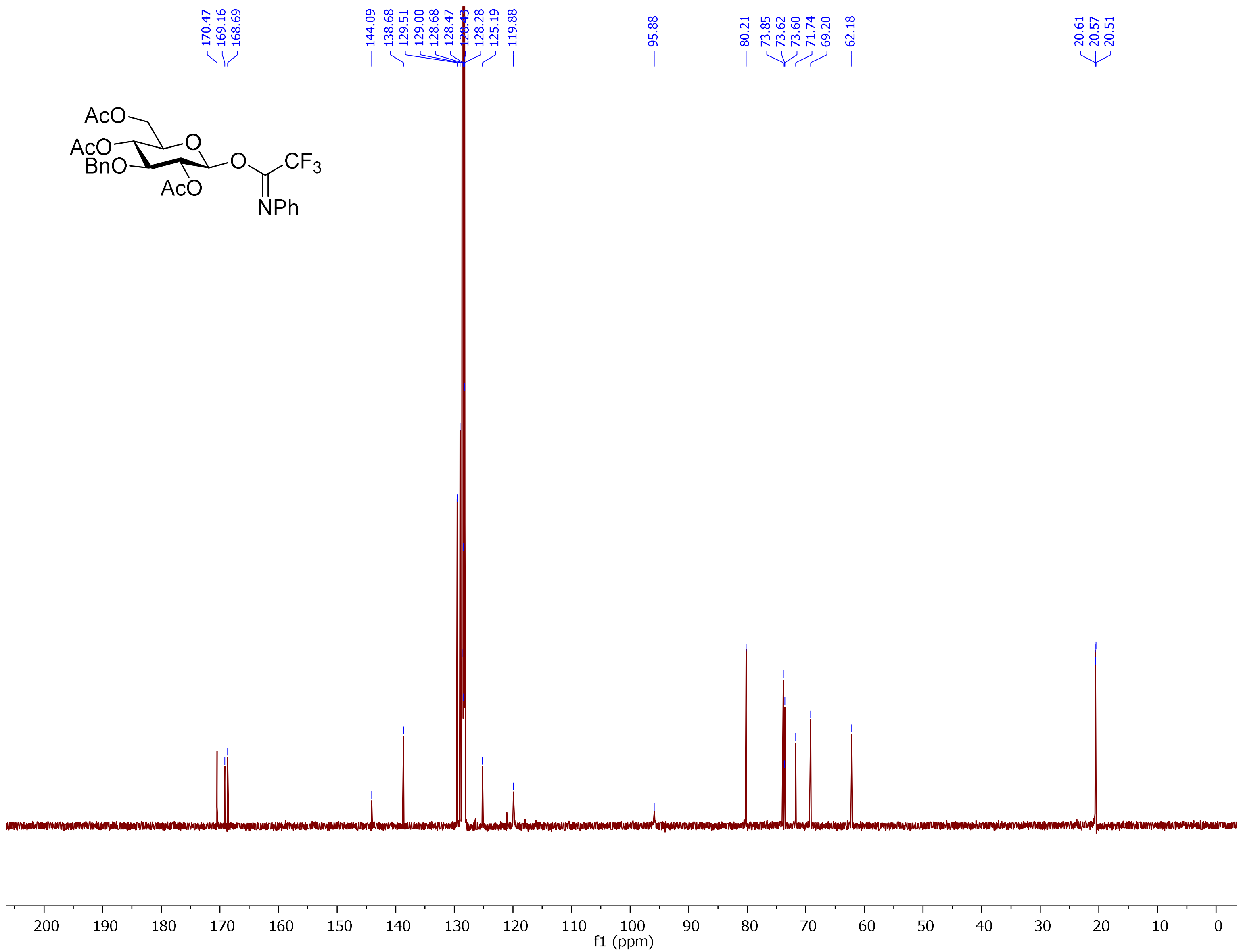

^{19}F NMR (564 MHz, C_6D_6) spectrum of 2,4,6-tri-*O*-acetyl-3-*O*-benzyl-β-D-glucopyranosyl *N*-phenyltrichloroacetmidate (**30β**)

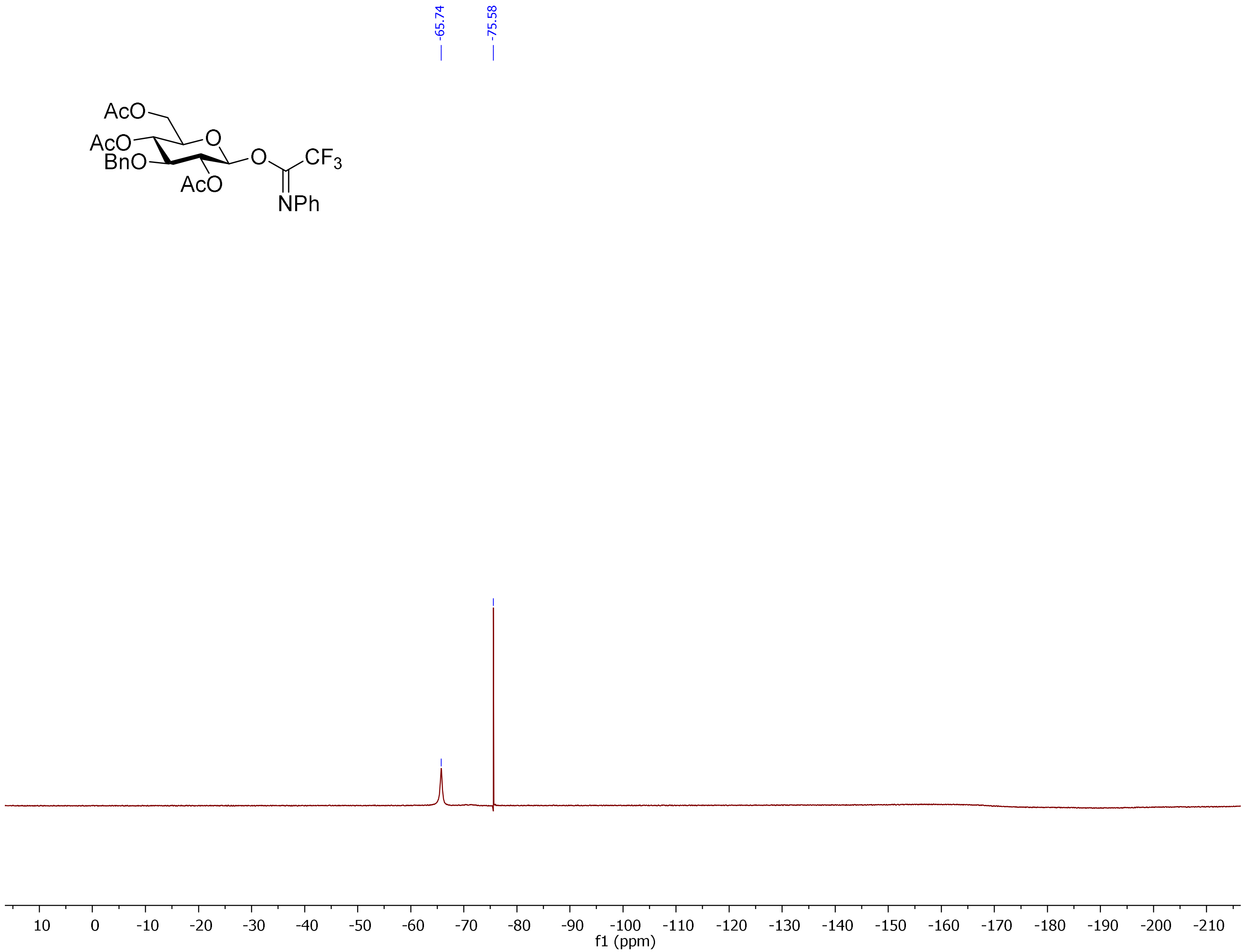

COSY NMR (600 MHz, C_6D_6) spectrum of 2,4,6-tri-*O*-acetyl-3-*O*-benzyl-β-D-glucopyranosyl *N*-phenyltrichloroacetmidate (**30β**)

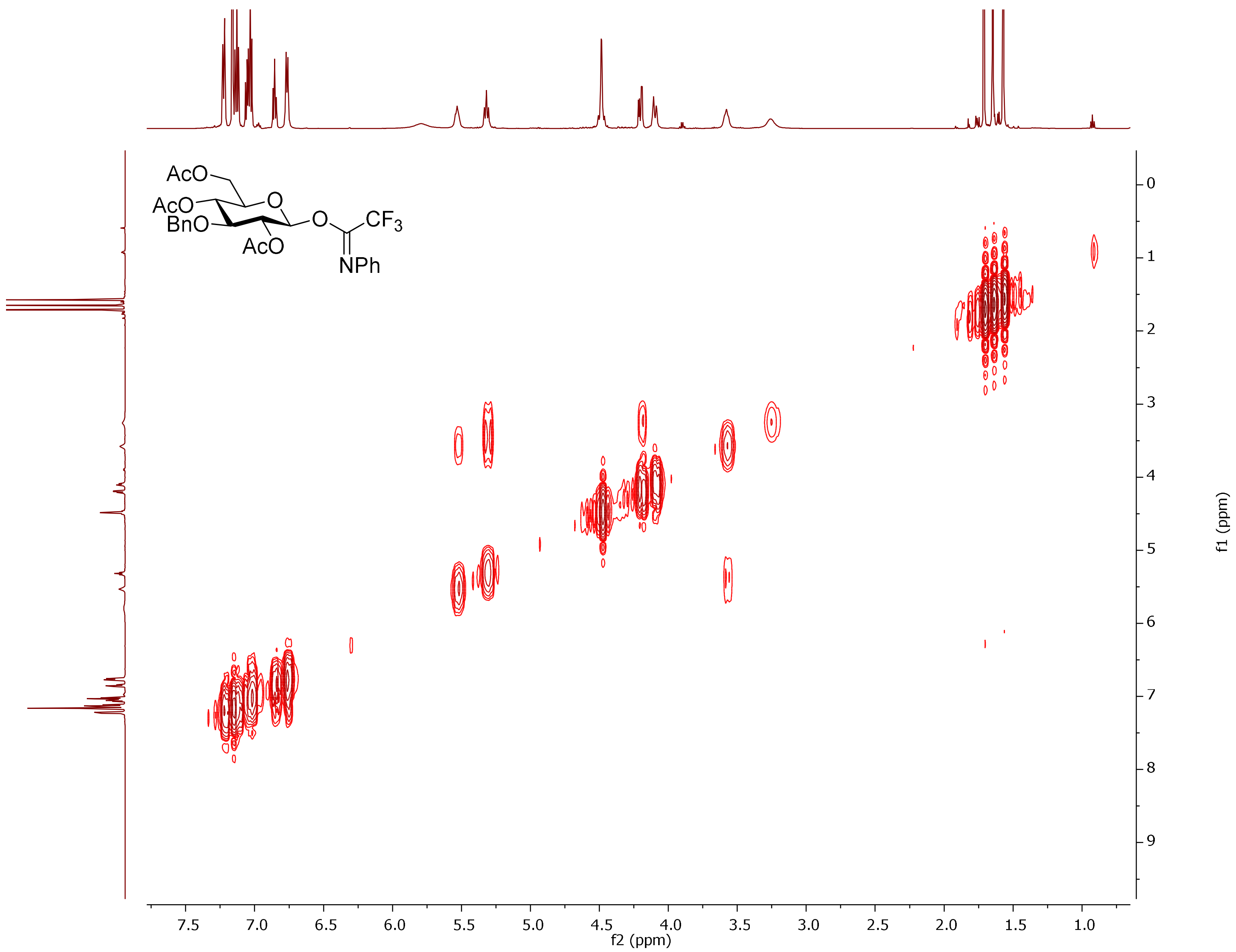

HSQC NMR (600 MHz, C_6D_6) spectrum of 2,4,6-tri-*O*-acetyl-3-*O*-benzyl-β-D-glucopyranosyl *N*-phenyltrichloroacetmidate (**30β**)

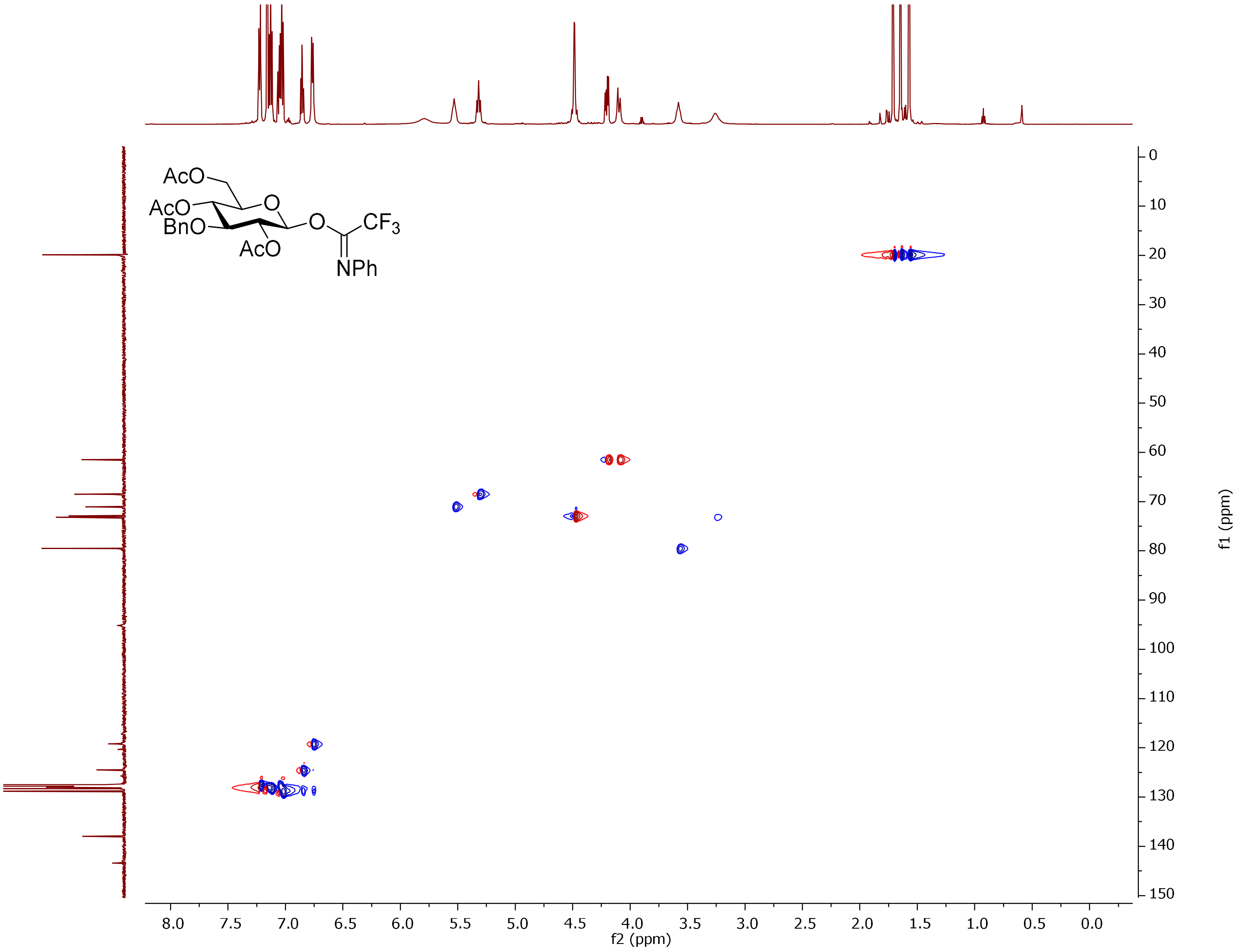

^{1}H NMR (600 MHz, $CDCl_3$) spectrum of 1,2,4,6-tetra-*O*-acetyl-β-D-glucopyranose (**31β**)

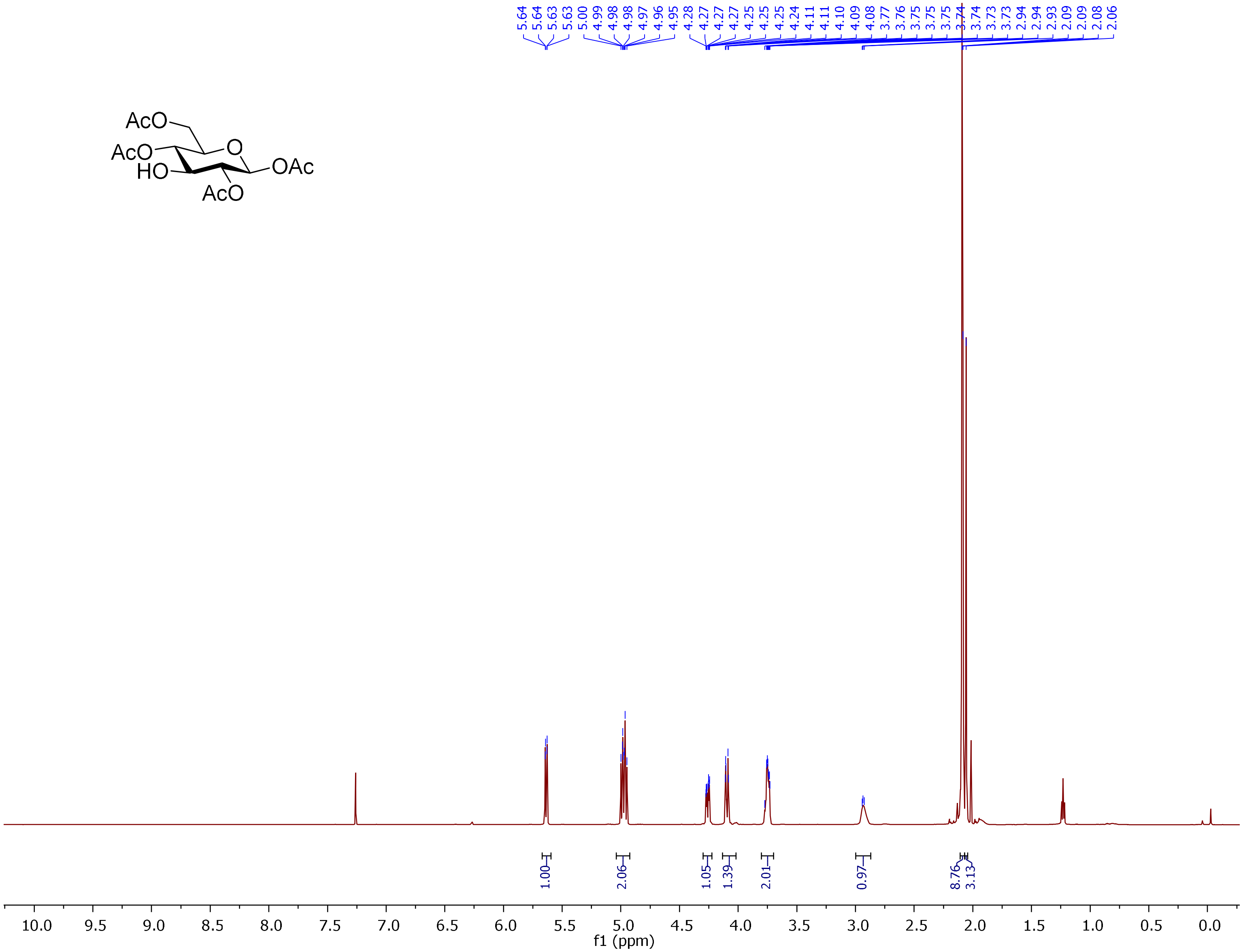

$^{13}C\{^{1}H\}$ NMR (151 MHz, $CDCl_3$) spectrum of 1,2,4,6-tetra-*O*-acetyl-β-D-glucopyranose (**31β**)

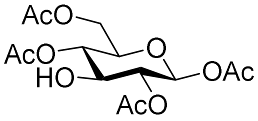

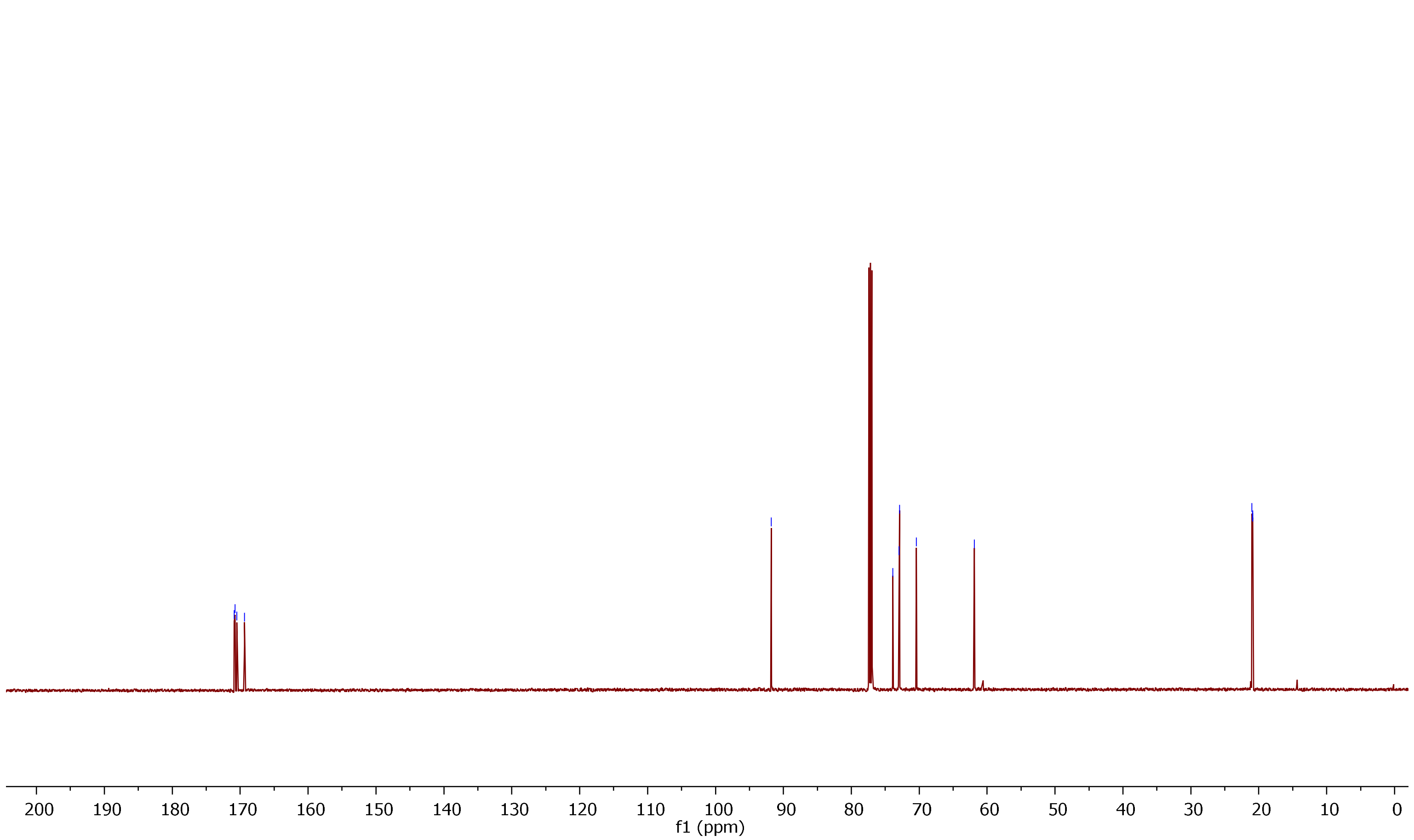

COSY NMR (600 MHz, $CDCl_3$) spectrum of 1,2,4,6-tetra-*O*-acetyl-β-D-glucopyranose (**31β**)

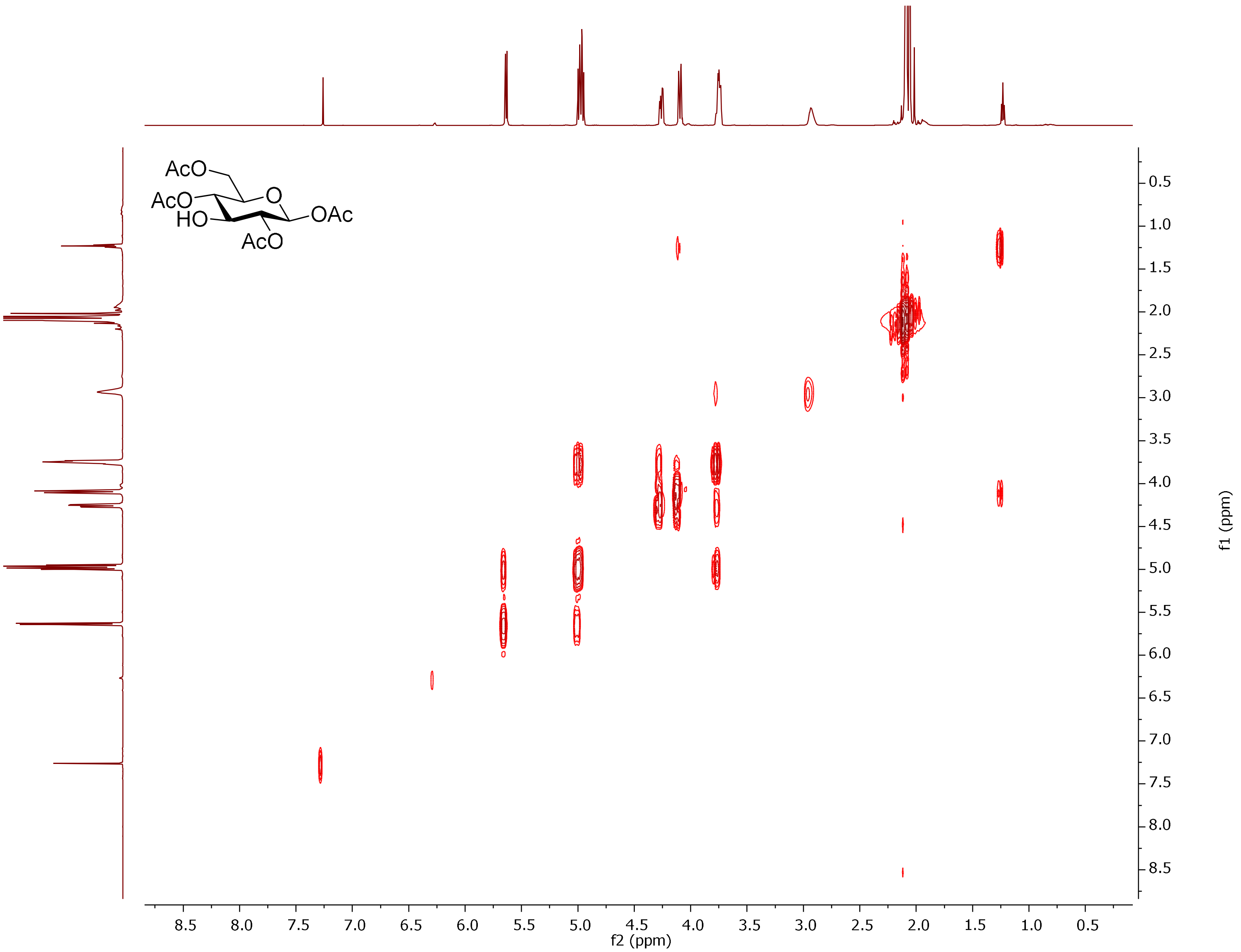

HSQC NMR (600 MHz, $CDCl_3$) spectrum of 1,2,4,6-tetra-*O*-acetyl-β-D-glucopyranose (**31β**)

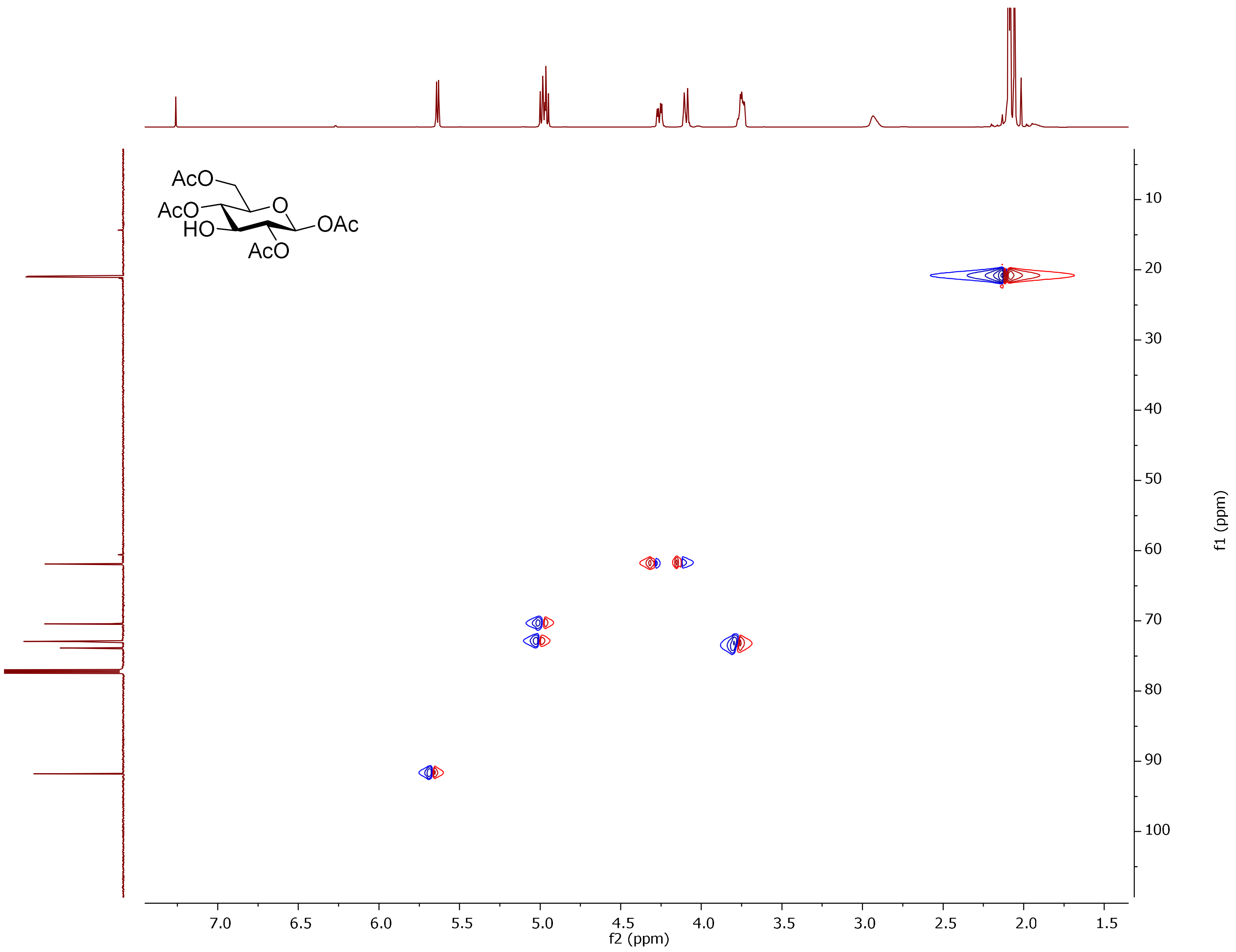

^{1}H NMR (600 MHz, $CDCl_3$) spectrum of 1,2,4,6-Tetra-*O*-acetyl-3-*O*-(2,4,6-tri-*O*-acetyl-3-*O*-benzyl-α-D-glucopyranosyl)-β-D-glucpoyranose (**32α**)

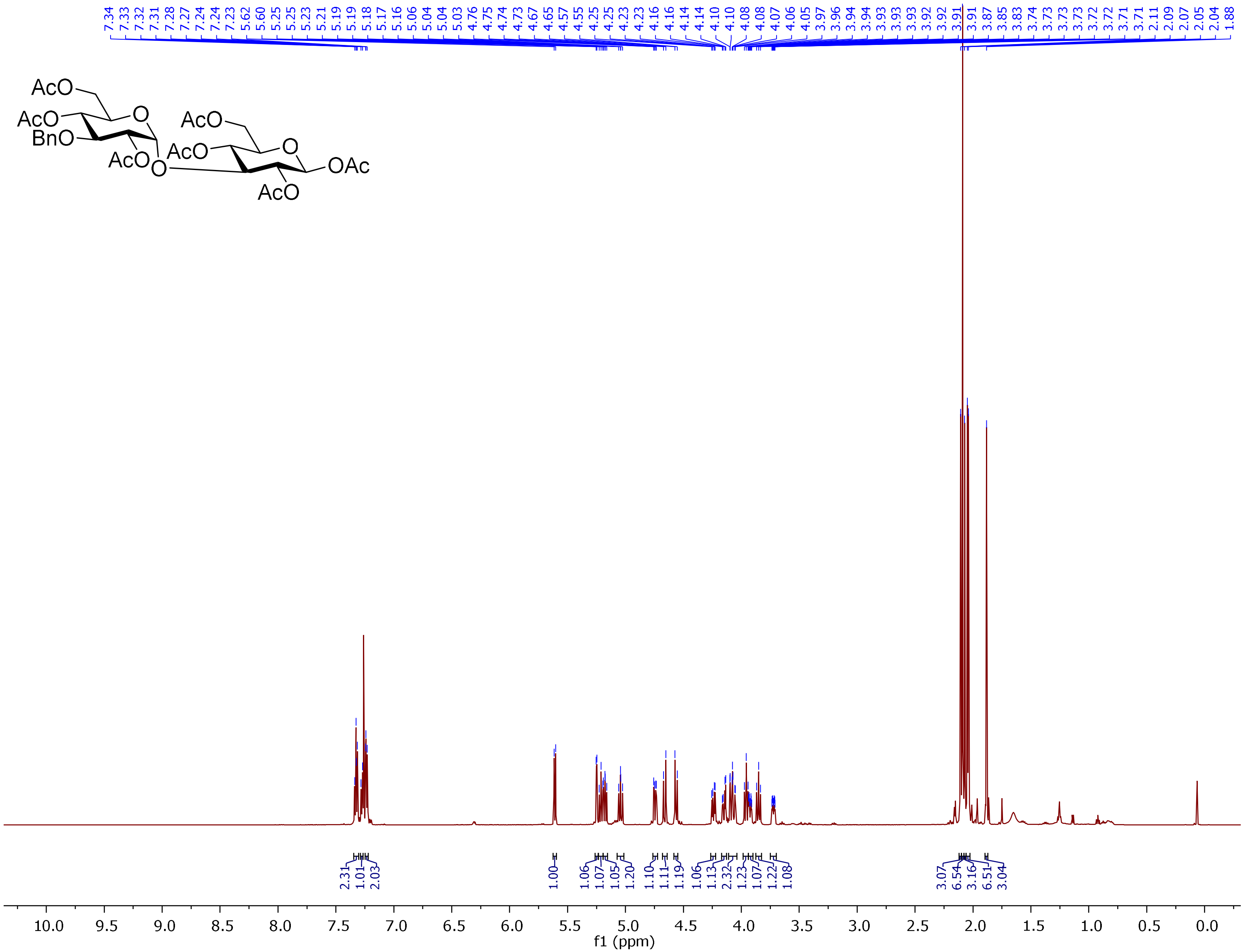

$^{13}C\{^{1}H\}$ NMR (151 MHz, $CDCl_3$) spectrum of 1,2,4,6-Tetra-*O*-acetyl-3-*O*-(2,4,6-tri-*O*-acetyl-3-*O*-benzyl-α-D-glucopyranosyl)-β-D-glucpoyranose (**32α**)

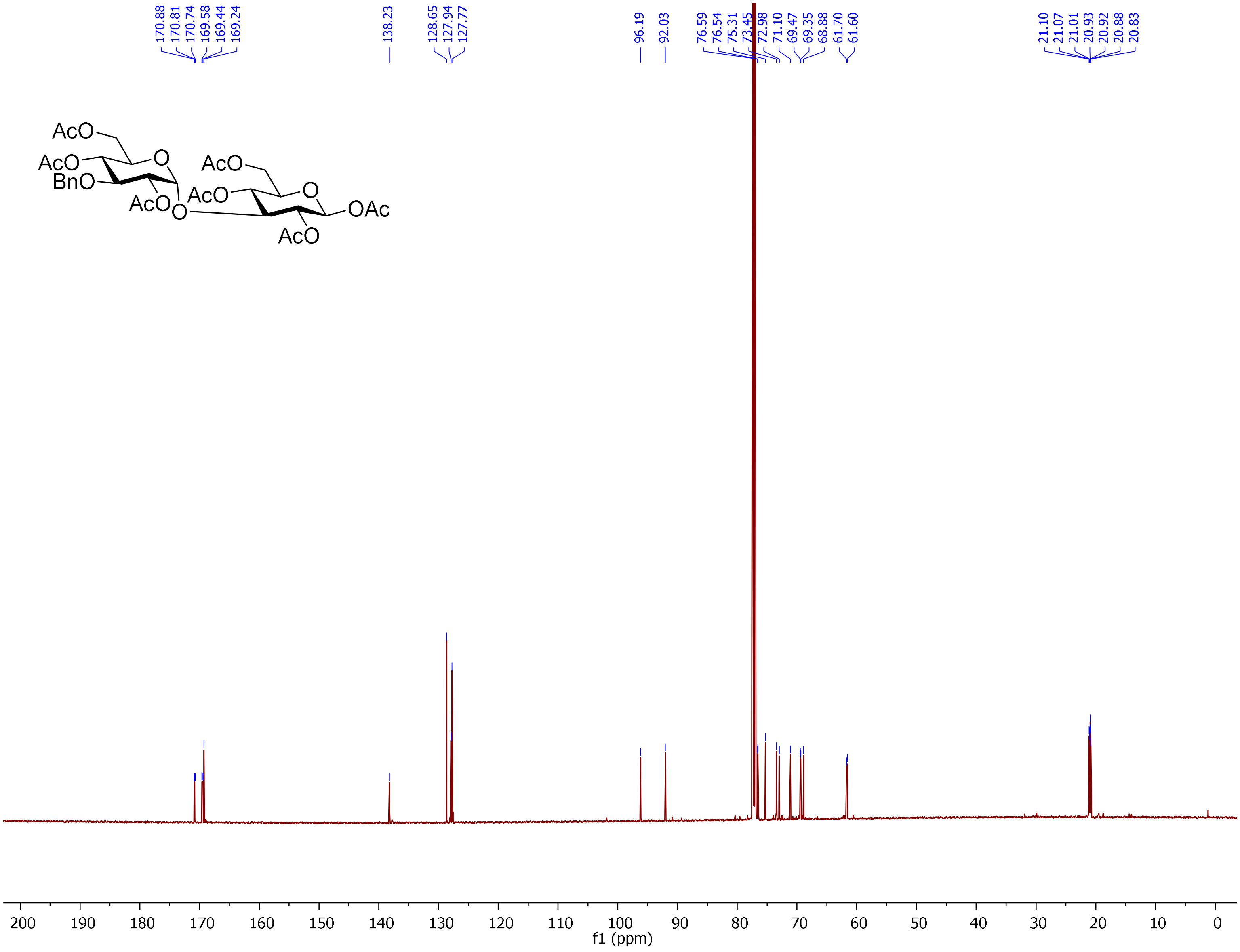

COSY NMR (600 MHz, $CDCl_3$) spectrum of 1,2,4,6-Tetra-*O*-acetyl-3-*O*-(2,4,6-tri-*O*-acetyl-3-*O*-benzyl-α-D-glucopyranosyl)-β-D-glucpoyranose (**32α**)

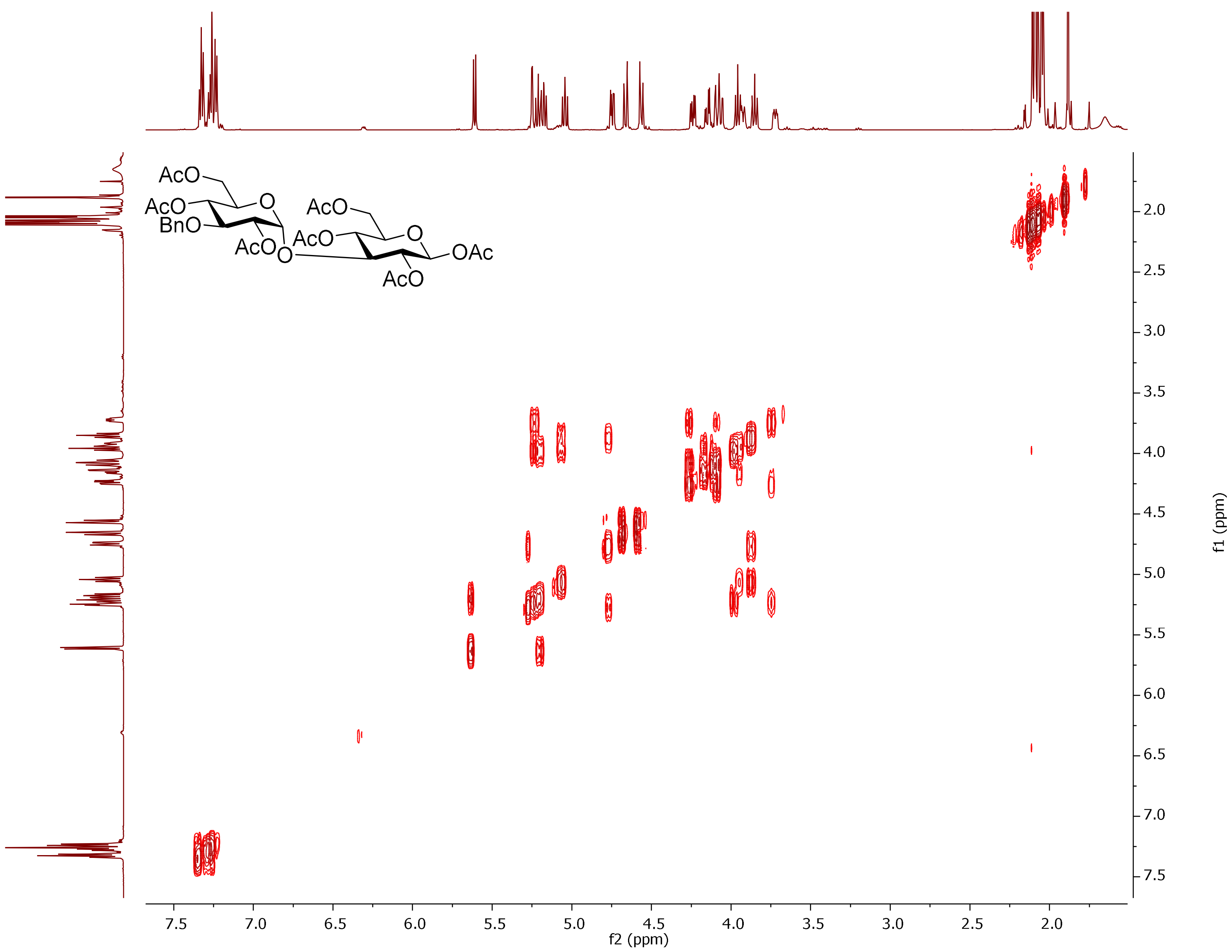

HSQC NMR (600 MHz, CDCl$_3$) spectrum of 1,2,4,6-Tetra-*O*-acetyl-3-*O*-(2,4,6-tri-*O*-acetyl-3-*O*-benzyl-α-D-glucopyranosyl)-β-D-glucpoyranose (**32α**)

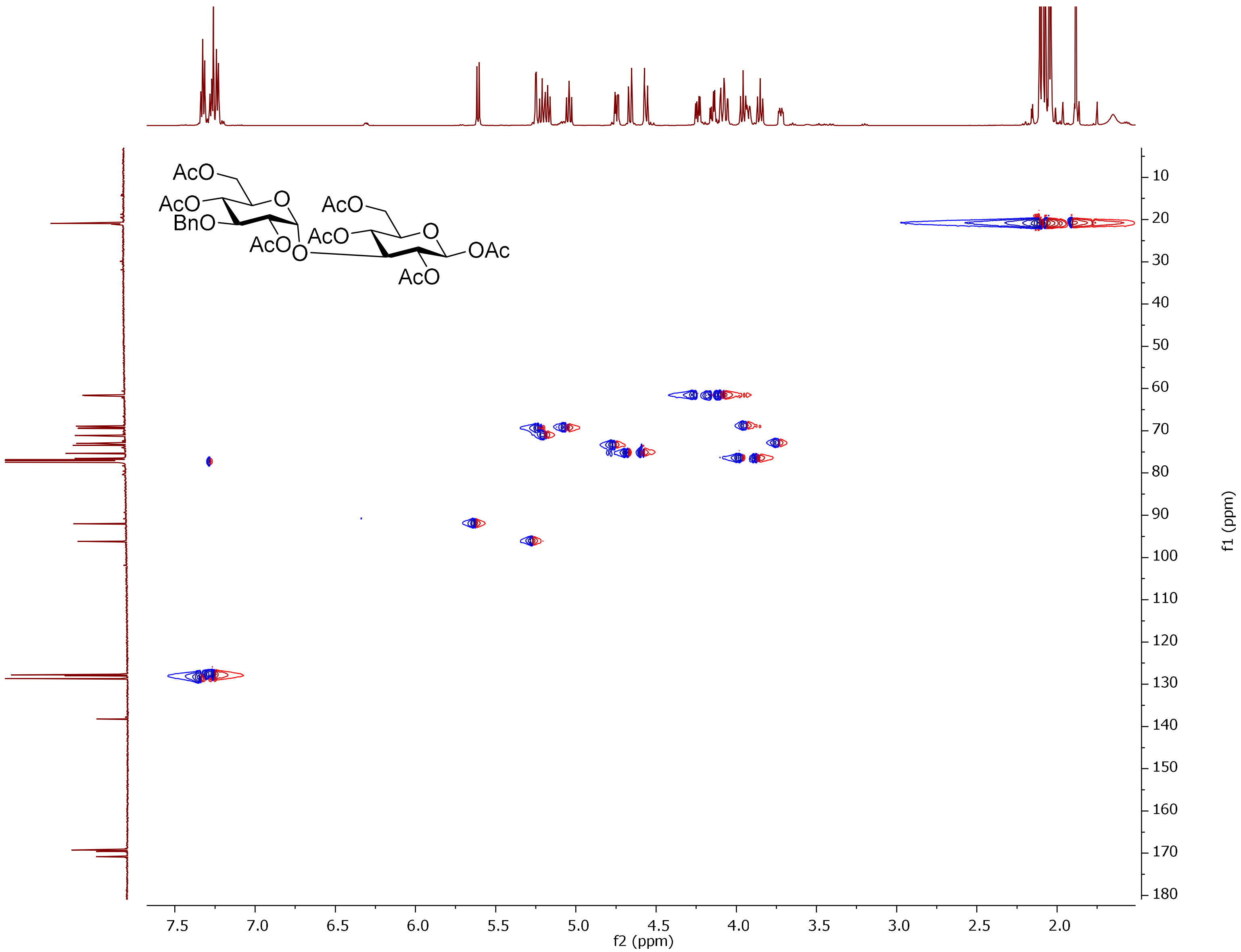

HMBC NMR (600 MHz, $CDCl_3$) spectrum of 1,2,4,6-Tetra-*O*-acetyl-3-*O*-(2,4,6-tri-*O*-acetyl-3-*O*-benzyl-α-D-glucopyranosyl)-β-D-glucpoyranose (**32α**)

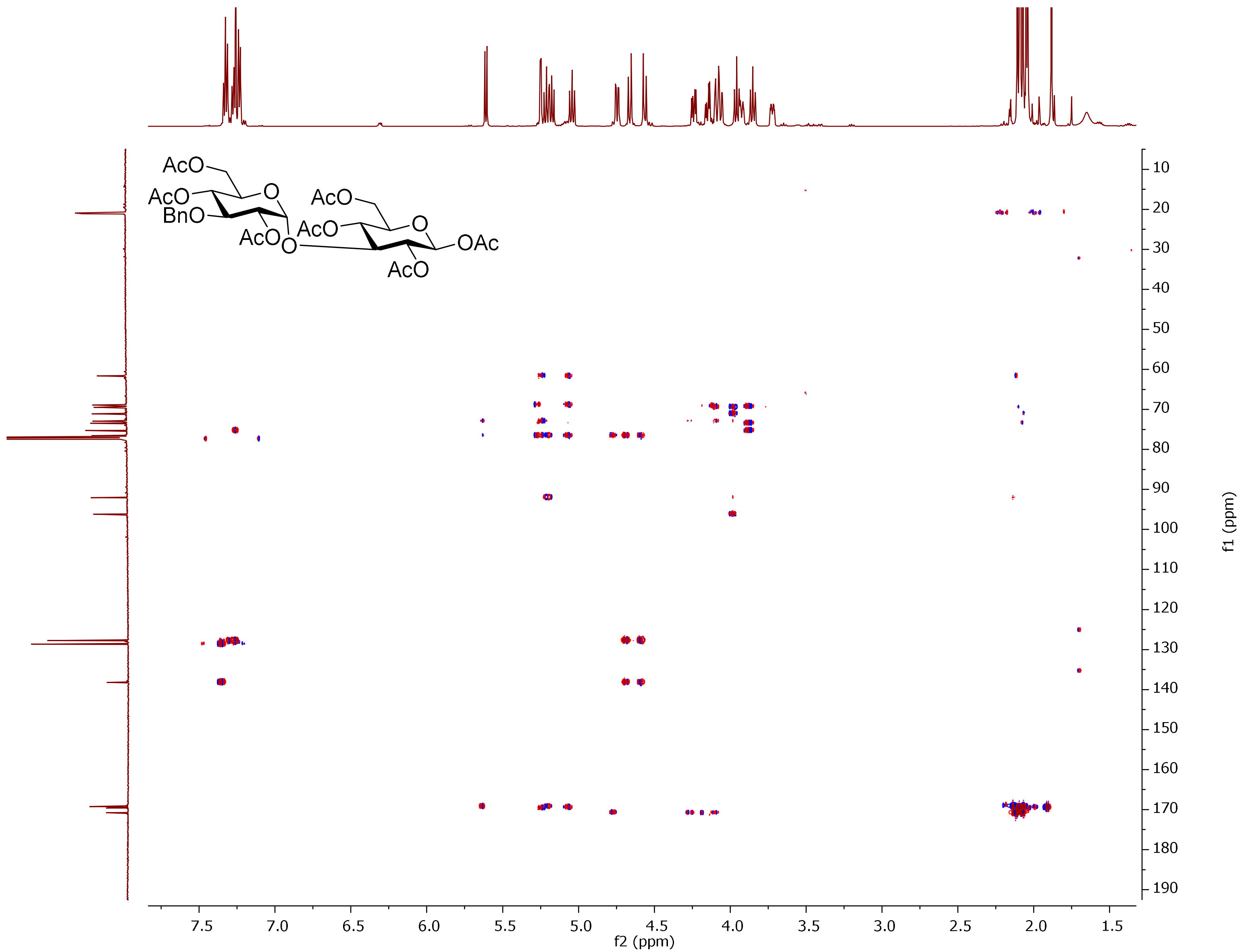

^{1}H NMR (600 MHz, $CDCl_3$) spectrum of 1,2,4,6-Tetra-*O*-acetyl-3-*O*-(2,4,6-tri-*O*-acetyl-3-*O*-benzyl-β-D-glucopyranosyl)-β-D-glucpoyranose (**32β**)

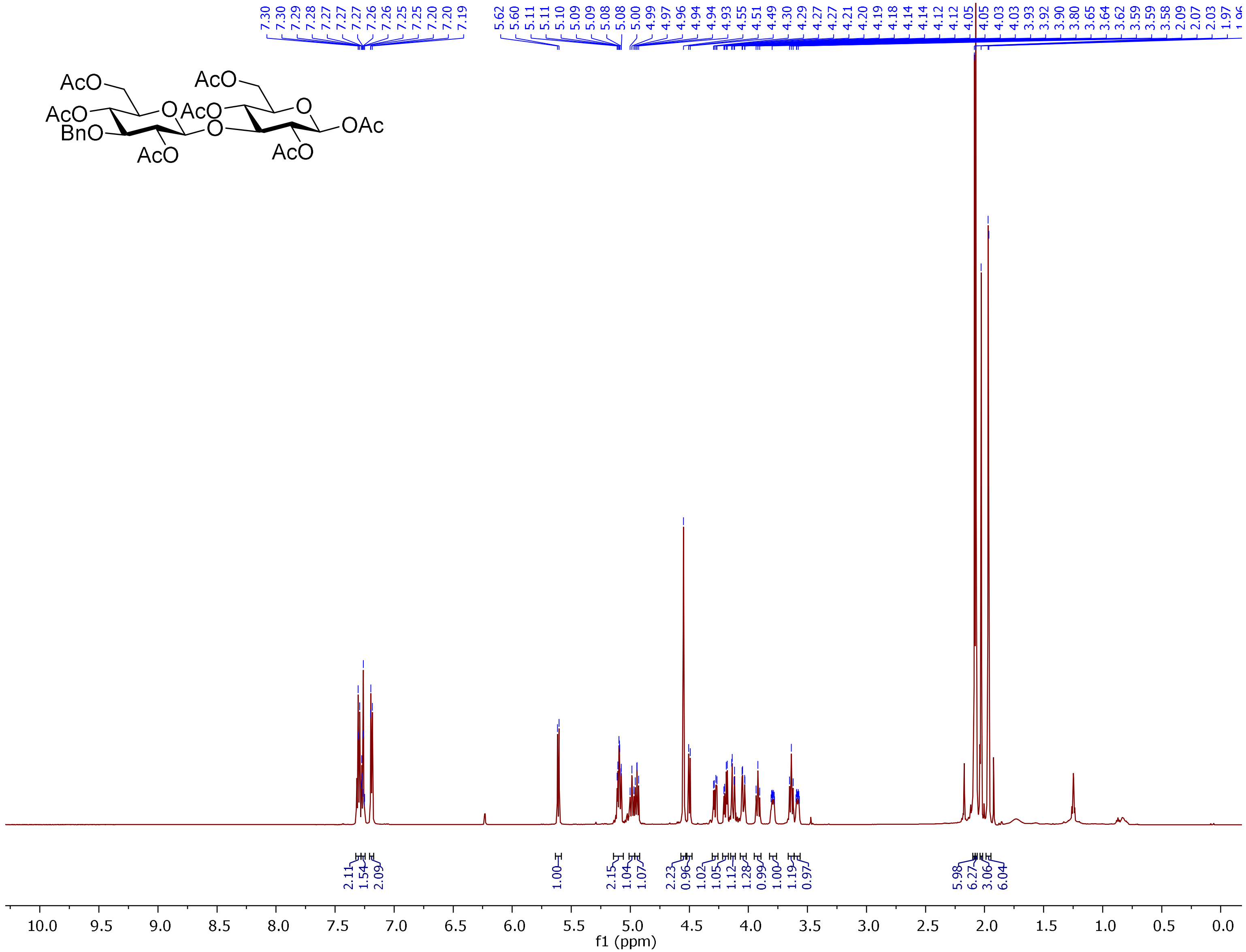

$^{13}C\{^{1}H\}$ NMR (151 MHz, $CDCl_3$) spectrum of 1,2,4,6-Tetra-*O*-acetyl-3-*O*-(2,4,6-tri-*O*-acetyl-3-*O*-benzyl-β-D-glucopyranosyl)-β-D-glucpoyranose (**32β**)

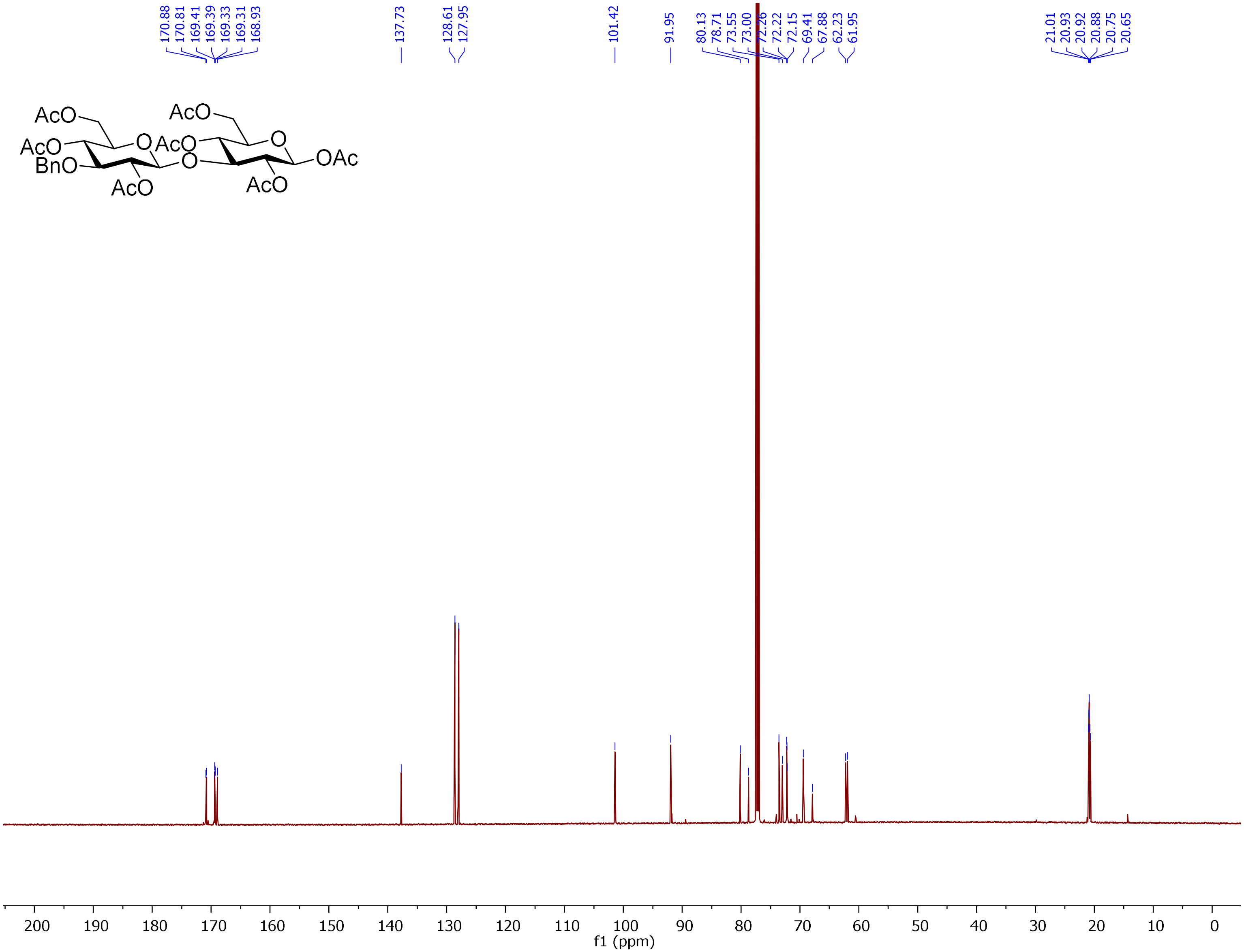

COSY NMR (600 MHz, $CDCl_3$) spectrum of 1,2,4,6-Tetra-*O*-acetyl-3-*O*-(2,4,6-tri-*O*-acetyl-3-*O*-benzyl-β-D-glucopyranosyl)-β-D-glucpoyranose (**32β**)

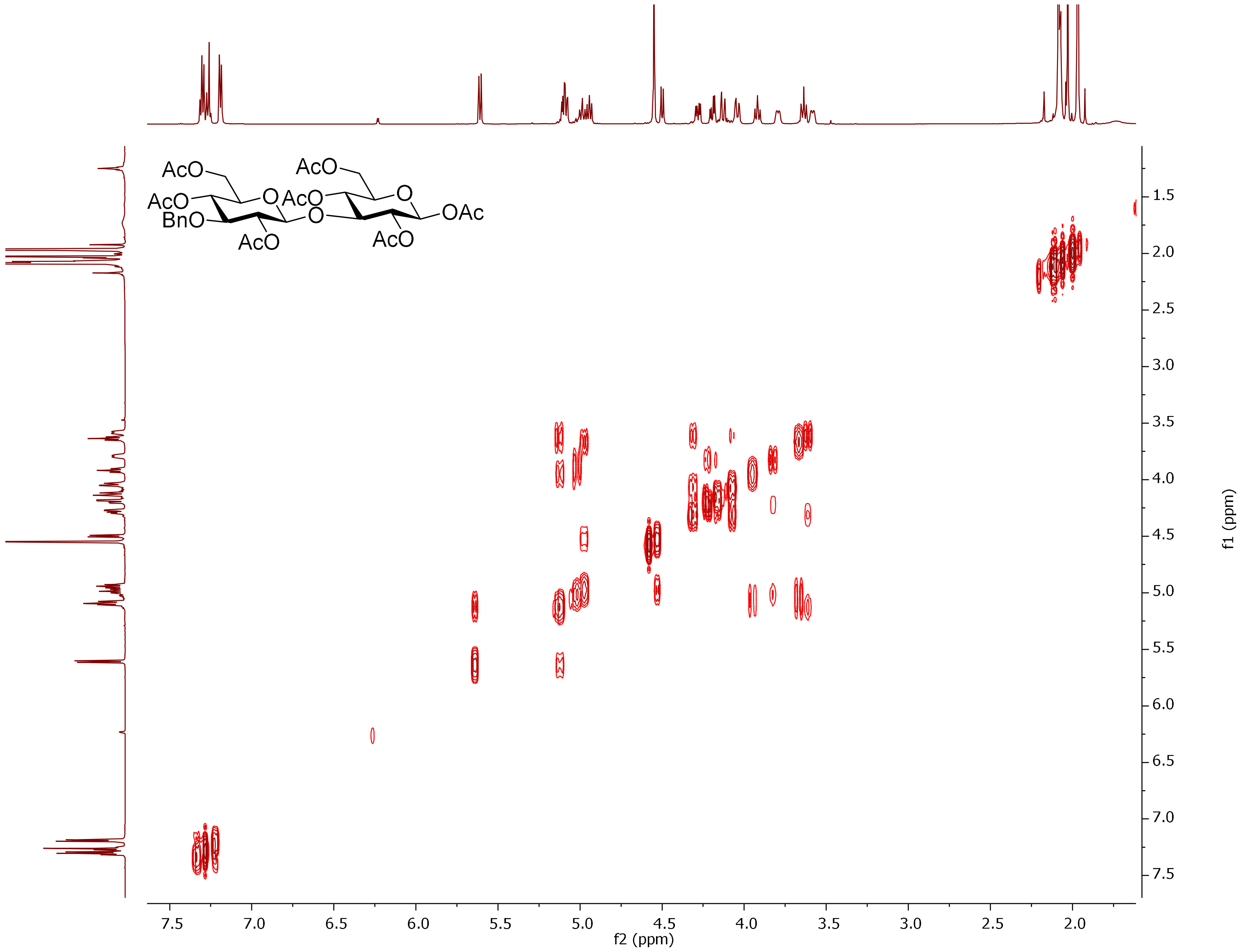

HSQC NMR (600 MHz, $CDCl_3$) spectrum of 1,2,4,6-Tetra-*O*-acetyl-3-*O*-(2,4,6-tri-*O*-acetyl-3-*O*-benzyl-β-D-glucopyranosyl)-β-D-glucpoyranose (**32β**)

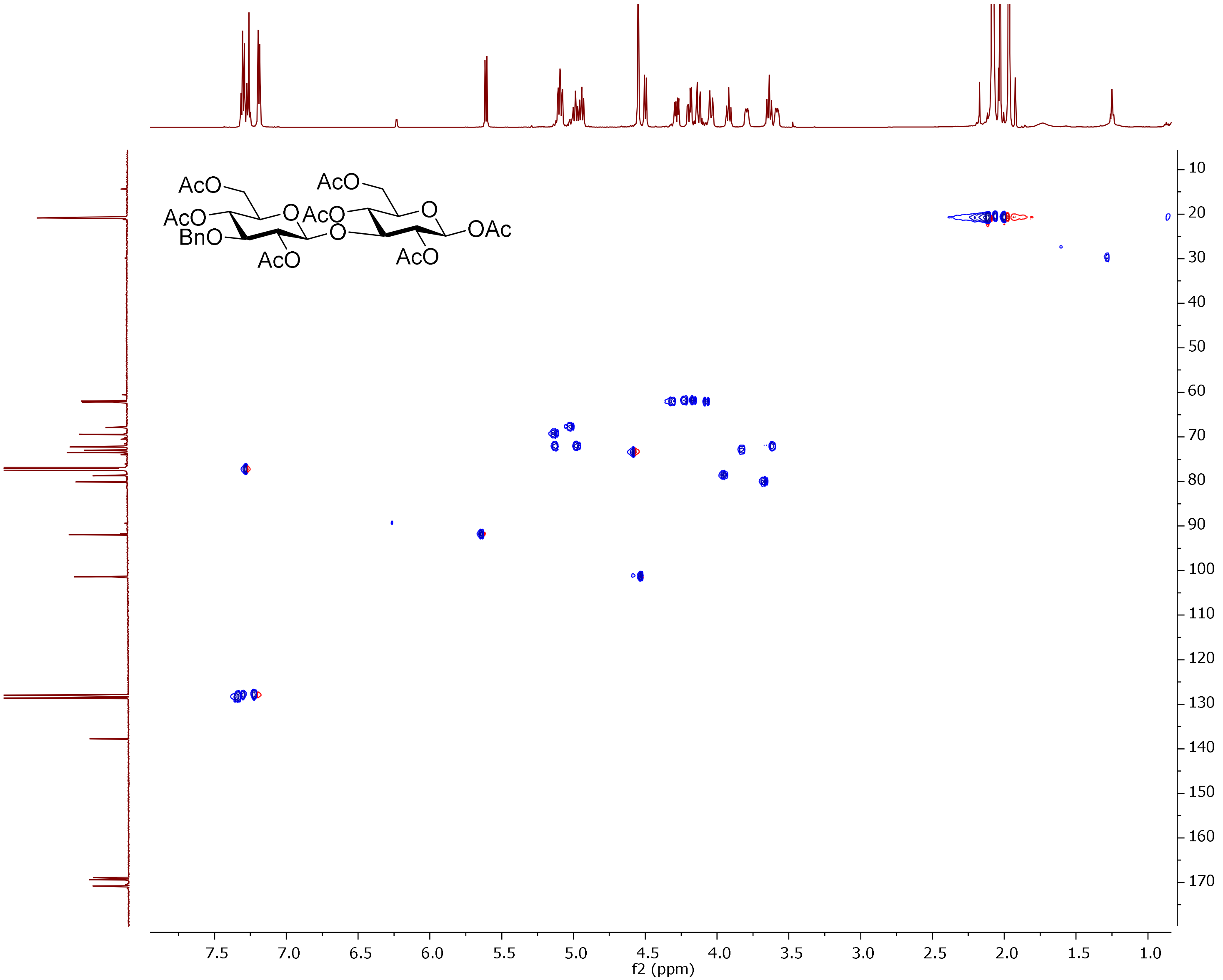

HMBC NMR (600 MHz, $CDCl_3$) spectrum of 1,2,4,6-Tetra-*O*-acetyl-3-*O*-(2,4,6-tri-*O*-acetyl-3-*O*-benzyl-β-D-glucopyranosyl)-β-D-glucpoyranose (**32β**)

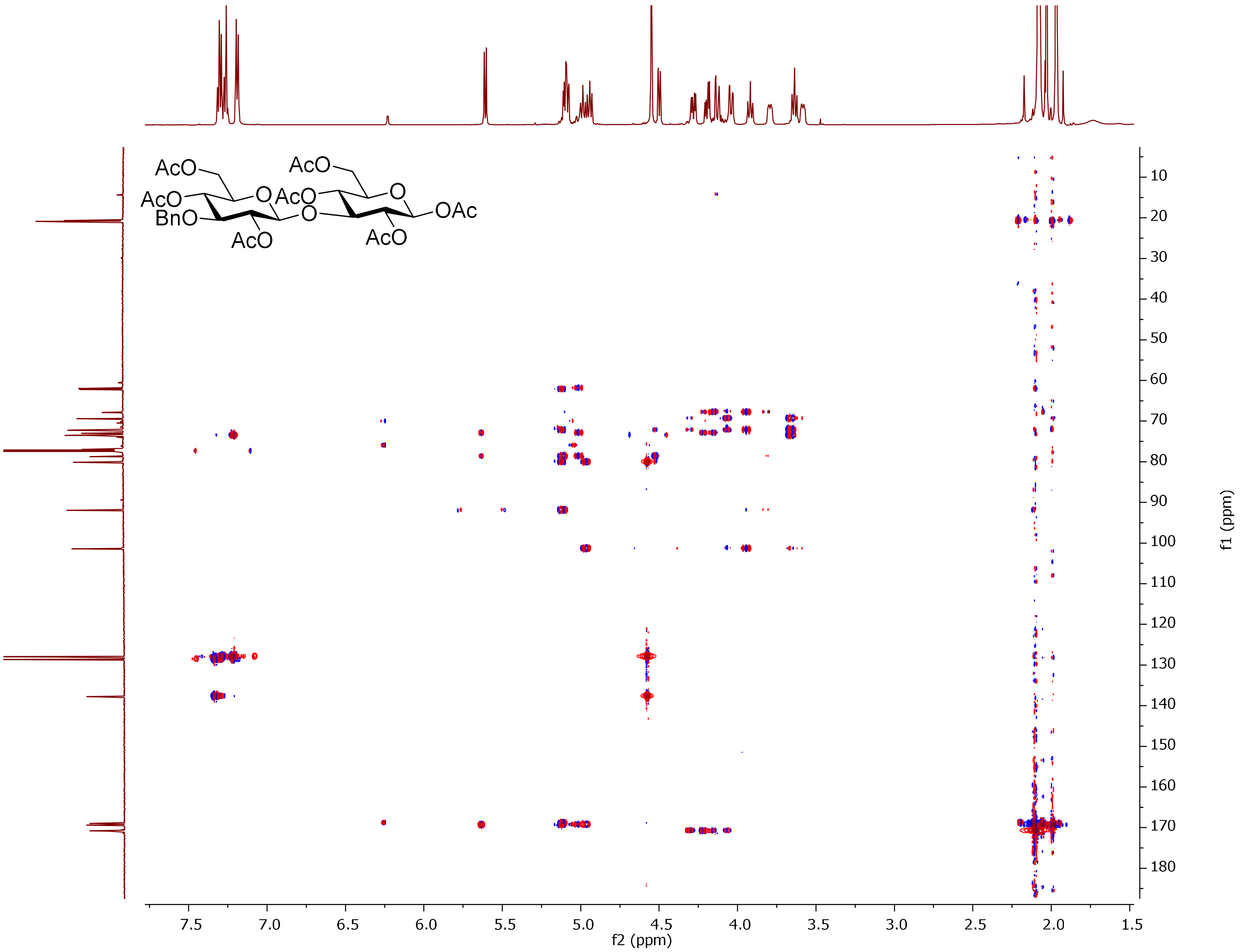

Crude ^{1}H NMR (600 MHz, $CDCl_3$) spectrum of **32** (Donor:Acceptor 1:1, 0.033 M, -25 ºC, TMSOTf, Set-1)

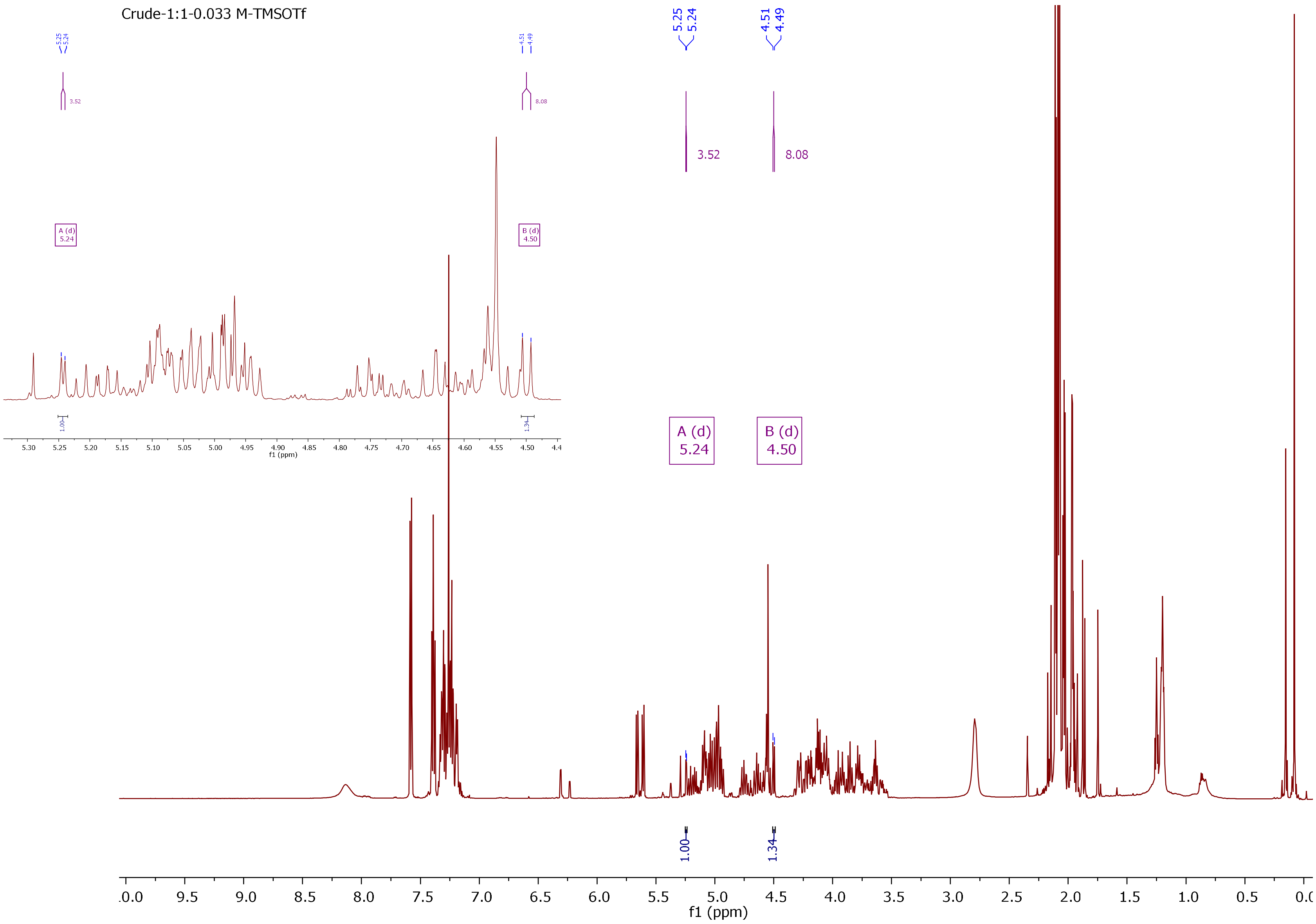

Crude ^1H NMR (600 MHz, $CDCl_3$) spectrum of **32** (Donor:Acceptor 1:1, 0.033 M, -25 ºC, TMSOTf, Set-2)

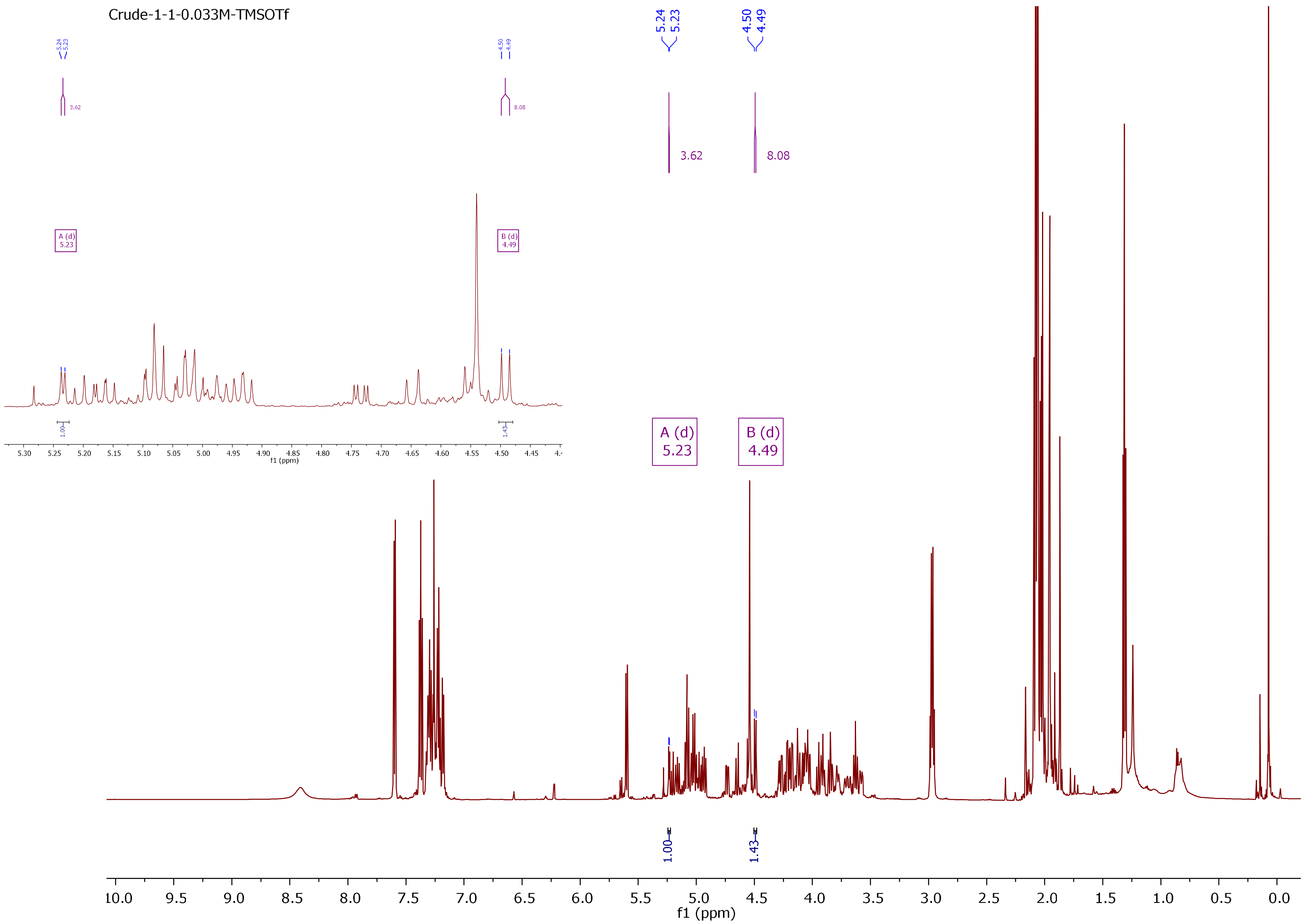

Crude ^{1}H NMR (600 MHz, $CDCl_3$) spectrum of **32** (Donor:Acceptor 1:1, 0.2 M, -25 ºC, TMSOTf, Set-1)

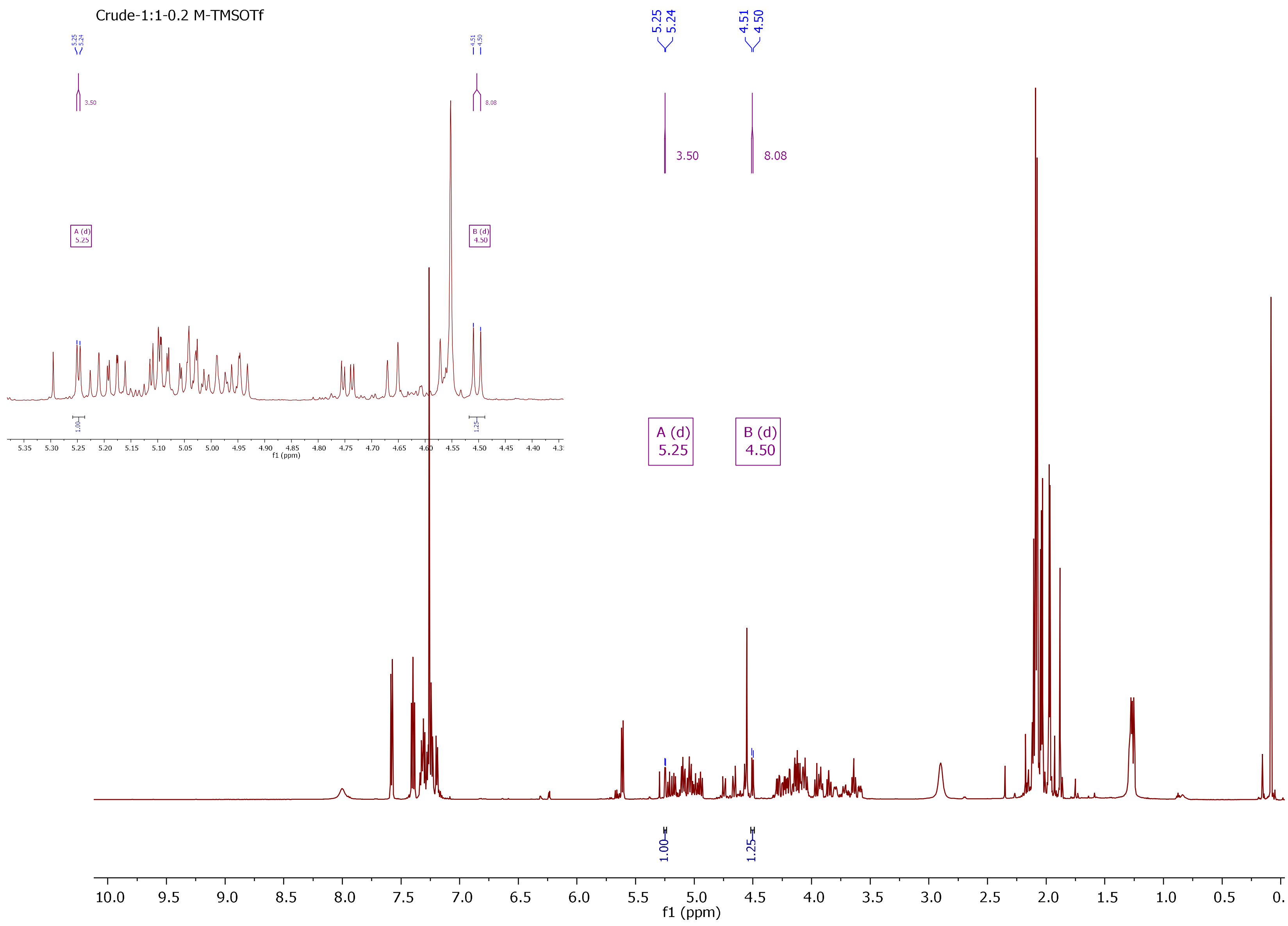

Crude ^{1}H NMR (600 MHz, $CDCl_3$) spectrum of **32** (Donor:Acceptor 1:1, 0.2 M, -25 ºC, TMSOTf, Set-2)

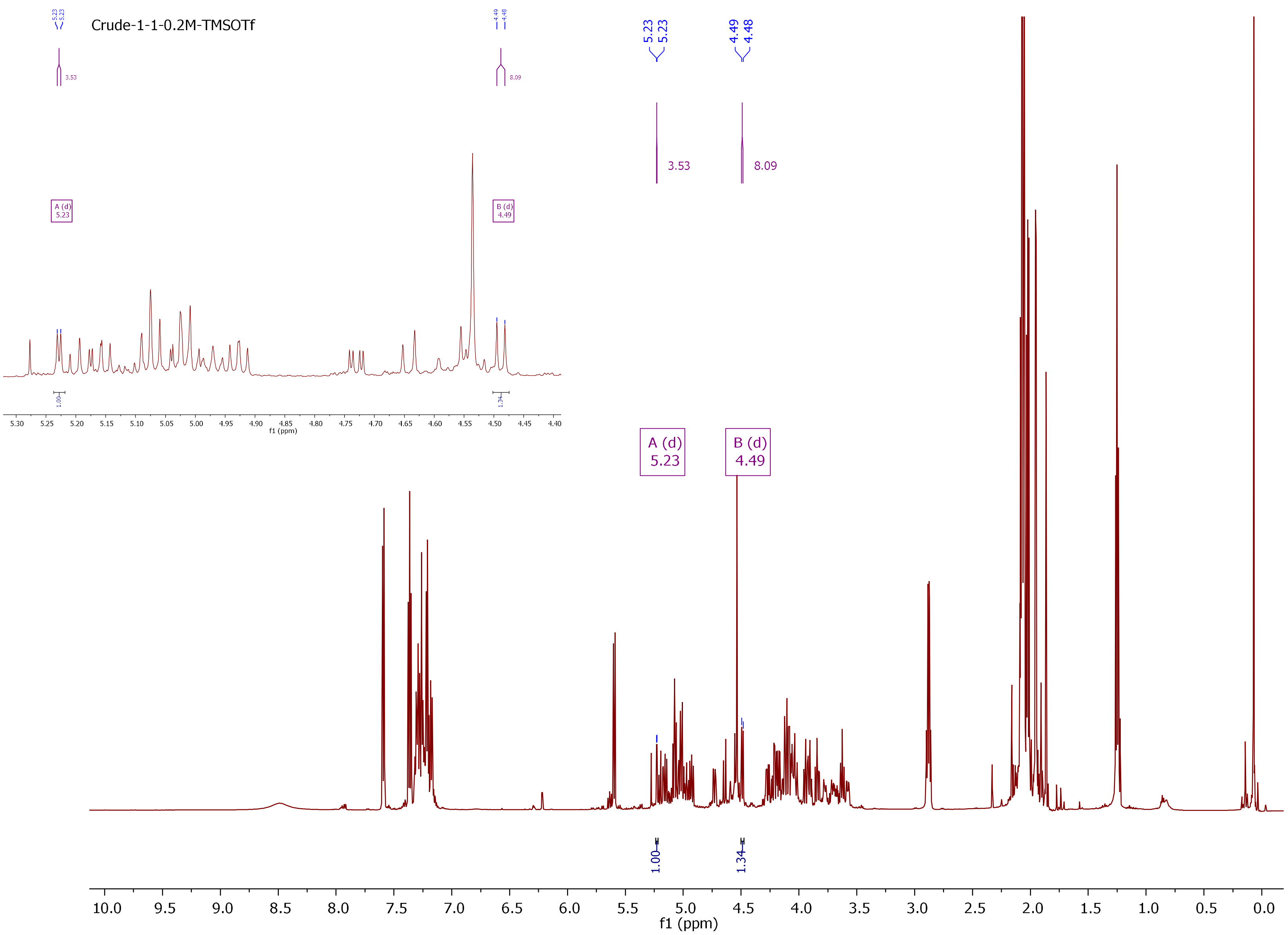

Crude ^{1}H NMR (600 MHz, $CDCl_3$) spectrum of **32** (Donor:Acceptor 1:1, 0.3 M, -25 ºC, TMSOTf, Set-1)

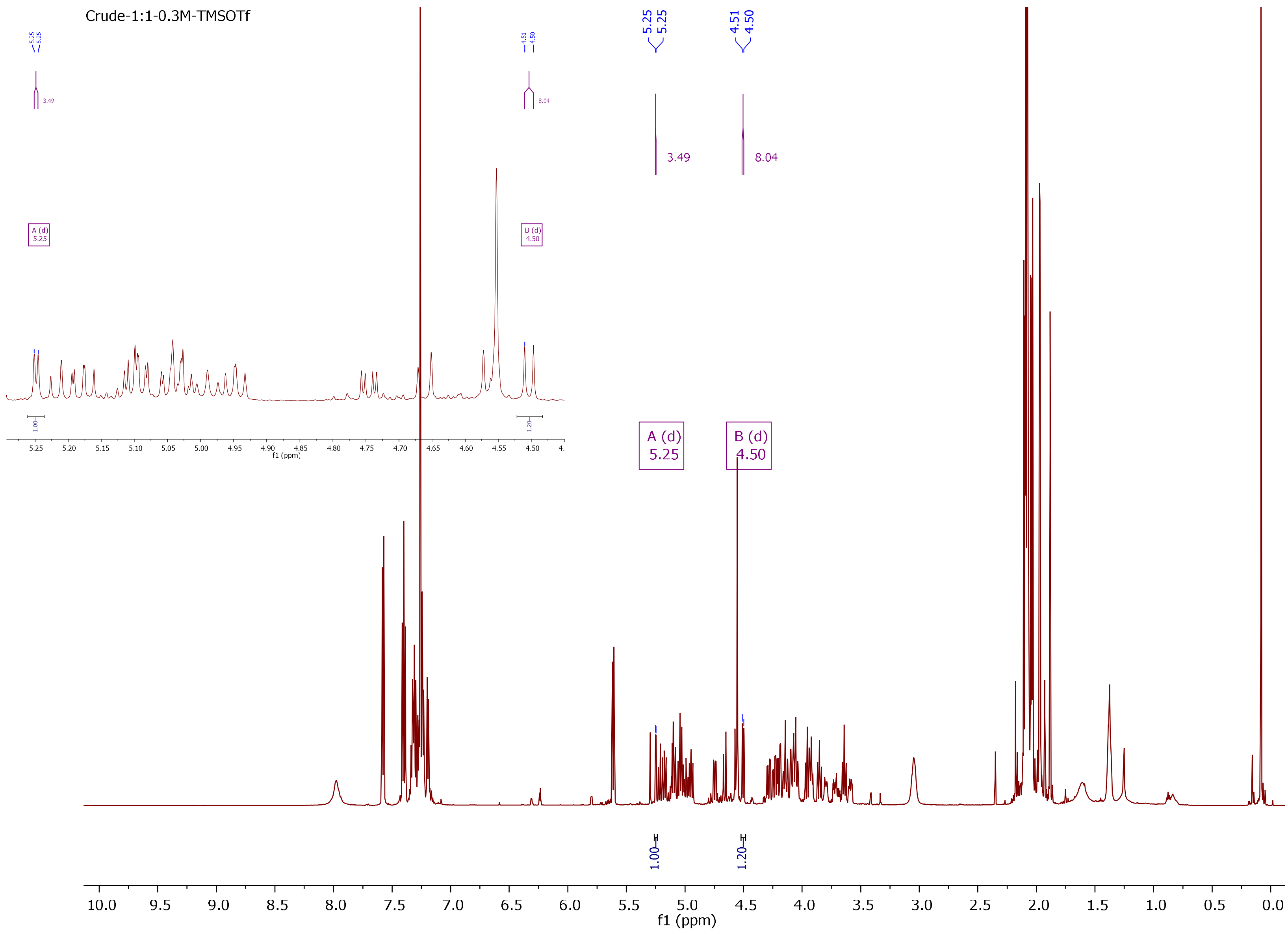

Crude ^{1}H NMR (600 MHz, $CDCl_3$) spectrum of **32** (Donor:Acceptor 1:1, 0.3 M, -25 ºC, TMSOTf, Set-2)

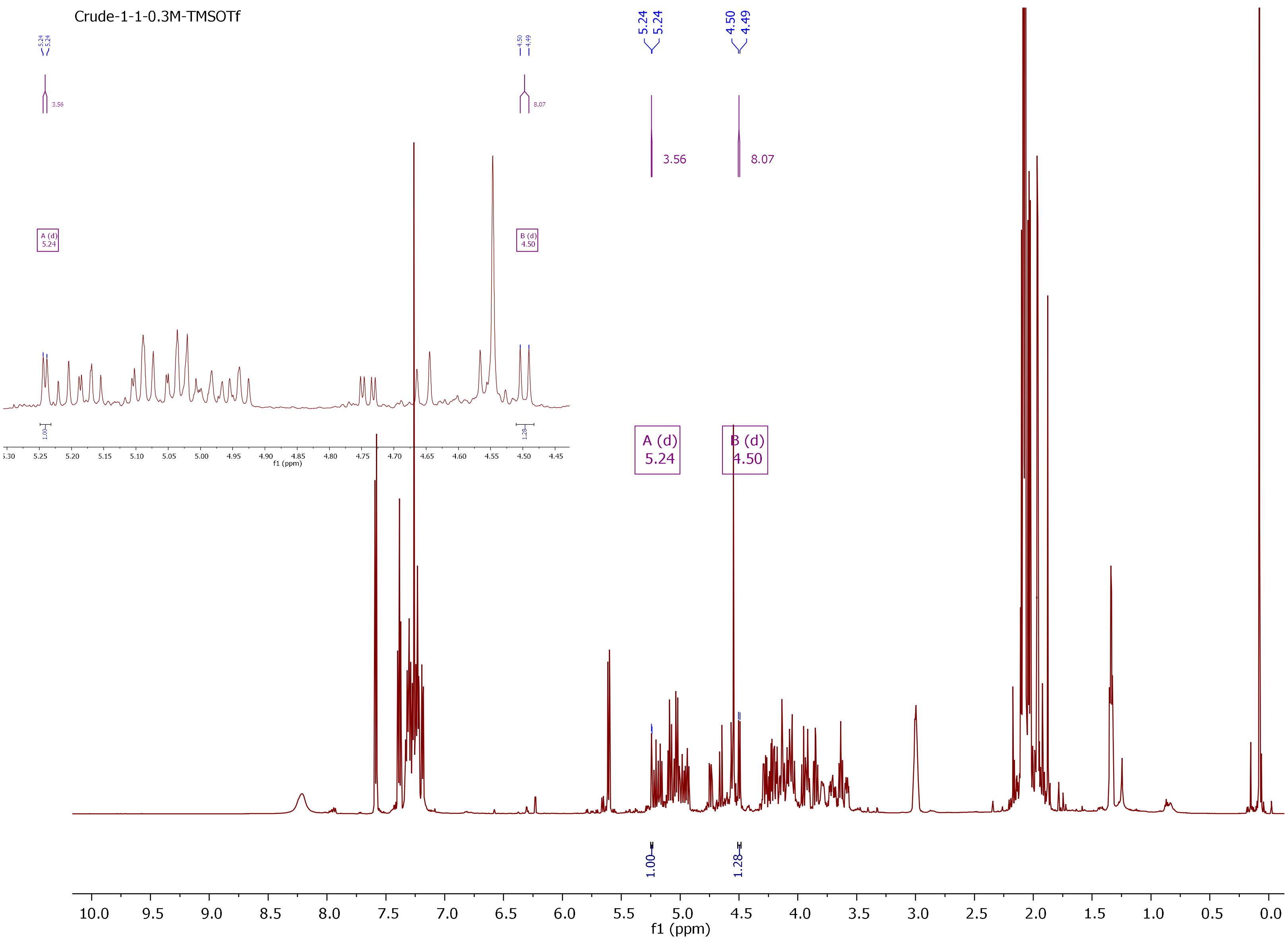

Crude ^{1}H NMR (600 MHz, $CDCl_3$) spectrum of **32** (Donor:Acceptor 1:1, 0.033 M, -25 ºC, $BF_3.OEt_2$, Set-1)

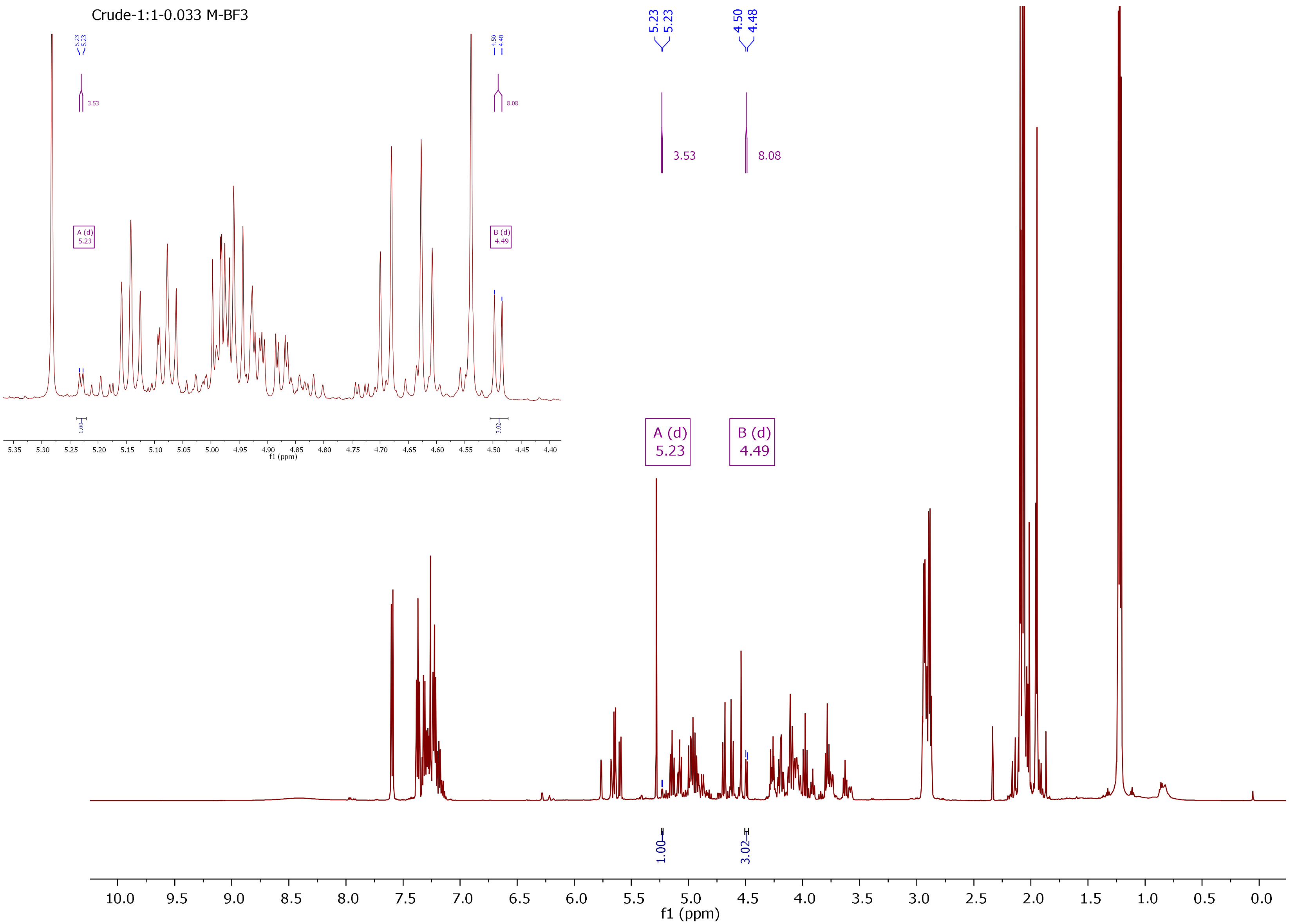

Crude ^{1}H NMR (600 MHz, $CDCl_3$) spectrum of **32** (Donor:Acceptor 1:1, 0.033 M, -25 ºC, $BF_3.OEt_2$, Set-2)

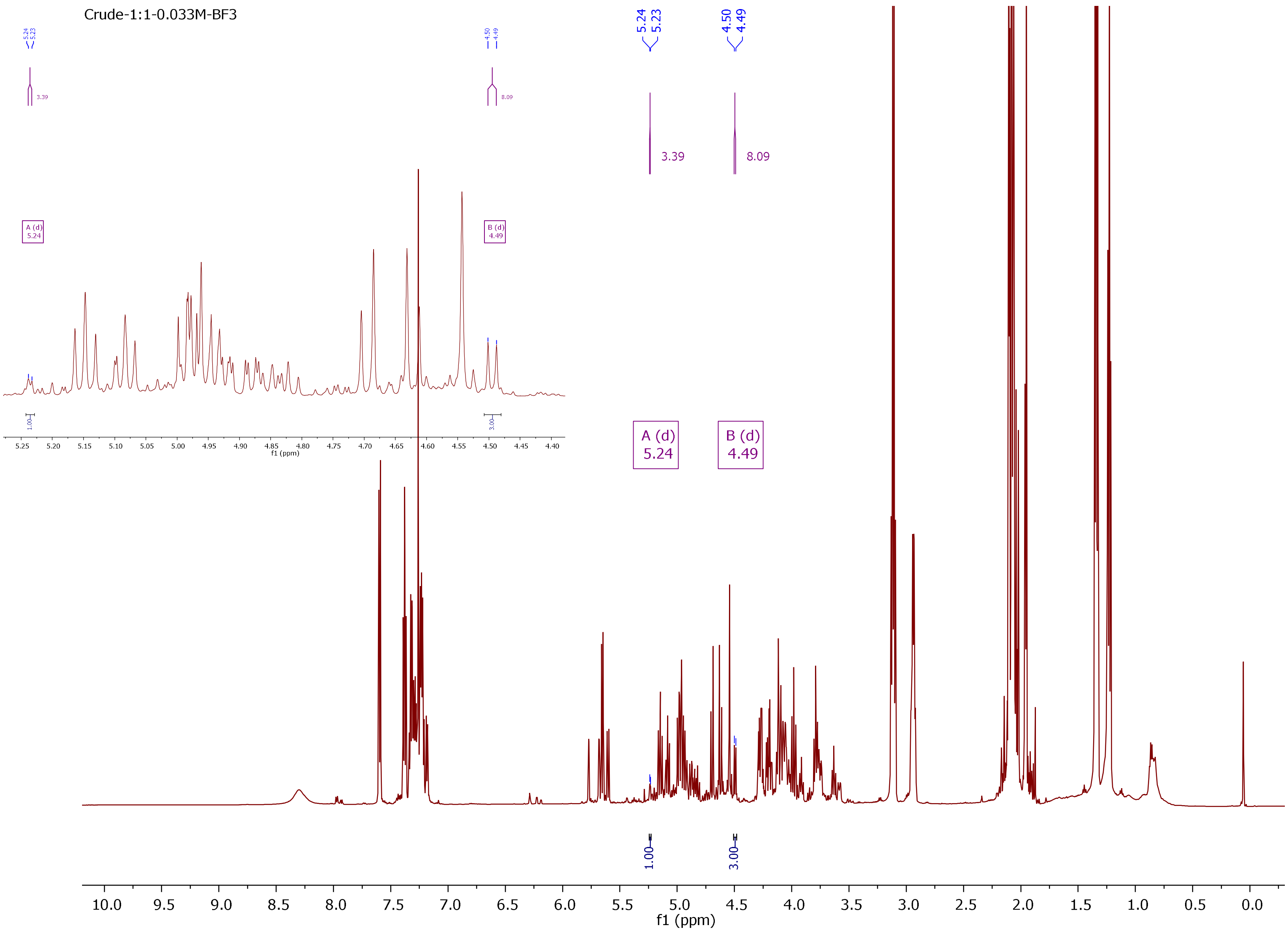

Crude ^{1}H NMR (600 MHz, $CDCl_3$) spectrum of **32** (Donor:Acceptor 1:1, 0.2 M, -25 ºC, $BF_3.OEt_2$, Set-1)

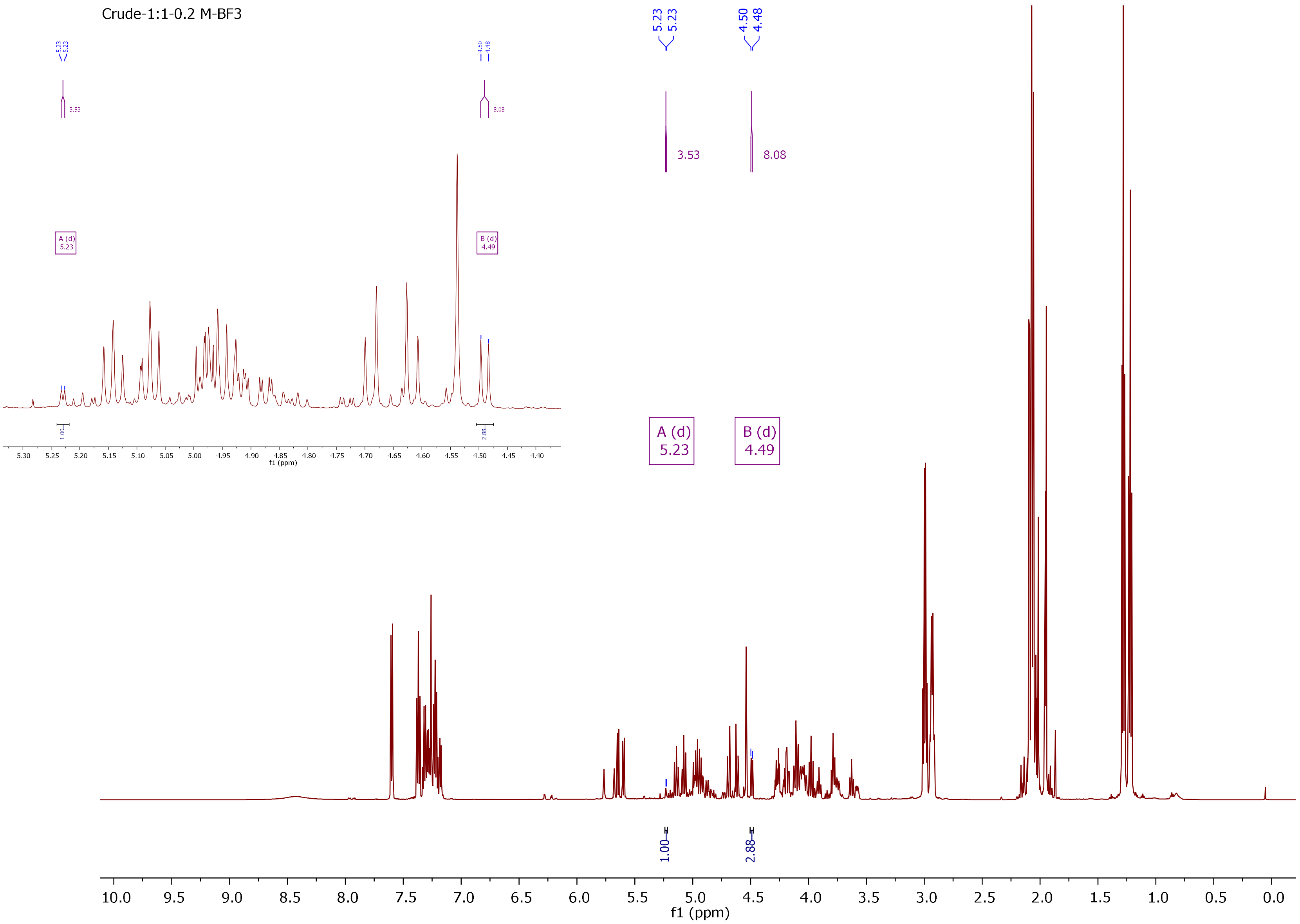

Crude ^{1}H NMR (600 MHz, $CDCl_3$) spectrum of **32** (Donor:Acceptor 1:1, 0.2 M, -25 ºC, $BF_3.OEt_2$, Set-2)

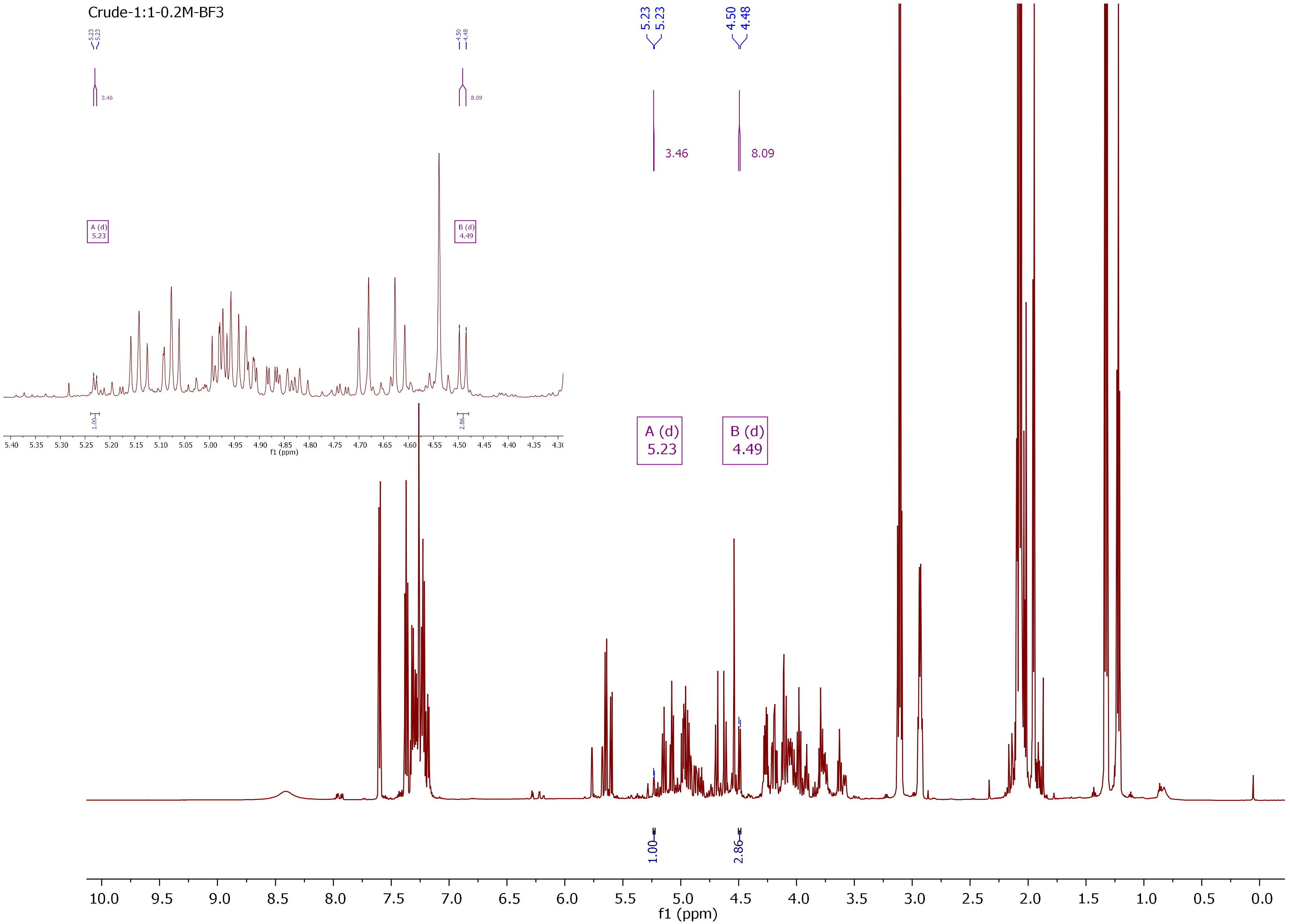

Crude ^{1}H NMR (600 MHz, $CDCl_3$) spectrum of **32** (Donor:Acceptor 1:1, 0.25M, -25 ºC, $BF_3.OEt_2$, Set-1)

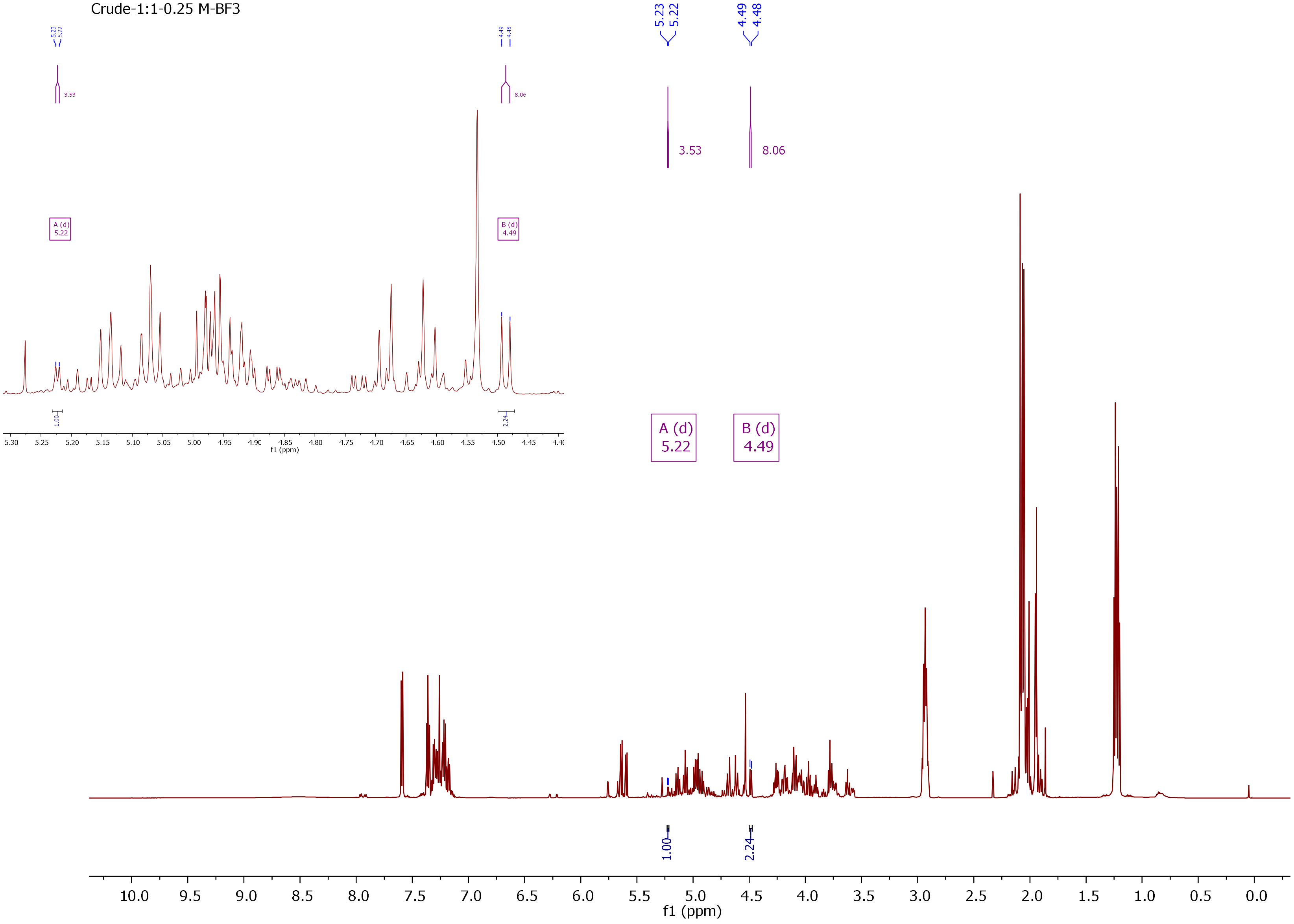

Crude ^{1}H NMR (600 MHz, $CDCl_3$) spectrum of **32** (Donor:Acceptor 1:1, 0.25M, -25 ºC, $BF_3.OEt_2$, Set-2)

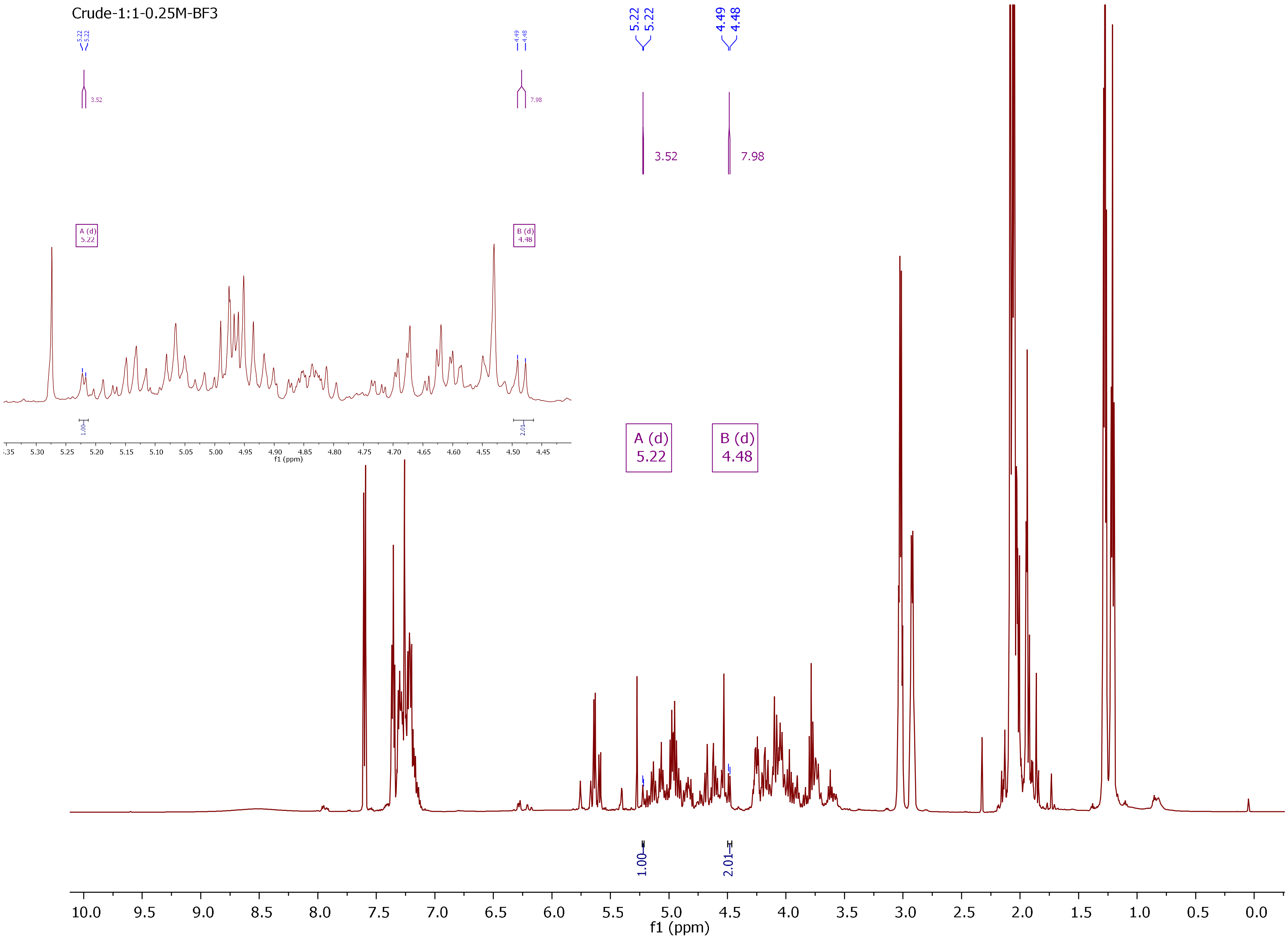

Crude ^{1}H NMR (600 MHz, $CDCl_3$) spectrum of **32** (Donor:Acceptor 1:1, 0.3 M, -25 ºC, $BF_3.OEt_2$, Set-1)

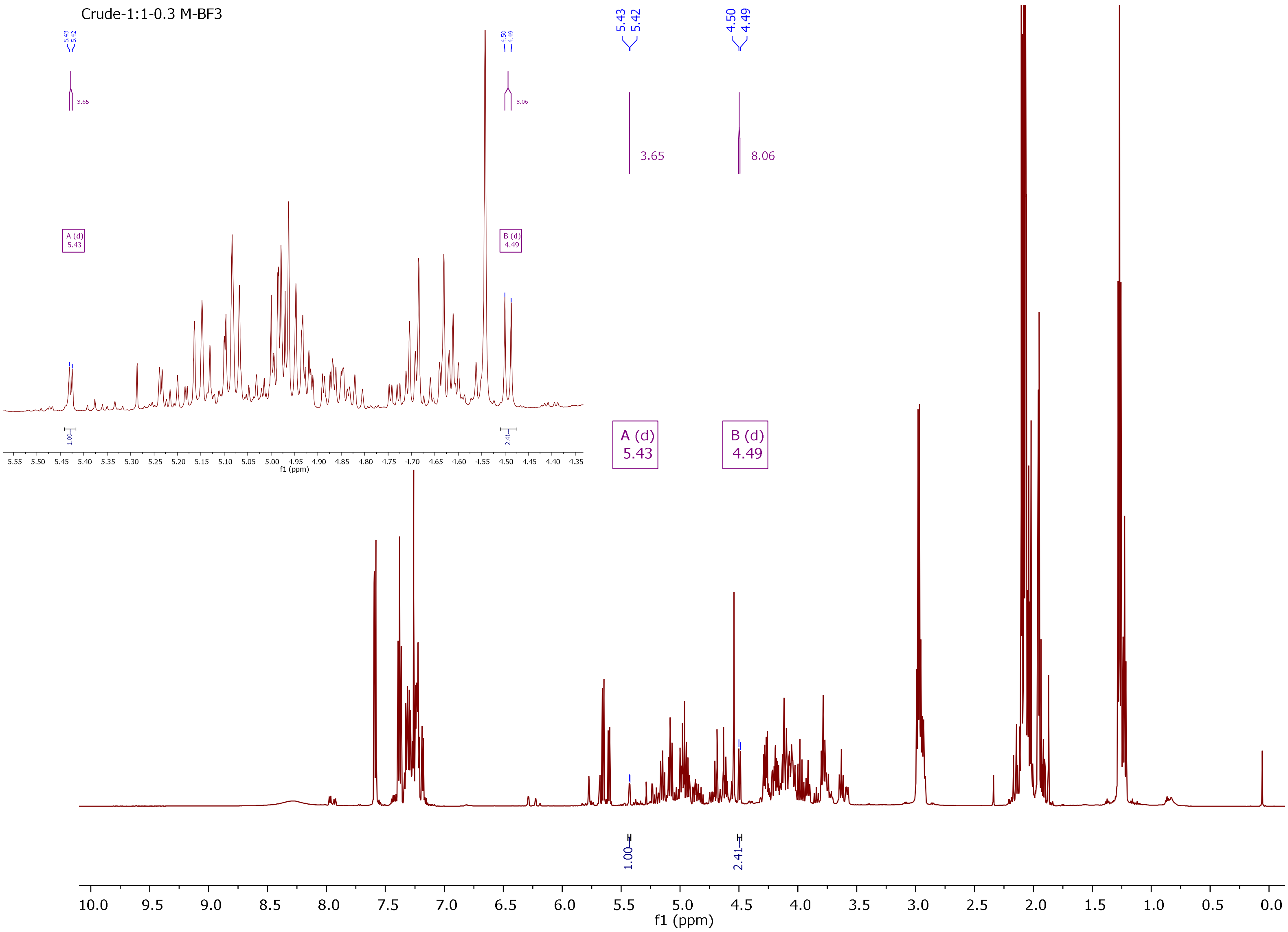

Crude ^{1}H NMR (600 MHz, $CDCl_3$) spectrum of **32** (Donor:Acceptor 1:1, 0.3 M, -25 ºC, $BF_3.OEt_2$, Set-2)

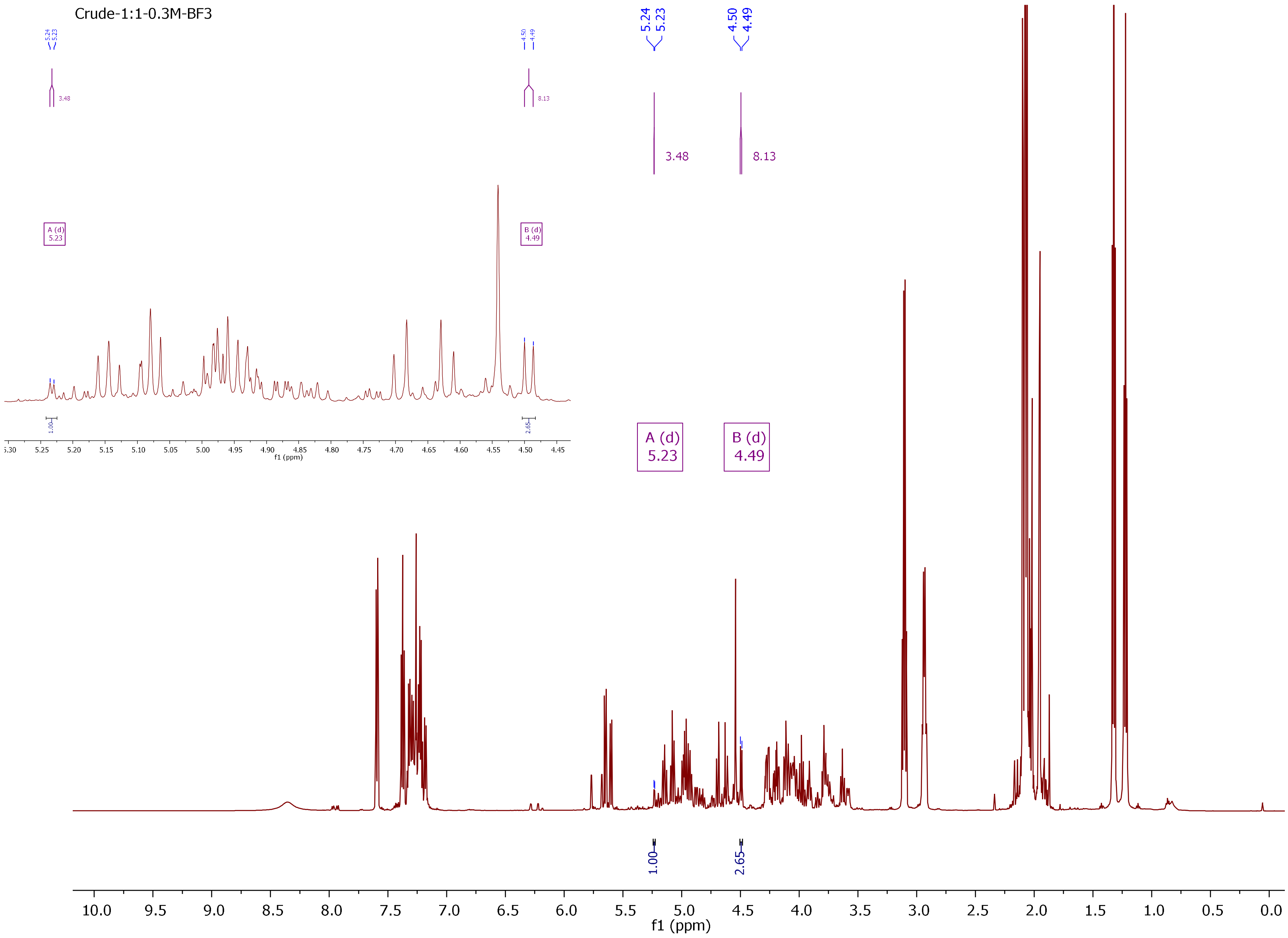

Crude ^{1}H NMR (600 MHz, $CDCl_3$) spectrum of **32** (Donor:Acceptor 1:1, 0.35 M, -25 ºC, $BF_3.OEt_2$, Set-1)

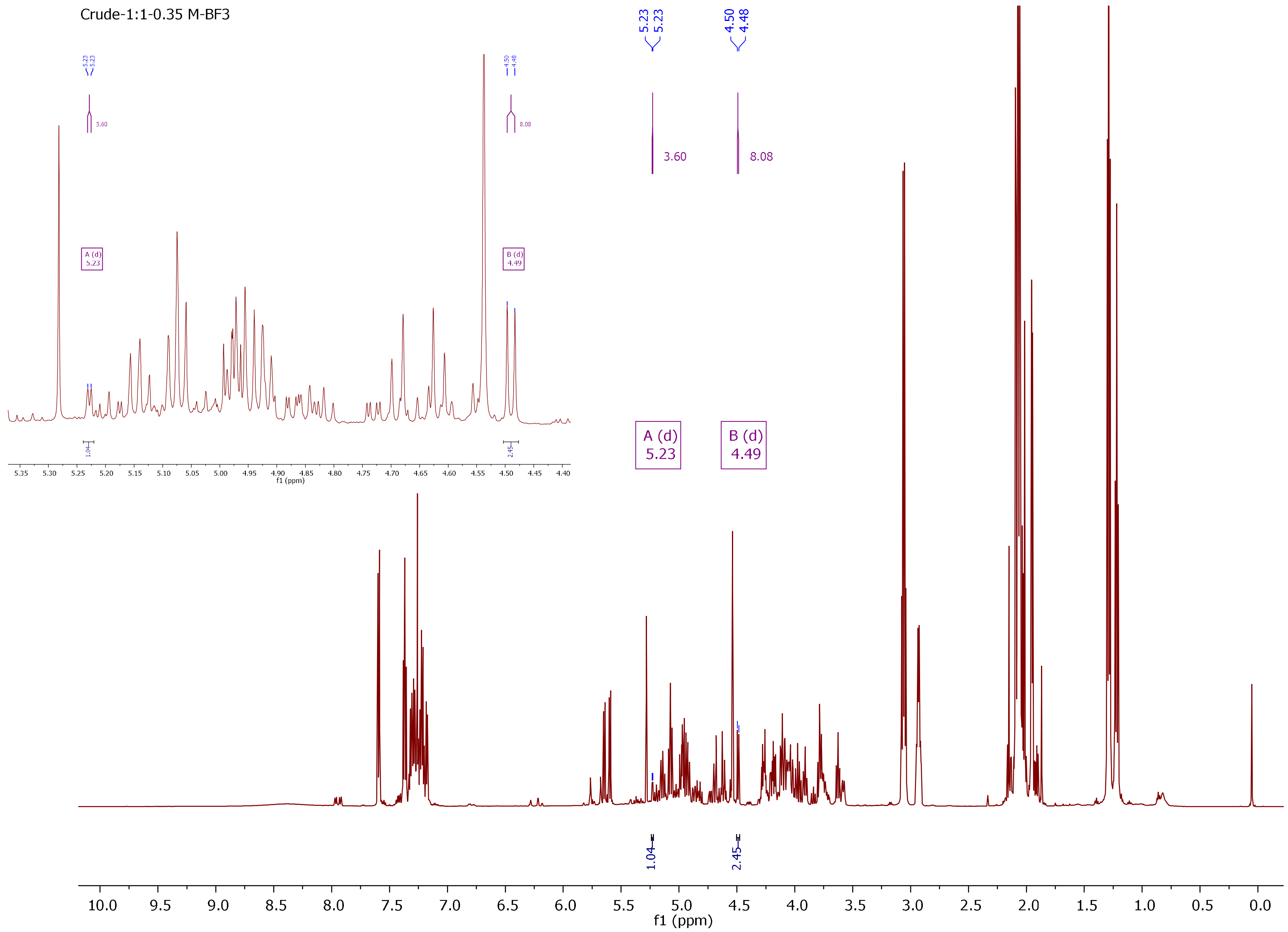

Crude ^{1}H NMR (600 MHz, $CDCl_3$) spectrum of **32** (Donor:Acceptor 1:1, 0.35 M, -25 ºC, $BF_3.OEt_2$, Set-2)

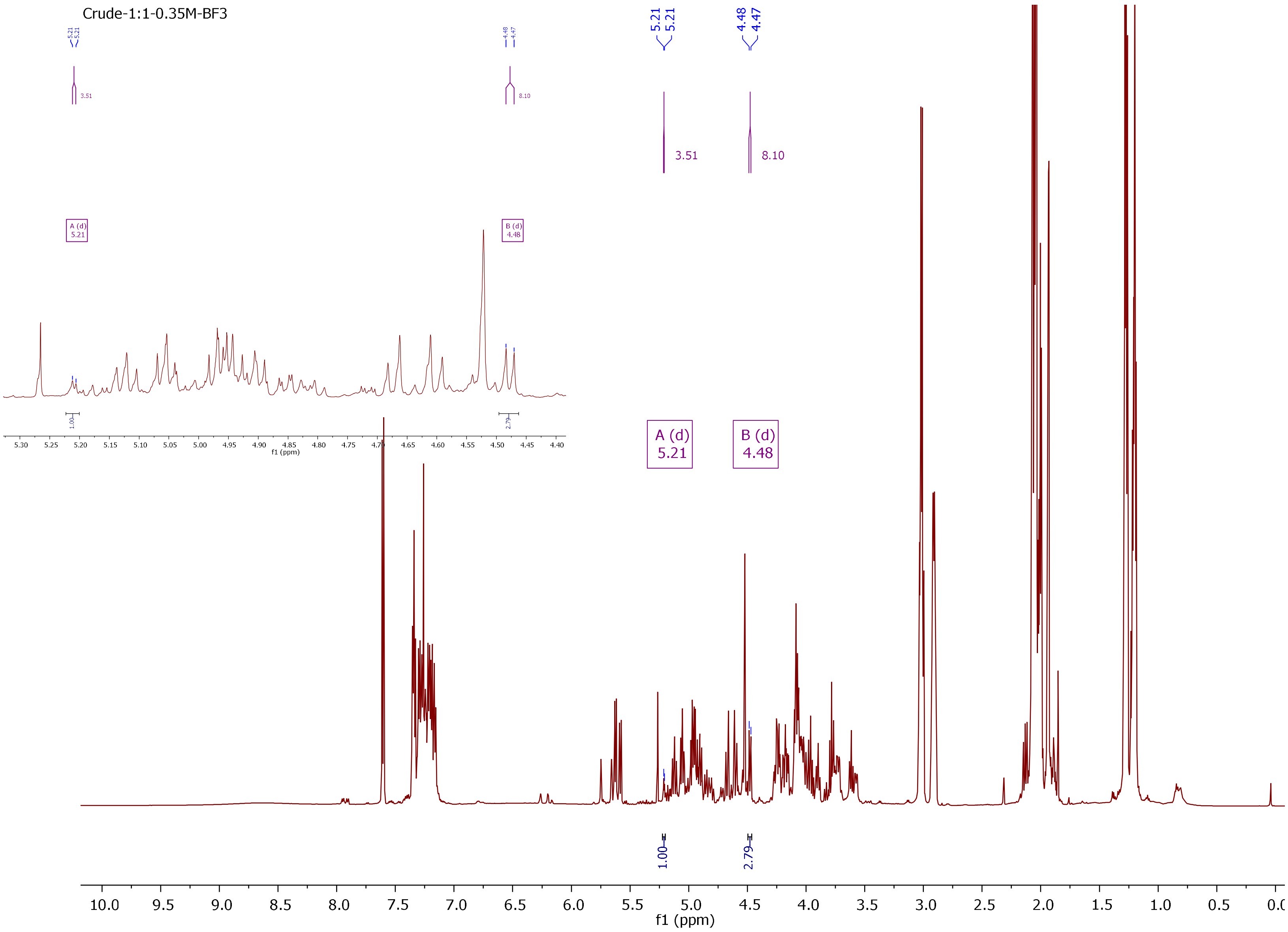

Crude ^{1}H NMR (600 MHz, $CDCl_3$) spectrum of **32** (Donor:Acceptor 1:1, 0.4 M, -25 ºC, $BF_3.OEt_2$, Set-1)

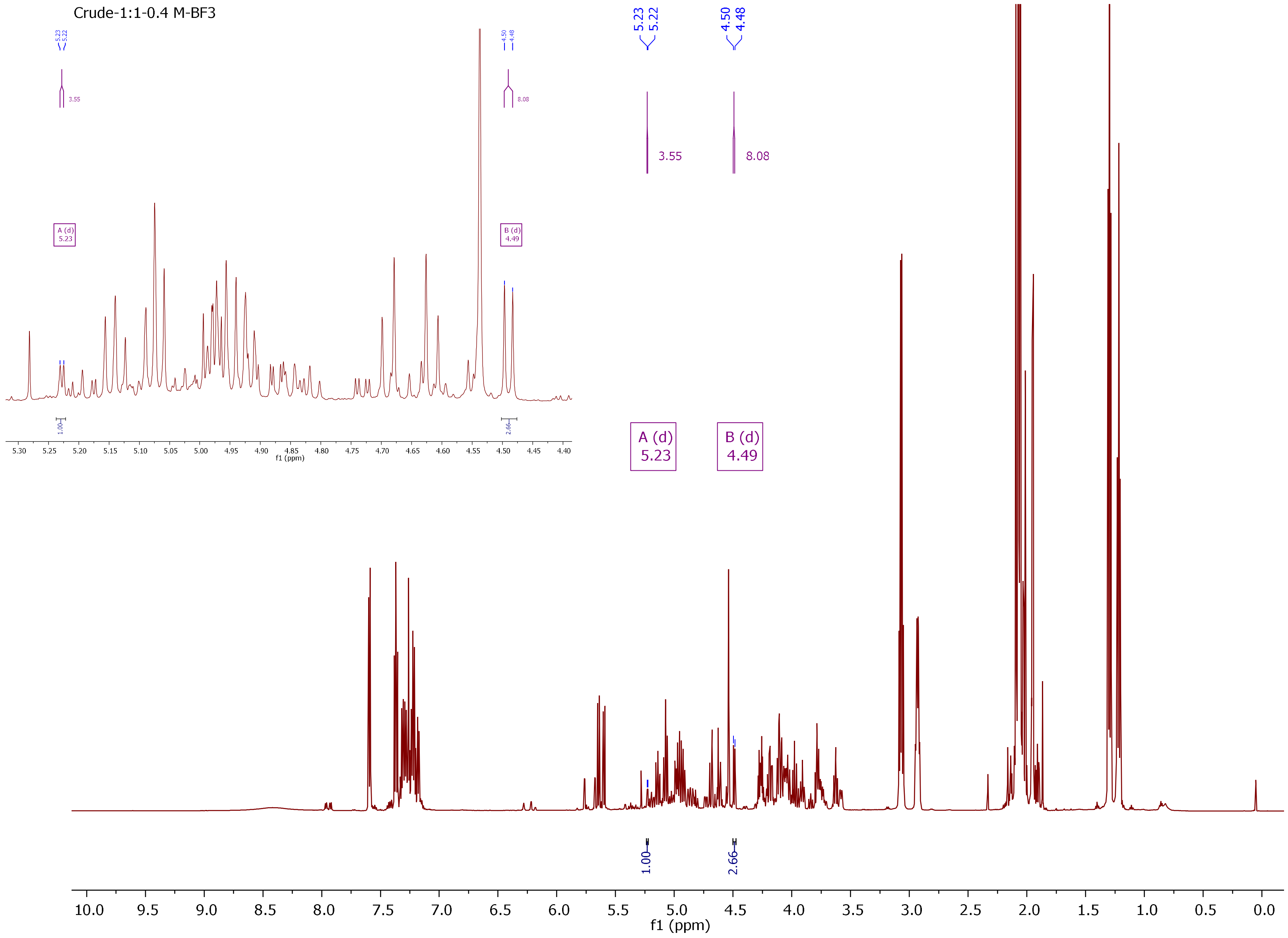

Crude ^{1}H NMR (600 MHz, $CDCl_3$) spectrum of **32** (Donor:Acceptor 1:1, 0.4 M, -25 ºC, $BF_3.OEt_2$, Set-2)

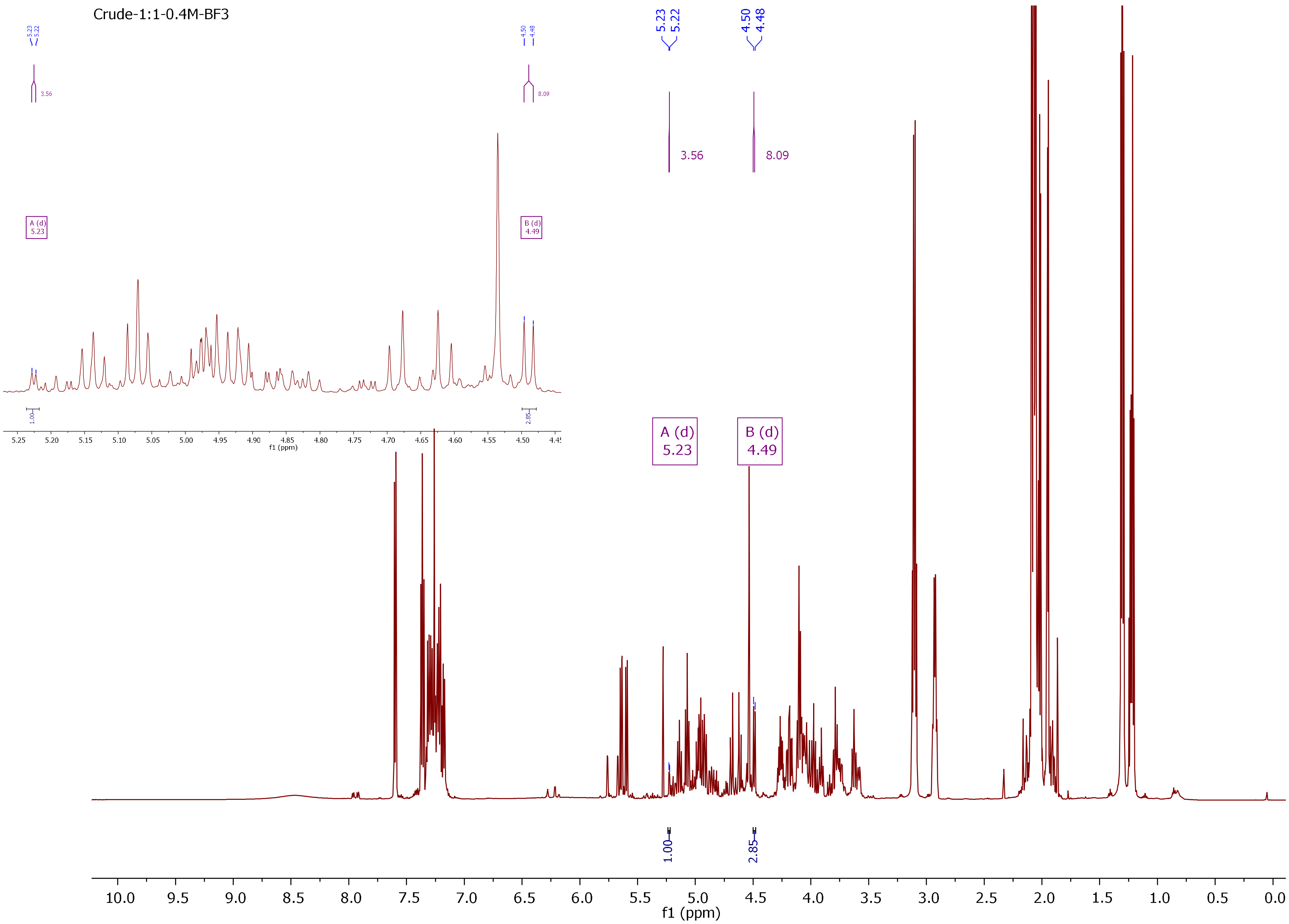

Supplement: Supplementary file 1 — ja4c14402_si_001.pdf [file ja4c14402_si_001.pdf]
